# Copper-Catalyzed Synthesis of 4-CF_3_-1,2,3-Triazoles: An Efficient and Facile Approach via Click Reaction

**DOI:** 10.3390/molecules29061191

**Published:** 2024-03-07

**Authors:** Tinghong Tang, Cuiting Chen, Xin Fu, Huilan Xu, Luyong Wu, Wenhao Chen

**Affiliations:** Key Laboratory of Tropical Medicinal Resource Chemistry of Ministry of Education, College of Chemistry and Chemical Engineering, Hainan Normal University, Haikou 571158, China; tangtinghong884@163.com (T.T.); cct13976904659@163.com (C.C.); 18876055422@163.com (X.F.); xhl010410@163.com (H.X.)

**Keywords:** 4-trifluoromethyl-1,2,3-triazole, 2-bromo-3,3,3-trifluoropropene, copper-catalyzed, click reaction, azide

## Abstract

Incorporation of a trifluoromethyl group with 1,2,3-triazoles motifs was described. We explored a click reaction approach for regioselective synthesis of 1-susbstituted-4-trifluoromethyl-1,2,3-triazoles in which 1,8-diazabicyclo[5.4.0]undec-7-ene (DBU) reacts with commercial 2-bromo-3,3,3-trifluoropropene (BTP) to form 3,3,3-trifloropropyne (TFP) in situ. Arising from merits associated with the availability and stability of BTP, and the high efficiencies of CuI/1,10-Phenanthroline (Phen)-catalyzed cycloaddition reactions of azides with alkynes, this readily performed click process takes place to form the target 1,2,3-triazoles in high yields, and with a wide azide substrate scope. The potential value of this protocol was demonstrated by its application to a gram-scale reaction.

## 1. Introduction

Since the time of its discovery in strategies to optimize the pharmacological properties of drug candidates [1,2,3,4], incorporation of the trifluoromethyl group has become a promising approach to discover new and/or improve existing bioactive compounds [5,6,7,8]. Although several valuable methods have been developed for the synthesis of trifluoromethyl-substituted compounds [9,10,11,12,13,14,15,16,17,18,19,20], methods that incorporate this group in an efficient and regiospecific manner remain in high demand [21,22,23,24,25,26,27], especially in the case of heterocyclic compounds [28,29,30,31,32,33,34,35].

**Scheme 1:**
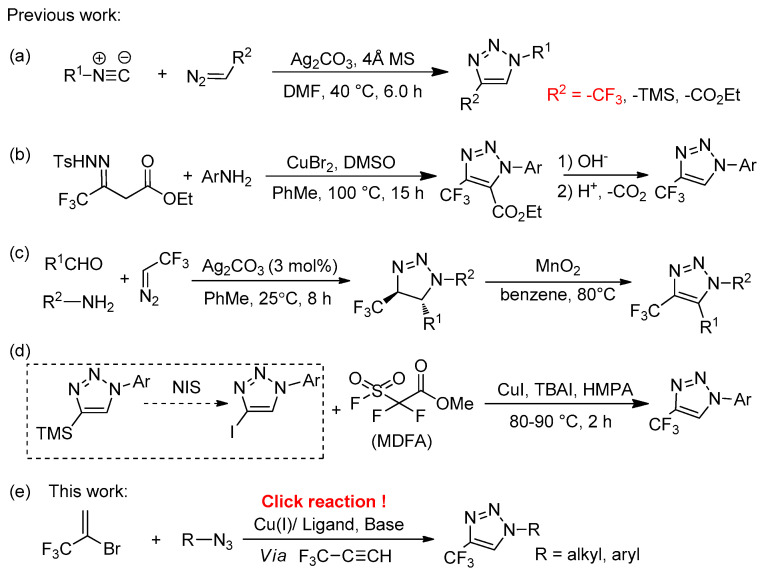
The synthesis of 4-CF_3_-1,2,3-triazoles.

In the past 20 years, 1,2,3-triazoles have been shown to be superior substances in drug discovery and biochemical applications [36,37,38,39,40,41]. As a result, considerable interest exists in devising strategies to prepare C4-, C5- or 1-trifluoromethyl-substituted 1,2,3-triazoles [42,43,44,45,46,47,48,49,50,51,52,53,54,55]. Currently, several noteworthy reactions exist that regioselectively generate 5-trifluoromethyl-1,2,3-triazoles [44,45,46,47,48,49,50,51,52], including trifluoromethylation of 5-iodotriazole [44,45]/5-stannyl triazoles [46], copper(I)-catalyzed interrupted click reaction [47,48], 1,3-dipolar cycloaddition [53] and annulation of perfluoroalkyl *N*-mesylhydrazones [49] or trifluoroacetimidoyl chlorides [50]. In contrast, much less attention has been given to the development of concise methods to produce 4-trifluoromethyl-1,2,3-triazoles. Among efforts aimed at this goal is the 2015 study by Ma and coworkers that demonstrated that silver-catalyzed cycloaddition reactions of arylisocyanides with 2,2,2-trifluorodiazoethane can be used to prepare 1-substituted-1,2,3-triazoles possessing a trifluoromethyl group at C4 (Scheme 1a) [54]. Unfortunately, use of alkylsubstituted isocyanides in this process gave rise to the formation of the corresponding 1,2,3-triazoles in low 28–54% yields. In 2021, Panish’s group developed a method to generate 4-trifluoromethyl-1,2,3-triazoles through copper-catalyzed reaction of 4,4,4-trifluoro-3-(2-tosylhdrazineylidene)butanoate with aromatic amines (Scheme 1b) [55]. The scope of substrate explored in this effort suggests that alkyl amines are not compatible with this approach. Although other multistep routes have been devised to prepare 4-trifluoromethyl-1,2,3-triazoles (Scheme 1c,d) [54,56,57,58,59], a facile method to produce 1-aryl- and 1-alkyl-substituted 4-trifluoromethyl-1,2,3-triazoles from low cost and commercially available CF_3_-building blocks is still lacking.

Click (cycloaddition) reactions of azides with alkynes are well-known, versatile protocols for construction of 1,2,3-triazoles [60,61,62]. Our continuing interest in the chemistry of 1,2,3-triazoles [63,64,65,66,67] and the importance of trifluoromethyl-substituted 1,2,3-triazoles encouraged us to evaluate the possibility of performing click reactions of 3,3,3-trifluoropropyne (TFP) with aryl- and alkyl-azides under mild and practical conditions. In contemplating possible methods to carry out these click reactions, we recognized that 2-bromo-3,3,3-trifluoropropylene (BTP) is a commercially available reagent [68,69,70,71,72], and that its [3+2]-cycloaddition reactions have been employed to produce CF_3_-substituted pyrazoles [73,74,75,76], -isoxazoles [77] and -pyrroles [78]. Particularly informative are the observations that BTP can be readily transformed to TFP by treatment with bases [79,80,81,82,83] like *i*-Pr_2_NLi and 1,8-diazabicyclo[5.4.0]undec-7-ene DBU, and that compared to gaseous TFP, BTP is an inflammable, storage-stable and easily handled liquid. Regrettably, it was inscrutably neglected to utilize BTP in click reactions to synthesize the 4-trifluoromethyl 1,2,3-triazoles.

Based on this information, we investigated copper-catalyzed click reactions of azides with BTP in the presence of DBU. In these processes, we expected that in the presence of DBU, BTP would be transformed to TFP, which then would undergo copper-catalyzed cycloaddition with azides to form the target 4-trifluoromethyl substituted-1,2,3-triazoles (Scheme 1e).

## 2. Results

The initial phase of this study was designed to evaluate the feasibility of the process described above. We observed that reaction of phenyl azide (**2a**) and BTP (**1**) in *N*,*N*-dimethylformamide (DMF) containing DBU or other bases at 100 °C did not generate the desired 1,2,3-triazole **3a** (Table 1, entry 1). The result indicated that 1,3-dipolar cycloaddition of BTP with azide with bases could not construct 1,2,3-triazoles. However, when CuI was included in the mixture, reaction occurred to form 1-phenyl-4-trifluoromethyl-1,2,3-triazole (**3a**) in a highly regioselective manner and <50% yields (^1^H- and ^13^C-NMR analysis) (entry 2). Moreover, when the copper ligand 1,10-phenanthroline (Phen) is included and CH_3_CN is utilized as solvent, the **3a**-forming click process takes place at temperatures >35 °C for 4 h but with a low 37% yield (entry 3). Importantly, at 65 °C, this reaction generates **3a** at an excellent 95% yield (entry 6). The results of a screening study showed that when less expensive copper salts (CuBr, CuCl, Cu(OAc)_2_, CuSO_4_·5H_2_O), and other ligands (see in SI), solvents (entries 10–11, Table 1) or bases (see in SI) are utilized, the process takes place with lower yields. As in the results of other copper-catalyzed click reactions, Cu(I)/Phen was shown to have high catalytic efficiency in this process. Two substituted 1,10-phenanthrolines are included in the mixture as ligands to give similar yields (entries 12 and 13). Furthermore, reduction in the amount of DBU to 2.0 eq. does not impact the yield (entry 15), and a decrease in the amount of Phen to 5 mol% lowers the yield only slightly (entry 16). The survey showed that the optimized condition for the process forming **3a** involves the use of CuI (10 mol%), Phen (10 mol%) and DBU (2.0 eq.) in CH_3_CN at 65 °C for 4 h.

Next, the aryl azide scope of this process carried out under the optimized conditions was evaluated. Firstly, aryl azides bearing both electron-donating and -withdrawing substituents at *para*-, *meta*- and *ortho*-positions react to produce corresponding 4-trifluoromethyl-1,2,3-triazoles in high to excellent yields (75–99%, Table 2). These results indicate that the efficiency of this transformation is not sensitive to electronic density and steric hindrance of the aromatic substituents. Furthermore, disubstituted aromatic azides participate efficiently in the click protocol (**3u**–**3aa**, 80–91%), and α-napththyl azide reacts smoothly to form triazole **3ab** in 84% yield.

To reveal its versatility, we determined if the scope of the click reaction includes alkyl azides. In the effort, we observed that a wide variety of alkyl azides react with BTP under the optimized reaction conditions to generate the corresponding 1-alkyl-4-trifluoromethyl-1,2,3-triazoles in modest to high yields (Table 3). Of particular interest was the contrast between the earlier finding that 4-trifluoromethyl-1-adamantyl-1,2,3-triazole is generated at 52% by reaction of trifluorodiazoethane and adamantyl isonitrile [53] and our observation that this substance (**4d**, Table 3) is produced at a 91% yield by reaction of BTP with adamantyl azide. Obviously, this copper-catalyzed click process is more efficient to produce 4-trifluoromethyl 1,2,3-triazoles with high compatibility with functional groups. In addition, both (1-azidoethyl) benzene and (2-azidoethyl) benzene react to form the corresponding triazoles **4e** and **4f**, and reactions of arylmethyl azides with BTP produce the corresponding products in excellent yields. Exceptions to this trend are found in reactions of 4-pyridylmethyl and *N*-phthalimidylethyl azide that take place less efficiently. The decreasing yields might be attributed to the coordination of the nitrogen-containing substrates with the copper catalyst to affect the catalytic capacity.

**Scheme 2:**
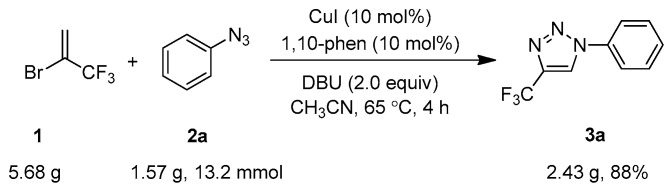
Gram-scale synthesis of **3a**.

To illustrate the value of the newly developed method further, a gram-scale reaction of **2a** with BTP was carried out under the standard conditions. Notably, this process formed 1,2,3-triazole **3a** at a relatively high isolated yield of 88% (Scheme 2).

**Scheme 3:**
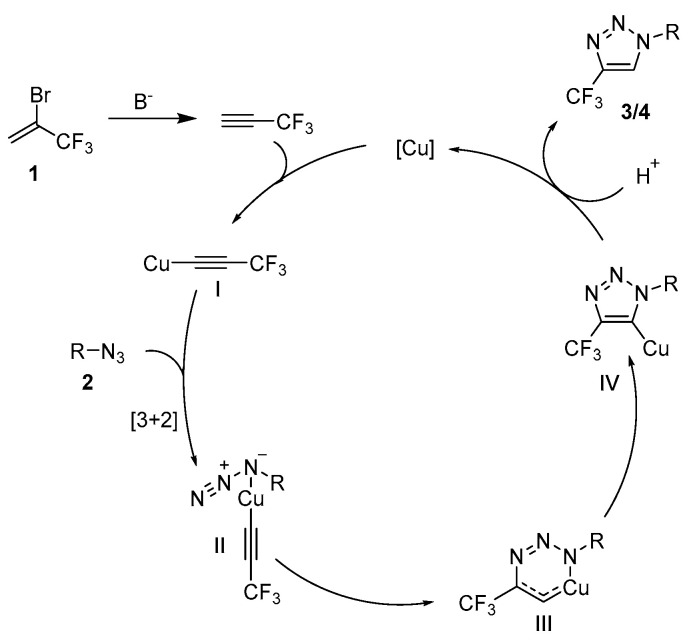
Proposed pathway for reaction of BTP with azides to form 1-substituted-4-trifluoromethyl-1,2,3-triazoles.

When heating the mixture of phenyl azide (**2a**), BTP (**1**) and bases, the product **3a** was not observed. We speculated that this process is not 1,3-dipolar cycloaddition of BTP with azide. Based upon experiments and the literature [81,82,83,84], a possible pathway was proposed as Scheme 3. Firstly, BTP is converted to TFP by treatment with DBU as the base. Then, TFP undergoes copper-catalyzed cycloaddition with azide to form trifluoromethyl 1,2,3-triazoles **3** or **4**.

## 3. Materials and Methods

### 3.1. General Information

Melting points were measured with a Beijing-Taike X-4 apparatus without correction. ^1^H NMR, ^19^F NMR and ^13^C NMR spectra were recorded using a Bruker Advance 400 MHz (Bruker, Faellanden, Switzerland) or a JEOL RESONANCE ECZ600R spectrometer (Akishima, Tokyo, Japan). Chemical shifts were reported in ppm from the solvent resonance as the internal standard (CDCl_3_: *δ*_H_ = 7.26 ppm, *δ*_C_ = 77.16 ppm). Coupling constants (*J*) are reported in Hertz (Hz). The following abbreviations are used to describe peak splitting patterns when appropriate: s = singlet, d = doublet, dd = double doublet, ddd = double doublet of doublets, t = triplet, dt = double triplet, q = quatriplet, m = multiplet. HRMS was obtained on an LCMS-IT-TOF (Thermo Fisher Scientific, Waltham, MA, USA). Reagents were received from commercial sources. Solvents were freshly dried and degassed according to the published procedures prior to use. Isolation was performed by column chromatography on silica gel (200~300 mesh) (Qingdao, China). ^1^H NMR, ^19^F NMR and ^13^C NMR spectra shown in the Supplementary Materials.

### 3.2. General Procedure for the Synthesis of Triazoles 3 and 4

A 10 mL crimp-seal veal was charged with an azide (**2**) (0.50 mmol), 2-bromo-3,3,3-trifluoropropylene (**1**) (219 mg, 1.25 mmol), acetonitrile (4.0 mL), CuI (9.5 mg, 10 mol%), 1,10-phenanthroline (9.0 mg, 10 mol%) and DBU (152 mg, 1.0 mmol). The mixture was then stirred at 65 °C for 4 h. After cooling to room temperature, the mixture was diluted with ethyl acetate (40 mL), and then washed with water (10 mL × 4). The organic phase was washed with saturated brine, dried by anhydrous Na_2_SO_4_ and concentrated in vacuo to give a residue that was subjected to silica gel column chromatography (petroleum ether/ethyl acetate) (V:V = 20:1 − 1:1.2) to give 1-substituted-4-trifluoromethyl-1,2,3-triazoles.

### 3.3. Data for Compounds of 4-Trifluoromethyl-1,2,3-Triazole

1-Phenyl-4-(trifluoromethyl)-*1H*-1,2,3-triazole (**3a**) [53].

White solid (101 mg, mp: 77–78 °C, yield: 95%). Isolated by column chromatography on silica gel (petroleum ether/ethyl acetate = 20:1, R*_f_* = 0.3). ^1^H NMR (400 MHz, CDCl_3_, ppm) *δ* 8.29 (s, 1H), 7.74 (dd, *J* = 7.5, 2.1 Hz, 2H), 7.61–7.49 (m, 3H). ^19^F NMR (377 MHz, CDCl_3_, ppm) *δ* −61.19 (s). ^13^C NMR (101 MHz, CDCl_3_, ppm) *δ* 139.57 (q, *J* = 39.6 Hz), 136.26, 130.10, 129.89, 121.73, 120.99, 120.49 (q, J = 267.9 Hz).

1-(o-Tolyl)-4-(trifluoromethyl)-*1H*-1,2,3-triazole (**3b**) [53].

Pale red oil (94 mg, yield: 83%). Isolated by column chromatography on silica gel (petroleum ether/ethyl acetate = 20:1, R*_f_* = 0.4). ^1^H NMR (400 MHz, CDCl_3_, ppm) *δ* 8.08 (s, 1H), 7.45 (td, *J* = 7.2, 1.6 Hz, 1H), 7.41–7.37 (m, 1H), 7.36–7.30 (m, 2H), 2.20 (s, 3H). ^19^F NMR (377 MHz, CDCl_3_, ppm) *δ* −61.01 (s). ^13^C NMR (101 MHz, CDCl_3_, ppm) *δ* 138.85 (q, *J* = 39.5 Hz), 135.56, 133.81, 131.28, 126.60, 124.81, 120.57 (q, *J* = 267.8 Hz), 17.75.

1-(*m*-Tolyl)-4-(trifluoromethyl)-*1H*-1,2,3-triazole (**3c**) [53].

Yellowish solid (109 mg, mp: 59–61 °C, yield: 96%). Isolated by column chromatography on silica gel (petroleum ether/ethyl acetate = 20:1, R*_f_* = 0.4). ^1^H NMR (400 MHz, CDCl_3_, ppm) *δ* 8.30 (d, *J* = 2.5 Hz, 1H), 7.56 (s, 1H), 7.51 (d, *J* = 8.5 Hz, 1H), 7.42 (t, *J* = 7.8 Hz, 1H), 7.31 (d, *J* = 7.5 Hz, 1H), 2.45 (s, 3H). ^19^F NMR (377 MHz, CDCl_3_, ppm) *δ* −61.20 (s). ^13^C NMR (101 MHz, CDCl_3_, ppm) *δ* 139.31 (q, *J* = 39.2 Hz), 136.23, 130.63, 129.87, 121.67 (d, *J* = 5.3 Hz), 120.52 (q, *J* = 267.8 Hz), 118.07, 21.42. HRMS (ESI): calcd. for C10H_8_F_3_N_3_ [M + Na]^+^: 250.0563, found: 250.0557.

1-(*p*-Tolyl)-4-(trifluoromethyl)-*1H*-1,2,3-triazole (**3d**) [53].

Yellowish solid (113 mg, mp: 94–95 °C, yield: 99%). Isolated by column chromatography on silica gel (petroleum ether/ethyl acetate = 20:1, R*_f_* = 0.3). ^1^H NMR (400 MHz, CDCl_3_, ppm) *δ* 8.26 (s, 1H), 7.63–7.57 (m, 2H), 7.34 (d, *J* = 8.2 Hz, 2H), 2.43 (s, 3H). ^19^F NMR (_3_77 MHz, CDCl_3_, ppm) *δ* −61.18 (s). ^13^C NMR (101 MHz, CDCl_3_, ppm) *δ* 140.23, 139.48 (q, *J* = 39.4 Hz), 134.00, 130.61, 121.61, 120.92, 120.54 (q, *J* = 267.8 Hz), 21.22.

1-(2-Fluorophenyl)-4-(trifluoromethyl)-*1H*-1,2,3-triazole (**3e**) [53].

White solid (87 mg, mp: 57–58 °C, yield: 75%). Isolated by column chromatography on silica gel (petroleum ether/ethyl acetate = 20:1, R*_f_* = 0.3). ^1^H NMR (400 MHz, CDCl_3_, ppm) *δ* 8.41 (s, 1H), 7.99–7.92 (m, 1H), 7.55–7.48 (m, 1H), 7.40–7.30 (m, 2H). ^19^F NMR (377 MHz, CDCl_3_, ppm) *δ* −61.25 (s), −123.77–123.86 (m). ^13^C NMR (101 MHz, CDCl_3_, ppm) *δ* 153.51 (d, J = 251.5 Hz), 139.44 (q, *J* = 39.8 Hz), 131.42 (d, *J* = 8.0 Hz), 125.66 (d, *J* = 3.8 Hz), 125.12, 124.61 (dd, *J* = 8.5, 2.9 Hz), 124.49 (q, *J* = 267.8 Hz), 117.43, 117.23.

1-(3-Fluorophenyl)-4-(trifluoromethyl)-*1H*-1,2,3-triazole (**3f**) [53].

White solid (90 mg, mp: 87–88 °C, yield: 78%). Isolated by column chromatography on silica gel (petroleum ether/ethyl acetate = 20:1, R*_f_* = 0.3). ^1^H NMR (600 MHz, CDCl_3_, ppm) *δ* 8.34 (d, *J* = 0.8 Hz, 1H), 7.57–7.53 (m, 3H), 7.24–7.21 (m, 1H). ^19^F NMR (565 MHz, CDCl_3_, ppm) *δ* −61.23 (s), −108.75 (dd, *J* = 14.0, 7.4 Hz). ^13^C NMR (151 MHz, CDCl_3_, ppm) *δ* 163.24 (d, *J* = 250.0 Hz), 139.89 (q, *J* = 39.8 Hz), 137.37 (d, *J* = 9.9 Hz), 131.71 (d, *J* = 8.9 Hz), 121.70, 120.34 (q, *J* = 268.0 Hz), 116.95 (d, *J* = 21.2 Hz), 116.37, 108.96 (d, *J* = 26.4 Hz).

1-(4-Fluorophenyl)-4-(trifluoromethyl)-*1H*-1,2,3-triazole (**3g**) [53].

White solid (112 mg, mp: 84–85 °C, yield: 97%). Isolated by column chromatography on silica gel (petroleum ether/ethyl acetate = 20:1, R*_f_* = 0.3). ^1^H NMR (600 MHz, CDCl_3_, ppm) *δ* 8.21 (s, 1H), 7.69–7.64 (m, 2H), 7.22–7.17 (m, 2H). ^19^F NMR (565 MHz, CDCl_3_, ppm) *δ* −61.16 (s), −110.16–−110.24 (m). ^13^C NMR (151 MHz, CDCl_3_, ppm) *δ* 163.15 (d, *J* = 250.9 Hz), 139.76 (q, *J* = 39.7 Hz), 132.57, 123.22 (d, *J* = 8.8 Hz), 121.90, 120.41 (q, *J* = 267.9 Hz), 117.21 (d, *J* = 23.4 Hz).

4-(Trifluoromethyl)-1-(3-(trifluoromethyl)phenyl)-*1H*-1,2,3-triazole (**3h**).

White solid (136 mg, mp: 87–89 °C, yield: 97%). Isolated by column chromatography on silica gel (petroleum ether/ethyl acetate = 20:1, R*_f_* = 0.4). ^1^H NMR (600 MHz, CDCl_3_, ppm) *δ* 8.41 (s, 1H), 8.04 (s, 1H), 7.99 (d, *J* = 8.0 Hz, 1H), 7.79 (d, *J* = 7.9 Hz, 1H), 7.75 (t, *J* = 7.9 Hz, 1H). ^19^F NMR (565 MHz, CDCl_3_, ppm) *δ* −61.26 (s), −62.86 (s). ^13^C NMR (151 MHz, CDCl_3_, ppm) *δ* 140.13 (q, *J* = 39.8 Hz), 136.67, 132.93 (q, *J* = 33.6 Hz), 131.07, 126.63 (d, *J* = 3.5 Hz), 124.20, 121.77 (d, *J* = 3.1 Hz), 121.48 (q, *J* = 90.9 Hz), 120.31 (q, *J* = 273.0 Hz), 118.09 (d, *J* = 3.8 Hz). HRMS (ESI): calcd. for C_10_H_5_F_6_N_3_ [M + H]^+^: 282.0460, found: 282.0454.

4-(Trifluoromethyl)-1-(4-(trifluoromethyl)phenyl)-*1H*-1,2,3-triazole (**3i**) [53].

White solid (122 mg, mp: 137–138 °C, yield: 87%). Isolated by column chromatography on silica gel (petroleum ether/ethyl acetate = 20:1, R*_f_* = 0.3). ^1^H NMR (600 MHz, CDCl_3_, ppm) *δ* 8.39 (s, 1H), 7.93 (d, *J* = 8.4 Hz, 2H), 7.86 (d, *J* = 8.4 Hz, 2H). ^19^F NMR (565 MHz, CDCl_3_, ppm) *δ* −61.24 (s), −62.70 (s). ^13^C NMR (151 MHz, CDCl_3_, ppm) *δ* 140.17 (q, *J* = 39.7 Hz), 138.72, 132.02 (q, *J* = 32.8 Hz), 127.56 (d, *J* = 3.4 Hz), 125.28 (q, *J* = 272.6 Hz), 123.82 (q, *J* = 261.38 Hz), 121.65, 121.16.

1-(4-(Trifluoromethoxy)phenyl)-4-(trifluoromethyl)-*1H*-1,2,3-triazole (**3j**) [59].

White solid (127 mg, mp: 115–116 °C, yield: 86%). Isolated by column chromatography on silica gel (petroleum ether/ethyl acetate = 20:1, R*_f_* = 0.3). ^1^H NMR (600 MHz, CDCl_3_, ppm) *δ* 8.26 (s, 1H), 7.77–7.72 (m, 2H), 7.36 (d, *J* = 8.7 Hz, 2H). ^19^F NMR (565 MHz, CDCl_3_, ppm) *δ* −57.94 (s), −61.24 (s). ^13^C NMR (151 MHz, CDCl_3_, ppm) *δ* 149.95, 139.97 (q, *J* = 39.8 Hz), 134.61, 123.04 (q, *J* = 259.7 Hz), 122.68, 122.61, 122.14 (q, *J* = 268.8 Hz), 121.79 (d, *J* = 3.0 Hz).

1-(4-Methoxyphenyl)-4-(trifluoromethyl)-*1H*-1,2,3-triazole (**3k**) [53].

Pale yellowish solid (104 mg, mp: 120–122 °C, yield: 86%). Isolated by column chromatography on silica gel (petroleum ether/ethyl acetate = 10:1, R*_f_* = 0.3). ^1^H NMR (600 MHz, CDCl_3_, ppm) *δ* 8.21 (s, 1H), 7.62 (d, *J* = 9.0 Hz, 2H), 7.04 (d, *J* = 9.0 Hz, 2H), 3.87 (s, 3H). ^19^F NMR (565 MHz, CDCl_3_, ppm) *δ* −61.05 (s). ^13^C NMR (151 MHz, CDCl_3_, ppm) *δ* 160.69, 139.42 (q, *J* = 39.5 Hz), 129.60, 122.73, 121.73, 120.55 (q, *J* = 267.7 Hz), 115.13, 55.79.

1-(2-Chlorophenyl)-4-(trifluoromethyl)-*1H*-1,2,3-triazole (**3l**) [53].

Yellowish oil (99 mg, yield: 80%). Isolated by column chromatography on silica gel (petroleum ether/ethyl acetate = 20:1, R*_f_* = 0.3). ^1^H NMR (600 MHz, CDCl_3_, ppm *δ* 8.32 (s, 1H), 7.65–7.60 (m, 2H), 7.55–7.47 (m, 2H). ^19^F NMR (565 MHz, CDCl_3_, ppm) *δ* −60.98 (s). ^13^C NMR (151 MHz, CDCl_3_, ppm) *δ* 138.89 (q, *J* = 39.7 Hz), 134.02, 131.78, 131.08, 128.79, 128.33, 127.96, 125.45 (d, *J* = 2.4 Hz), 120.45 (q, *J* = 267.9 Hz).

1-(3-Chlorophenyl)-4-(trifluoromethyl)-*1H*-1,2,3-triazole (**3m**) [53].

White solid (105 mg, mp: 76–78 °C, yield: 85%). Isolated by column chromatography on silica gel (petroleum ether/ethyl acetate = 20:1, R*_f_* = 0.3). ^1^H NMR (600 MHz, CDCl_3_, ppm) *δ* 8.33 (s, 1H), 7.80 (s, 1H), 7.65 (d, *J* = 8.0 Hz, 1H), 7.51 (d, *J* = 11.4 Hz, 2H). ^19^F NMR (565 MHz, CDCl_3_, ppm) *δ* −61.19 (s). ^13^C NMR (151 MHz, CDCl_3_, ppm) *δ* 139.89 (q, *J* = 39.7 Hz), 137.10, 136.07, 131.26, 130.05, 121.67, 121.37, 120.33 (q, *J* = 268.0 Hz), 119.03.

1-(4-Chlorophenyl)-4-(trifluoromethyl)-*1H*-1,2,3-triazole (**3n**) [53].

White solid (100 mg, mp: 126–128 °C, yield: 81%). Isolated by column chromatography on silica gel (petroleum ether/ethyl acetate = 20:1, R*_f_* = 0.4). ^1^H NMR (600 MHz, CDCl_3_, ppm) *δ* 8.32 (s, 1H), 7.71 (d, *J* = 9.0 Hz, 2H), 7.57 (d, *J* = 9.0 Hz, 2H), ^19^F NMR (565 MHz, CDCl_3_, ppm) *δ* −61.17 (s). ^13^C NMR (151 MHz, CDCl_3_, ppm) *δ* 139.83 (q, *J* = 39.7 Hz), 135.89, 134.75, 130.35, 122.24, 121.68, 120.36 (q, *J* = 267.9 Hz).

1-(2-Bromophenyl)-4-(trifluoromethyl)-*1H*-1,2,3-triazole (**3o**).

Yellowish oil (126 mg, yield: 86%). Isolated by column chromatography on silica gel (petroleum ether/ethyl acetate = 20:1, R*_f_* = 0.3). ^1^H NMR (600 MHz, CDCl_3_, ppm) *δ* 8.27 (s, 1H), 7.78 (dd, *J* = 8.1, 1.3 Hz, 1H), 7.57–7.51 (m, 2H), 7.46 (td, *J* = 8.1, 1.8 Hz, 1H). ^19^F NMR (565 MHz, CDCl_3_, ppm) *δ* −60.93 (s). ^13^C NMR (151 MHz, CDCl_3_, ppm) *δ* 138.77 (q, *J* = 39.7 Hz), 135.62, 134.21, 132.15, 128.88, 128.33, 125.51 (d, *J* = 2.0 Hz), 120.44 (q, *J* = 267.9 Hz), 118.67. HRMS (ESI): calcd. for C_9_H_5_BrF_3_N_3_ [M + Na]^+^: 313.9511, found: 313.9506.

1-(3-Bromophenyl)-4-(trifluoromethyl)-*1H*-1,2,3-triazole (**3p**).

White solid (140 mg, mp: 77–79 °C, yield: 96%). Isolated by column chromatography on silica gel (petroleum ether/ethyl acetate = 20:1, R*_f_* = 0.3). ^1^H NMR (600 MHz, CDCl_3_, ppm) *δ* 8.32 (s, 1H), 7.94 (s, 1H), 7.70 (dd, *J* = 8.1, 1.2 Hz, 1H), 7.64 (d, *J* = 8.1 Hz, 1H), 7.45 (t, *J* = 8.1 Hz, 1H). ^19^F NMR (565 MHz, CDCl_3_, ppm) *δ* −61.17 (s). ^13^C NMR (151 MHz, CDCl_3_, ppm) *δ* 139.89 (q, *J* = 39.8 Hz), 137.16, 132.99, 131.47, 124.19, 123.70, 121.69, 120.32 (q, *J* = 268.0 Hz), 119.54. HRMS (ESI): calcd. for C_9_H_5_BrF_3_N_3_ [M + Na]^+^: 313.9511, found: 313.9504.

1-(4-Bromophenyl)-4-(trifluoromethyl)-*1H*-1,2,3-triazole (**3q**) [53].

White solid (120 mg, mp: 134–136 °C, yield: 82%). Isolated by column chromatography on silica gel (petroleum ether/ethyl acetate = 20:1, R*_f_* = 0.3). ^1^H NMR (600 MHz, CDCl_3_, ppm) *δ* 8.31 (s, 1H), 7.70 (d, *J* = 8.4 Hz, 2H), 7.64 (d, *J* = 9.0 Hz, 2H). ^19^F NMR (565 MHz, CDCl_3_, ppm) *δ* −61.15 (s). ^13^C NMR (151 MHz, CDCl_3_, ppm) *δ* 139.87 (q, *J* = 39.7 Hz), 135.24, 133.35, 123.81, 122.45, 121.59, 120.34 (q, *J* = 268.0 Hz).

4-(4-(Tri*_f_*luoromethyl)-*1H*-1,2,3-triazol-1-yl)benzonitrile (**3r**) [59].

White solid (105 mg, mp: 160–161 °C, yield: 88%). Isolated by column chromatography on silica gel (petroleum ether/ethyl acetate = 10:1, R*_f_* = 0.3). ^1^H NMR (600 MHz, CDCl_3_, ppm) *δ* 8.42 (s, 1H), 7.98–7.94 (m, 2H), 7.92–7.89 (m, 2H). ^19^F NMR (565 MHz, CDCl_3_, ppm) *δ* −61.26 (s). ^13^C NMR (151 MHz, CDCl_3_, ppm) *δ* 140.32 (q, *J* = 40.1 Hz), 139.07, 134.30, 121.93 (q, *J* = 265.1 Hz), 121.59, 121.28, 117.45, 113.81.

1-(3-Nitrophenyl)-4-(trifluoromethyl)-*1H*-1,2,3-triazole (**3s**).

Yellowish solid (110 mg, mp: 141–142 °C, yield: 88%). Isolated by column chromatography on silica gel (petroleum ether/ethyl acetate = 20:1, R*_f_* = 0.3). ^1^H NMR (600 MHz, CDCl_3_, ppm) *δ* 8.64 (t, *J* = 2.1 Hz, 1H), 8.44 (s, 1H), 8.40 (dd, *J* = 8.2, 1.9 Hz, 1H), 8.21 (dd, *J* = 8.1, 2.0 Hz, 1H), 7.83 (t, *J* = 8.2 Hz, 1H). ^19^F NMR (565 MHz, CDCl_3_, ppm) *δ* −61.23 (s). ^13^C NMR (151 MHz, CDCl_3_, ppm) *δ* 149.15, 140.66 (q, *J* = 39.3 Hz), 137.01, 131.54, 126.55, 124.45, 121.95 (q, *J* = 271.6 Hz), 121.72, 115.96. HRMS (ESI): calcd. for C_9_H_5_F_3_N_4_O_2_ [M + H]^+^: 259.0437, found: 259.0435.

Methyl 2-(4-(trifluoromethyl)-*1H*-1,2,3-triazol-1-yl)benzoate (**3t**).

Yellowish oil (114 mg, yield: 84%). Isolated by column chromatography on silica gel (petroleum ether/ethyl acetate = 8:1, R*_f_* = 0.3). ^1^H NMR (600 MHz, CDCl_3_, ppm) *δ* 8.17 (s, 1H), 8.07 (dd, *J* = 7.7, 1.6 Hz, 1H), 7.71 (td, *J* = 7.7, 1.6 Hz, 1H), 7.66 (td, *J* = 7.7, 1.3 Hz, 1H), 7.49 (d, *J* = 7.8 Hz, 1H), 3.70 (s, 3H). ^19^F NMR (565 MHz, CDCl_3_, ppm) *δ* −60.87 (s). ^13^C NMR (151 MHz, CDCl_3_, ppm) *δ* 164.91, 138.61 (q, *J* = 39.5 Hz), 135.40, 133.26, 131.77, 130.94, 127.34, 125.66 (d, *J* = 2.1 Hz), 120.53 (q, *J* = 267.7 Hz), 52.73. HRMS (ESI): calcd. for C_11_H_8_F_3_N_3_O_2_ [M + Na]^+^: 294.0461, found: 294.0454.

1-(3-Fluoro-4-methylphenyl)-4-(trifluoromethyl)-*1H*-1,2,3-triazole (**3u**).

White solid (100 mg, mp: 111–113 °C, yield: 82%). Isolated by column chromatography on silica gel (petroleum ether/ethyl acetate = 20:1, R*_f_* = 0.4). ^1^H NMR (600 MHz, CDCl_3_, ppm) *δ* 8.29 (s, 1H), 7.47 (d, *J* = 9.6 Hz, 1H), 7.42 (d, *J* = 8.3 Hz, 1H), 7.37 (t, *J* = 7.9 Hz, 1H), 2.35 (s, 3H). ^19^F NMR (565 MHz, CDCl_3_, ppm) *δ* −61.20 (s), −112.68–−112.79 (m). ^13^C NMR (151 MHz, CDCl_3_, ppm) *δ* 161.46 (d, *J* = 248.3 Hz), 139.71 (q, *J* = 39.7 Hz), 135.05 (d, *J* = 9.9 Hz), 132.79 (d, *J* = 6.0 Hz), 127.16 (d, *J* = 17.3 Hz), 121.61, 120.40 (q, *J* = 268.0 Hz), 116.06 (d, *J* = 3.6 Hz), 108.52 (d, *J* = 27.4 Hz), 14.47 (d, *J* = 2.9 Hz). HRMS (ESI): calcd. for C_10_H_7_F_4_N_3_ [M + H]^+^: 246.0649, found: 246.0644.

1-(3-Bromo-4-methylphenyl)-4-(trifluoromethyl)-*1H*-1,2,3-triazole (**3v**).

Yellowish solid (139 mg, mp: 87–89 °C, yield: 91%). Isolated by column chromatography on silica gel (petroleum ether/ethyl acetate = 20:1, R*_f_* = 0.4). ^1^H NMR (600 MHz, CDCl_3_, ppm) *δ* 8.28 (s, 1H), 7.94 (d, *J* = 2.2 Hz, 1H), 7.59 (dd, *J* = 8.2, 2.2 Hz, 1H), 7.42 (d, *J* = 8.2 Hz, 1H), 2.47 (s, 3H). ^19^F NMR (565 MHz, CDCl_3_, ppm) *δ* −61.13 (s). ^13^C NMR (151 MHz, CDCl_3_, ppm) *δ* 140.20, 139.74 (q, *J* = 39.7 Hz), 134.85, 131.91, 125.80, 124.77, 121.62, 120.38 (q, *J* = 268.0 Hz), 119.69, 22.79. HRMS (ESI): calcd. for C_10_H_7_BrF_3_N_3_ [M + Na]^+^: 327.9668, found: 327.9661.

1-(3-Chloro-4-fluorophenyl)-4-(trifluoromethyl)-*1H*-1,2,3-triazole (**3w**) [53].

White solid (112 mg, mp: 70–71 °C, yield: 84%). Isolated by column chromatography on silica gel (petroleum ether/ethyl acetate = 20:1, R*_f_* = 0.4). ^1^H NMR (600 MHz, CDCl_3_, ppm) *δ* 8.31 (s, 1H), 7.87 (dd, *J* = 6.1, 2.7 Hz, 1H), 7.66 (ddd, *J* = 8.9, 3.8, 2.8 Hz, 1H), 7.36 (t, *J* = 8.5 Hz, 1H). ^19^F NMR (565 MHz, CDCl_3_, ppm) *δ* −61.22 (s), −112.22 (dd, *J* = 13.1, 7.3 Hz). ^13^C NMR (151 MHz, CDCl_3_, ppm) *δ* 158.75 (d, *J* = 253.5 Hz), 139.99 (q, *J* = 39.8 Hz), 132.79 (d, *J* = 3.2 Hz), 123.77, 123.16 (d, *J* = 19.4 Hz), 122.04 (q, *J* = 268.18 Hz), 121.87, 120.96 (d, *J* = 7.8 Hz), 118.13 (d, *J* = 23.0 Hz).

1-(3-Bromo-4-fluorophenyl)-4-(trifluoromethyl)-*1H*-1,2,3-triazole (**3x**).

Yellowish solid (126 mg, mp: 95–98 °C, yield: 81%). Isolated by column chromatography on silica gel (petroleum ether/ethyl acetate = 20:1, R*_f_* = 0.4). ^1^H NMR (600 MHz, CDCl_3_, ppm) *δ* 8.30 (s, 1H), 8.01 (dd, *J* = 5.7, 2.7 Hz, 1H), 7.71 (ddd, *J* = 8.9, 3.9, 2.7 Hz, 1H), 7.33 (dd, *J* = 8.8, 7.7 Hz, 1H). ^19^F NMR (565 MHz, CDCl_3_, ppm) *δ* −61.19 (s), −104.28 (dd, *J* = 13.0, 6.5 Hz). ^13^C NMR (151 MHz, CDCl_3_, ppm) *δ* 159.78 (d, *J* = 252.0 Hz), 139.98 (q, *J* = 39.8 Hz), 133.04, 126.55, 122.01–121.62 (m), 120.26 (q, *J* = 268.0 Hz), 117.98, 117.82, 110.80 (d, *J* = 23.0 Hz). HRMS (ESI): calcd. for C_9_H_4_BrF_4_N_3_ [M + H]^+^: 309.9597, found: 309.9592.

1-(4-Bromo-3-fluorophenyl)-4-(trifluoromethyl)-*1H*-1,2,3-triazole (**3y**).

Yellowish solid (127 mg, mp: 115–117 °C, yield: 82%). Isolated by column chromatography on silica gel (petroleum ether/ethyl acetate = 20:1, R*_f_* = 0.4). ^1^H NMR (600 MHz, CDCl_3_, ppm) *δ* 8.34 (s, 1H), 7.77 (dd, *J* = 8.6, 7.1 Hz, 1H), 7.64 (dd, *J* = 8.5, 2.5 Hz, 1H), 7.47 (ddd, *J* = 8.7, 2.4, 1.1 Hz, 1H). ^19^F NMR (565 MHz, CDCl_3_, ppm) *δ* −61.25 (s), −101.84 (t, *J* = 8.6 Hz). ^13^C NMR (151 MHz, CDCl_3_, ppm) *δ* 159.68 (d, *J* = 250.9 Hz), 140.08 (q, *J* = 39.9 Hz), 136.33 (d, *J* = 8.9 Hz), 135.10, 121.59 (d, *J* = 2.9 Hz), 120.21 (q, *J* = 268.1 Hz), 117.26 (d, *J* = 3.8 Hz), 110.72 (d, *J* = 21.1 Hz), 109.75 (d, *J* = 27.3 Hz). HRMS (ESI): calcd. for C_9_H_4_BrF_4_N_3_ [M + Na]^+^: 331.9417, found: 331.9411.

1-(3-Bromo-4-chlorophenyl)-4-(trifluoromethyl)-*1H*-1,2,3-triazole (**3z**).

Yellowish solid (137 mg, mp: 111–113 °C, yield: 84%). Isolated by column chromatography on silica gel (petroleum ether/ethyl acetate = 20:1, R*_f_* = 0.4). ^1^H NMR (600 MHz, CDCl_3_, ppm) *δ* 8.33 (d, *J* = 0.8 Hz, 1H), 8.07 (d, *J* = 2.5 Hz, 1H), 7.69 (dd, *J* = 8.7, 2.5 Hz, 1H), 7.65 (d, *J* = 8.6 Hz, 1H). ^19^F NMR (565 MHz, CDCl_3_, ppm) *δ* −61.20 (s). ^13^C NMR (151 MHz, CDCl_3_, ppm) *δ* 140.04 (q, *J* = 39.8 Hz), 136.34, 135.16, 131.66, 126.00, 124.07, 121.87–121.67 (d, *J* = 2.0 Hz), 120.69, 120.22 (q, *J* = 268.2 Hz). HRMS (ESI): calcd. for C_9_H_4_BrClF_3_N_3_ [M + K]_+_: 363.8861, found: 363.8876.

1-(3-Chloro-4-(trifluoromethoxy)phenyl)-4-(trifluoromethyl)-*1H*-1,2,3-triazole (**3aa**).

Yellowish solid (132 mg, mp: 72–74 °C, yield: 80%). Isolated by column chromatography on silica gel (petroleum ether/ethyl acetate = 20:1, R*_f_* = 0.4). ^1^H NMR (600 MHz, CDCl_3_, ppm) *δ* 8.35 (s, 1H), 7.96 (d, *J* = 2.6 Hz, 1H), 7.74 (dd, *J* = 8.9, 2.6 Hz, 1H), 7.54 (d, *J* = 8.8 Hz, 1H). ^19^F NMR (565 MHz, CDCl_3_, ppm) *δ* −57.83 (s), −61.29 (s). ^13^C NMR (151 MHz, CDCl_3_, ppm) *δ* 146.09, 140.19 (q, *J* = 39.9 Hz), 135.01, 129.55, 123.89, 123.61, 122.22 (d, *J* = 260.9 Hz), 121.99 (d, *J* = 268.3 Hz), 121.77 (d, *J* = 2.9 Hz), 120.30. HRMS (ESI): calcd. for C_10_H_4_ClF_6_N_3_O [M + K]^+^: 369.9579, found: 369.9582.

1-(Naphthalen-1-yl)-4-(trifluoromethyl)-*1H*-1,2,3-triazole (**3ab**) [53].

Pale red oil (110 mg, yield: 84%). Isolated by column chromatography on silica gel (petroleum ether/ethyl acetate = 10:1, R*_f_* = 0.3). ^1^H NMR (600 MHz, CDCl_3_, ppm) *δ* 8.24 (s, 1H), 8.06 (dd, *J* = 7.3, 2.2 Hz, 1H), 7.97 (d, *J* = 8.2 Hz, 1H), 7.62–7.59 (m, 1H), 7.59–7.54 (m, 3H), 7.51 (d, *J* = 8.5 Hz, 1H). ^19^F NMR (565 MHz, CDCl_3_, ppm) *δ* −60.78 (s). ^13^C NMR (151 MHz, CDCl_3_, ppm) *δ* 139.01 (q, *J* = 39.6 Hz), 134.24, 132.65, 131.36, 128.57, 128.45, 128.30, 127.49, 125.89, 125.02, 123.95, 121.76, 120.60 (q, *J* = 267.9 Hz).

1-Octyl-4-(trifluoromethyl)-*1H*-1,2,3-triazole (**4a**).

Yellowish oil (106 mg, yield: 85%). Isolated by column chromatography on silica gel (petroleum ether/ethyl acetate = 10:1, R*_f_* = 0.3). ^1^H NMR (600 MHz, CDCl_3_, ppm) *δ* 7.87 (s, 1H), 4.40 (t, *J* = 7.3 Hz, 2H), 1.96–1.89 (m, 2H), 1.35–1.18 (m, 10H), 0.85 (t, *J* = 7.1 Hz, 3H). ^19^F NMR (565 MHz, CDCl_3_, ppm) *δ* −61.01 (s). ^13^C NMR (151 MHz, CDCl_3_, ppm) *δ* 138.89 (q, *J* = 39.3 Hz), 123.04, 120.64 (q, *J* = 267.6 Hz), 50.99, 31.75, 30.23, 29.06, 28.94, 26.45, 22.65, 14.09. HRMS (ESI): calcd. for C_11_H_18_F_3_N_3_ [M + Na]^+^: 272.1345, found: 272.1339.

1-Nonyl-4-(trifluoromethyl)-*1H*-1,2,3-triazole (**4b**).

Yellowish oil (109 mg, yield: 83%). Isolated by column chromatography on silica gel (petroleum ether/ethyl acetate = 10:1, R*_f_* = 0.3). ^1^H NMR (600 MHz, CDCl_3_, ppm) *δ* 7.87 (s, 1H), 4.40 (t, *J* = 7.3 Hz, 2H), 1.92 (dt, *J* = 14.6, 7.3 Hz, 2H), 1.35–1.18 (m, 12H), 0.85 (t, *J* = 7.0 Hz, 3H). ^19^F NMR (565 MHz, CDCl_3_, ppm) *δ* −61.01 (s). ^13^C NMR (151 MHz, CDCl_3_, ppm) *δ* 138.89 (q, *J* = 39.2 Hz), 123.04, 120.64 (q, *J* = 267.5 Hz), 50.99, 31.86, 30.23, 29.36, 29.21, 28.98, 26.44, 22.70, 14.12. HRMS (ESI): calcd. for C_12_H_20_F_3_N_3_ [M + Na]^+^: 286.1502, found: 286.1497.

1-Cyclohexyl-4-(trifluoromethyl)-*1H*-1,2,3-triazole (**4c**) [53].

Yellowish solid (88 mg, mp: 34–36 °C, yield: 80%). Isolated by column chromatography on silica gel (petroleum ether/ethyl acetate = 10:1, R*_f_* = 0.3). ^1^H NMR (600 MHz, CDCl_3_, ppm) *δ* 7.84 (s, 1H), 4.51 (tt, *J* = 11.9, 3.9 Hz, 1H), 2.25 (dd, *J* = 13.8, 2.4 Hz, 2H), 1.95 (dd, *J* = 11.1, 3.1 Hz, 2H), 1.76 (dt, *J* = 16.1, 12.4 Hz, 3H), 1.48 (dt, *J* = 13.2, 3.3 Hz, 2H), 1.33 (s, 1H). ^19^F NMR (565 MHz, CDCl_3_, ppm) *δ* −60.93 (s). ^13^C NMR (151 MHz, CDCl_3_, ppm) *δ* 138.63 (q, *J* = 39.2 Hz), 120.94, 120.74 (q, *J* = 267.7 Hz), 60.89, 33.59, 25.15, 25.09.

1-((3s,5s,7s)-Adamantan-1-yl)-4-(trifluoromethyl)-*1H*-1,2,3-triazole (**4d**) [53].

White solid (125 mg, mp: 146–148 °C, yield: 92%). Isolated by column chromatography on silica gel (petroleum ether/ethyl acetate = 10:1, Rf = 0.3). ^1^H NMR (600 MHz, CDCl_3_, ppm) *δ* 7.90–7.89 (s, 1H), 2.27 (dd, *J* = 21.5, 2.9 Hz, 9H), 1.84–1.76 (m, 6H). ^19^F NMR (565 MHz, CDCl_3_, ppm) *δ* −60.81 (s). ^13^C NMR (151 MHz, CDCl_3_, ppm) *δ* 138.11 (q, *J* = 39.0 Hz), 120.87 (q, *J* = 267.8 Hz), 119.78, 60.92, 43.02, 35.83, 29.50.

1-(1-Phenylethyl)-4-(trifluoromethyl)-*1H*-1,2,3-triazole (**4e**) [53].

White solid (114 mg, mp: 57–58 °C, yield: 95%). Isolated by column chromatography on silica gel (petroleum ether/ethyl acetate = 20:1, R*_f_* = 0.3). ^1^H NMR (400 MHz, CDCl_3_, ppm) *δ* 7.69 (d, *J* = 7.4 Hz, 1H), 7.29 (q, *J* = 7.2, 6.7 Hz, 3H), 7.23–7.18 (m, 2H), 5.77 (q, *J* = 7.1 Hz, 1H), 1.92 (d, *J* = 7.1 Hz, 3H). ^19^F NMR (377 MHz, CDCl_3_, ppm) *δ* −61.05 (s). ^13^C NMR (101 MHz, CDCl_3_, ppm) *δ* 138.84, 138.80 (q, *J* = 39.1 Hz), 129.34, 129.11, 126.69, 122.21, 120.61 (q, *J* = 267.8 Hz), 61.16, 21.23. HRMS (ESI): calcd. for C_11_H_10_F_3_N_3_ [M + Na]^+^: 264.0719, found: 264.0713.

1-Phenethyl-4-(trifluoromethyl)-*1H*-1,2,3-triazole (**4f**).

White solid (91 mg, mp: 59–61 °C, yield: 80%). Isolated by column chromatography on silica gel (petroleum ether/ethyl acetate = 8:1, R*_f_* = 0.3). ^1^H NMR (600 MHz, CDCl_3_, ppm) *δ* 7.54 (s, 1H), 7.30 (dd, *J* = 7.9, 6.4 Hz, 2H), 7.28–7.25 (m, 1H), 7.08 (d, *J* = 7.0 Hz, 2H), 4.65 (t, *J* = 7.2 Hz, 2H), 3.23 (t, *J* = 7.2 Hz, 2H). ^19^F NMR (565 MHz, CDCl_3_, ppm) *δ* −61.01 (s). ^13^C NMR (151 MHz, CDCl_3_, ppm) *δ* 138.63 (q, *J* = 39.3 Hz), 136.41, 129.10, 128.71, 127.53, 123.55, 120.53 (q, *J* = 267.6 Hz), 52.27, 36.63. HRMS (ESI): calcd. for C_11_H_10_F_3_N_3_ [M + Na]^+^: 264.0719, found: 264.0715.

4-((4-(Trifluoromethyl)-*1H*-1,2,3-triazol-1-yl)methyl)pyridine (**4g**).

White solid (60 mg, mp: 104–105 °C, yield: 53%). Isolated by column chromatography on silica gel (petroleum ether/ethyl acetate = 4:1, R*_f_* = 0.3). ^1^H NMR (600 MHz, CDCl_3_, ppm) *δ* 8.63 (s, 2H), 7.90 (s, 1H), 7.13 (d, *J* = 5.4 Hz, 2H), 5.61 (s, 2H). ^19^F NMR (565 MHz, CDCl3, ppm) *δ* −61.07 (s). ^13^C NMR (151 MHz, CDCl_3_, ppm) *δ* 150.89, 142.43, 139.70 (q, *J* = 39.6 Hz), 123.62, 122.33, 120.30 (q, *J* = 267.9 Hz), 53.24. HRMS (ESI): calcd. for C_9_H_7_F_3_N_4_ [M + H]^+^: 229.0696, found: 229.0692.

1-(Thiophen-3-ylmethyl)-4-(trifluoromethyl)-*1H*-1,2,3-triazole (**4h**).

White solid (100 mg, mp: 82–83 °C, yield: 86%). Isolated by column chromatography on silica gel (petroleum ether/ethyl acetate = 10:1, R*_f_* = 0.3). ^1^H NMR (600 MHz, CDCl_3_, ppm) *δ* 7.76 (s, 1H), 7.40 (dd, *J* = 5.0, 3.0 Hz, 1H), 7.34 (d, *J* = 1.9 Hz, 1H), 7.03 (dd, *J* = 5.0, 1.3 Hz, 1H), 5.61 (s, 2H). ^19^F NMR (565 MHz, CDCl_3_, ppm) *δ* −60.99 (s). ^13^C NMR (151 MHz, CDCl_3_, ppm) *δ* 139.26 (q, *J* = 39.3 Hz), 133.86, 128.09, 127.08, 125.39, 122.98, 120.49 (q, *J* = 267.8 Hz), 49.55. HRMS (ESI): calcd. for C_8_H_6_F_3_N_3_S [M + Na]^+^: 256.0127, found: 256.0122.

1-(Benzo[b]thiophen-2-ylmethyl)-4-(trifluoromethyl)-*1H*-1,2,3-triazole (**4i**).

White solid (122 mg, mp: 127–129 °C, yield: 86%). Isolated by column chromatography on silica gel (petroleum ether/ethyl acetate = 10:1, R*_f_* = 0.3). ^1^H NMR (600 MHz, CDCl_3_, ppm) *δ* 7.91 (s, 1H), 7.81 (dd, *J* = 8.4, 1.8 Hz, 1H), 7.78 (dd, *J* = 6.5, 2.5 Hz, 1H), 7.42–7.36 (m, 4H), 5.85 (s, 2H). ^19^F NMR (565 MHz, CDCl_3_, ppm) *δ* −61.00 (s). ^13^C NMR (151 MHz, CDCl_3_, ppm) *δ* 140.47, 139.45 (q, *J* = 39.6 Hz), 139.10, 135.38, 125.72, 125.67, 125.17, 124.28, 123.16, 122.65, 120.42 (q, *J* = 268.2 Hz), 49.86. HRMS (ESI): calcd. for C_12_H_8_F_3_N_3_S [M + Na]^+^: 306.0283, found: 306.0280.

2-(2-(4-(trifluoromethyl)-*1H*-1,2,3-triazol-1-yl)ethyl)isoindoline-1,3-dione (**4j**).

White solid (84 mg, mp: 165–167 °C, yield: 54%). Isolated by column chromatography on silica gel (petroleum ether/ethyl acetate = 4:1, R*_f_* = 0.3). ^1^H NMR (600 MHz, CDCl_3_, ppm) *δ* 7.98 (d, *J* = 0.9 Hz, 1H), 7.81 (dd, *J* = 5.4, 3.0 Hz, 2H), 7.72 (dd, *J* = 5.5, 3.0 Hz, 2H), 4.76 (dd, *J* = 12.0, 6.0 Hz, 2H), 4.19 (dd, *J* = 12.0, 6.0 Hz, 2H). ^19^F NMR (565 MHz, CDCl_3_, ppm) *δ* −61.01 (s). ^13^C NMR (151 MHz, CDCl_3_, ppm) *δ* 167.73, 139.31 (q, *J* = 39.5 Hz), 134.58, 131.65, 123.79, 123.73 (d, *J* = 2.6 Hz), 120.41 (q, *J* = 267.8 Hz), 48.63, 37.57. HRMS (ESI): calcd. for C_13_H_9_F_3_N_4_O_2_ [M + Na]^+^: 333.0570, found: 333.0565.

1-(2-Phenoxyethyl)-4-(trifluoromethyl)-*1H*-1,2,3-triazole (**4k**).

White solid (102 mg, mp: 69–71 °C, yield: 80%). Isolated by column chromatography on silica gel (petroleum ether/ethyl acetate = 20:1, R*_f_* = 0.3). ^1^H NMR (400 MHz, CDCl_3_, ppm) *δ* 8.09 (s, 1H), 7.31 (dd, *J* = 8.7, 7.4 Hz, 2H), 7.01 (t, *J* = 7.4 Hz, 1H), 6.88 (dd, *J* = 8.7, 0.9 Hz, 2H), 4.83 (t, *J* = 4.8 Hz, 2H), 4.38 (t, *J* = 4.8 Hz, 2H). ^19^F NMR (565 MHz, CDCl_3_, ppm) *δ* −60.97 (s). ^13^C NMR (151 MHz, CDCl_3_, ppm) *δ* 157.61, *δ* 139.21 (q, *J* = 39.7 Hz), 129.92, 124.44, 122.18, 120.54 (q, *J* = 267.7 Hz), 114.61, 65.98, 50.42. HRMS (ESI): calcd. for C_11_H_10_F_3_N_3_O [M + Na]^+^: 280.0668, found: 280.0665.

1-(2,5,8,11-Tetraoxatridecan-13-yl)-4-(trifluoromethyl)-*1H*-1,2,3-triazole (**4l**).

Yellowish oil (120 mg, yield: 73%). Isolated by column chromatography on silica gel (petroleum ether/ethyl acetate = 1:1.2, R*_f_* = 0.3). ^1^H NMR (600 MHz, CDCl_3_, ppm) *δ* 8.20 (s, 1H), 4.61 (t, *J* = 4.8 Hz, 2H), 3.87 (t, *J* = 4.8 Hz, 2H), 3.63–3.60 (m, 10H), 3.54–3.50 (m, 2H), 3.34 (s, 3H). ^19^F NMR (565 MHz, CDCl_3_, ppm) *δ* −60.86 (s). ^13^C NMR (151 MHz, CDCl_3_, ppm) *δ* 138.80 (q, *J* = 39.3 Hz), 124.85, 120.72 (q, *J* = 267.6 Hz) 71.96, 70.63, 70.60, 70.55, 70.50, 69.03, 59.06, 50.75. HRMS (ESI): calcd. for C_12_H_20_F_3_N_3_O_4_ [M + Na]^+^: 350.1298, found: 350.1292.

Ethyl 4-(4-(trifluoromethyl)-*1H*-1,2,3-triazol-1-yl)butanoate (**4m**).

Yellowish oil (80 mg, yield: 64%). Isolated by column chromatography on silica gel (petroleum ether/ethyl acetate = 4:1, R*_f_* = 0.3). 1H NMR (600 MHz, CDCl_3_, ppm) *δ* 7.90 (s, 1H), 4.51 (t, *J* = 7.0 Hz, 2H), 4.13 (q, *J* = 7.1 Hz, 2H), 2.36 (t, *J* = 7.0 Hz, 2H), 2.26 (t, *J* = 7.0 Hz, 2H), 1.25 (t, *J* = 7.1 Hz, 3H). ^19^F NMR (565 MHz, CDCl_3_, ppm) *δ* −61.01 (s). ^13^C NMR (151 MHz, CDCl_3_, ppm) *δ* 172.23, 139.07 (q, *J* = 39.3 Hz), 123.44, 120.54 (q, *J* = 267.6 Hz), 61.01, 49.87, 30.60, 25.41, 14.25. HRMS (ESI): calcd. for C_9_H_12_F_3_N_3_O_2_ [M + Na]^+^: 274.0774, found: 274.0770.

1-Cinnamyl-4-(trifluoromethyl)-*1H*-1,2,3-triazole (**4n**).

White solid (102 mg, mp: 66–67 °C, yield: 81%). Isolated by column chromatography on silica gel (petroleum ether/ethyl acetate = 10:1, R*_f_* = 0.3). ^1^H NMR (600 MHz, CDCl_3_, ppm) *δ* 7.88 (s, 1H), 7.35 (d, *J* = 7.1 Hz, 2H), 7.30 (t, *J* = 7.3 Hz, 2H), 7.27 (d, *J* = 7.1 Hz, 1H), 6.69 (d, *J* = 15.8 Hz, 1H), 6.29 (dt, *J* = 15.7, 6.9 Hz, 1H), 5.15 (dd, *J* = 6.8, 1.4 Hz, 2H). ^19^F NMR (565 MHz, CDCl_3_, ppm) *δ* −60.97 (s). ^13^C NMR (151 MHz, CDCl_3_, ppm) *δ* 139.29 (q, *J* = 39.3 Hz), 136.88, 135.17, 129.05, 128.95, 126.94, 122.96, 120.59, 120.56 (q, *J* = 267.8 Hz), 52.94. HRMS (ESI): calcd. for C_12_H_10_F_3_N_3_ [M + Na]^+^: 276.0719, found: 276.0714.

## 4. Conclusions

In the study chronicled above, we demonstrated an efficient protocol to produce 1-substituted-4-trifluoromethyl-1,2,3-triazoles from BTP with azides. In this reaction, copper salt and ligands were necessary to catalyze the click process, and CuI (10 mol%)/Phen (10 mol%) gave the best yield with 2.0 eq. DBU as the base in CH_3_CN at 65 °C. With the mild reaction conditions, both aryl azide and alkyl azides bearing a range of electronically and sterically different groups participate in this process to give good to excellent yields. Moreover, this process can be conducted readily and on modestly large scales. Further studies are in progress to develop the chemistry of 4-trifluoromethyl-1,2,3-triazoles generated using this click reaction platform.

## Figures and Tables

**Table 1:** Optimization of the Reaction Conditions ^1^.

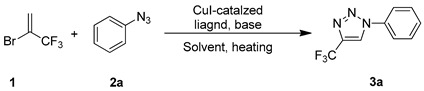						
Entry	CuI	Ligand ^2^	Solvent	Temp. (°C)	Time (h)	Yield ^3^
1	none	none	DMF	100	16	n.d.
2	10 mol%	none	DMF	80	16	47
3	10 mol%	Phen	CH_3_CN	35	4	37
4	10 mol%	Phen	CH_3_CN	50	4	76
5	10 mol%	Phen	CH_3_CN	50	10	87
6	10 mol%	Phen	CH_3_CN	65	4	95
7	10 mol%	Phen	CH_3_CN	75	4	92
8	10 mol%	Phen	CH_3_CN	65	3	93
9	10 mol%	Phen	CH_3_CN	65	2	79
10	10 mol%	Phen	DMF	65	4	94
11	10 mol%	Phen	DMSO	65	4	51
12 ^4^	10 mol%	L1	CH_3_CN	65	4	93
13 ^5^	10 mol%	L2	CH_3_CN	65	4	93
14	10 mol%	Phen	CH_3_CN	65	4	69
15	10 mol%	Phen	CH_3_CN	65	4	95
16 ^6^	5 mol%	Phen	CH_3_CN	65	4	87

^1^ Standard reaction conditions: **1** (1.25 mmol, 2.5 equiv), **2a** (0.5 mmol, 1.0 equiv.), CuI (0.05 mmol, 10 mol%), ligand (0.05 mmol, 10 mol%), solvent (4.0 mL), DBU (1.0 mmol, 2.0 equiv.), 65 °C, 4 h under air atmosphere. ^2^ Phen = 1,10-Phenanthroline. ^3^ Isolated yield after column chromatography. ^4^ L1 = 4,7-Dimethoxy-1,10-phenanthroline. ^5^ L2 = 3,4,7,8-Tetramethyl-1,10-phenanthroline. ^6^ 5 mmol% of CuI was loaded.

**Table 2:** Substrate Scope of 1-Aryl-4-CF_3_-1,2,3-Triazoles ^1,2^.

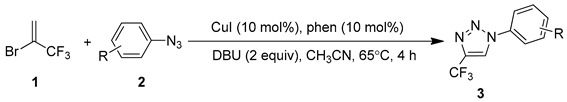
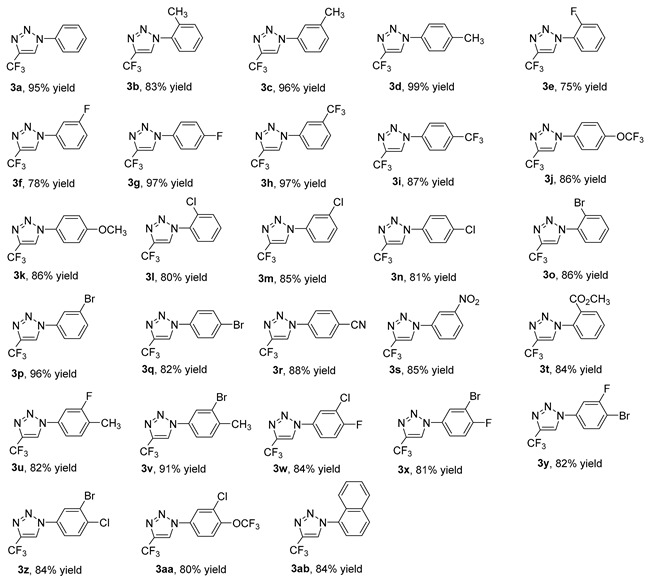

^1^ Reaction conditions: **1** (1.25 mmol, 2.5 equiv.), **2** (0.5 mmol, 1.0 equiv.), CuI (0.05 mmol, 10 mol%), Phen (0.05 mmol, 10 mol%), CH_3_CN (4.0 mL), DBU (1.0 mmol, 2.0 equiv.), 65 °C, 4 h under air atmosphere. ^2^ Isolated yields.

**Table 3:** Substrate Scope of 1-Alkyl-4-CF_3_-1,2,3-Triazoles ^1,2^.

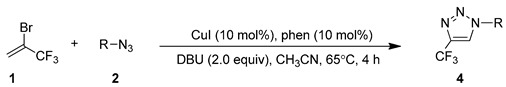
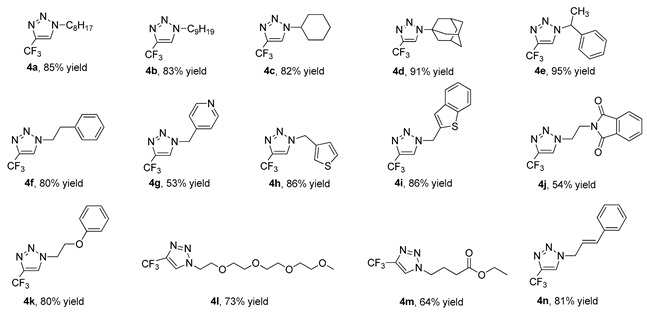

^1^ Reaction conditions: **1** (1.25 mmol, 2.5 equiv.), **2** (0.5 mmol, 1.0 equiv.), CuI (0.05 mmol, 10 mol%), Phen (0.05 mmol, 10 mol%), CH_3_CN (4.0 mL), DBU (1.0 mmol, 2.0 equiv.), 65 °C, 4 h under air atmosphere. ^2^ Isolated yields.

## Data Availability

^1^H- NMR and ^13^C- and ^19^F-NMR Data are available in section “MDPI Research Data Policies” at https://www.mdpi.com/ethics.

## Supplementary Materials

The following supporting information can be downloaded at: https://www.mdpi.com/article/10.3390/molecules29061191/s1, Table S1: Optimization of the Reaction Conditions; Copies of ^1^H NMR, ^19^F NMR and ^13^C NMR spectra [85,86,87,88,89,90,91,92,93,94].
