# Supplementary material for: Copper-Catalyzed Synthesis of 4-CF3-1,2,3-Triazoles: An Efficient and Facile Approach via Click Reaction
Source: Molecules. 2024 Mar 7;29(6):1191. doi: 10.3390/molecules29061191 (PMC10975795; doi:10.3390/molecules29061191)
Supplement: Supplementary file 1 [file molecules-29-01191-s001.zip › molecules-2876876-supplementary.pdf]

Copper-Catalyzed Synthesis of 4-CF3-1,2,3-Triazoles: An Ef-fi-cient and Facile Approach via Click Reaction

Tinghong Tang,[a] Cuiting Chen,[a] Xin Fu,[a] Huilan Xu,[a] Luyong Wu,[a,*] and Wenhao Chen[a,*]

[a] Key Laboratory of Tropical Medicinal Resource Chemistry of Ministry of Education, Hainan Normal University, Haikou 571158, P. R. China

E-mail: wuluyong@hainnu.edu.cn; 070103@hainnu.edu.cn

Table of Contents

General information………………………………………………………….. 2

Synthesis of starting azides……………………………………………………2

General procedure of synthesis of 4-trifluoromethyl-1,2,3-triazoles ………………3

Procedure for gram scale reaction…………………..….. 3

The optimization reaction conditions…..…...4

Copies of ^{1}H NMR, ^{19}F NMR and ^{13}C NMR spectra………………………………8

General information

Melting points were measured with a Beijing-Taike X-4 apparatus without corrected. ^{1}H NMR, ^{19}F NMR and ^{13}C NMR spectra were recorded using Bruker Advance 400MHz or JEOL RESONANCE ECZ600R spectrometer. Chemical shifts were reported in ppm from the solvent resonance as the internal standard ($CDCl_3$: δ_H = 7.26 ppm, δ_C = 77.16 ppm). Coupling constants (J) are reported in Hertz (Hz). The following abbreviations were used to describe peak splitting patterns when appropriate: s = singlet, d = doublet, dd = double doublet, ddd = double doublet of doublets, t = triplet, dt = double triplet, q = quatriplet, m = multiplet. HRMS were obtained on LCMS-IT-TOF. Reagents were received from commercial sources. Solvents were freshly dried and degassed according to the published procedures prior to use. Isolated by column chromatography on silica gel (200~300 mesh).

Synthesis of starting azides

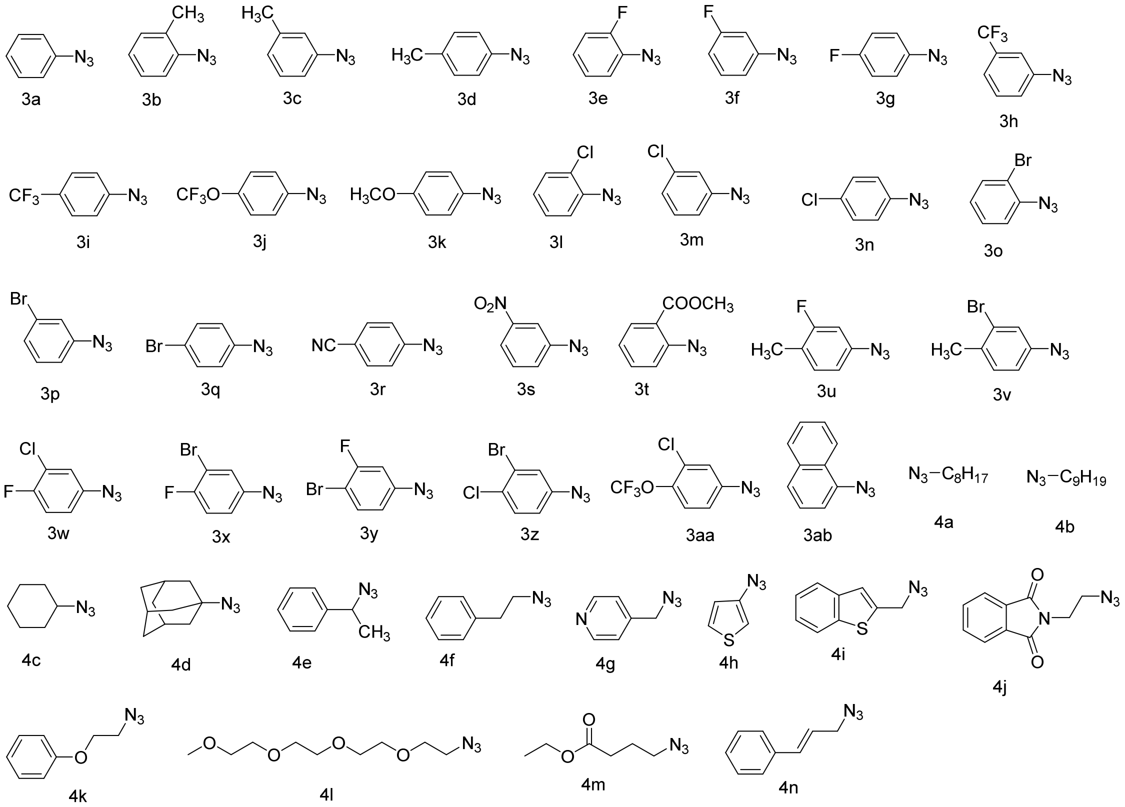

All azides used in this work were synthesized according to the reported methods.

Aromatic azides **3a-3ab** were synthesized from aromatic amines via successive diazotization and azidation.[1]; **4a-4c, 4e-4g, 4j, 4k, 4m, 4n** were prepared from corresponding bromoalkanes via nucleophilic substitution.[2]; azides **4d, 4h, 4i, 4l** were synthesized from corresponding alcohols via successive tosylation and azidation.[3]

General procedure of synthesis of 4-trifluoromethyl-1,2,3-triazoles.

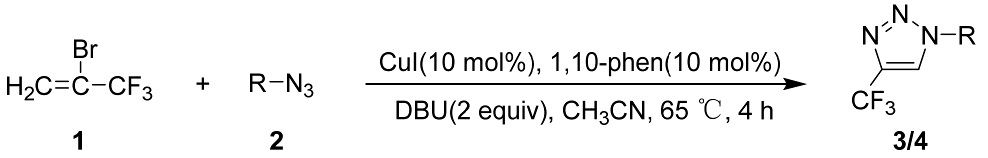

Organic azide (**2**) (0.50 mmol, 1.0 equiv), 2-bromo-3,3, 3-trifluoropropylene (**1**) (219 mg, 1.25 mmol, 2.5 equiv) were prepared in 10 mL vials with stirrer drying. acetonitrile (4.0 mL) solution was added with CuI(9.5 mg, 0.05 mmol, 10 mol%), 1,10- phenanthroline (9.0 mg, 0.05 mmol, 10 mol%) and DBU (152 mg, 1.0 mmol, 2.0 equiv), sealed with a lid, and the mixture was stirred at 65° C for 4 hours. After the reaction, the mixture was diluted with ethyl acetate (30 mL) and washed with water (10 mL×4) and salt water. The organic phase is dried with Na_2SO_4(anhydrous). After the solvent was removed by rotary evaporation, the residue was purified by silica gel column chromatography with petroleum ether/ethyl acetate (*V*: *V*= 20:1 - 1:1.2) as eluent to obtain 1-substituted -4-trifluoromethyl -1,2, 3-triazole compounds.

Procedure for gram scale reaction for synthesis of 1-phenyl-4-(trifluoromethyl)-1*H*-1,2,3-triazole (3a)

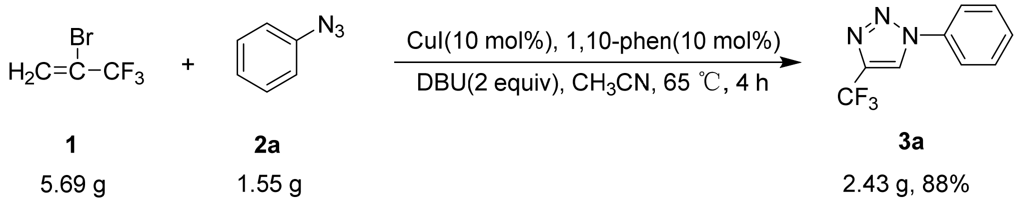

Organic azobenzene (**2a**) (1.55 g, 13.0 mmol, 1.0 equiv), 2-bromo-3,3, 3-trifluoropropylene (**1**) (5.69 g, 32.5 mmol, 2.5 equiv) were prepared in 100 mL reaction bottle with agitator drying. The acetonitrile (40 mL) solution of was added to CuI(247 mg, 1.3 mmol, 10 mol%), 1,10- phenanthroline (234 mg, 1.3 mmol, 10 mol%) and DBU(3.95

g, 26 mmol, 2.0 equiv), sealed with a lid, and the mixture was stirred at 65 ° C for 4 hours. After the reaction, the mixture was diluted with ethyl acetate (150 mL) and washed with water (50 mL × 4) and salt water. The organic phase is dried with Na_2SO_4(anhydrous). After the solvent was removed by rotary evaporation, the residue was purified by silica gel column chromatography with petroleum ether/ethyl acetate (*V*: *V*= 20:1) as eluent to obtain 1-phenyl-4-trifluoromethyl-1,2, 3-triazole compounds.

Table 1 Optimization of the Reaction Conditions[a].

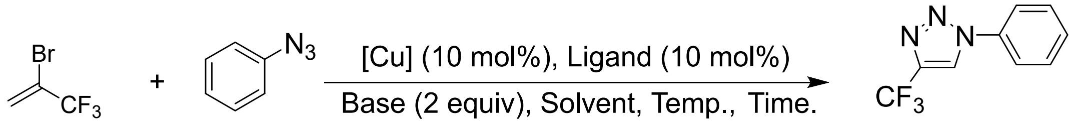

1 2a 3a

Entry	Catal.	Ligand	Base	Solvent	Temp .(℃)	Time (h)	Yield(%)[b]
1	-	-	DBU (3.5 eq.)	DMF	100	16	n.d.
2	-	-	Cs_2CO_3 (3.5 eq.)	DMF	100	16	n.d.
3	-	-	K_2CO_3 (3.5 eq.)	DMF	100	16	n.d.
4	-	-	KO^t Bu (3.5 eq.)	DMF	100	16	n.d.
5	-	-	NaO^t Bu (3.5 eq.)	DMF	100	16	n.d.
6	-	-	LiO^t Bu (3.5 eq.)	DMF	100	16	n.d.
7	CuI	-	DBU (3.5 eq.)	DMF	80	16	47
8	CuBr	-	DBU (3.5 eq.)	DMF	80	16	33
9	CuCl	-	DBU (3.5 eq.)	DMF	80	16	19
10	CuCl	-	DBU (3.5 eq.)	DMF	80	16	19
11	$Cu(OAc)_2$	-	DBU (3.5 eq.)	DMF	80	16	33
12	$Cu(OAc)_2$	-	DBU (3.5 eq.)	DMF	80	16	33
13	CuI (10 mol%)	Phen (10 mol%)	DBU (3.5 eq.)	CH_3CN	35	4	37
14	CuCl (10 mol%)	Phen (10 mol%)	DBU (3.5 eq.)	CH_3CN	35	4	29

15	CuBr (10 mol%)	Phen (10 mol%)	DBU (3.5 eq.)	CH_3CN	35	4	31
16	$CuBr_2$ (10 mol%)	Phen (10 mol%)	DBU (3.5 eq.)	CH_3CN	35	4	22
17	CuSCN (10 mol%)	Phen (10 mol%)	DBU (3.5 eq.)	CH_3CN	35	4	30
18	$CuSO_4 \cdot 5H_2O$ (10 mol%)	Phen (10 mol%)	DBU (3.5 eq.)	CH_3CN	35	4	20
19	CuI (10 mol%)	Phen (10 mol%)	DBU (3.5 eq.)	CH_3CN	50	4	76
20	CuI (10 mol%)	Phen (10 mol%)	DBU (3.5 eq.)	CH_3CN	65	4	95
21	CuI (10 mol%)	Phen (10 mol%)	DBU (3.5 eq.)	DMF	65	4	94
22	CuI (10 mol%)	Phen (10 mol%)	DBU (3.5 eq.)	DMSO	65	4	51
23	CuI (10 mol%)	Phen (10 mol%)	DBU (3.5 eq.)	NMP	65	4	56
24	CuI (10 mol%)	Phen (10 mol%)	DBU (3.5 eq.)	THF	65	4	18
25	CuI (10 mol%)	Phen (10 mol%)	DBU (3.5 eq.)	Toluene	65	4	n.d.
26	CuI (10 mol%)	TMEDA (10 mol%)	DBU (3.5 eq.)	CH_3CN	65	4	75
27	CuI (10 mol%)	Pyr. (10 mol%)	DBU (3.5 eq.)	CH_3CN	65	4	57
28	CuI (10 mol%)	PPh_3 (10 mol%)	DBU (3.5 eq.)	CH_3CN	65	4	15
29	CuI (10 mol%)	L1 (10 mol%)	DBU (3.5 eq.)	CH_3CN	65	4	15
30	CuI (10 mol%)	Phen (10 mol%)	DBU (3.5 eq.)	CH_3CN	50	10	87
31	CuI (10 mol%)	Phen (10 mol%)	DBU (3.5 eq.)	CH_3CN	65	3	93
32	CuI (10 mol%)	Phen (10 mol%)	DBU(3.5 eq.)	CH_3CN	65	2	79
33	CuI (10 mol%)	Phen (10 mol%)	DBU(3.5 eq.)	CH_3CN	75	4	92
34	CuCl(10 mol%)	Phen (10 mol%)	DBU (3.5 eq.)	CH_3CN	65	4	91
35	$CuCl_2 \cdot 2H_2O$ (10 mol%)	Phen (10 mol%)	DBU (3.5 eq.)	CH_3CN	65	4	80

36	CuBr (10 mol%)	Phen (10 mol%)	DBU (3.5 eq.)	CH_3CN	65	4	88
37	$CuBr_2$ (10 mol%)	Phen (10 mol%)	DBU (3.5 eq.)	CH_3CN	65	4	76
38	$Cu(OAc)_2$ (10 mol%)	Phen (10 mol%)	DBU (3.5 eq.)	CH_3CN	65	4	91
39	CuSCN (10 mol%)	Phen (10 mol%)	DBU (3.5 eq.)	CH_3CN	65	4	90
40	CuI (10 mol%)	L2 (10 mol%)	DBU (3.5 eq.)	CH_3CN	65	4	93
41	CuI (10 mol%)	L3 (10 mol%)	DBU (3.5 eq.)	CH_3CN	65	4	93
42	CuI (10 mol%)	—	DBU (3.5 eq.)	CH_3CN	65	4	69
43	CuI (10 mol%)	Phen (10 mol%)	DBU(2.0 eq.)	CH_3CN	65	4	95
44	CuI (10 mol%)	Phen (10 mol%)	Cs_2CO_3 (2.0 eq.)	CH_3CN	65	4	0
45	CuI (10 mol%)	Phen (10 mol%)	K_2CO_3 (2.0 eq.)	CH_3CN	65	4	0
46	CuI (10 mol%)	Phen (10 mol%)	K_3PO_4 (2.0 eq.)	CH_3CN	65	4	0
47	CuI (10 mol%)	Phen (10 mol%)	KOH (2.0 eq.)	CH_3CN	65	4	50
48	CuI (10 mol%)	Phen (10 mol%)	LiO^tBu (2.0 eq.)	CH_3CN	65	4	0
49	CuI (10 mol%)	Phen (10 mol%)	NaO^tBu (2.0 eq.)	CH_3CN	65	4	38
50	CuI (10 mol%)	Phen (10 mol%)	KO^tBu (2.0 eq.)	CH_3CN	65	4	51
51	CuI (10 mol%)	Phen (10 mol%)	TBD (2.0 eq.)	CH_3CN	65	4	56
52	CuI (10 mol%)	Phen (10 mol%)	Et_3N (2.0 eq.)	CH_3CN	65	4	0
53	CuI (5mol%)	Phen (10 mol%)	DBU (2.0 eq.)	CH_3CN	65	4	90

[a] Reaction conditions: **1** (1.25 mmol, 2.5 equiv), **2 a** (0.5 mmol, 1.0 equiv), Ligand (10 mol%), Solvent (4.0 mL); L1 = 2,2'-Bi-4-picoline; L2 = 4,7-diCH_3O- phen; L3 = 3,4,7,8-tetra-CH_3-phen.

[b] Isolated yields.

References：

[1] a) Benati, L.; Bencivenni, G.; Leardini, R.; Minozzi, M.; Nanni, D.; Scialpi, R.; Spagnolo, P.; Zanardi, G. Radical Reduction of Aromatic Azides to Amines with Triethylsilane. *J. Org. Chem.* **2006**, *71*, 5822–5825; b) Zeng, L.; Li, J.; Cui, S. Rhodium-Catalyzed Atroposelective Click Cycloaddition of Azides and Alkynes. *Angew. Chem. Int. Ed*. **2022**, *61*, e202205037.

[2] a) Alvarez, S.G.; Alvarez, M.T. A Practical Procedure for the Synthesis of Alkyl Azides at Ambient Temperature in Dimethyl Sulfoxide in High Purity and Yield. *Synthesis* **1997**, *1997*, 413–414; b) Barbosa, F.C.; Oliveira RN, D. Synthesis of a new class of triazole-linked benzoheterocycles via 1,3-dipolar cycloaddition. *J. Braz. Chem. Soc*. **2011**, *22*, 592–597; c) Sallustrau, A.; Bregant, S.; Chollet, C.; Audisio, D.; Taran, F. Scalable and practical synthesis of clickable Cu-chelating azides. *Chem. Comm.* **2017**, *53*, 7890–7893; d) Piccinno, M.; Aragay, G.; Mihan, F.Y.; Ballester, P.; Dalla Cort, A. Unexpected Emission Properties of a 1,8-Naphthalimide Unit Covalently Appended to a Zn–Salophen. *Eur. J. Inorg. Chem*. **2015**, *2015*, 2664–2670; e) Wu, X.; Wu, W.; Cui, X.; Zhao, J.; Wu, M. Preparation of Bodipy–ferrocene dyads and modulation of the singlet/triplet excited state of bodipy via electron transfer and triplet energy transfer. *J. Mater. Chem. C.* **2016**, *4*, 2843–2853.

[3] a) Añón, E.; Costero, A.M.; Gaviña, P.; Parra, M.; El Haskouri, J.; Amorós, P.; Martínez-Máñez, R.; Sancenón, F. Not always what closes best opens better: Mesoporous nanoparticles capped with organic gates. *Sci. Technol. Adv. Mat*. **2019**, *20*, 699–709; b) Siles, R.; Kawasaki, Y.; Ross, P.; Freire, E. Synthesis and biochemical evaluation of triazole/tetrazole-containing sulfonamides against thrombin and related serine proteases. *Bioorg. Med. Chem. Lett*. **2011**, *21*, 5305–5309; c) Peroković, V. P., Car, Ž., Draženović, J., Stojković, R., Milković, L., Antica, M.; Škalamera, Đ.; Tomić, S.; Ribić, R. Design, Synthesis, and Biological Evaluation of Desmuramyl Dipeptides Modified by Adamantyl-1,2,3-triazole. *Molecules* **2021**, *26*, 6352.

Copies of ^{1}H NMR, ^{19}F NMR and ^{13}C NMR spectra

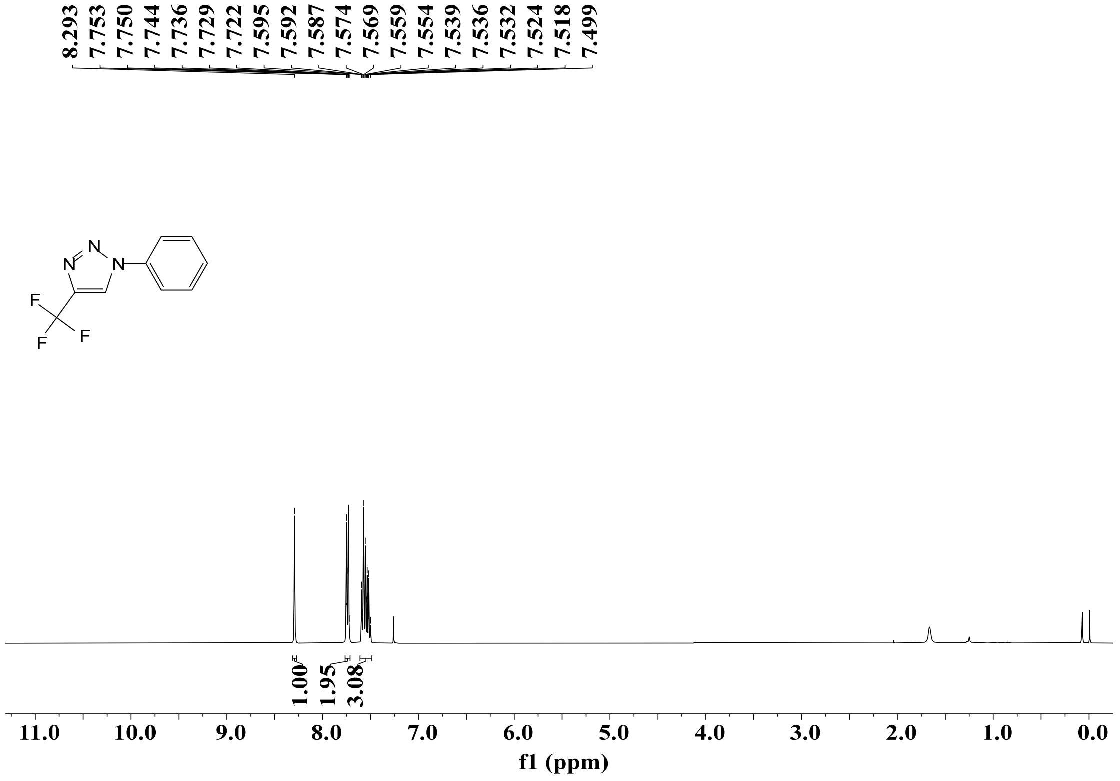

3a-^{1}H NMR

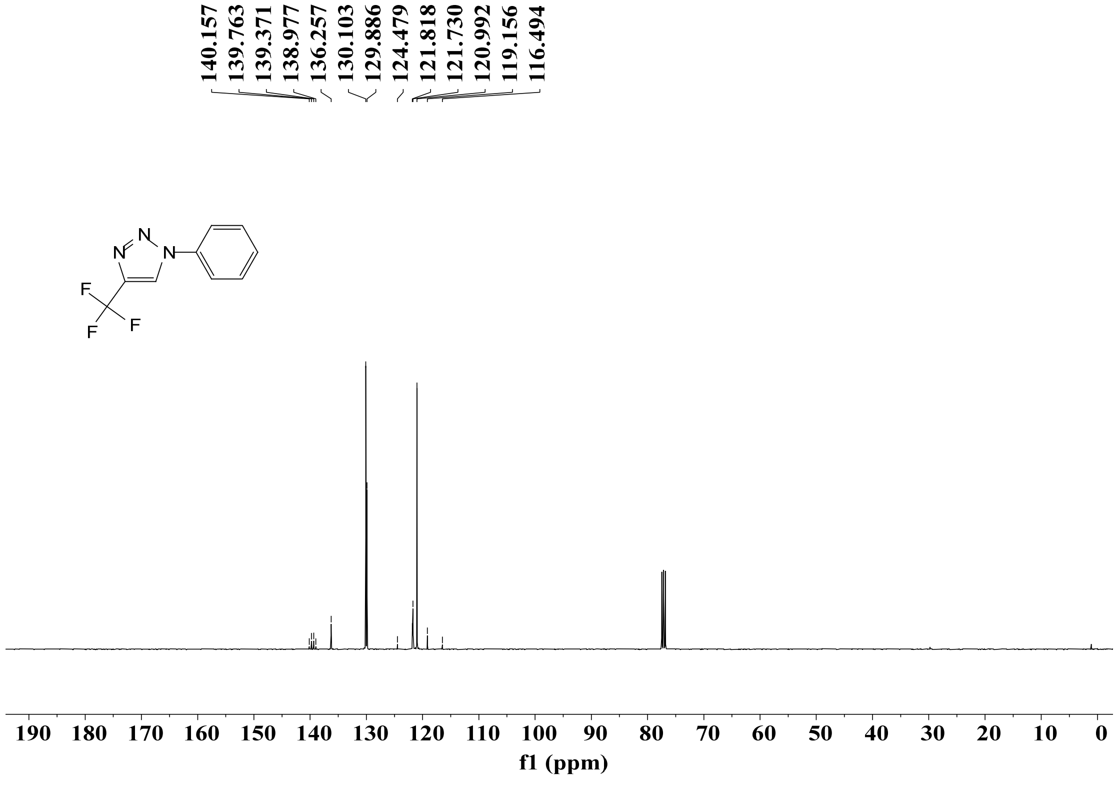

3a-^{13}C NMR

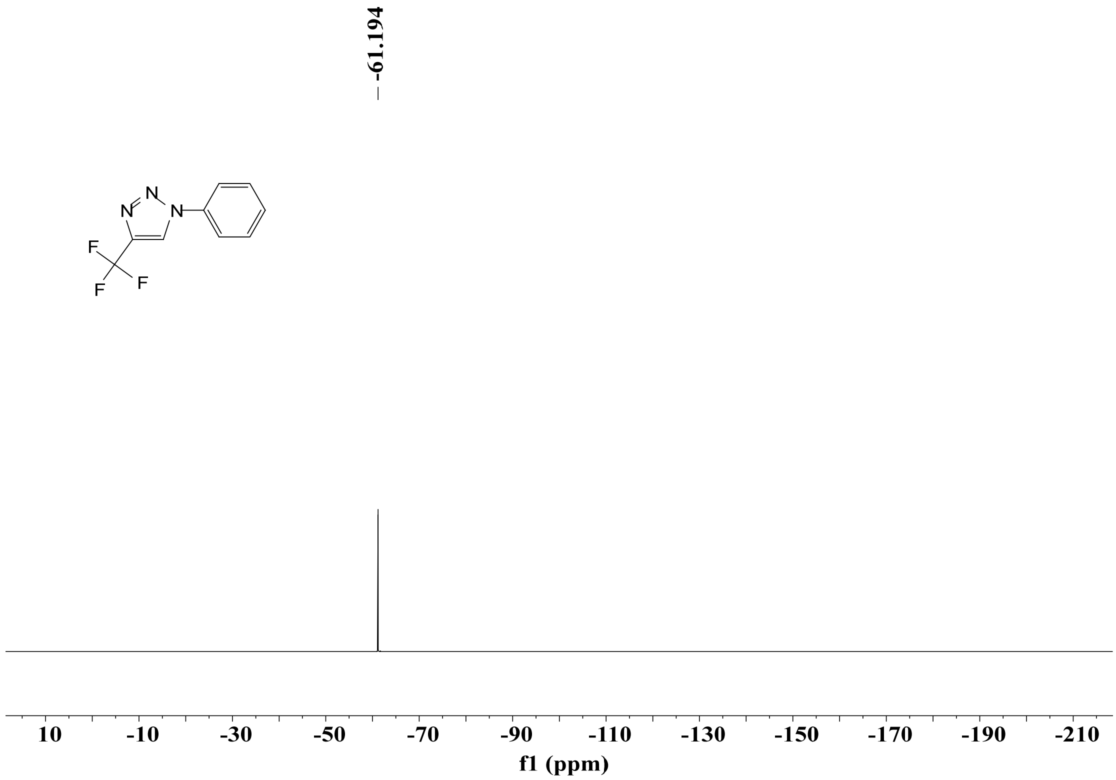

3a-^{19}F NMR

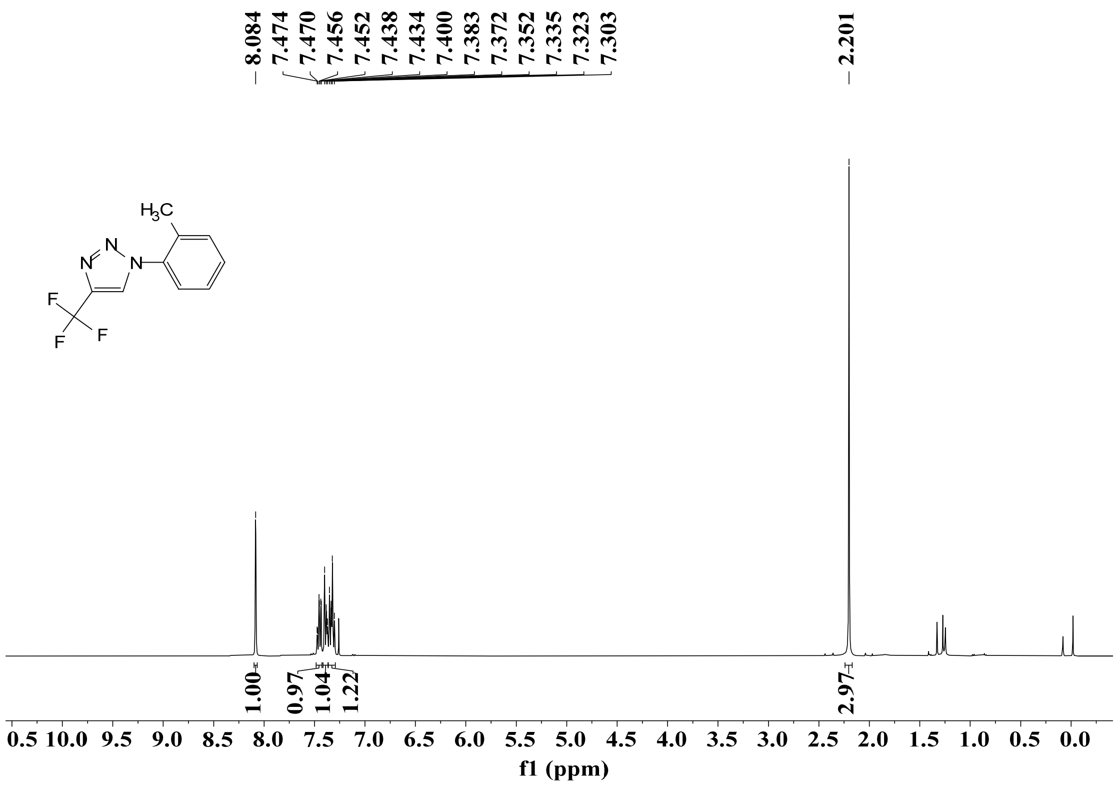

3b-¹H NMR

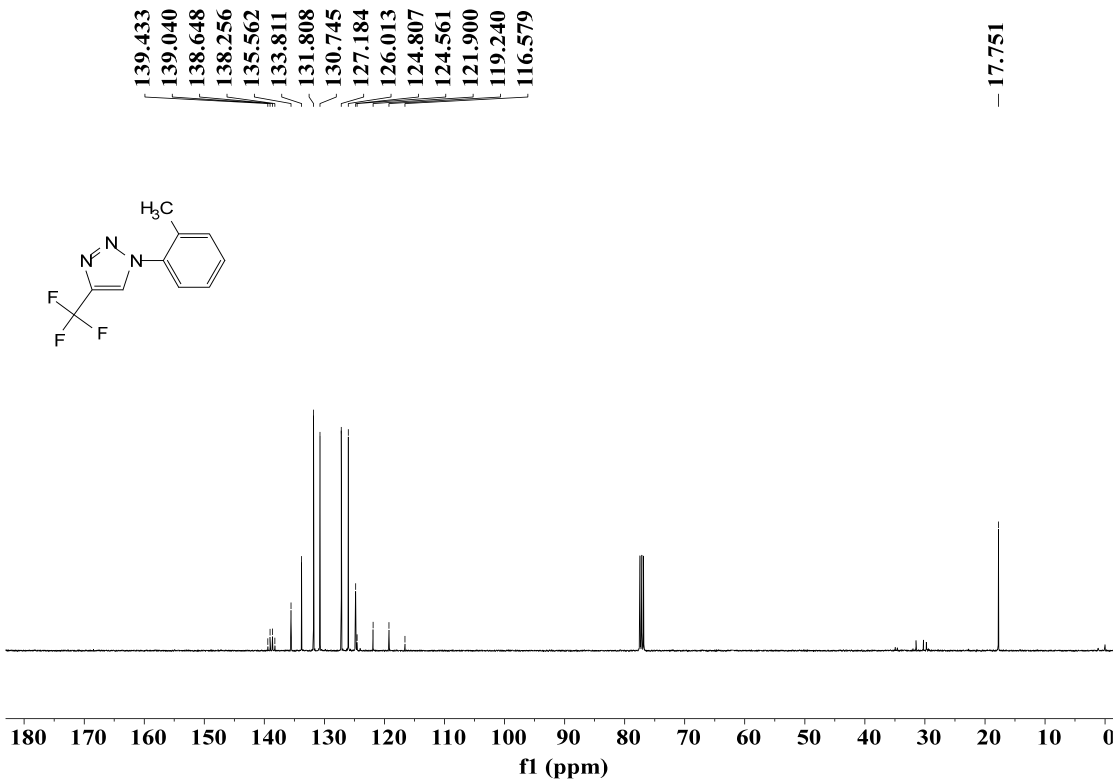

3b-¹³C NMR

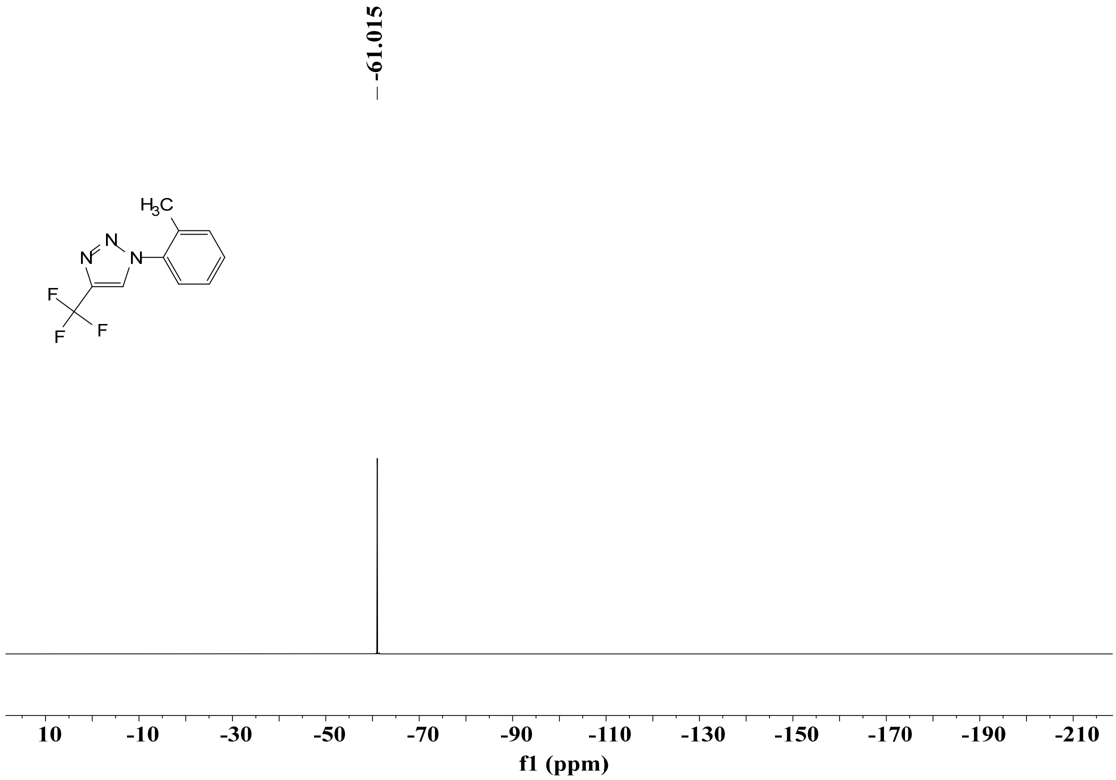
-61.015
H3C
N N N
F F F
10 -10 -30 -50 -70 -90 -110 -130 -150 -170 -190 -210
f1 (ppm)

3b-^{19}F NMR

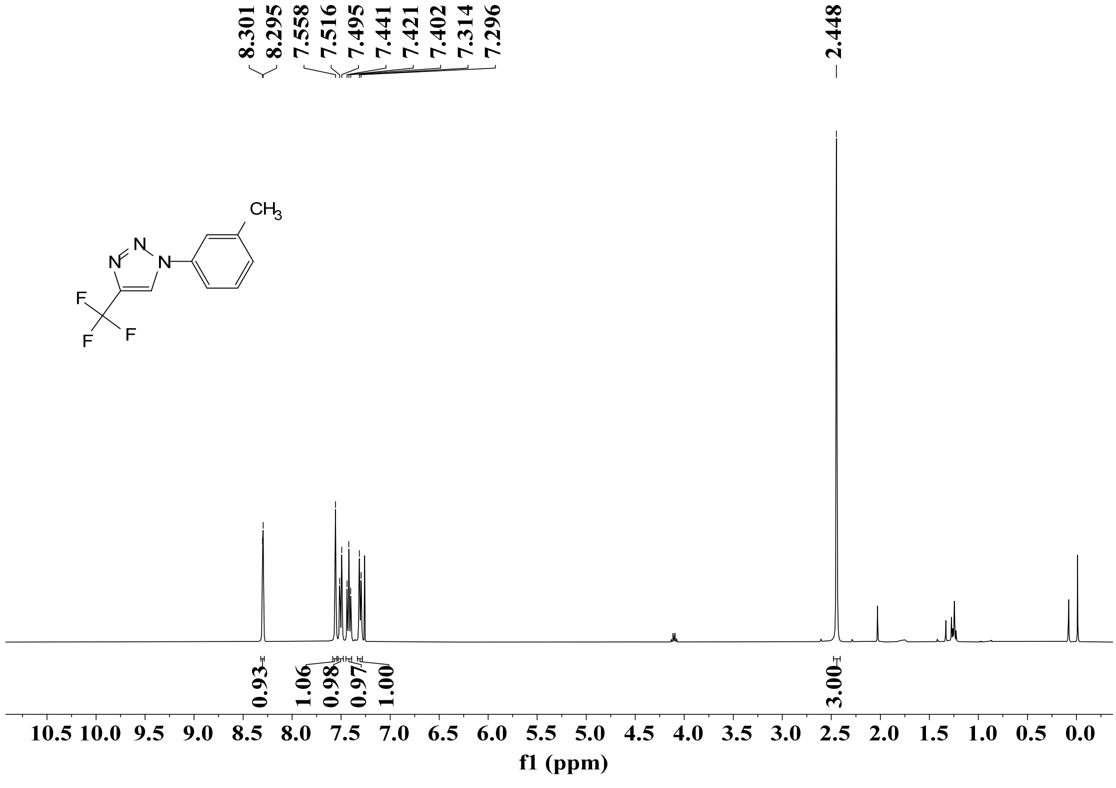
8.301 8.295 7.558 7.516 7.495 7.441 7.421 7.402 7.314 7.296
2.448
CH3
N N N
F F F
0.93 1.06 0.98 0.97 1.00
3.00
10.5 10.0 9.5 9.0 8.5 8.0 7.5 7.0 6.5 6.0 5.5 5.0 4.5 4.0 3.5 3.0 2.5 2.0 1.5 1.0 0.5 0.0
f1 (ppm)

3c-¹H NMR

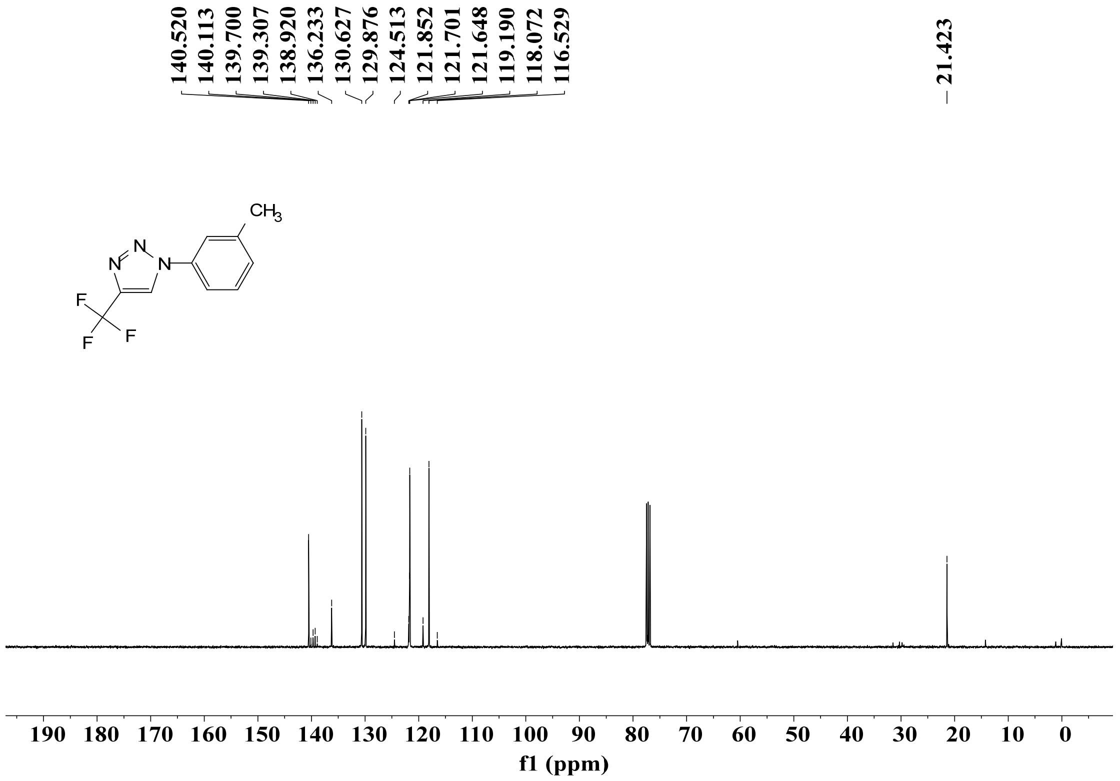

3c-^{13}C NMR

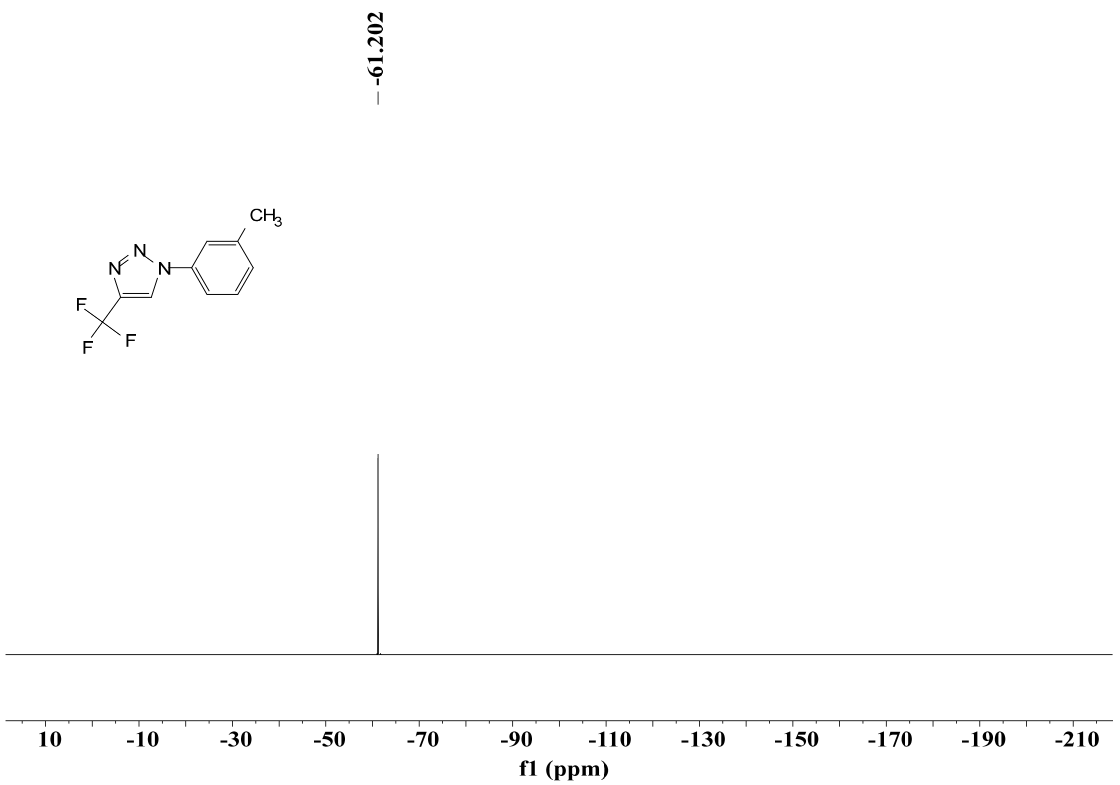

3c-^{19}F NMR

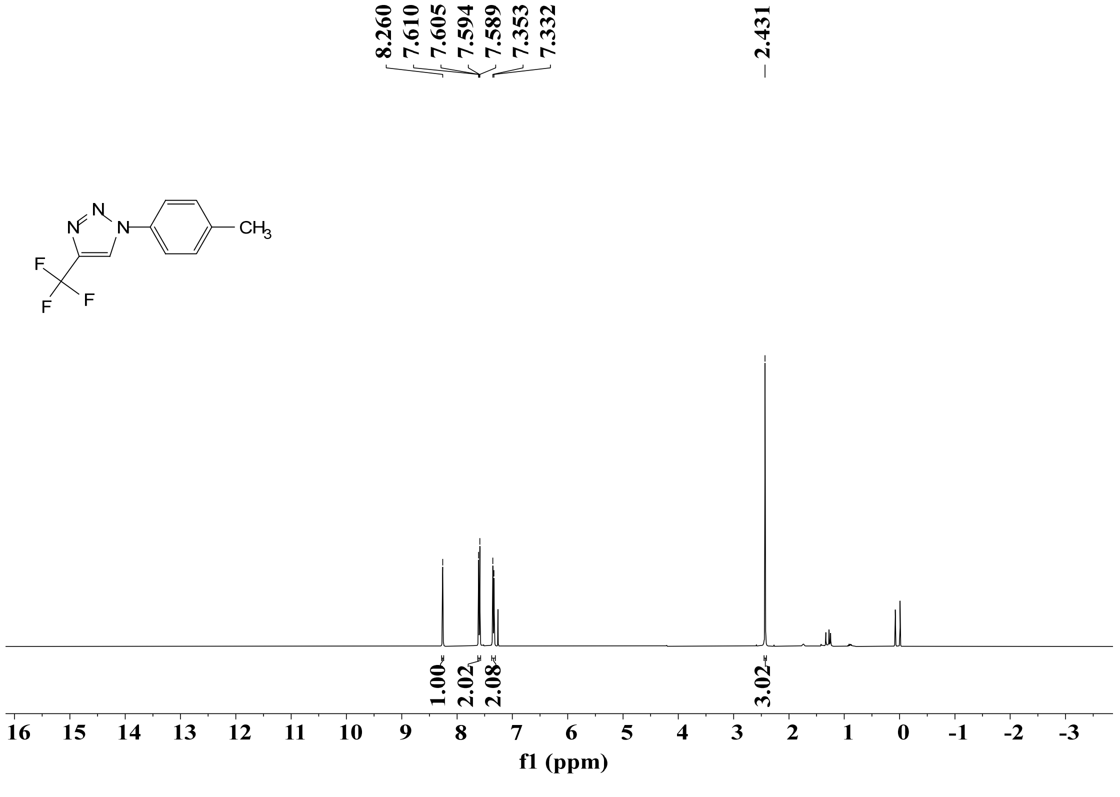

3d-^{1}H NMR

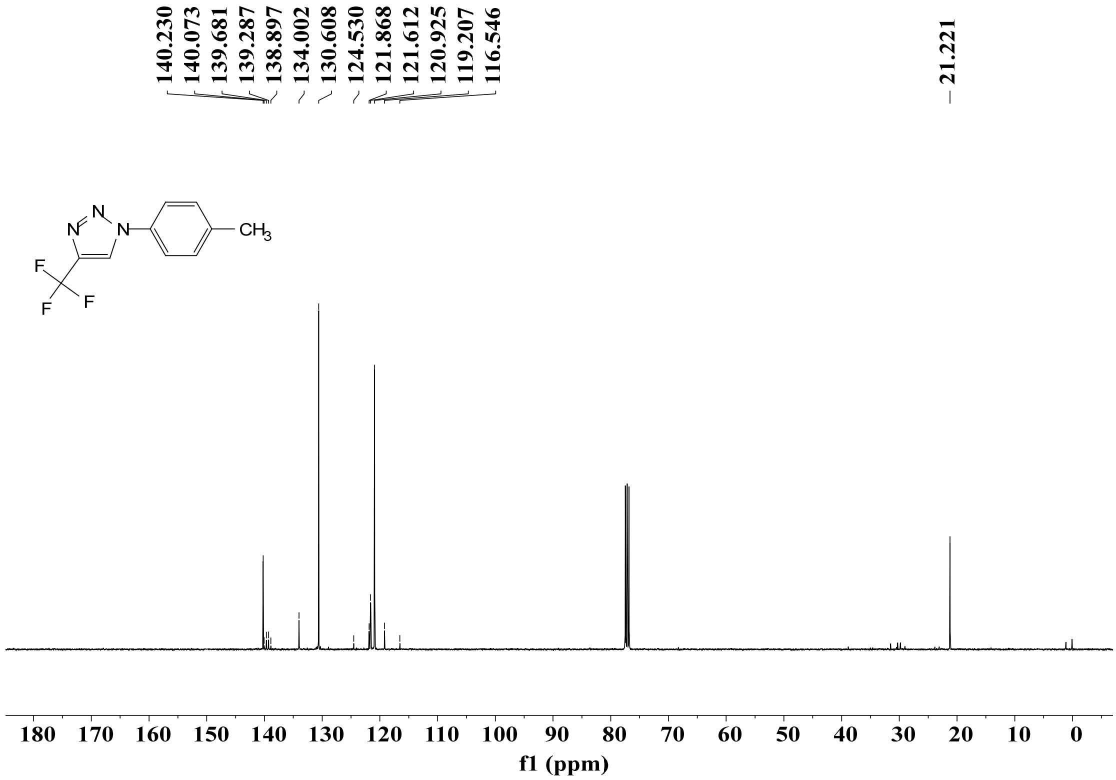
140.230
140.073
139.681
139.287
138.897
134.002
130.608
124.530
121.868
121.612
120.925
119.207
116.546
21.221
N
N
N
CH3
F
F
F
180 170 160 150 140 130 120 110 100 90 80 70 60 50 40 30 20 10 0
f1 (ppm)

3d-^{13}C NMR

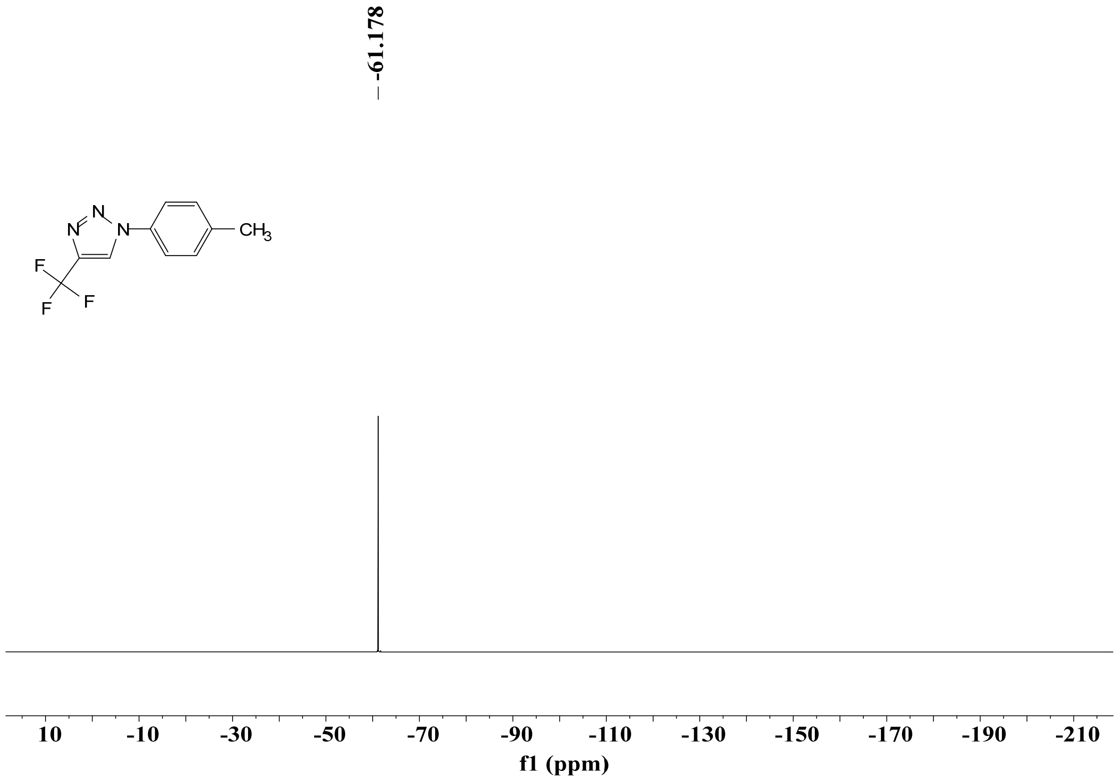

3d-^{19}F NMR

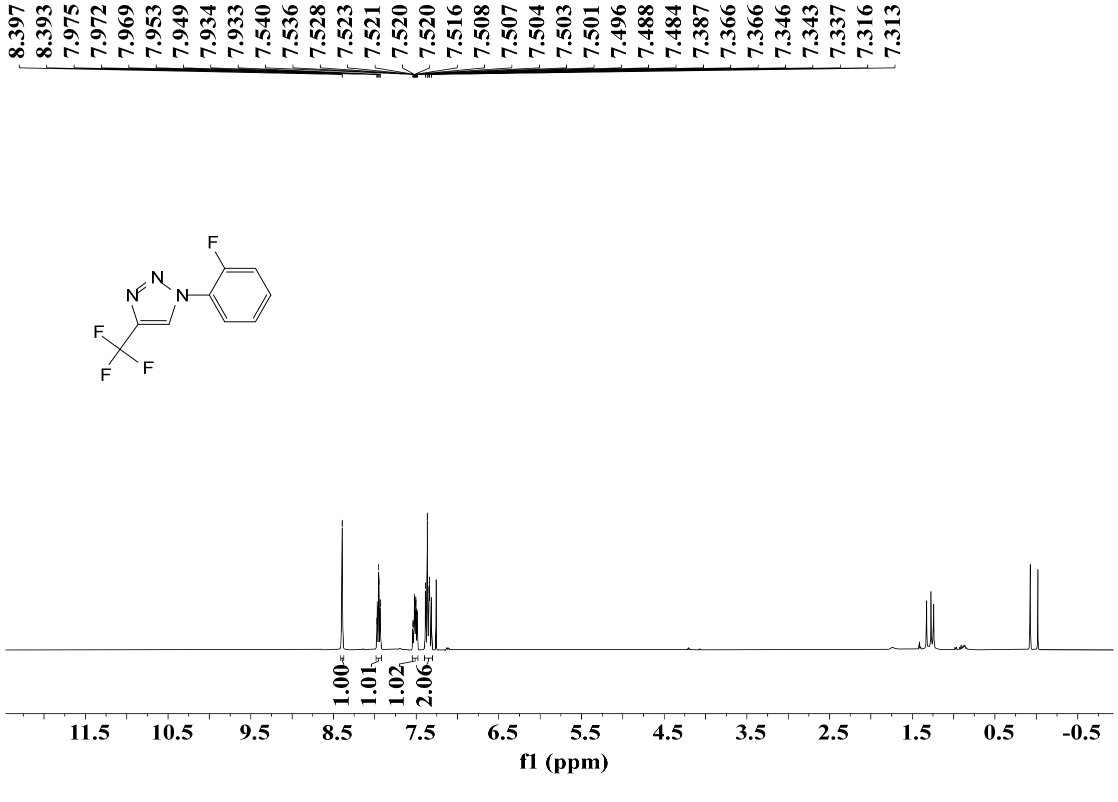

3e-^{1}H NMR

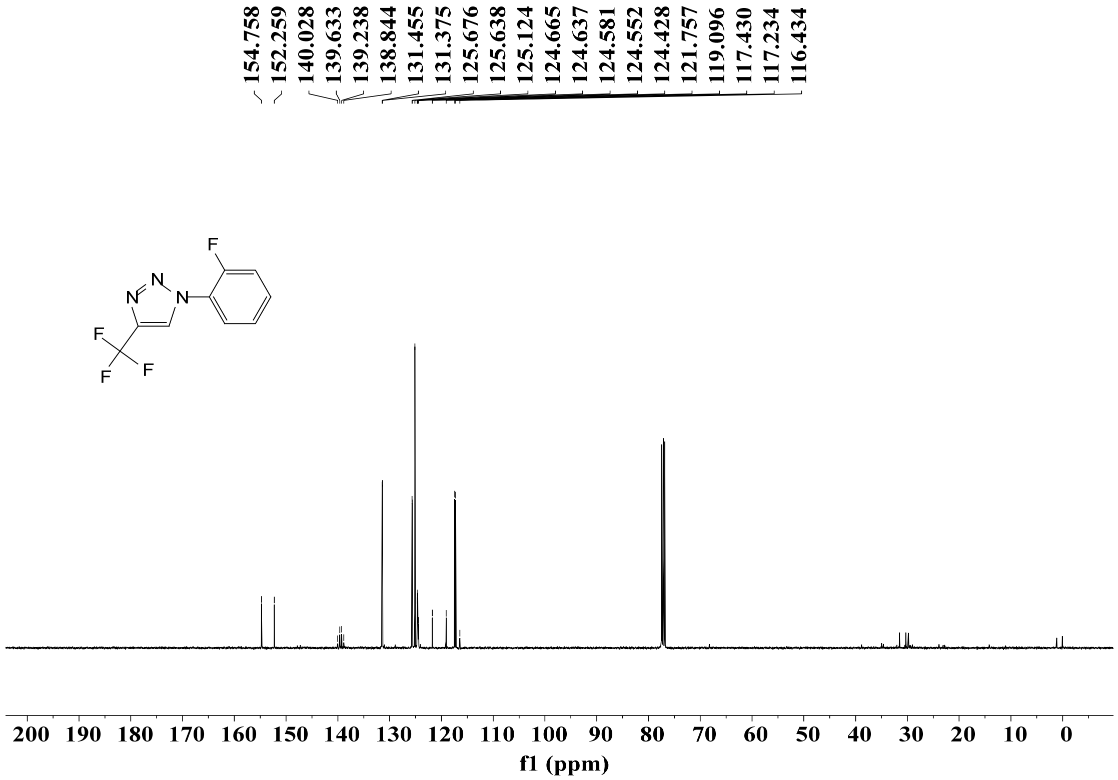
154.758
152.259
140.028
139.633
139.238
138.844
131.455
131.375
125.676
125.638
125.124
124.665
124.637
124.581
124.552
124.428
121.757
119.096
117.430
117.234
116.434
F
N
N
N
F
F
F
200 190 180 170 160 150 140 130 120 110 100 90 80 70 60 50 40 30 20 10 0
f1 (ppm)

3e-^{13}C NMR

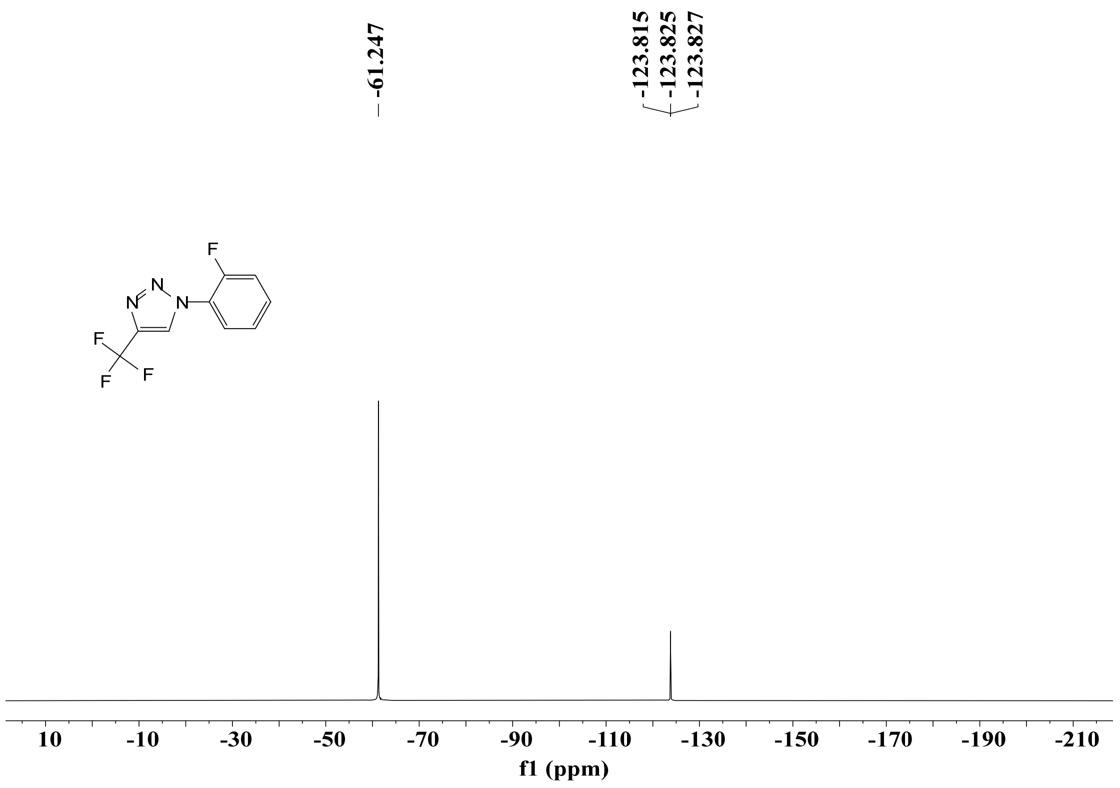

3e-^{19}F NMR

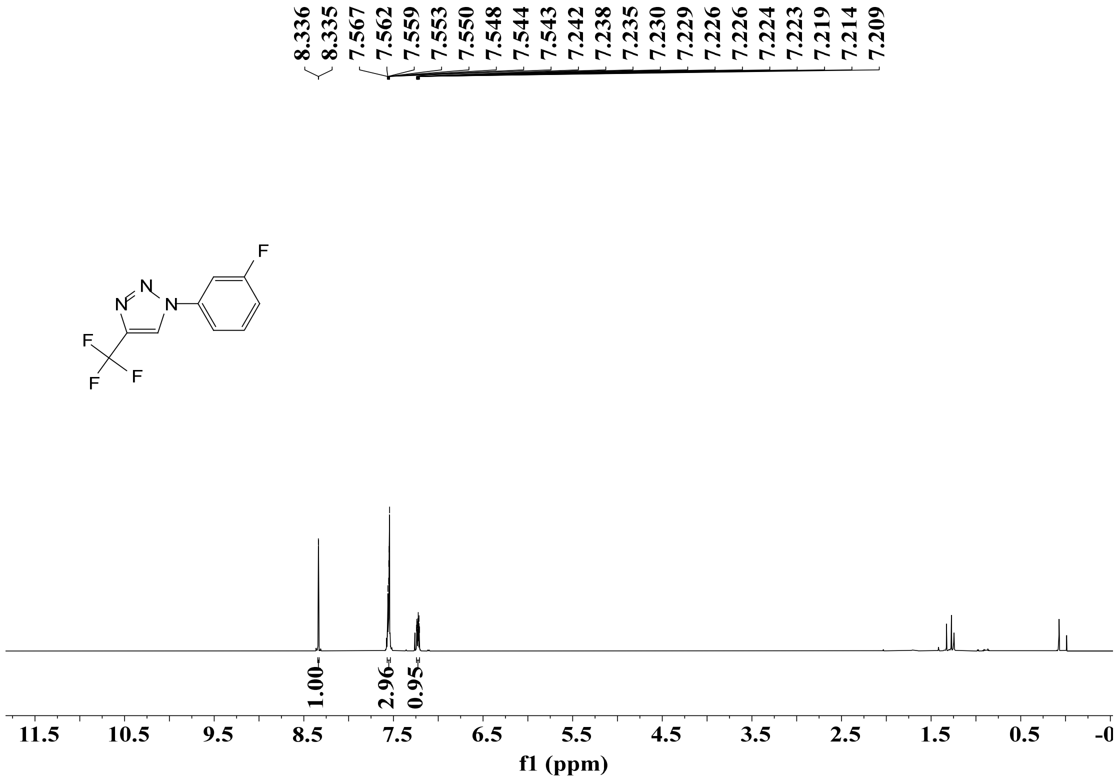

3f-^{1}H NMR

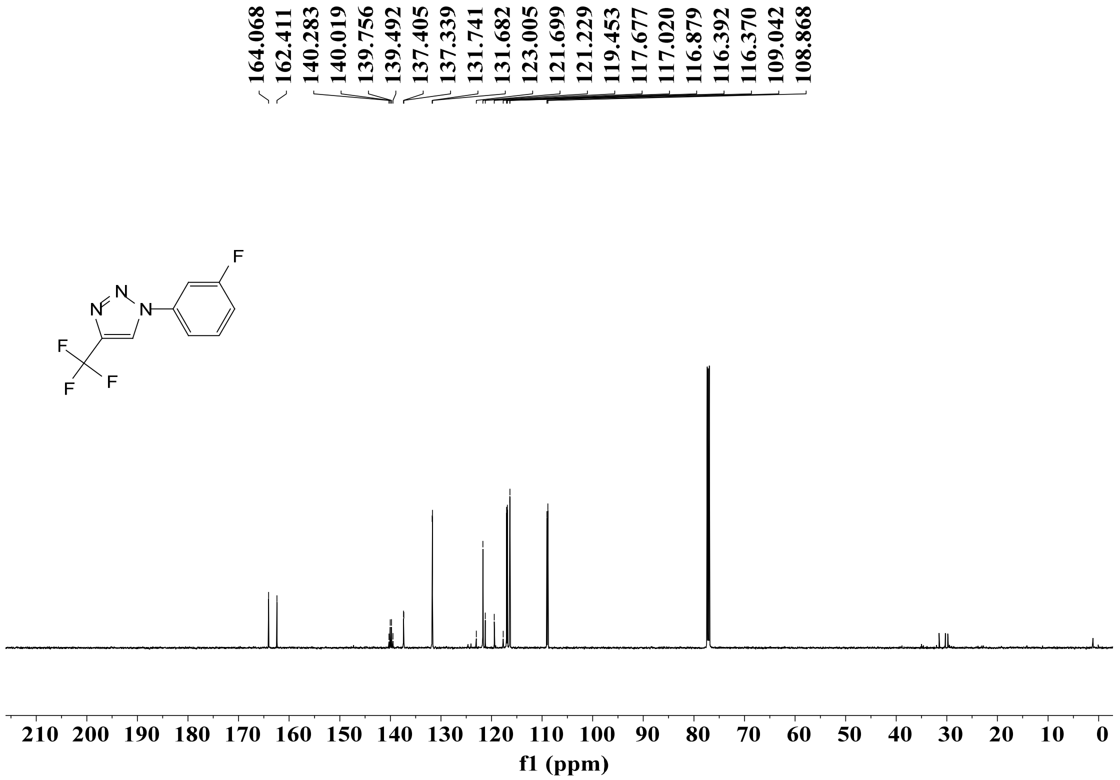
164.068
162.411
140.283
140.019
139.756
139.492
137.405
137.339
131.741
131.682
123.005
121.699
121.229
119.453
117.677
117.020
116.879
116.392
116.370
109.042
108.868
F
N
N
N
F
F
F
210 200 190 180 170 160 150 140 130 120 110 100 90 80 70 60 50 40 30 20 10 0
f1 (ppm)

3f-^{13}C NMR

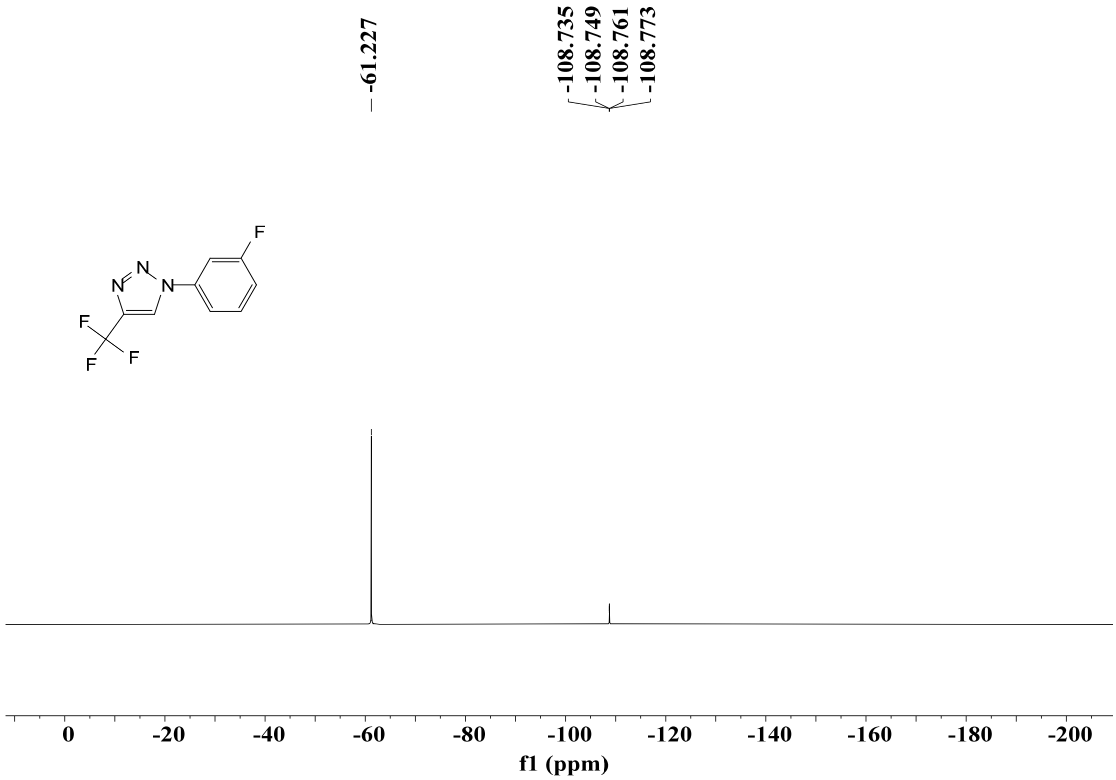

3f-^{19}F NMR

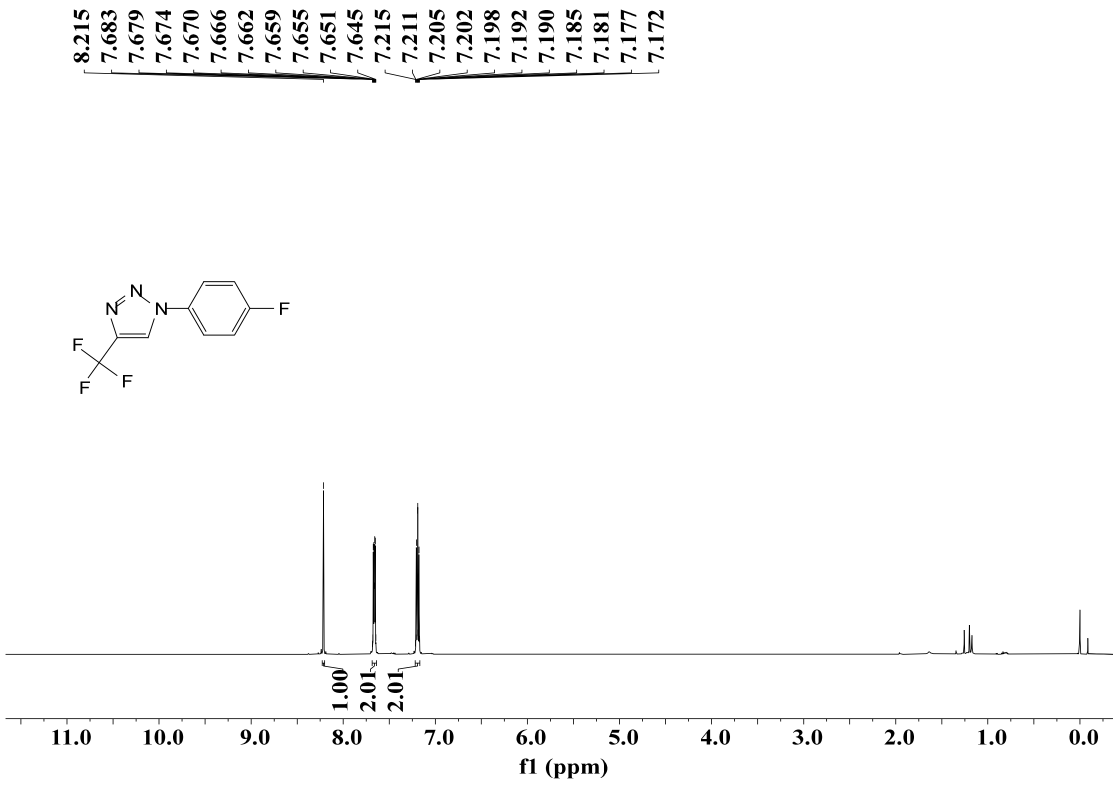

3g-^{1}H NMR

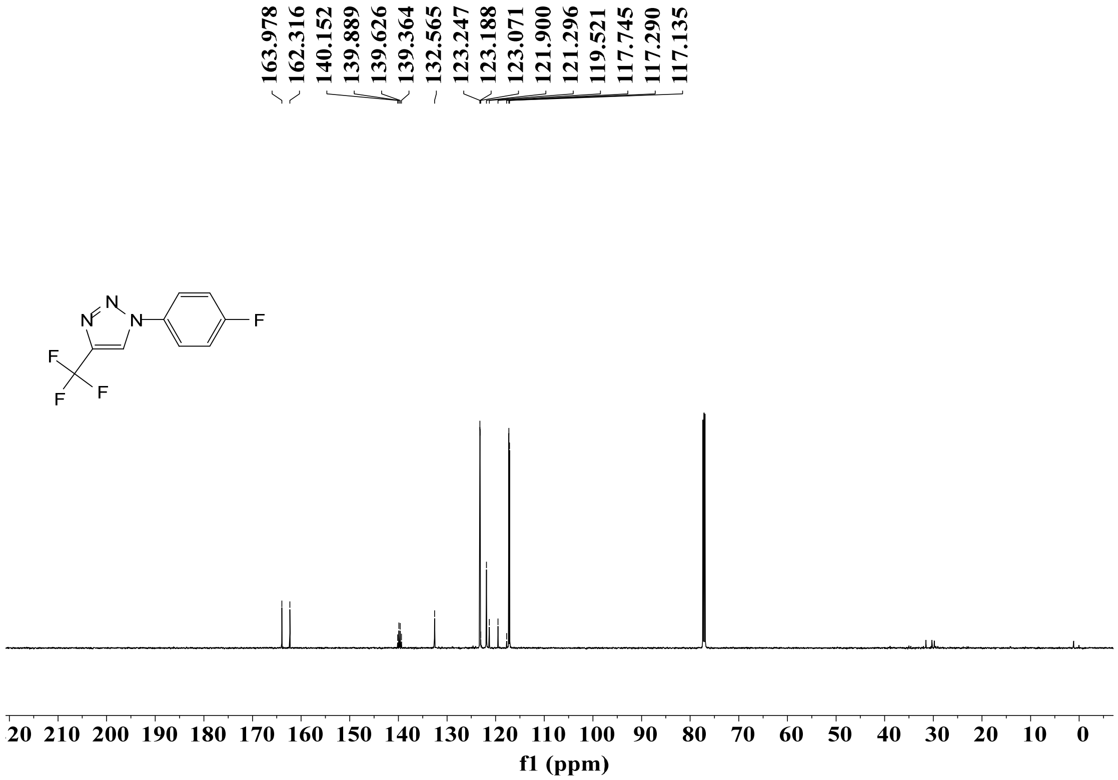

163.978
162.316
140.152
139.889
139.626
139.364
132.565
123.247
123.188
123.071
121.900
121.296
119.521
117.745
117.290
117.135
F
F
F
N
N
N
F
20 210 200 190 180 170 160 150 140 130 120 110 100 90 80 70 60 50 40 30 20 10 0
f1 (ppm)

3g-^{13}C NMR

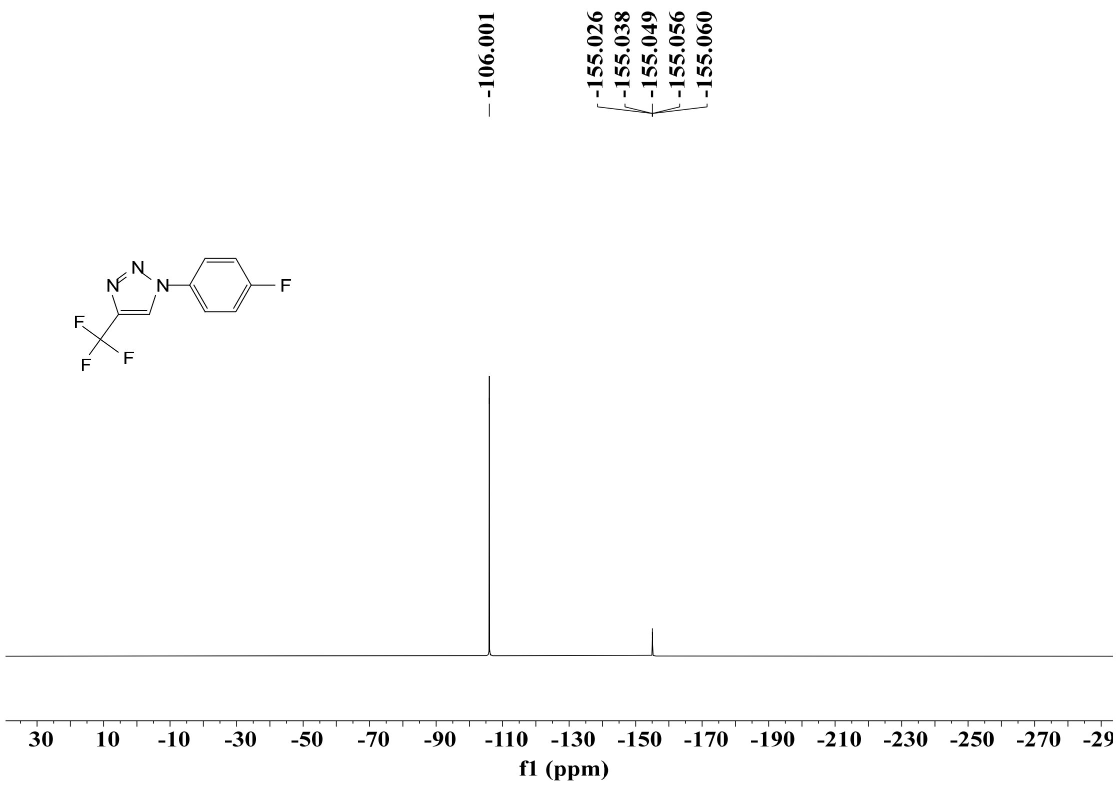

3g-^{19}F NMR

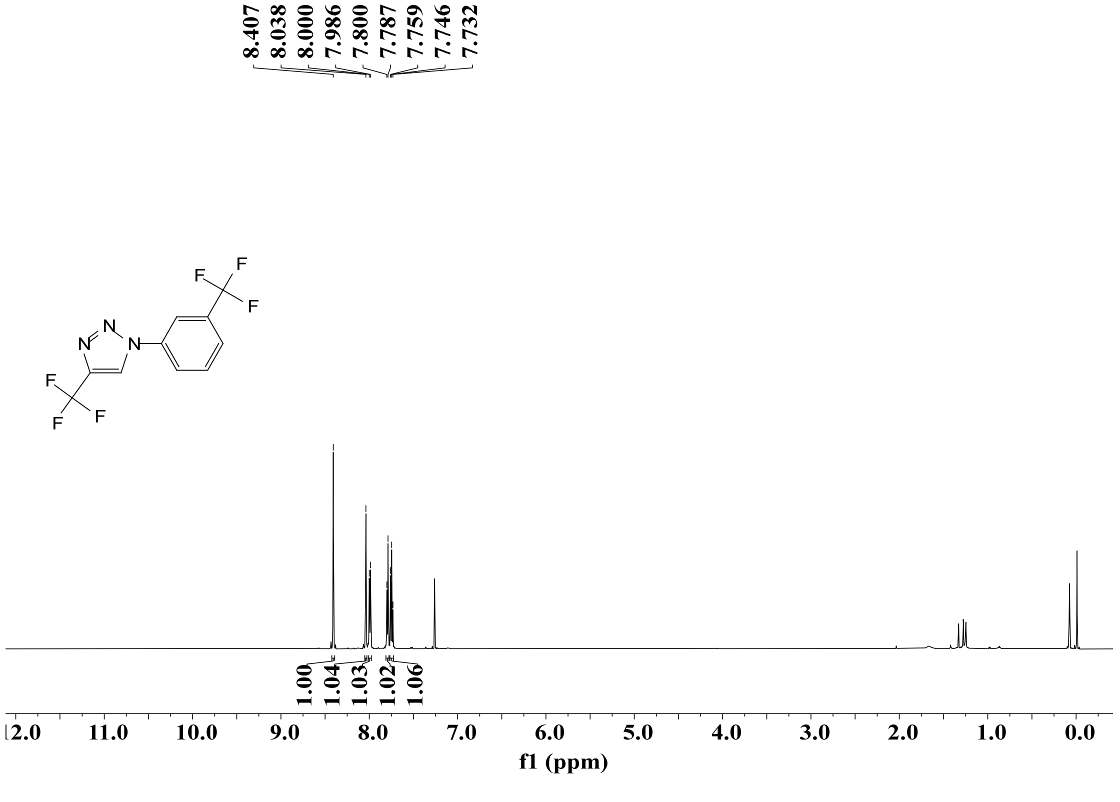

3h-^{1}H NMR

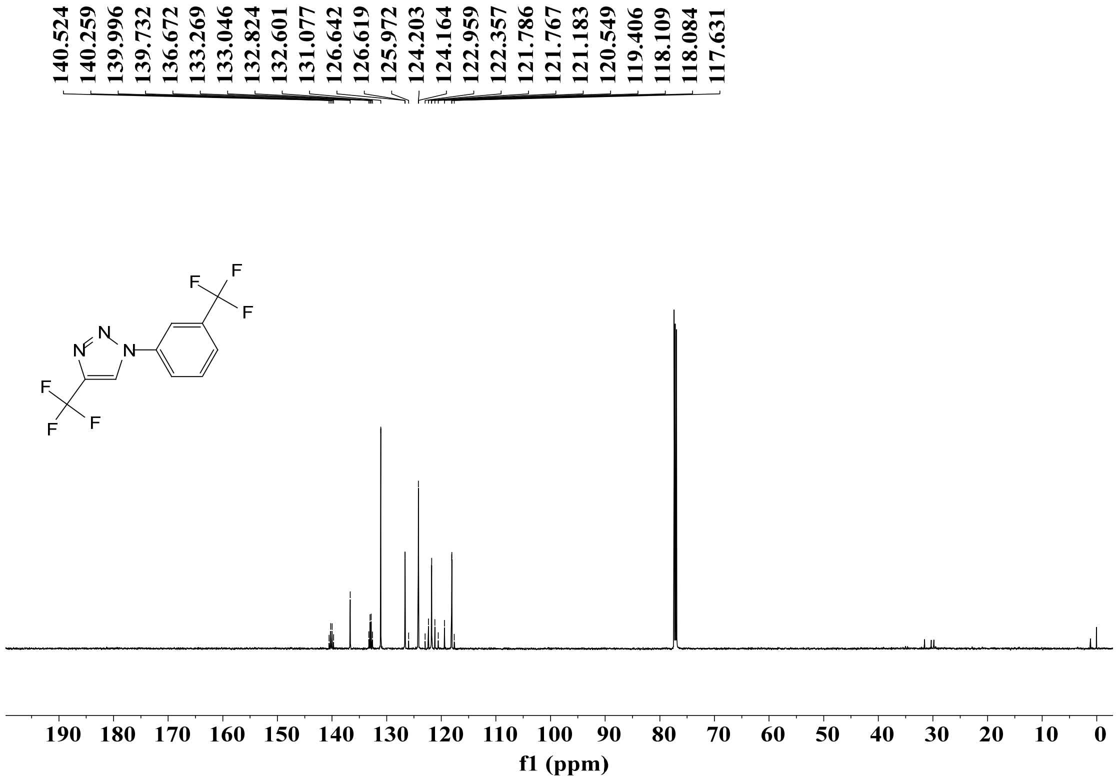
140.524
140.259
139.996
139.732
136.672
133.269
133.046
132.824
132.601
131.077
126.642
126.619
125.972
124.203
124.164
122.959
122.357
121.786
121.767
121.183
120.549
119.406
118.109
118.084
117.631
190 180 170 160 150 140 130 120 110 100 90 80 70 60 50 40 30 20 10 0
f1 (ppm)

3h-^{13}C NMR

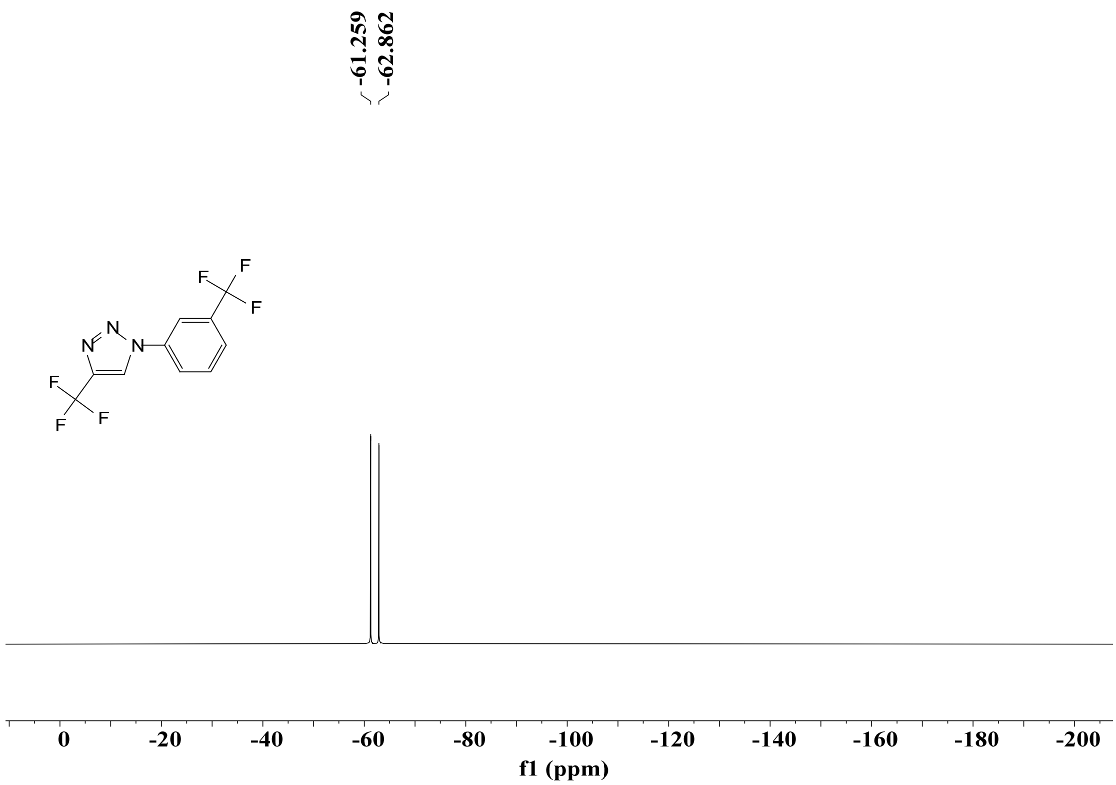

3h-^{19}F NMR

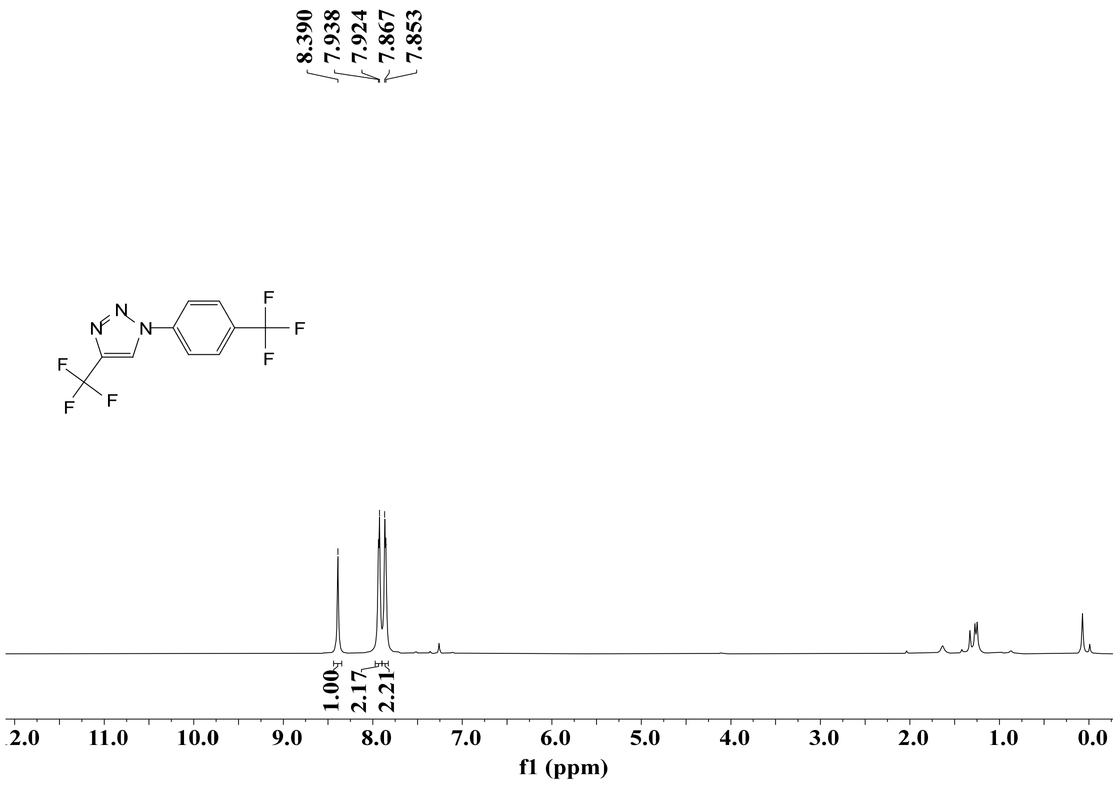

3i-^{1}H NMR

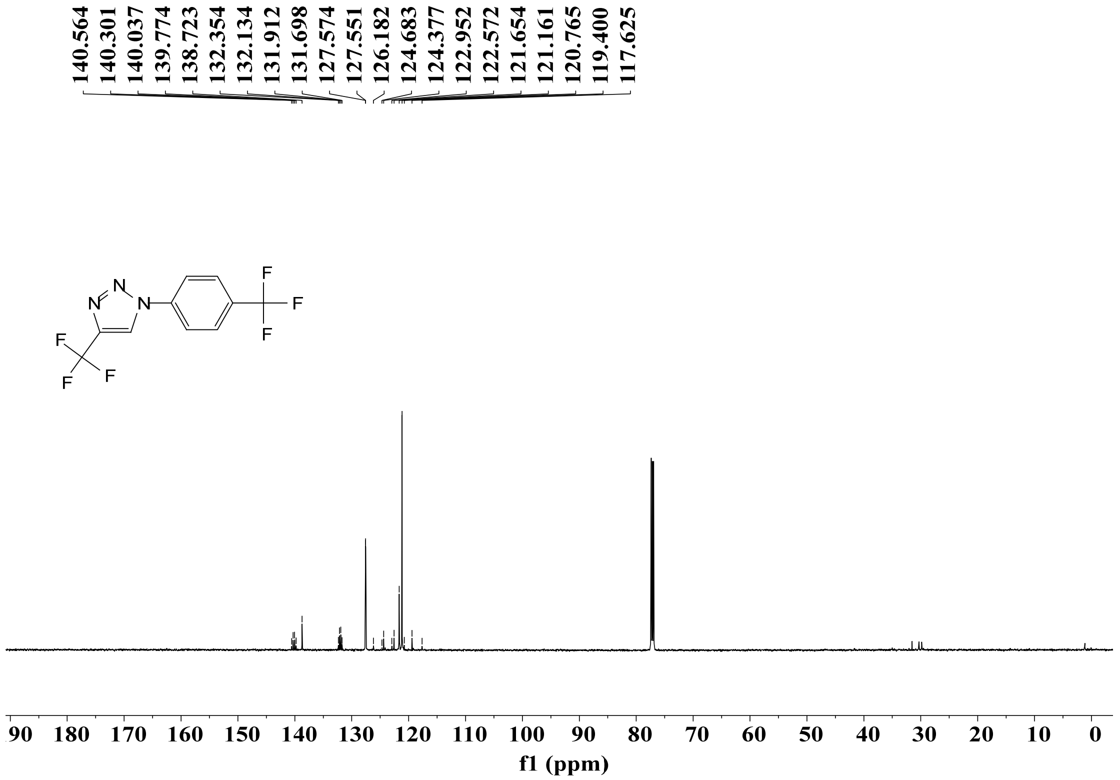
140.564
140.301
140.037
139.774
138.723
132.354
132.134
131.912
131.698
127.574
127.551
126.182
124.683
124.377
122.952
122.572
121.654
121.161
120.765
119.400
117.625
N
N
N
F
F
F
F
F
F
90 180 170 160 150 140 130 120 110 100 90 80 70 60 50 40 30 20 10 0
f1 (ppm)

3i-^{13}C NMR

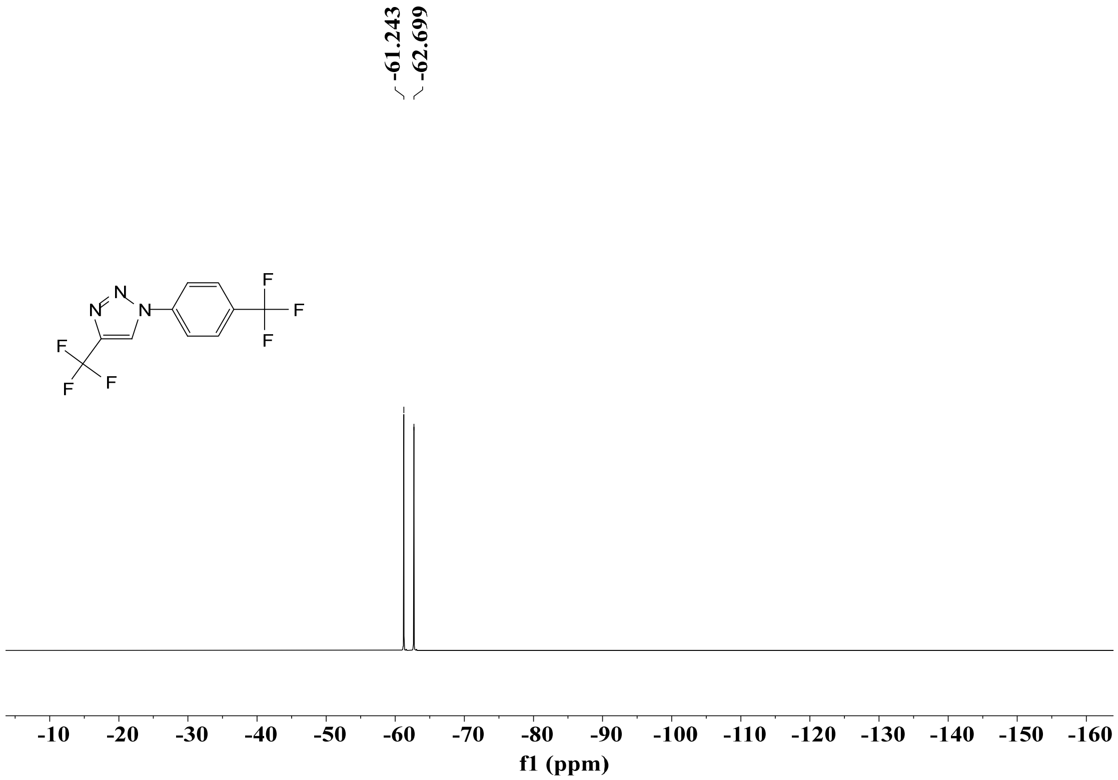

3i-^{19}F NMR

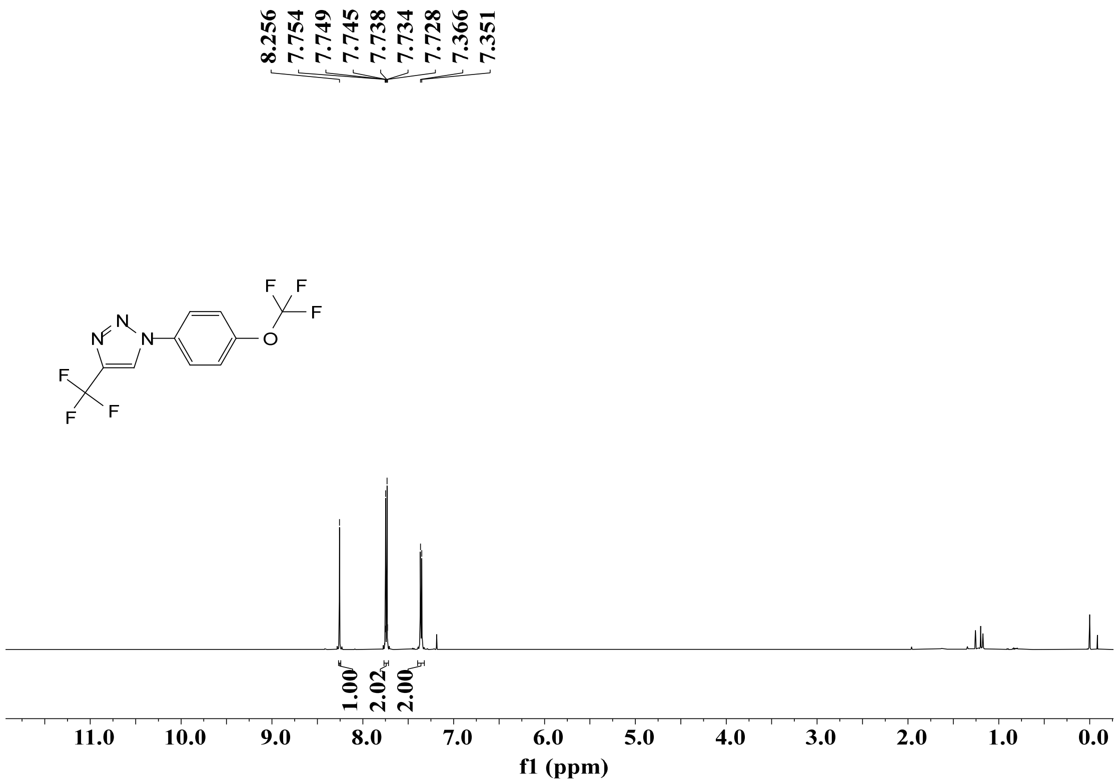

3j-^{1}H NMR

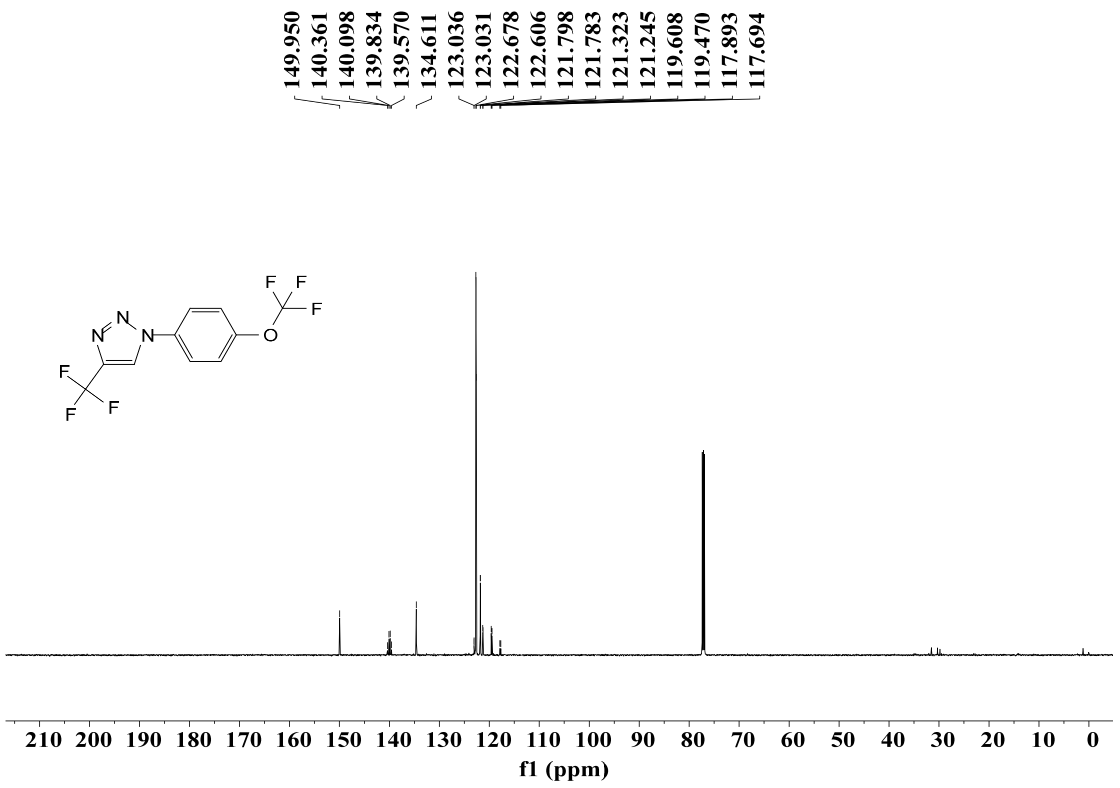

149.950
140.361
140.098
139.834
139.570
134.611
123.036
123.031
122.678
122.606
121.798
121.783
121.323
121.245
119.608
119.470
117.893
117.694
210 200 190 180 170 160 150 140 130 120 110 100 90 80 70 60 50 40 30 20 10 0
f1 (ppm)

3j-^{13}C NMR

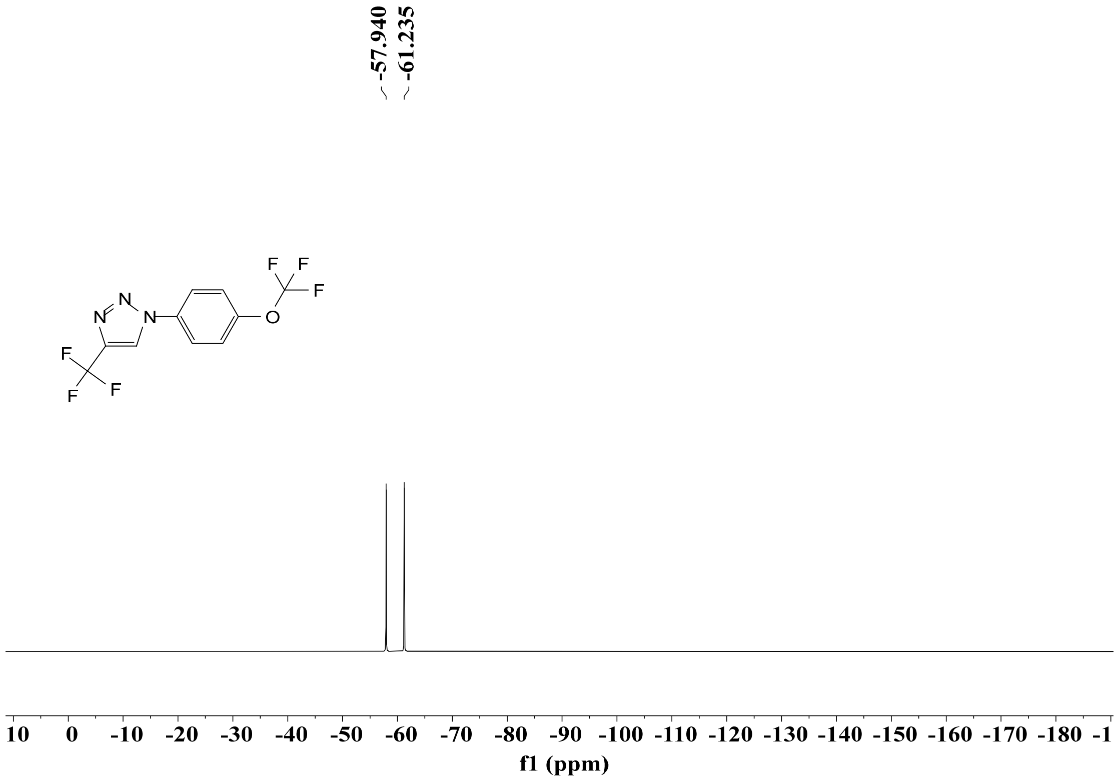

3j-^{19}F NMR

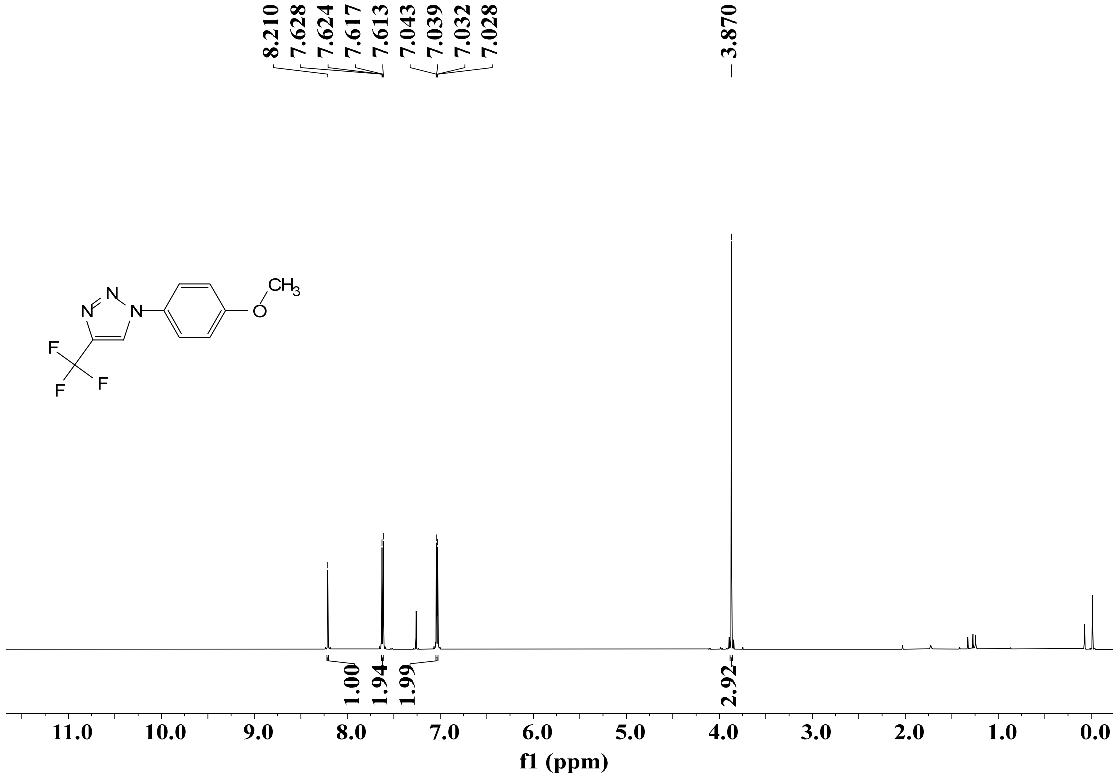

3k-^{1}H NMR

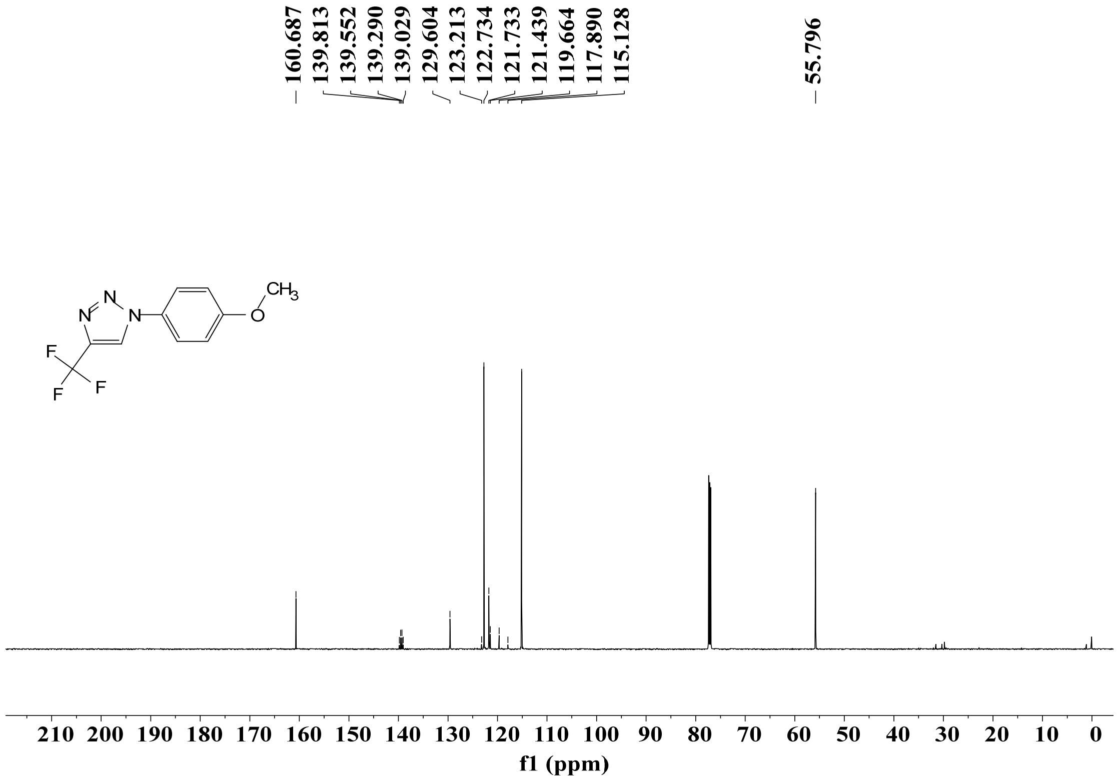
160.687
139.813
139.552
139.290
139.029
129.604
123.213
122.734
121.733
121.439
119.664
117.890
115.128
55.796
CH3
N
N
N
O
F
F
F
210 200 190 180 170 160 150 140 130 120 110 100 90 80 70 60 50 40 30 20 10 0
f1 (ppm)

3k-^{13}C NMR

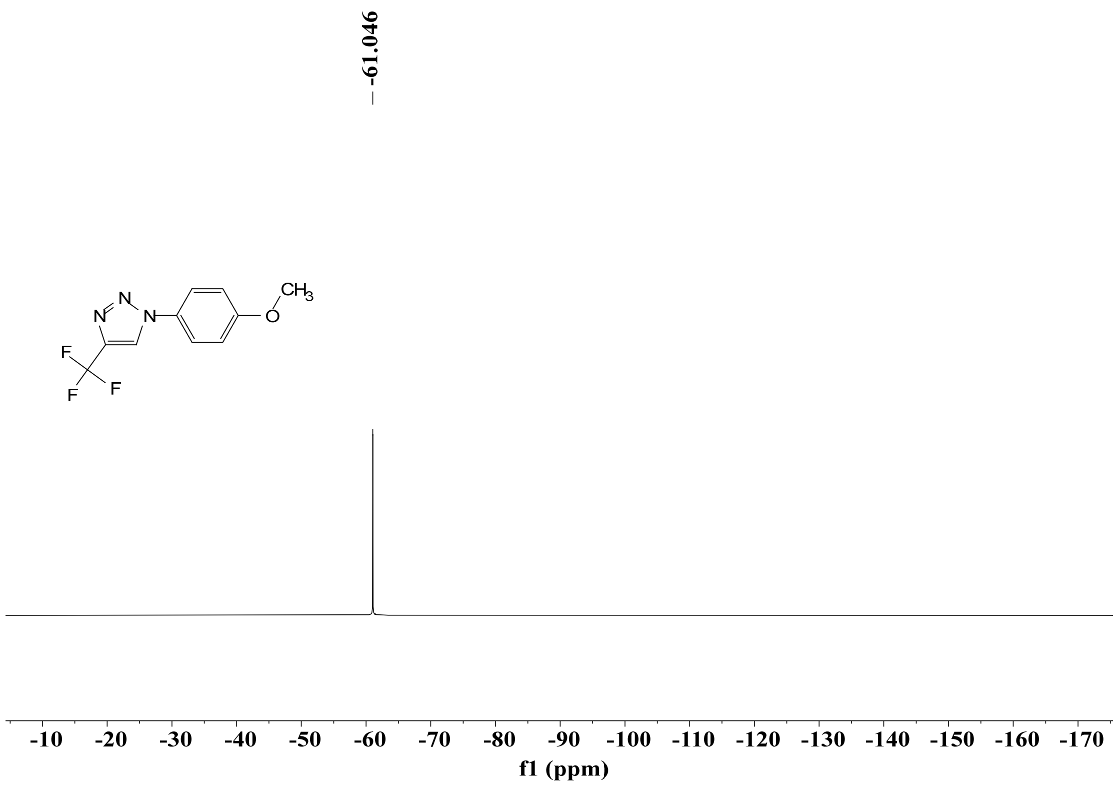

3k-^{19}F NMR

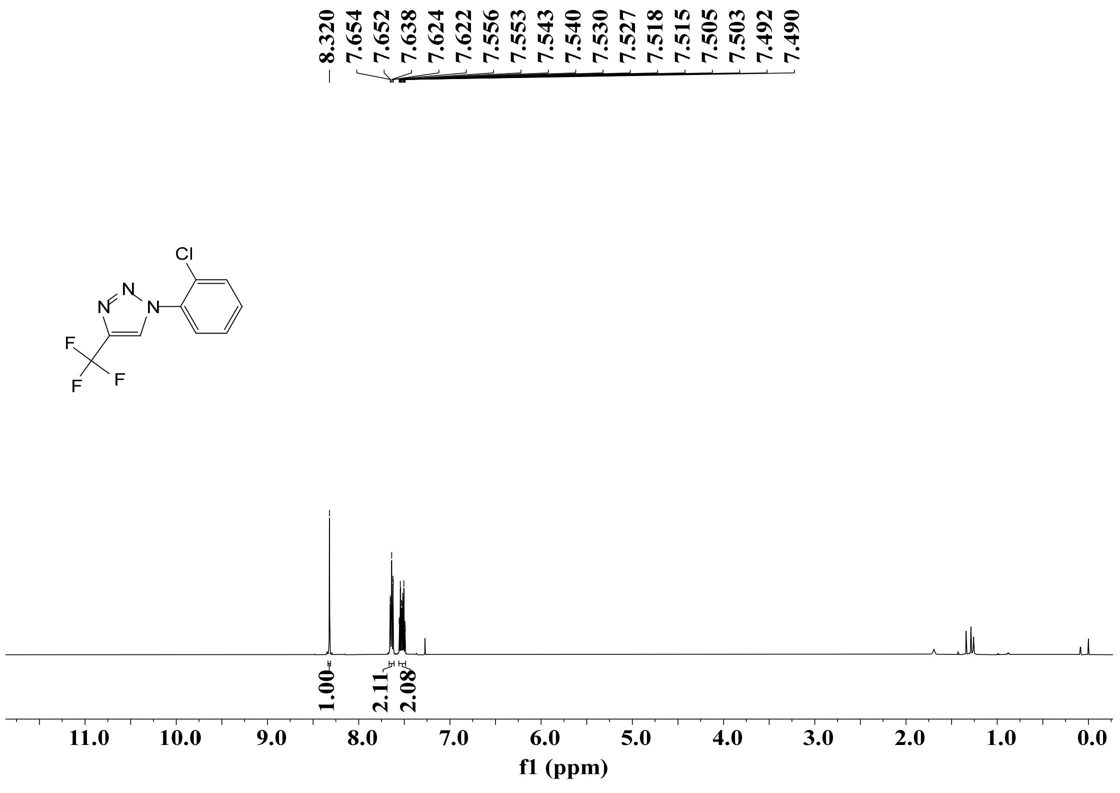

3l-^{1}H NMR

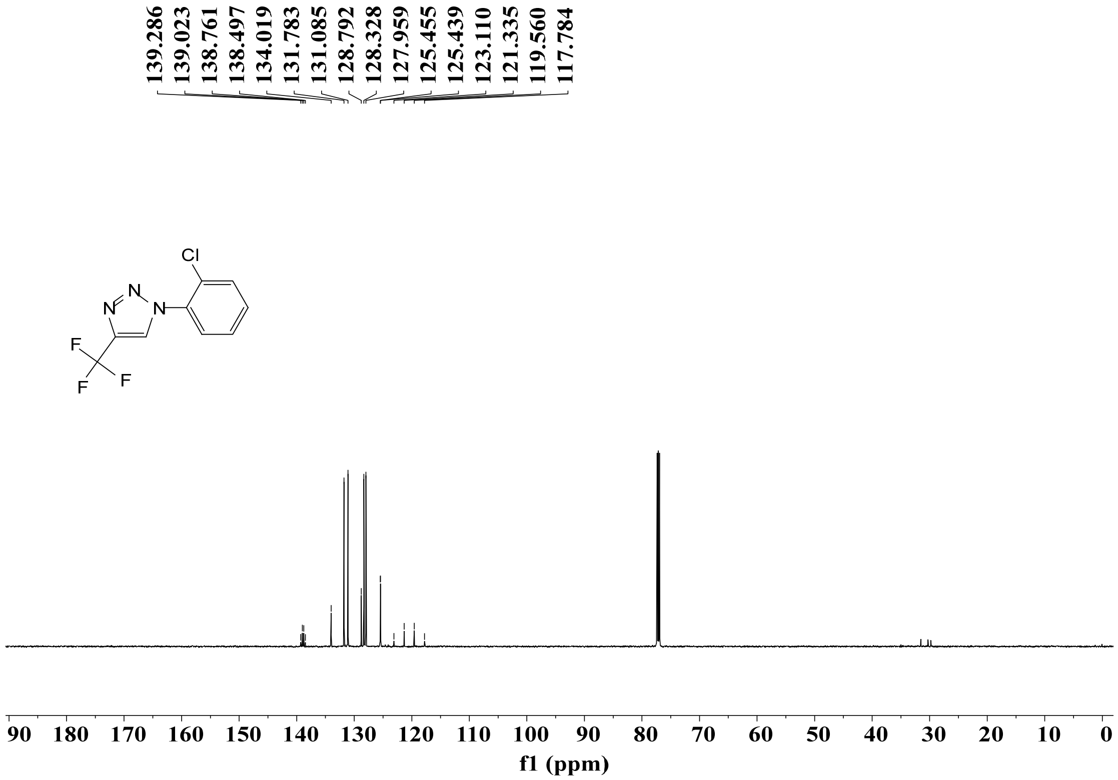
139.286
139.023
138.761
138.497
134.019
131.783
131.085
128.792
128.328
127.959
125.455
125.439
123.110
121.335
119.560
117.784
Cl
N
N
N
F
F
F
90 180 170 160 150 140 130 120 110 100 90 80 70 60 50 40 30 20 10 0
f1 (ppm)

3l-^{13}C NMR

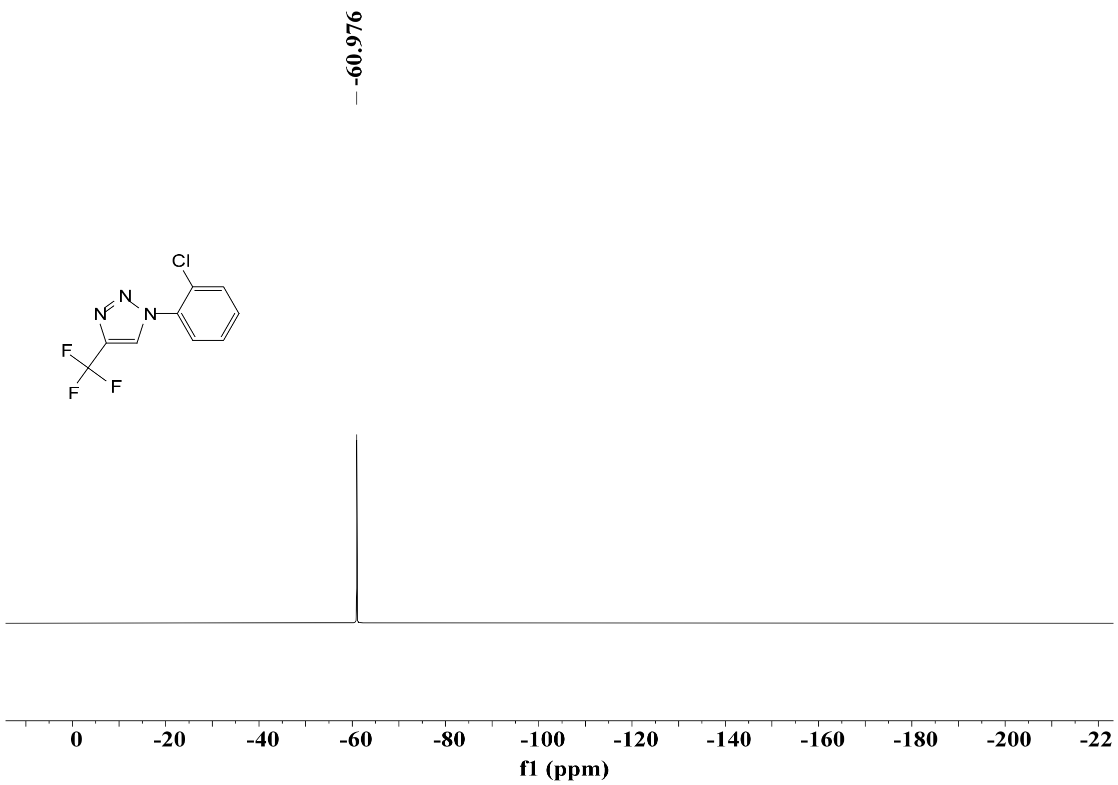

3l-^{19}F NMR

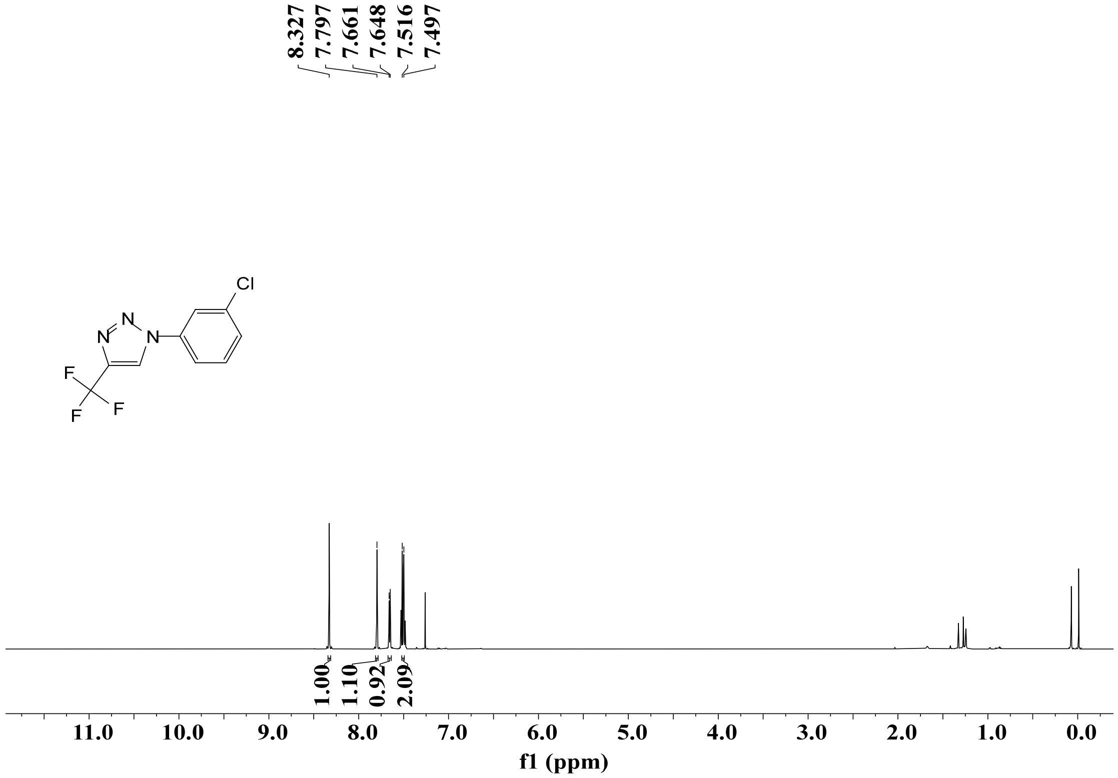

3m-^{1}H NMR

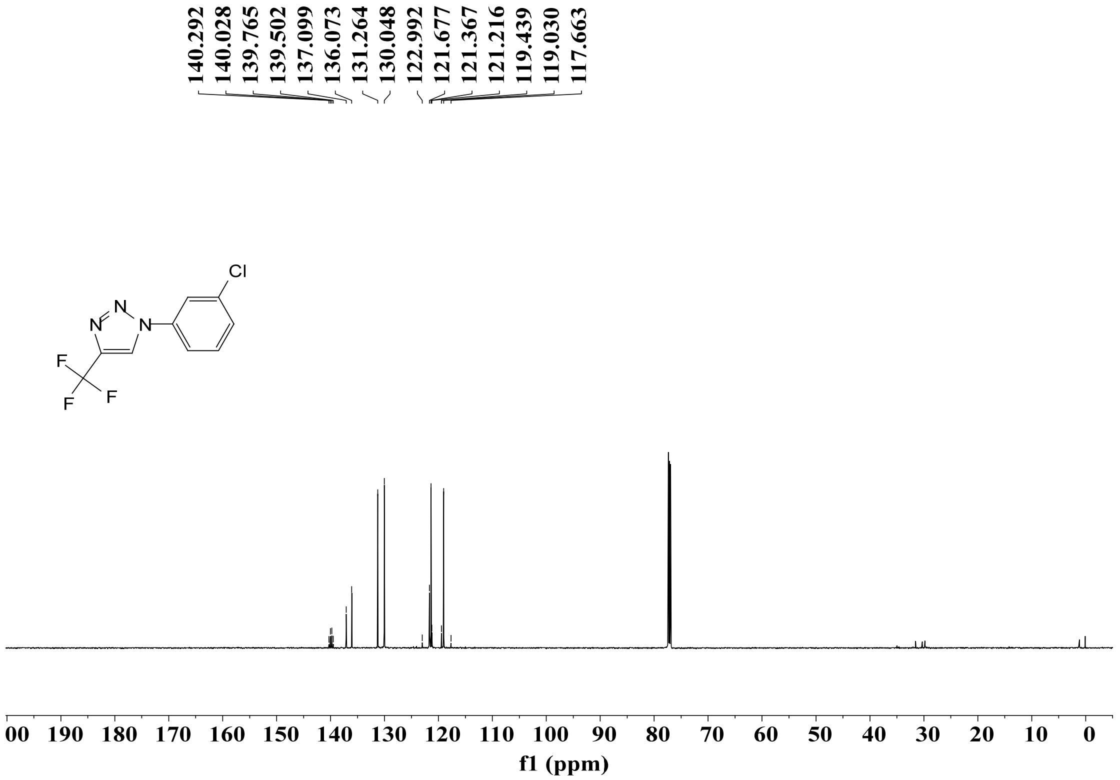
140.292
140.028
139.765
139.502
137.099
136.073
131.264
130.048
122.992
121.677
121.367
121.216
119.439
119.030
117.663
Cl
N
N
N
F
F
F
00 190 180 170 160 150 140 130 120 110 100 90 80 70 60 50 40 30 20 10 0
f1 (ppm)

3m-^{13}C NMR

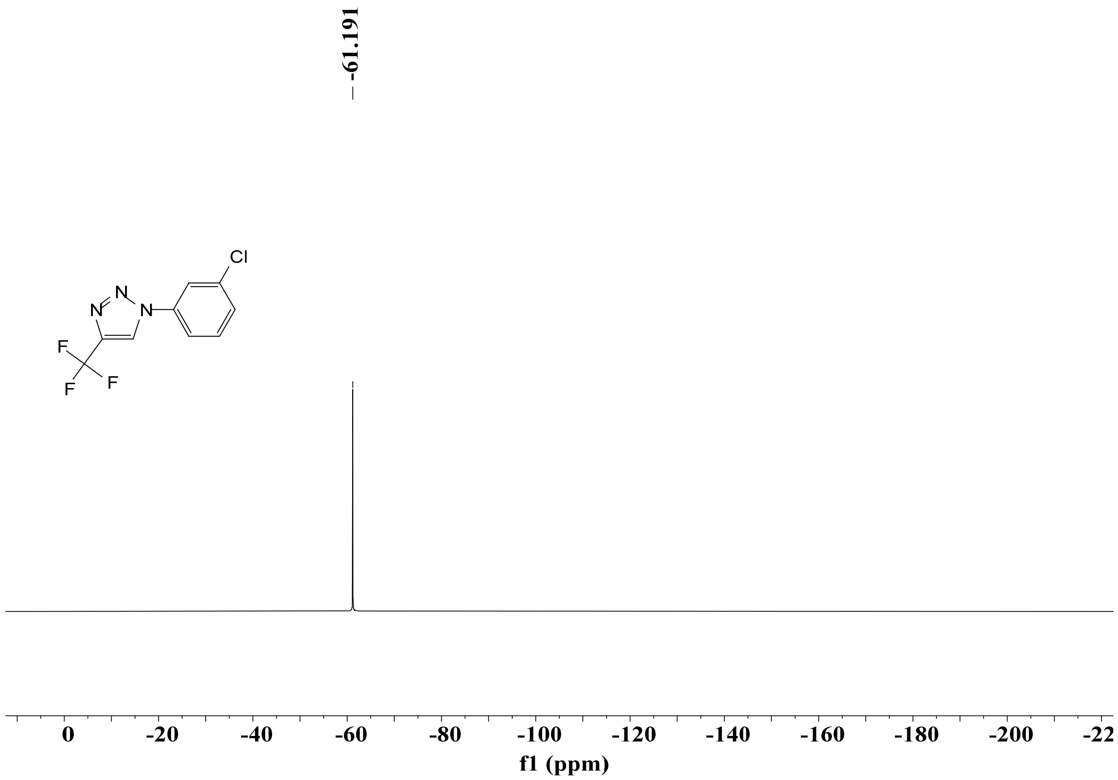

3m-^{19}F NMR

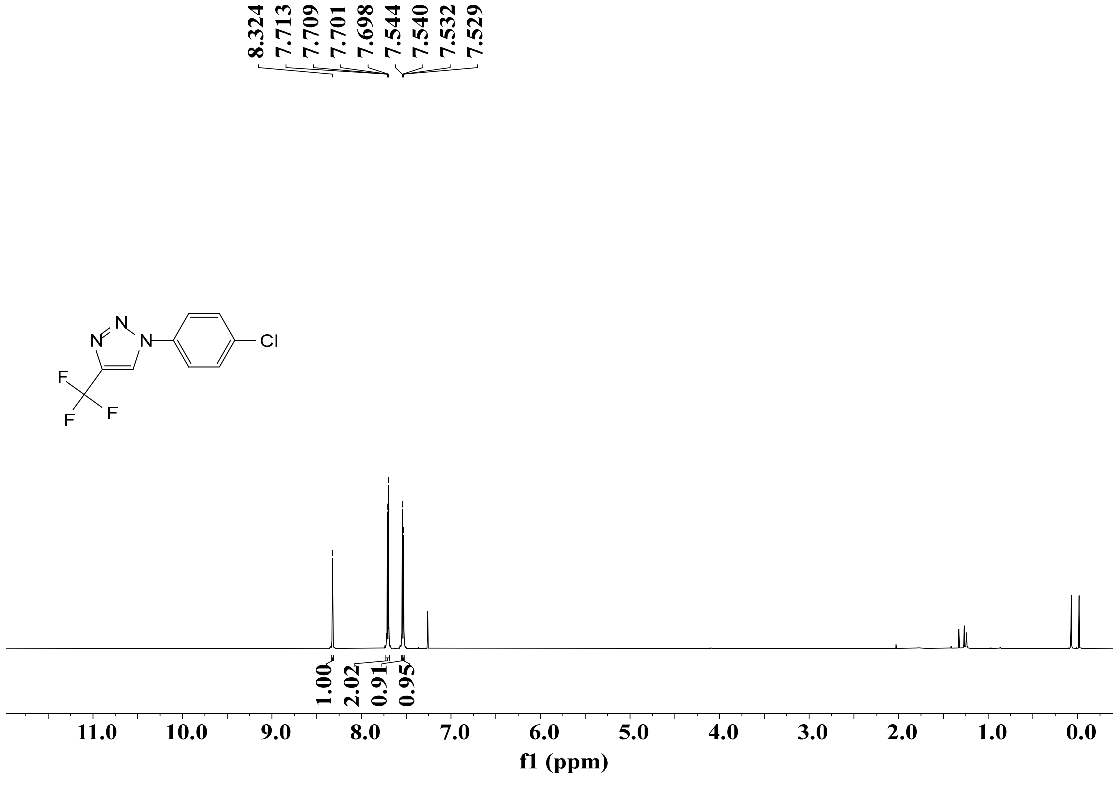

3n-^{1}H NMR

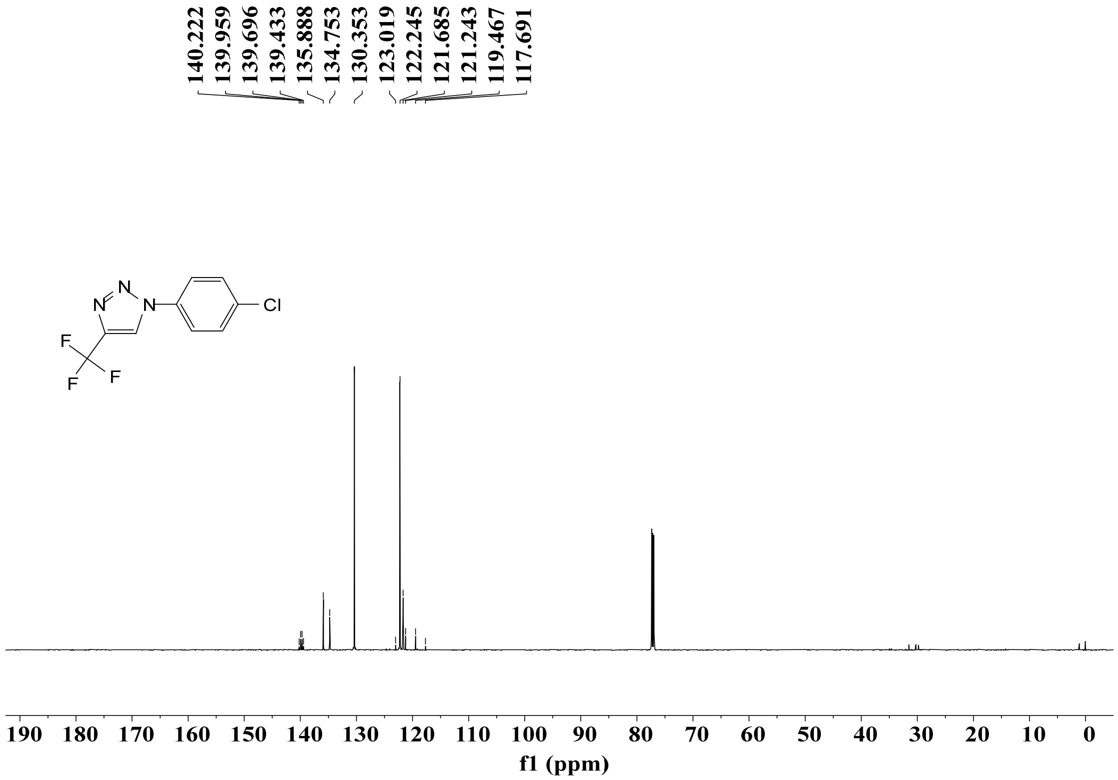
140.222
139.959
139.696
139.433
135.888
134.753
130.353
123.019
122.245
121.685
121.243
119.467
117.691
N
N
N
Cl
F
F
F
190
180
170
160
150
140
130
120
110
100
90
80
70
60
50
40
30
20
10
0
f1 (ppm)

3n-^{13}C NMR

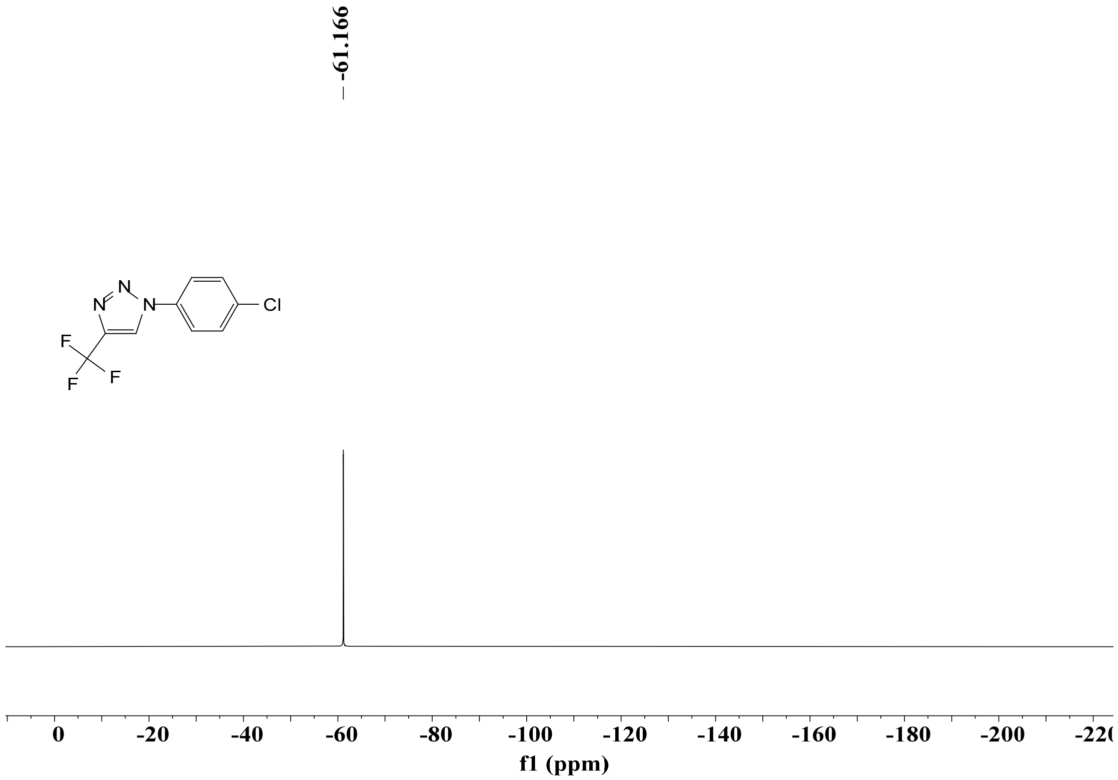

3n-^{19}F NMR

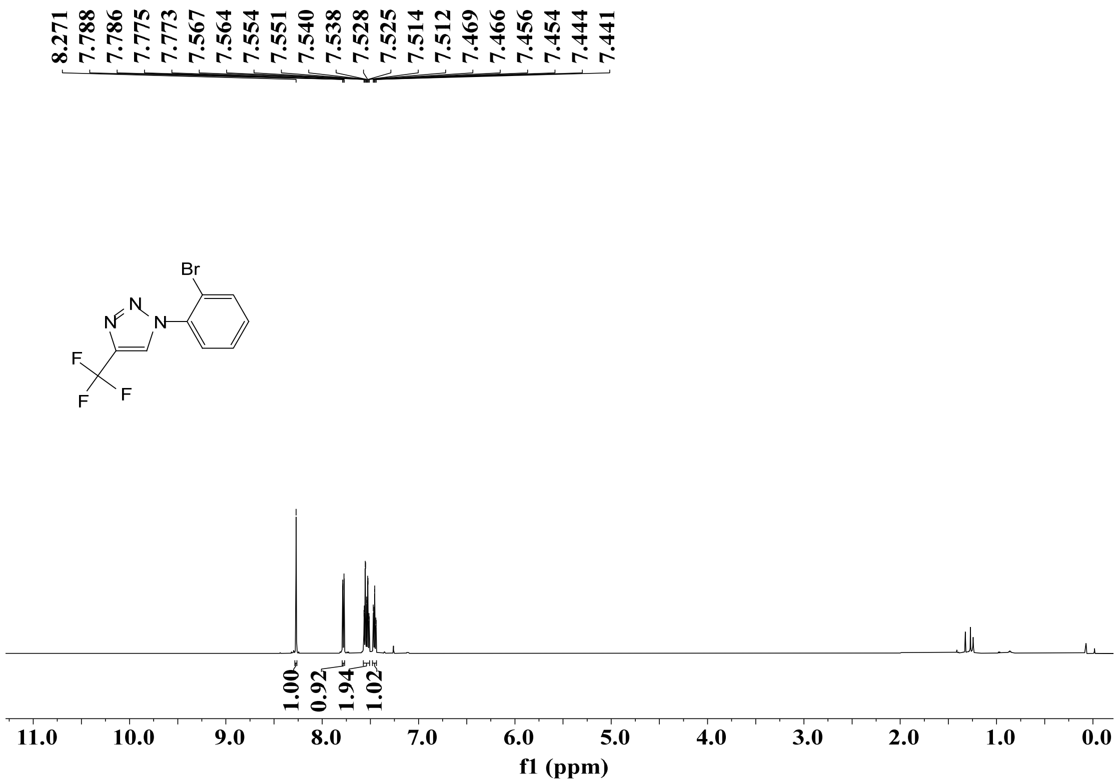

3o-^{1}H NMR

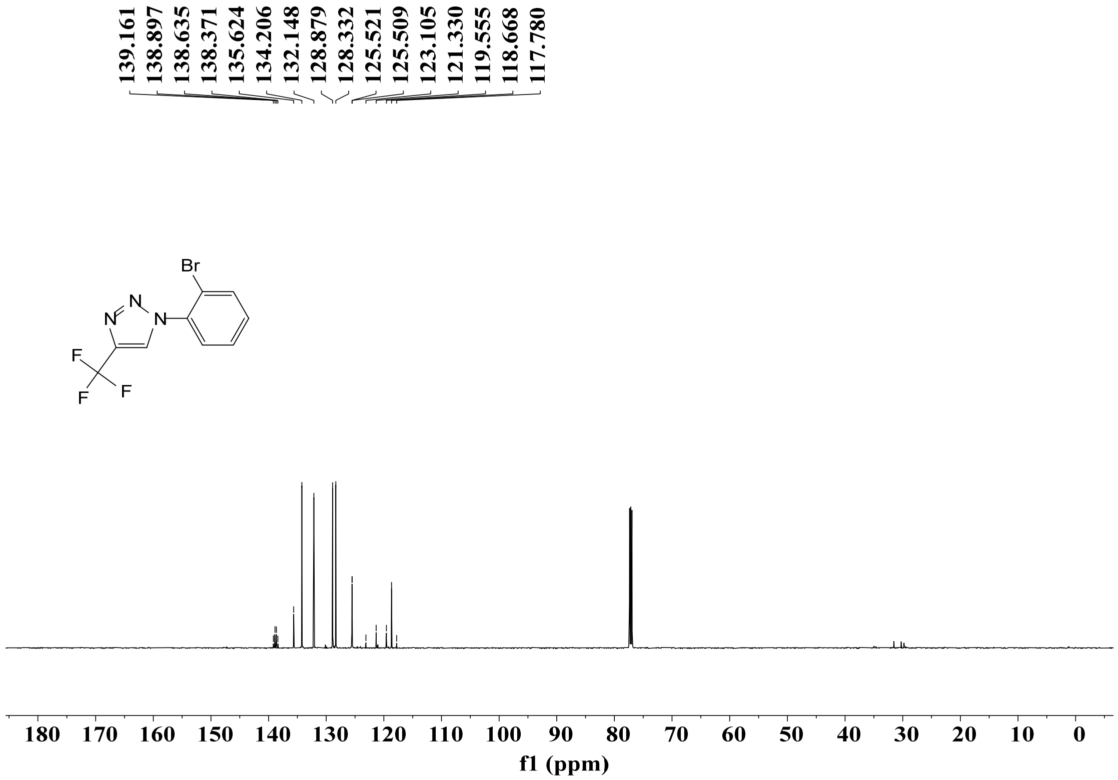
139.161
138.897
138.635
138.371
135.624
134.206
132.148
128.879
128.332
125.521
125.509
123.105
121.330
119.555
118.668
117.780
Br
N
N
N
F
F
F
180 170 160 150 140 130 120 110 100 90 80 70 60 50 40 30 20 10 0
f1 (ppm)

3o-^{13}C NMR

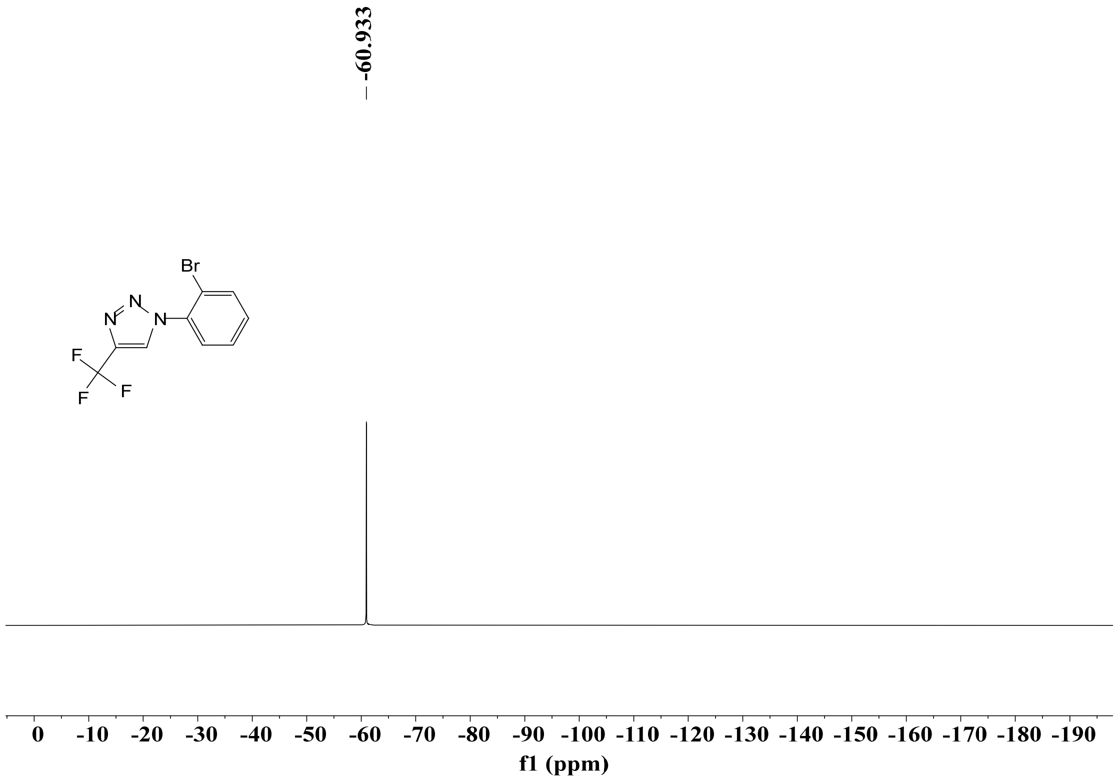

3o-^{19}F NMR

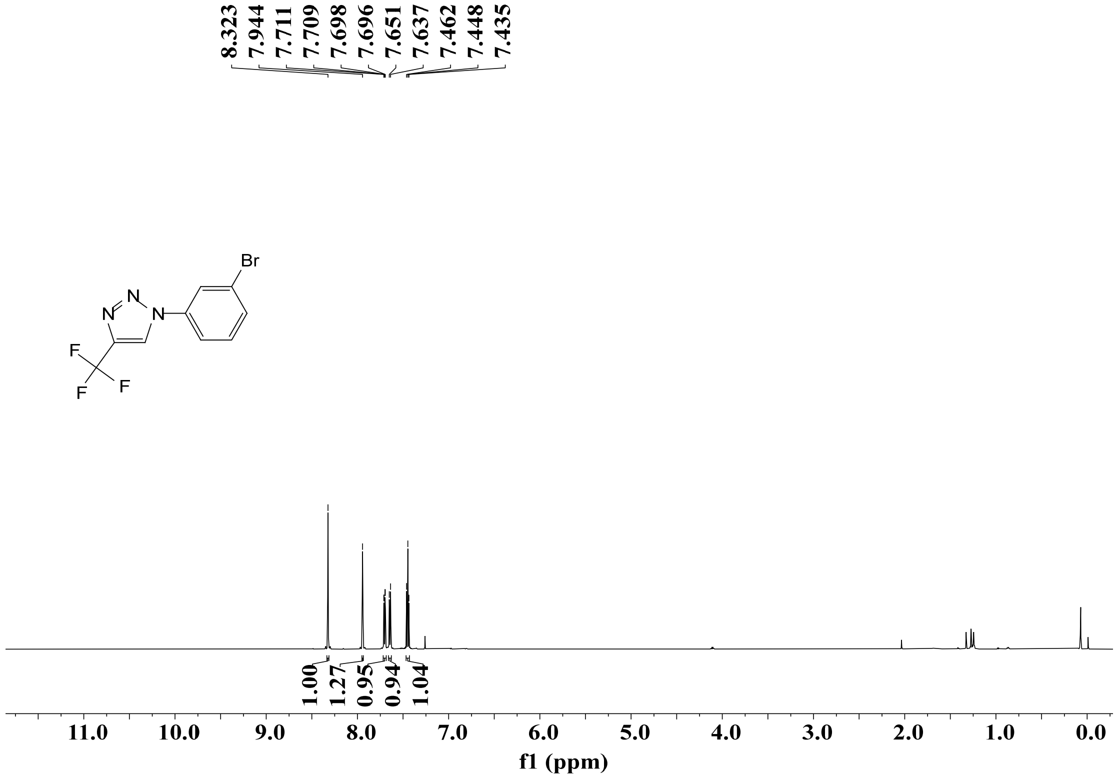

3p-^{1}H NMR

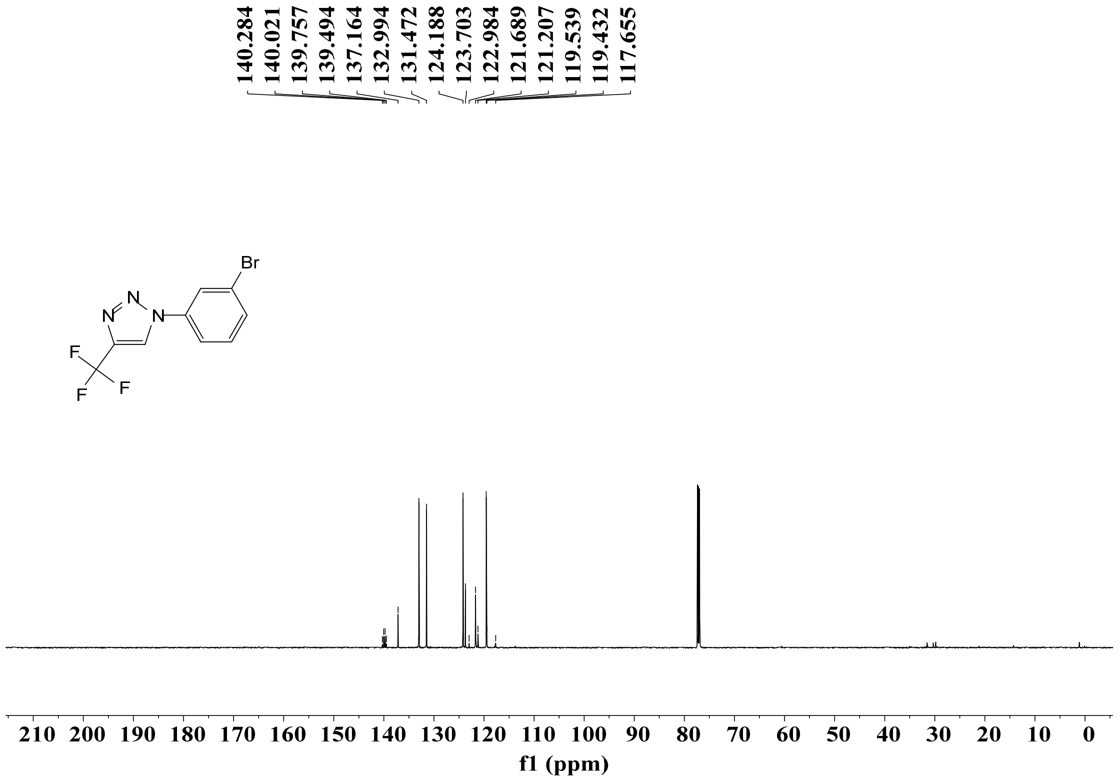
140.284
140.021
139.757
139.494
137.164
132.994
131.472
124.188
123.703
122.984
121.689
121.207
119.539
119.432
117.655
Br
N
N
N
F
F
F
210 200 190 180 170 160 150 140 130 120 110 100 90 80 70 60 50 40 30 20 10 0
f1 (ppm)

3p-^{13}C NMR

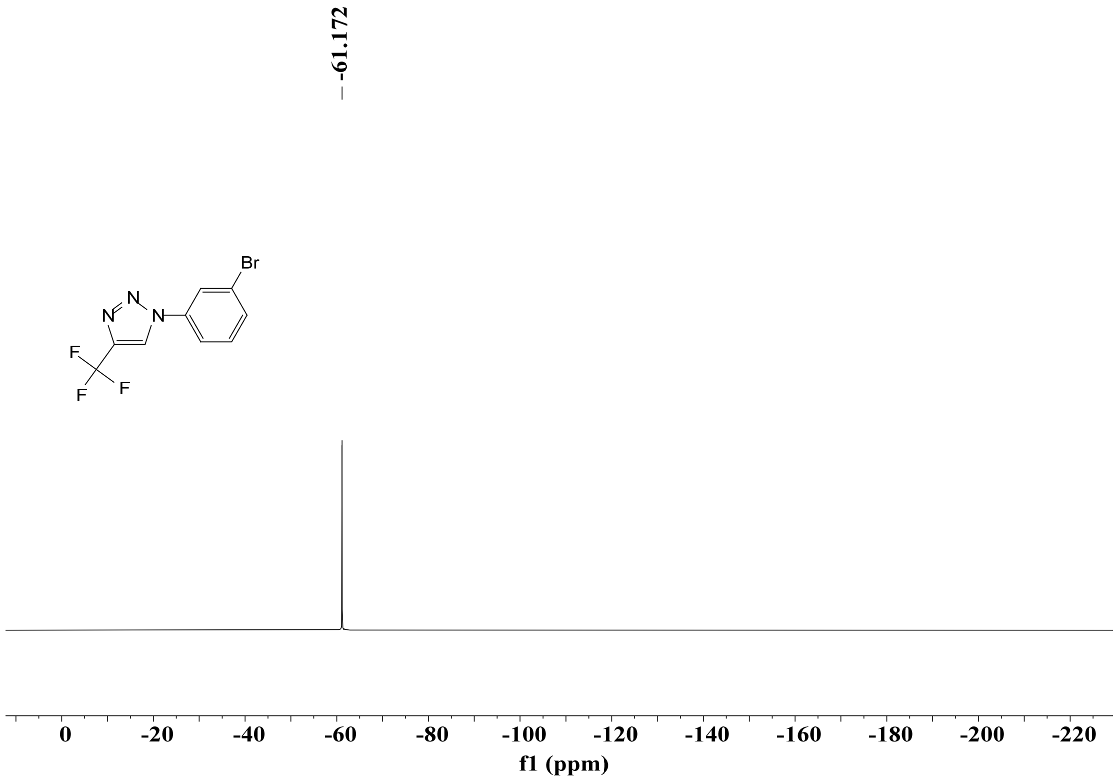

3p-^{19}F NMR

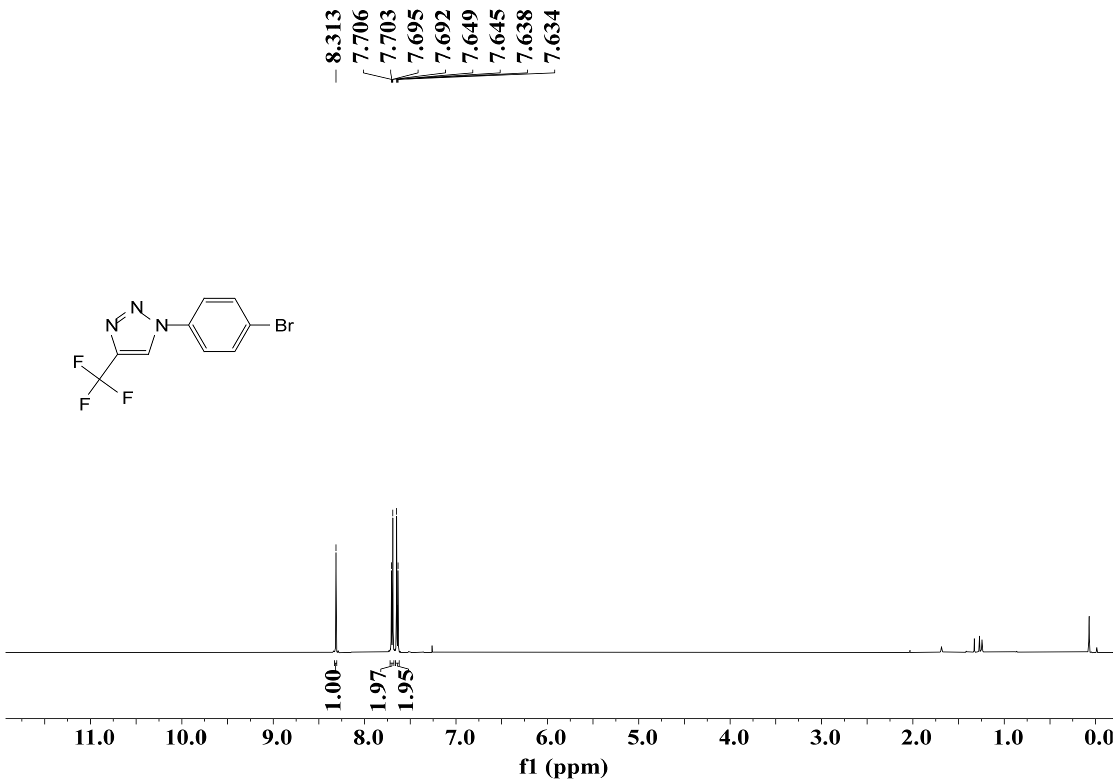

3q-^{1}H NMR

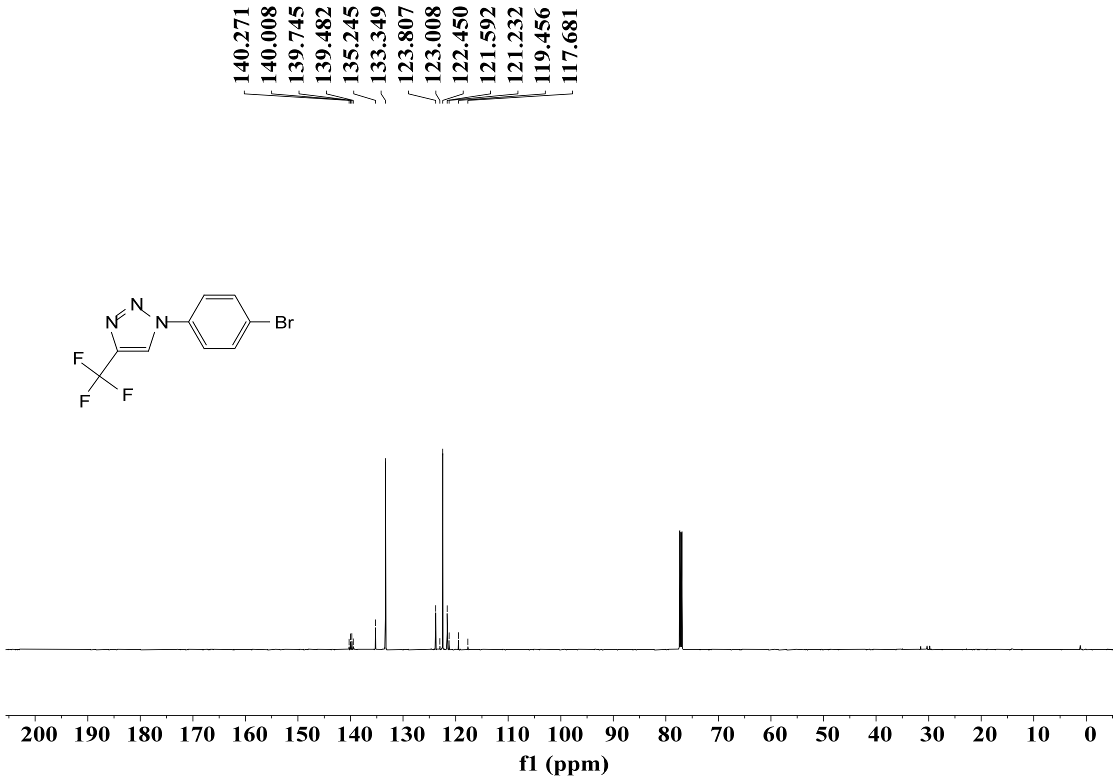
140.271
140.008
139.745
139.482
135.245
133.349
123.807
123.008
122.450
121.592
121.232
119.456
117.681
N
N
N
Br
F
F
F
200 190 180 170 160 150 140 130 120 110 100 90 80 70 60 50 40 30 20 10 0
f1 (ppm)

3q-^{13}C NMR

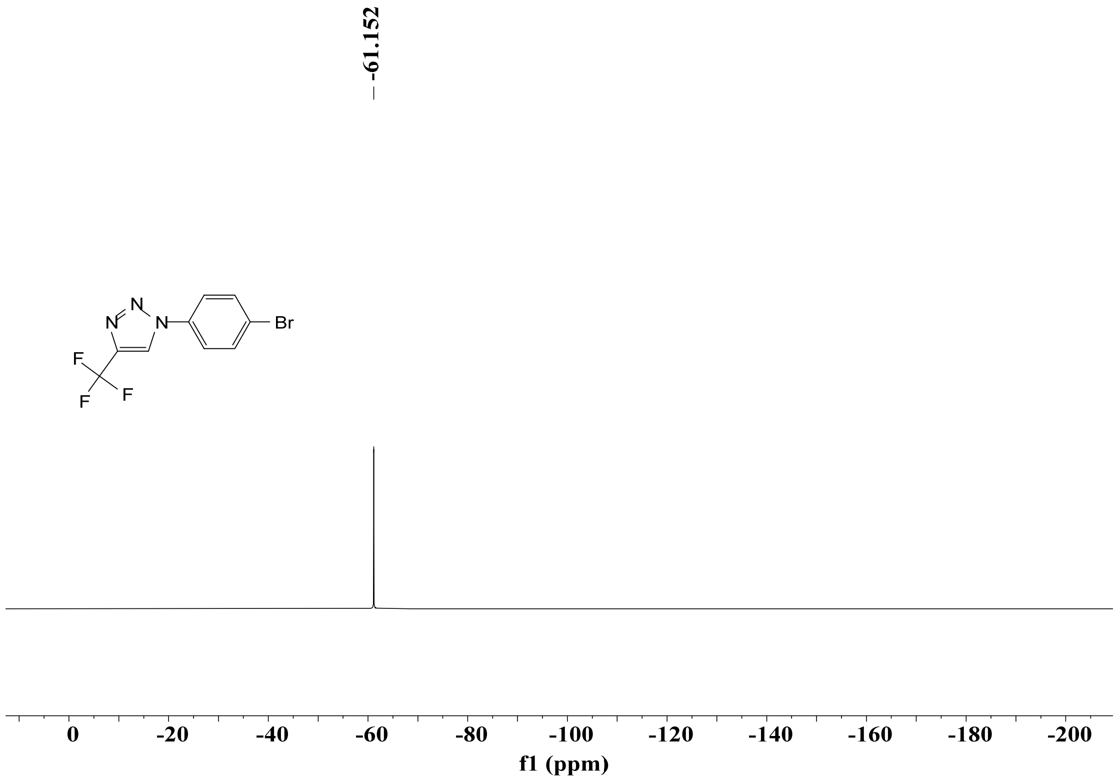

3q-^{19}F NMR

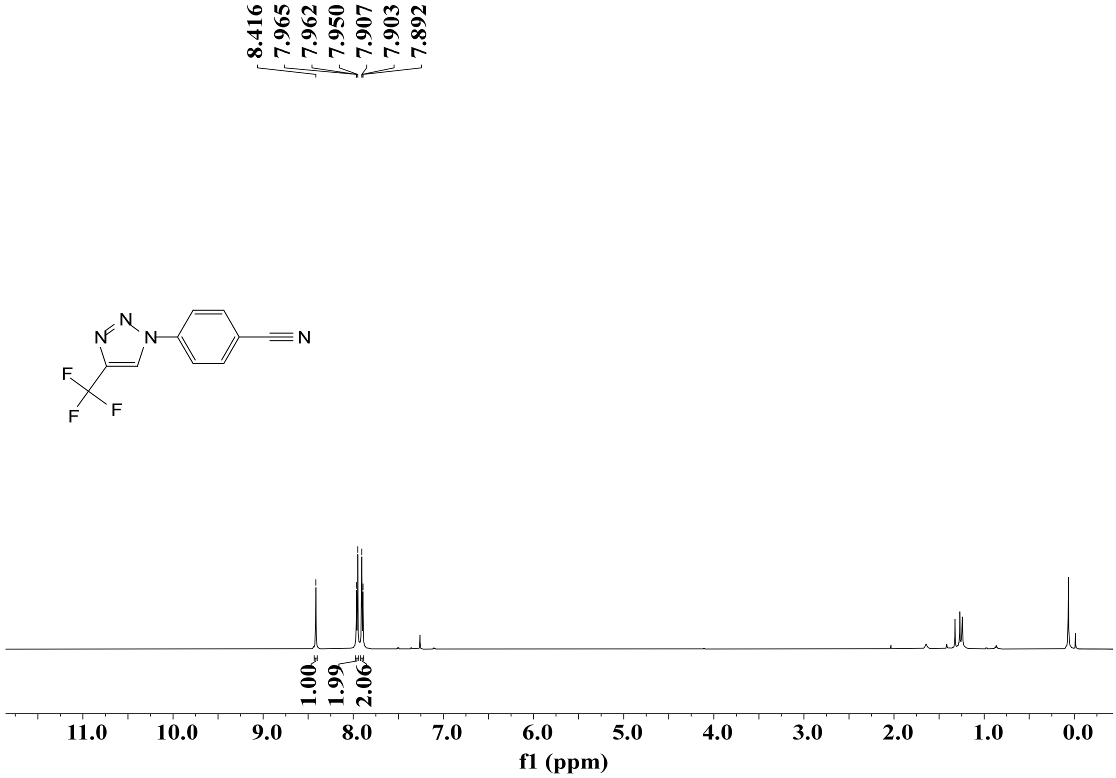

3r-^{1}H NMR

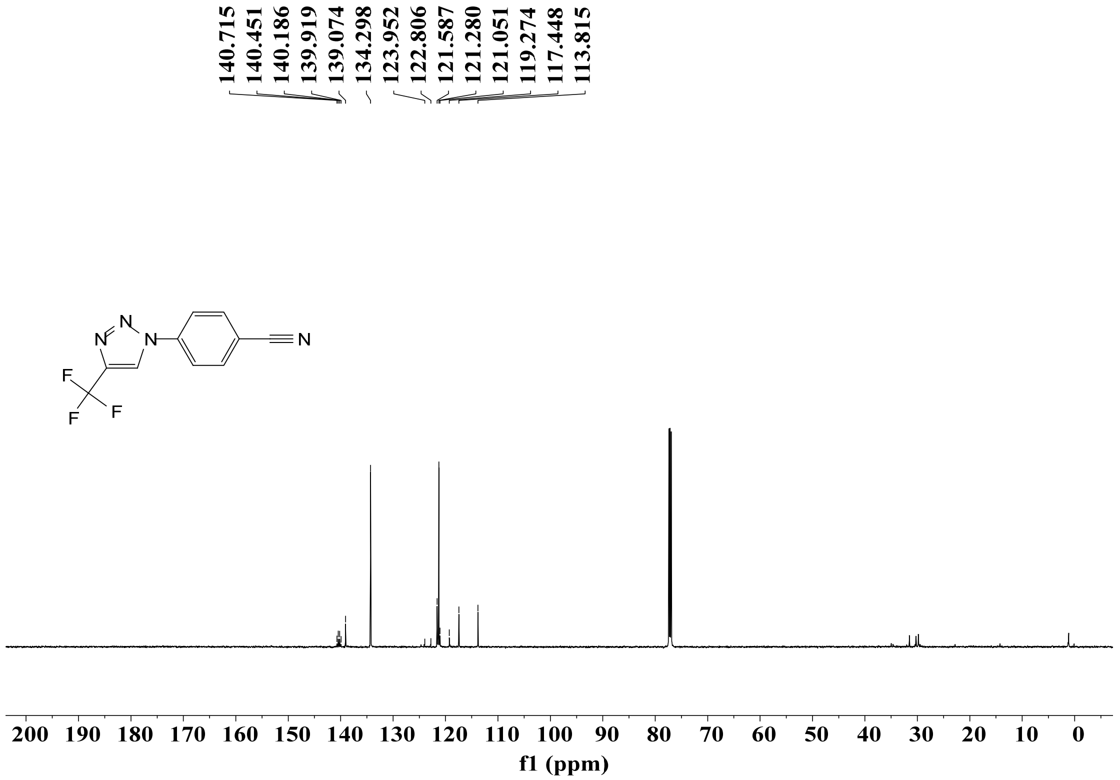

140.715
140.451
140.186
139.919
139.074
134.298
123.952
122.806
121.587
121.280
121.051
119.274
117.448
113.815
N
N
N
N
F
F
F
200 190 180 170 160 150 140 130 120 110 100 90 80 70 60 50 40 30 20 10 0
f1 (ppm)

3r-^{13}C NMR

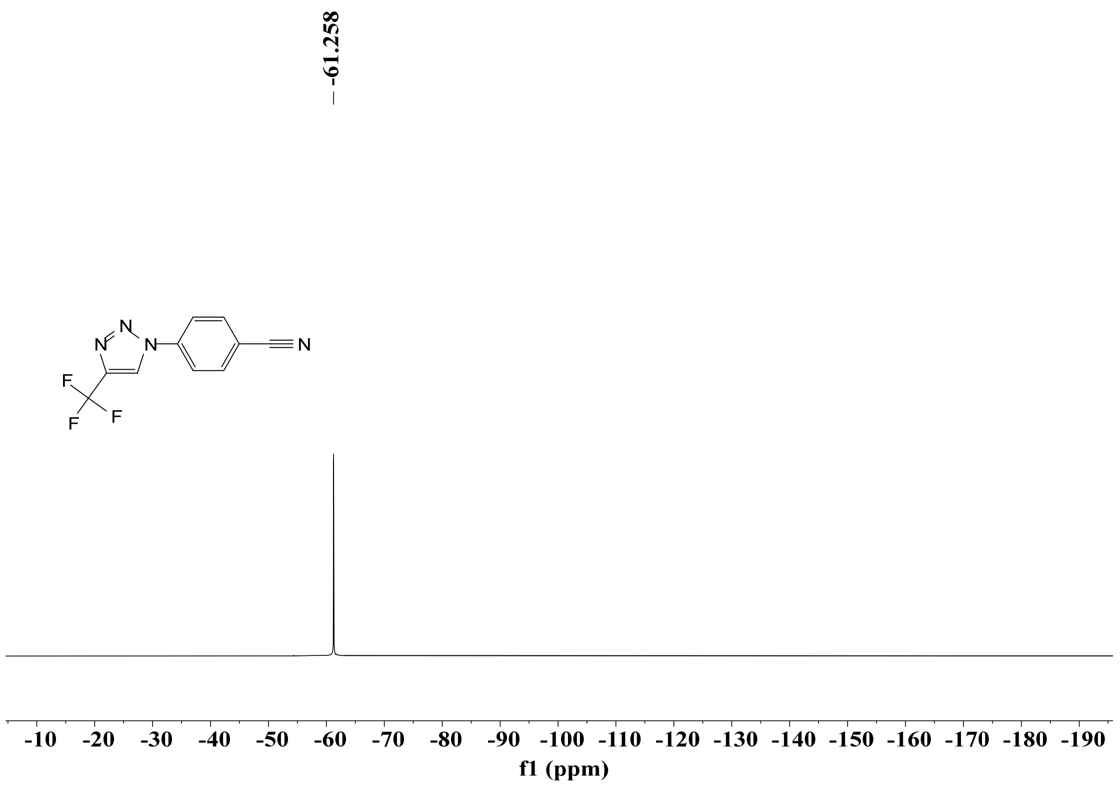

3r-^{19}F NMR

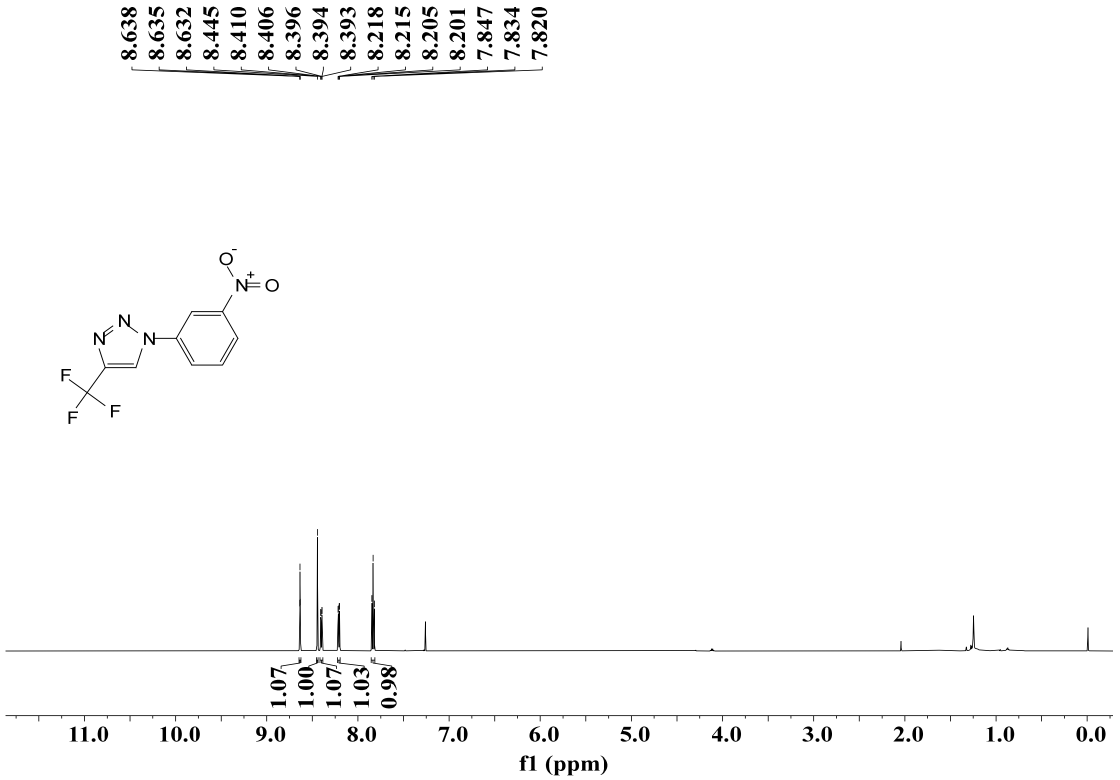

3s-^{1}H NMR

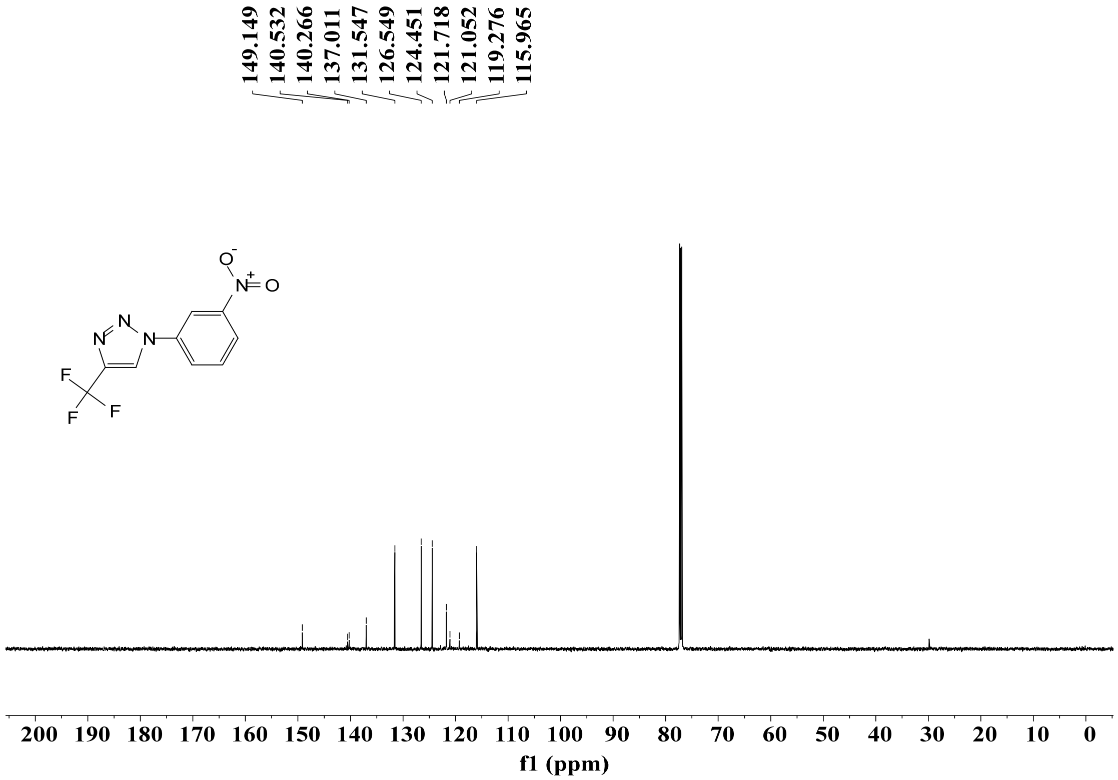
149.149
140.532
140.266
137.011
131.547
126.549
124.451
121.718
121.052
119.276
115.965
200 190 180 170 160 150 140 130 120 110 100 90 80 70 60 50 40 30 20 10 0
f1 (ppm)

3s-^{13}C NMR

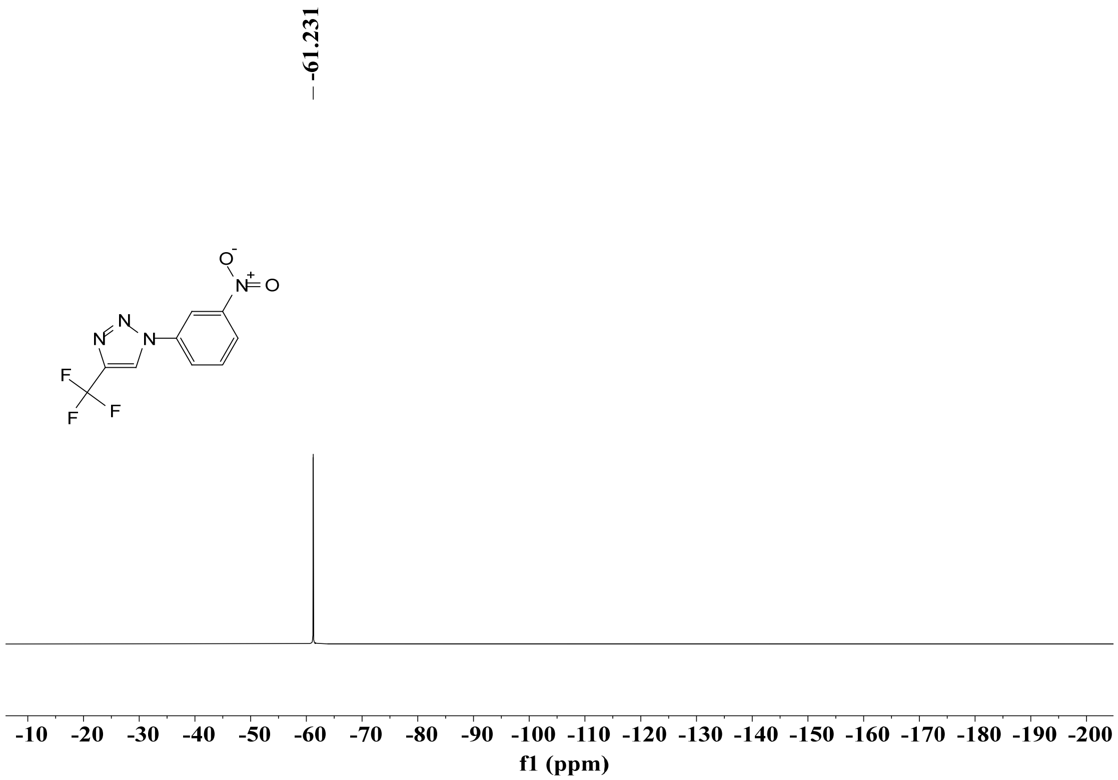

3s-^{19}F NMR

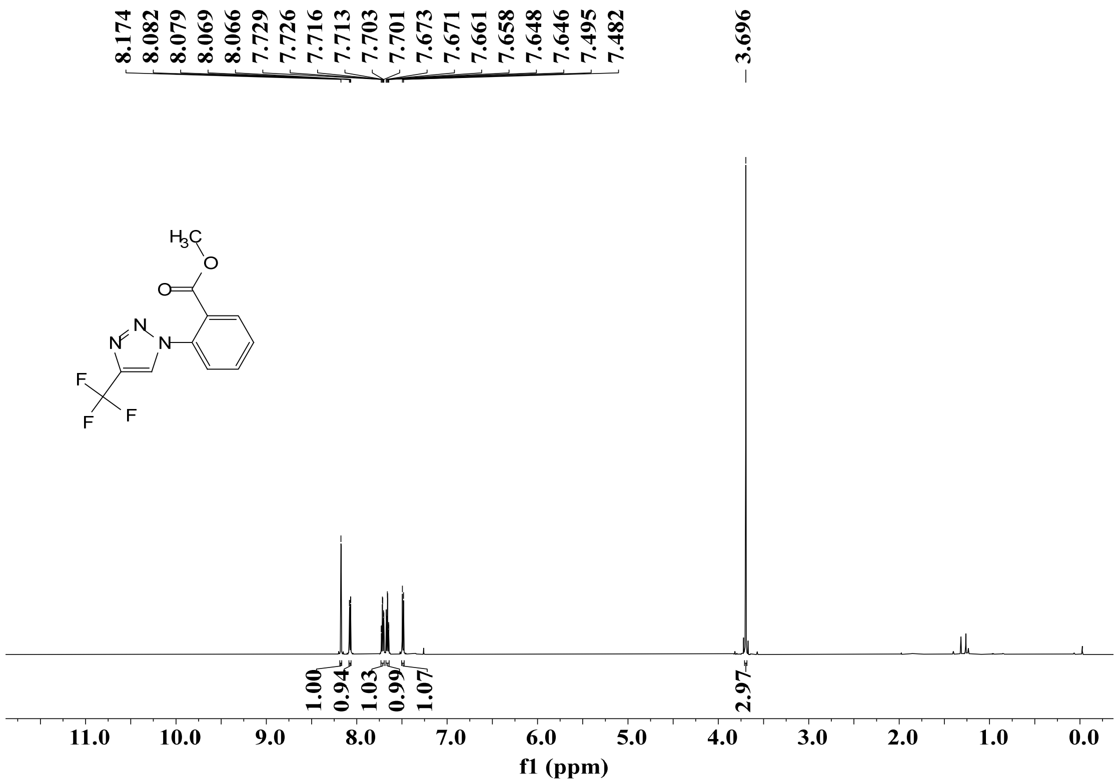

3t-^{1}H NMR

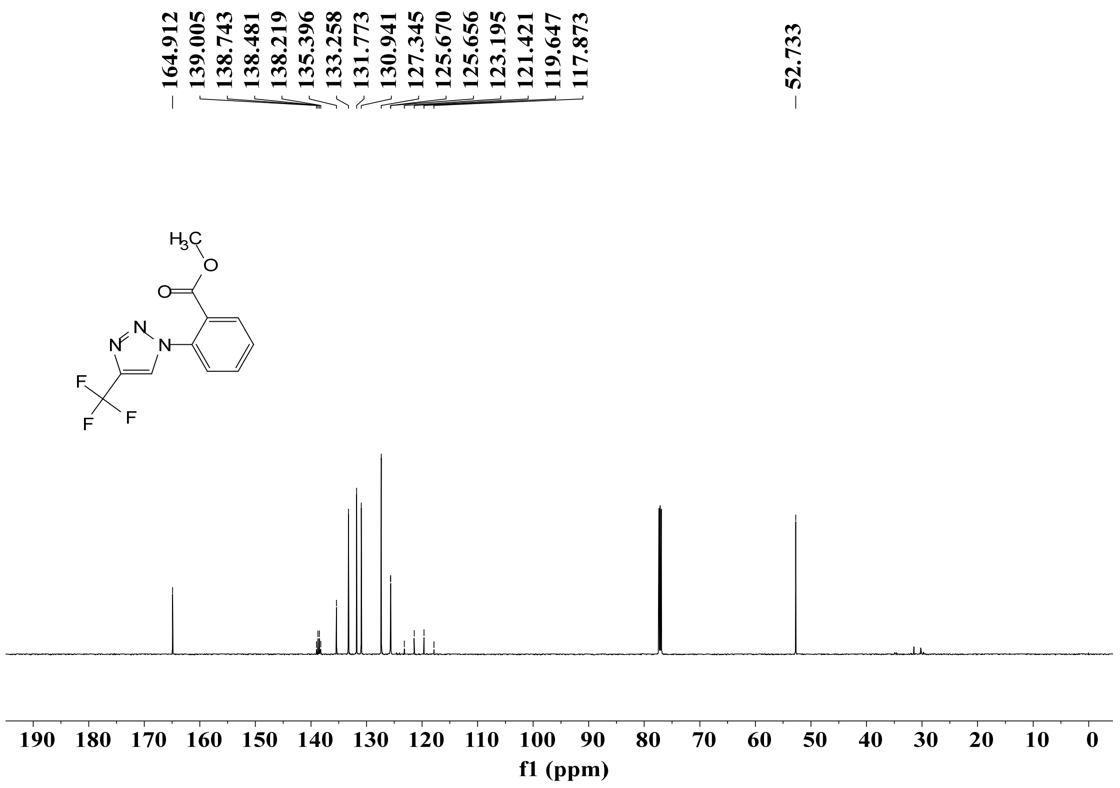
164.912
139.005
138.743
138.481
138.219
135.396
133.258
131.773
130.941
127.345
125.670
125.656
123.195
121.421
119.647
117.873
52.733
H3C
O
O
N
N
N
F
F
F
190 180 170 160 150 140 130 120 110 100 90 80 70 60 50 40 30 20 10 0
f1 (ppm)

3t-^{13}C NMR

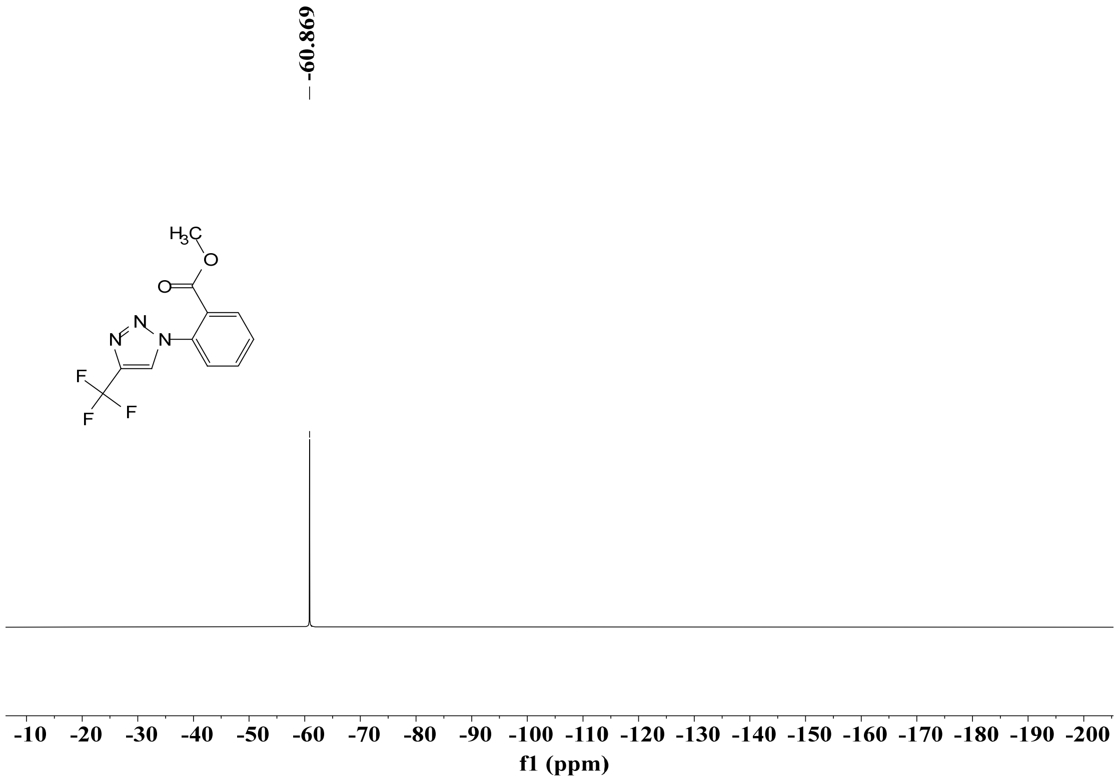

3t-^{19}F NMR

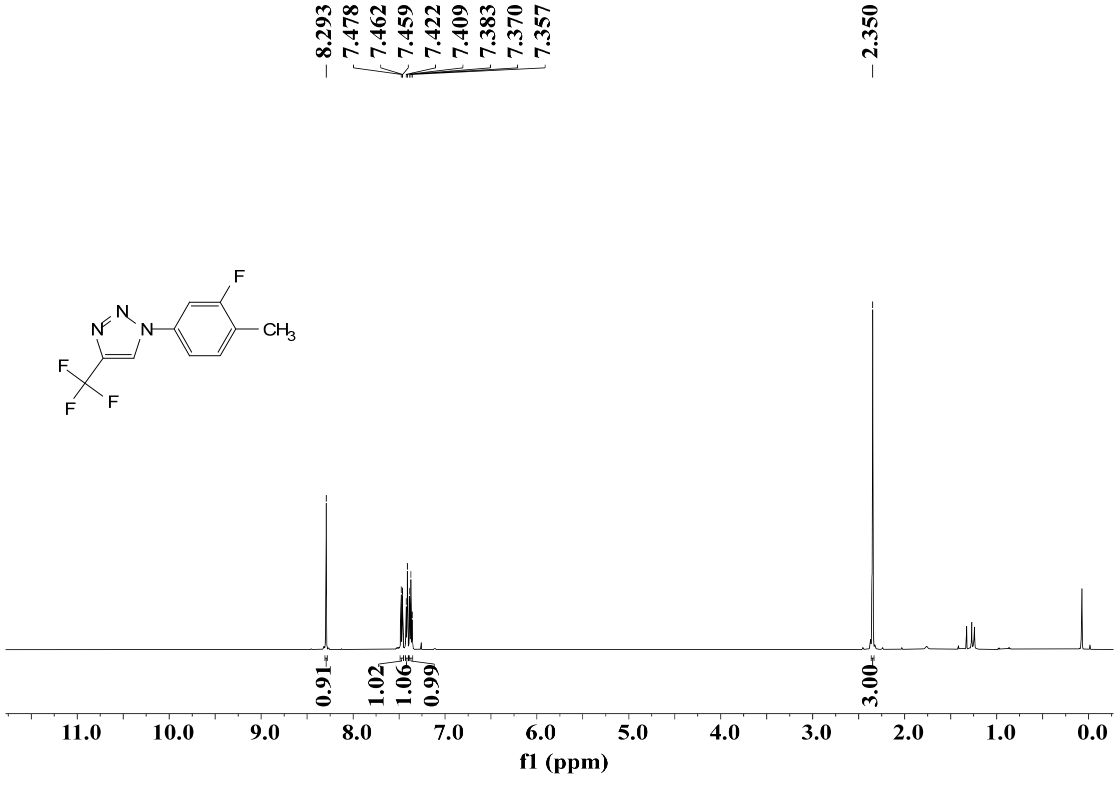

3u-^{1}H NMR

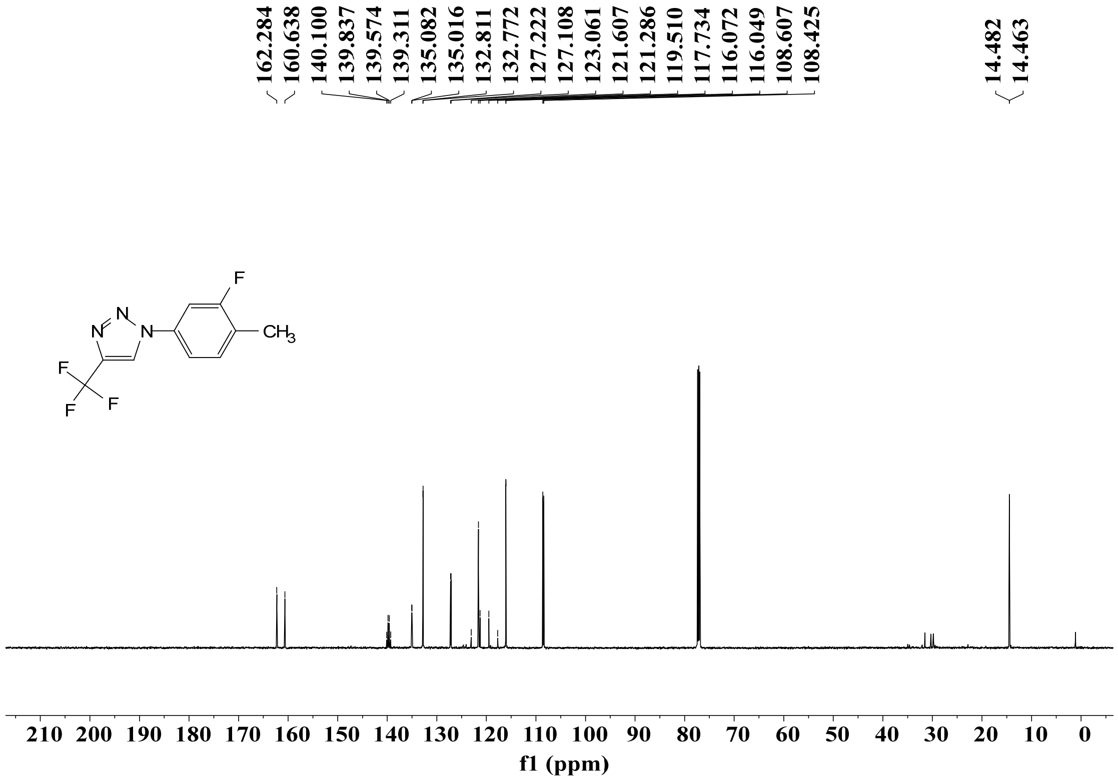
162.284
160.638
140.100
139.837
139.574
139.311
135.082
135.016
132.811
132.772
127.222
127.108
123.061
121.607
121.286
119.510
117.734
116.072
116.049
108.607
108.425
14.482
14.463
F
N
N
N
CH3
F
F
F
210 200 190 180 170 160 150 140 130 120 110 100 90 80 70 60 50 40 30 20 10 0
f1 (ppm)

3u-^{13}C NMR

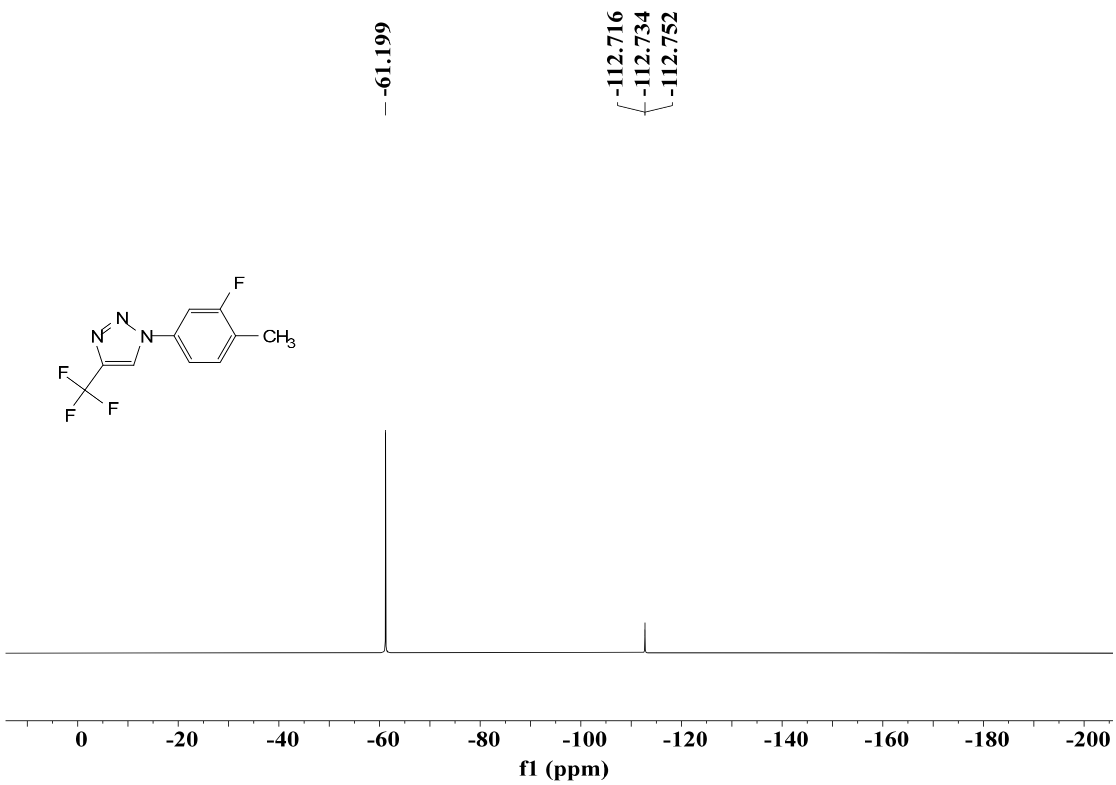

3u-^{19}F NMR

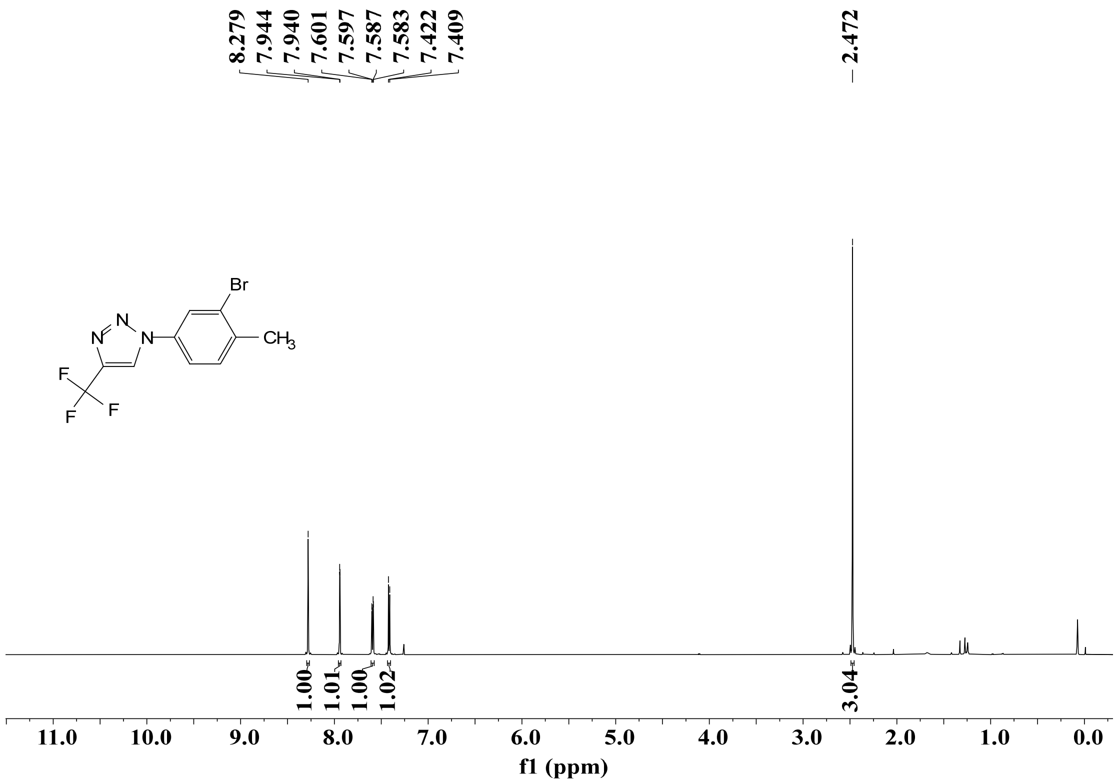

3v-^{1}H NMR

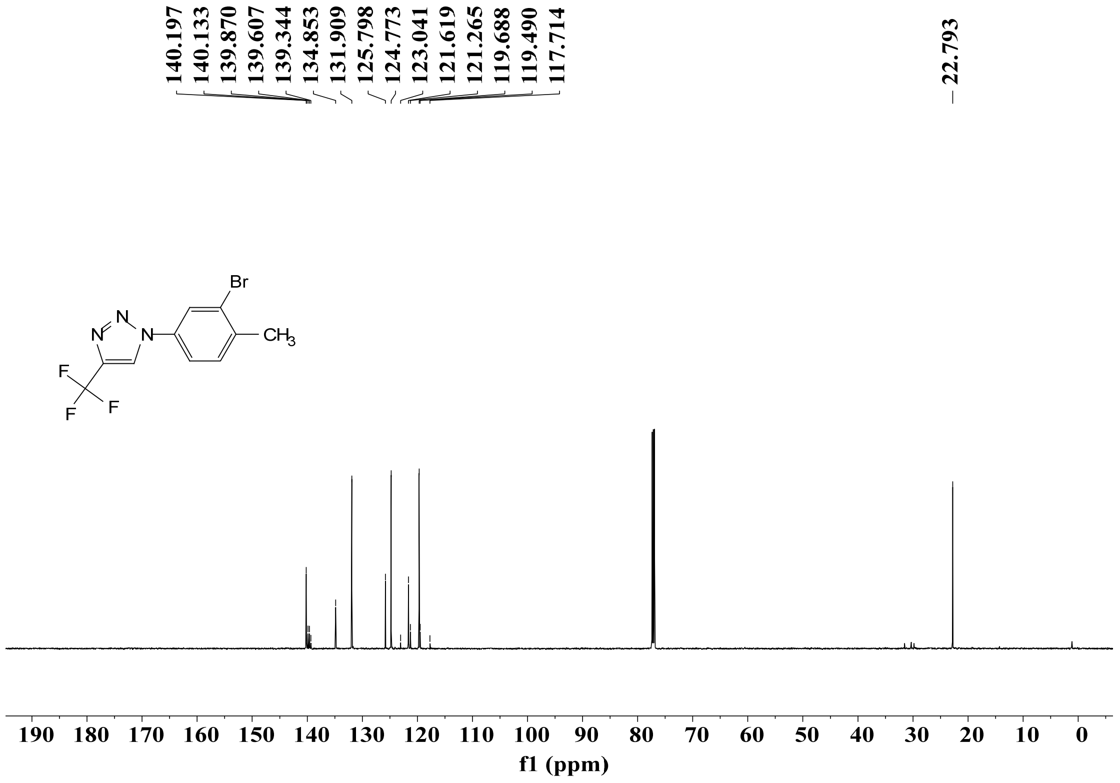
140.197
140.133
139.870
139.607
139.344
134.853
131.909
125.798
124.773
123.041
121.619
121.265
119.688
119.490
117.714
22.793
Br
N
N
N
CH3
F
F
F
190 180 170 160 150 140 130 120 110 100 90 80 70 60 50 40 30 20 10 0
f1 (ppm)

3v-^{13}C NMR

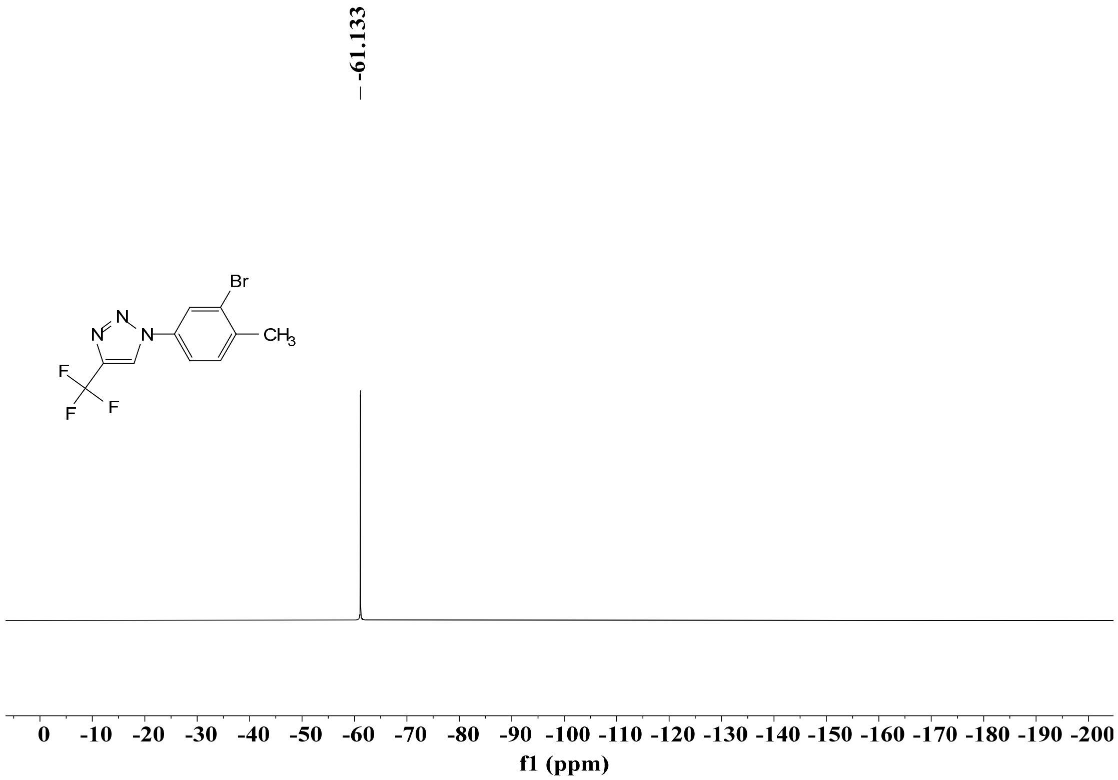

3v-^{19}F NMR

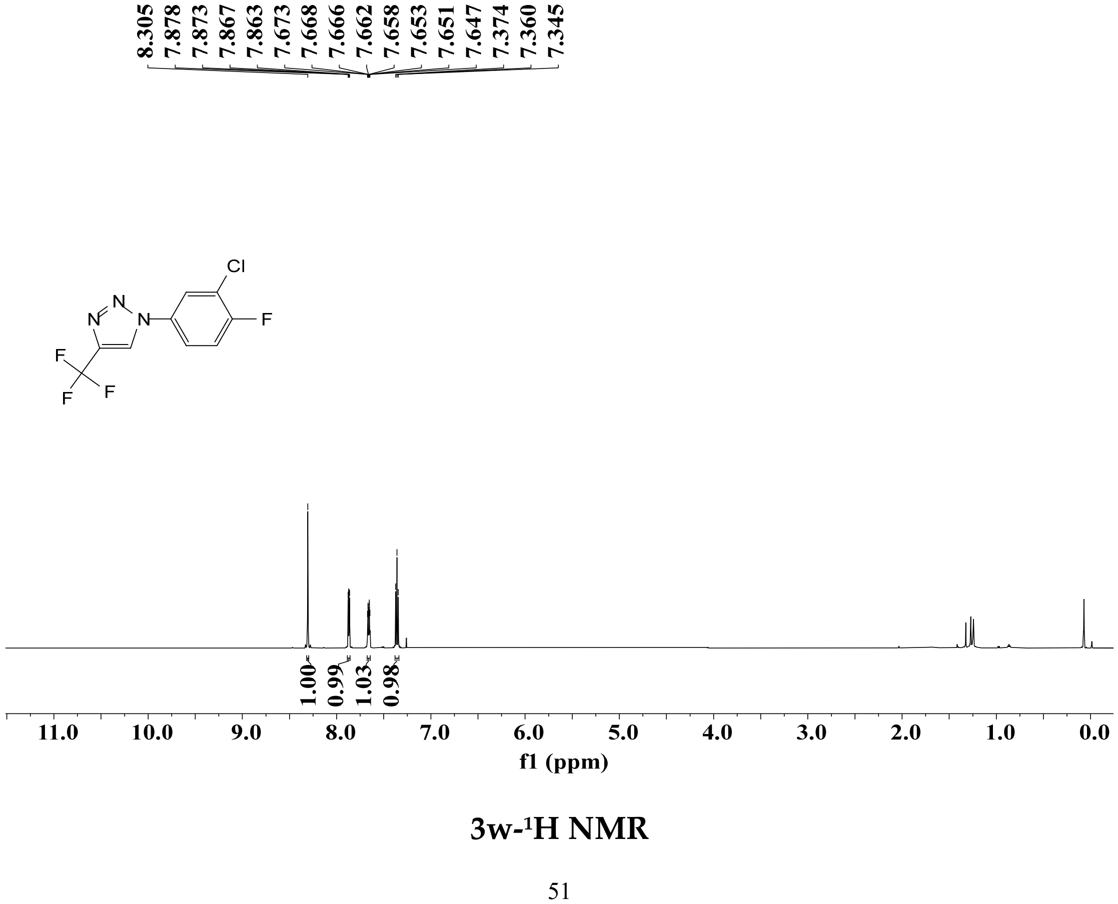

3w-^{1}H NMR

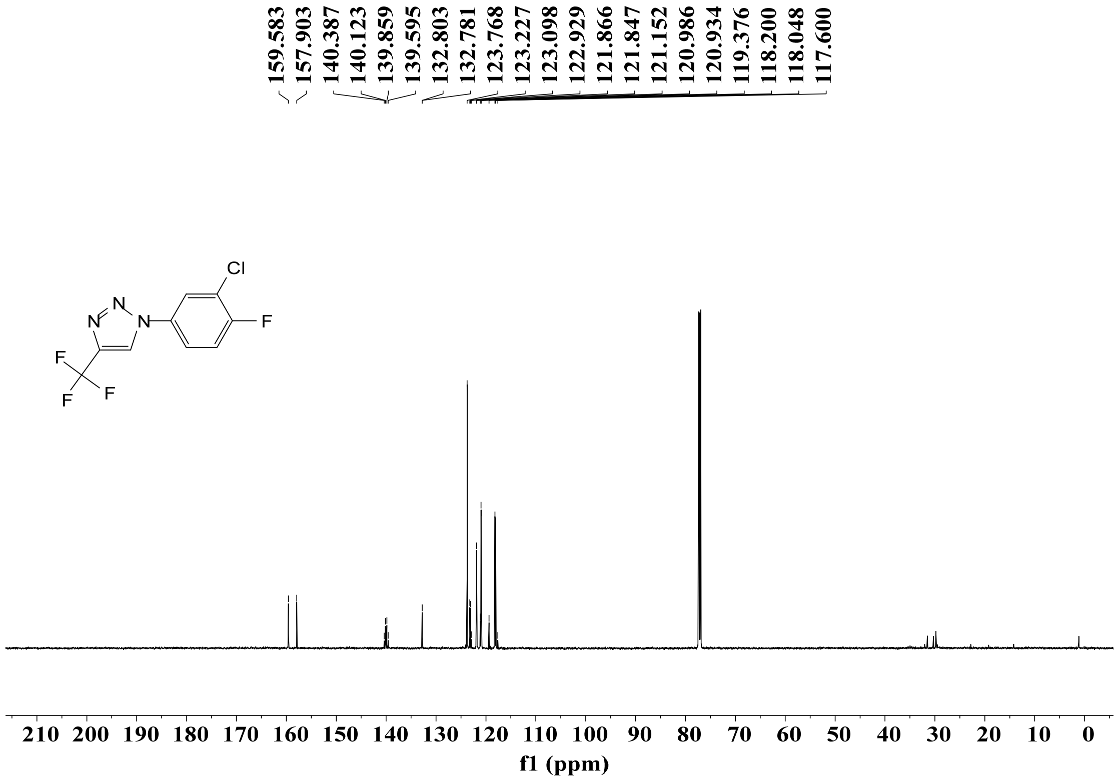
159.583
157.903
140.387
140.123
139.859
139.595
132.803
132.781
123.768
123.227
123.098
122.929
121.866
121.847
121.152
120.986
120.934
119.376
118.200
118.048
117.600
Cl
N
N
N
F
F
F
F
210 200 190 180 170 160 150 140 130 120 110 100 90 80 70 60 50 40 30 20 10 0
f1 (ppm)

3w-^{13}C NMR

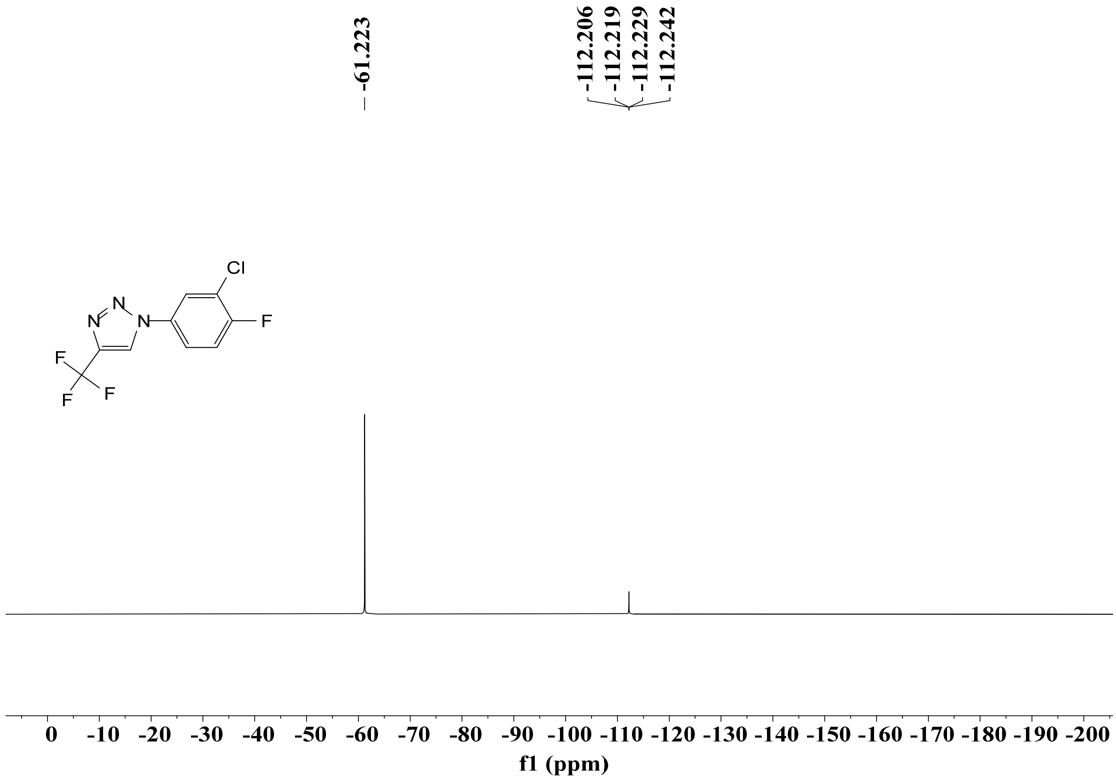

3w-^{19}F NMR

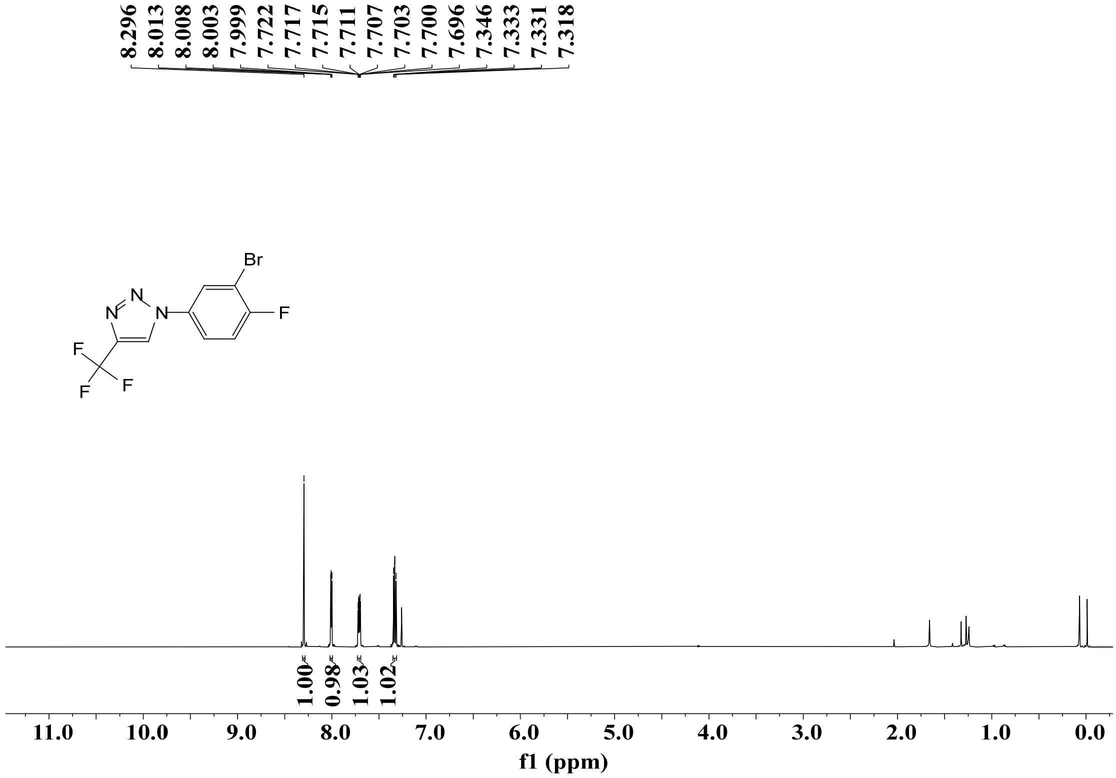

3x-^{1}H NMR

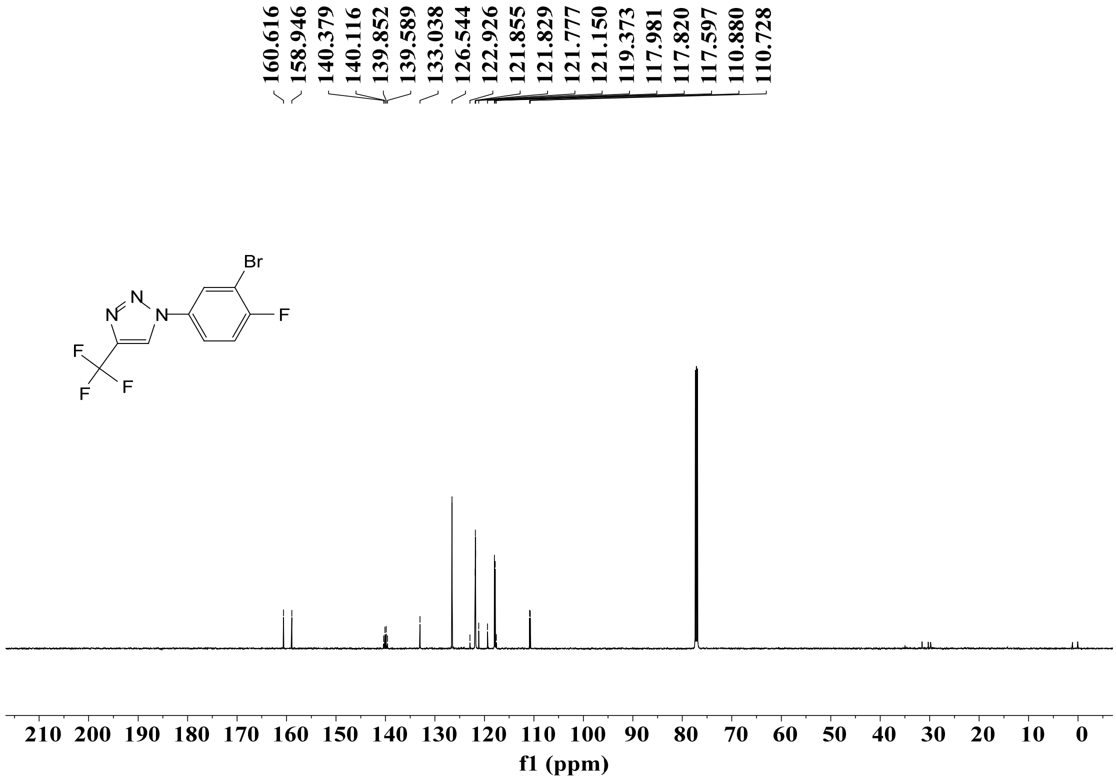
160.616
158.946
140.379
140.116
139.852
139.589
133.038
126.544
122.926
121.855
121.829
121.777
121.150
119.373
117.981
117.820
117.597
110.880
110.728
Br
N
N
N
F
F
F
F
210 200 190 180 170 160 150 140 130 120 110 100 90 80 70 60 50 40 30 20 10 0
f1 (ppm)

3x-¹³C NMR

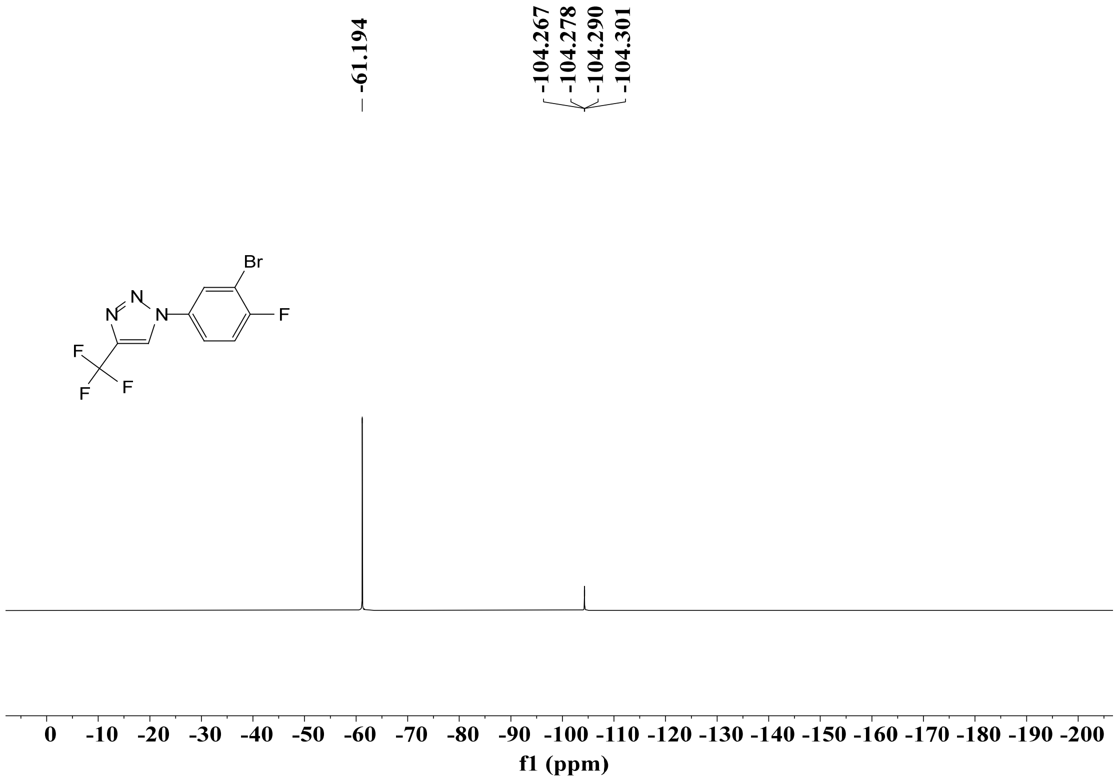

3x-^{19}F NMR

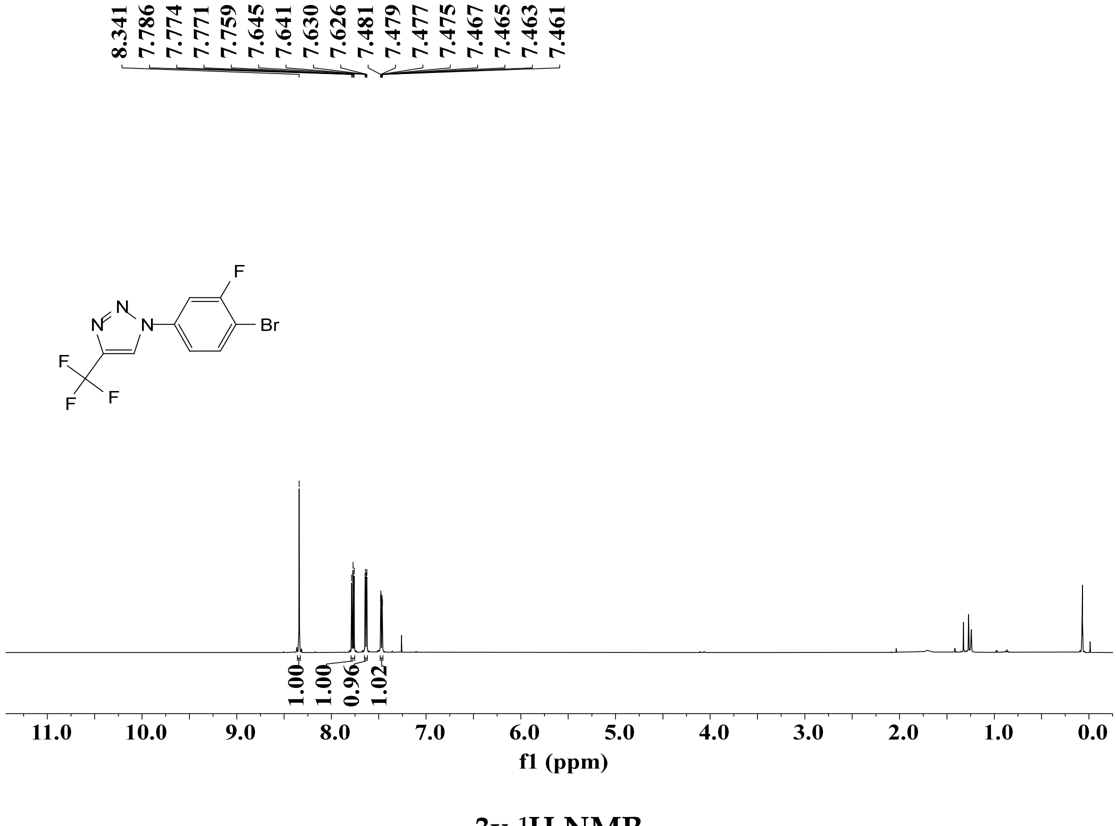

3y-^{1}H NMR

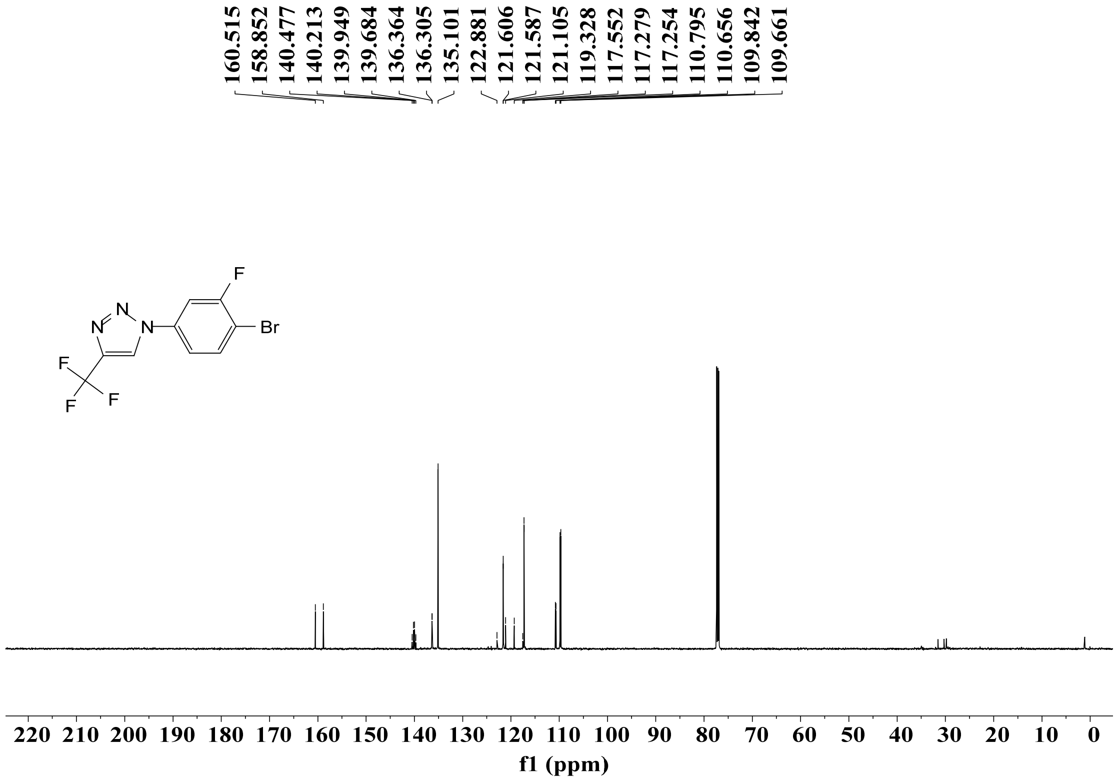
160.515
158.852
140.477
140.213
139.949
139.684
136.364
136.305
135.101
122.881
121.606
121.587
121.105
119.328
117.552
117.279
117.254
110.795
110.656
109.842
109.661
F
N
N
N
Br
F
F
F
220 210 200 190 180 170 160 150 140 130 120 110 100 90 80 70 60 50 40 30 20 10 0
f1 (ppm)

3y-^{13}C NMR

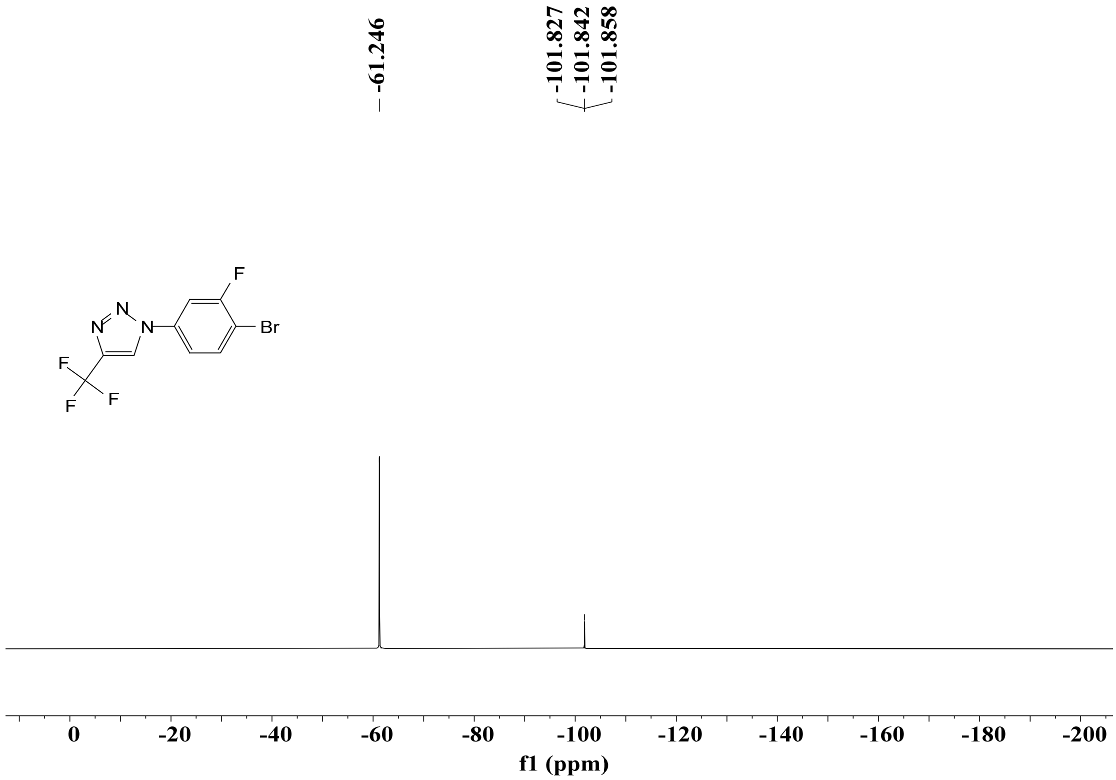

3y-^{19}F NMR

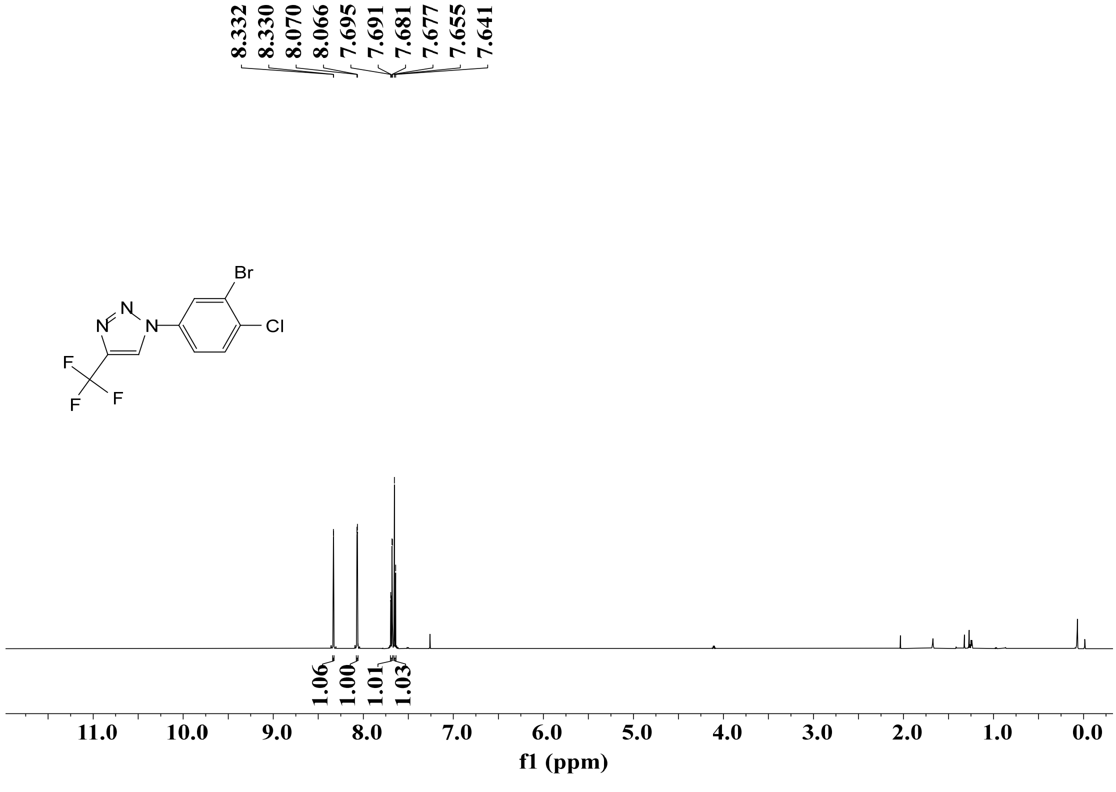

3z-^{1}H NMR

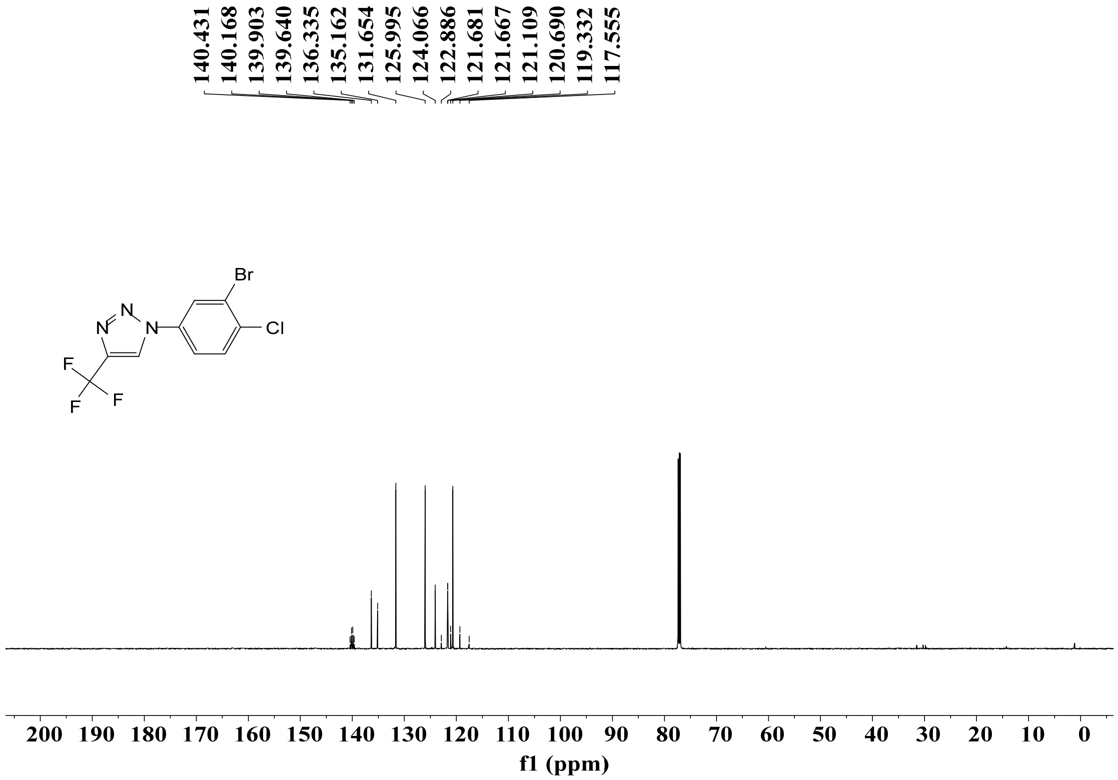
140.431
140.168
139.903
139.640
136.335
135.162
131.654
125.995
124.066
122.886
121.681
121.667
121.109
120.690
119.332
117.555
Br
Cl
N
N
N
F
F
F
200 190 180 170 160 150 140 130 120 110 100 90 80 70 60 50 40 30 20 10 0
f1 (ppm)

3z-^{13}C NMR

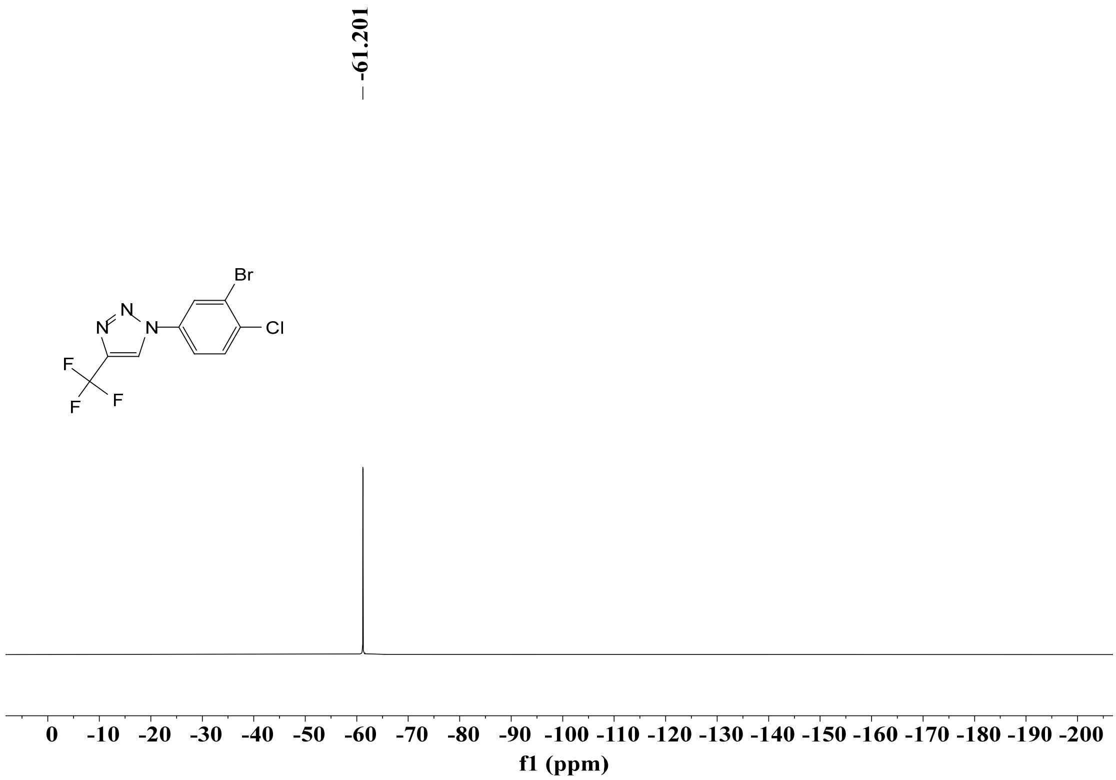

3z-^{19}F NMR

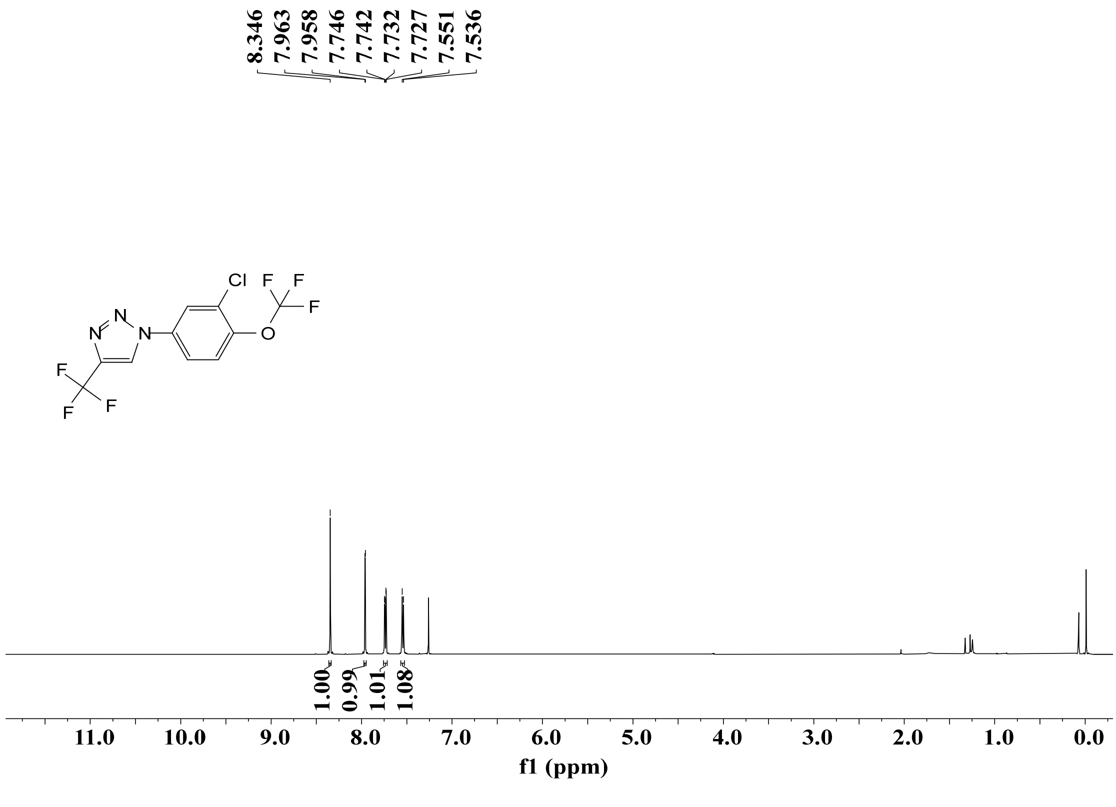

3aa-^{1}H NMR

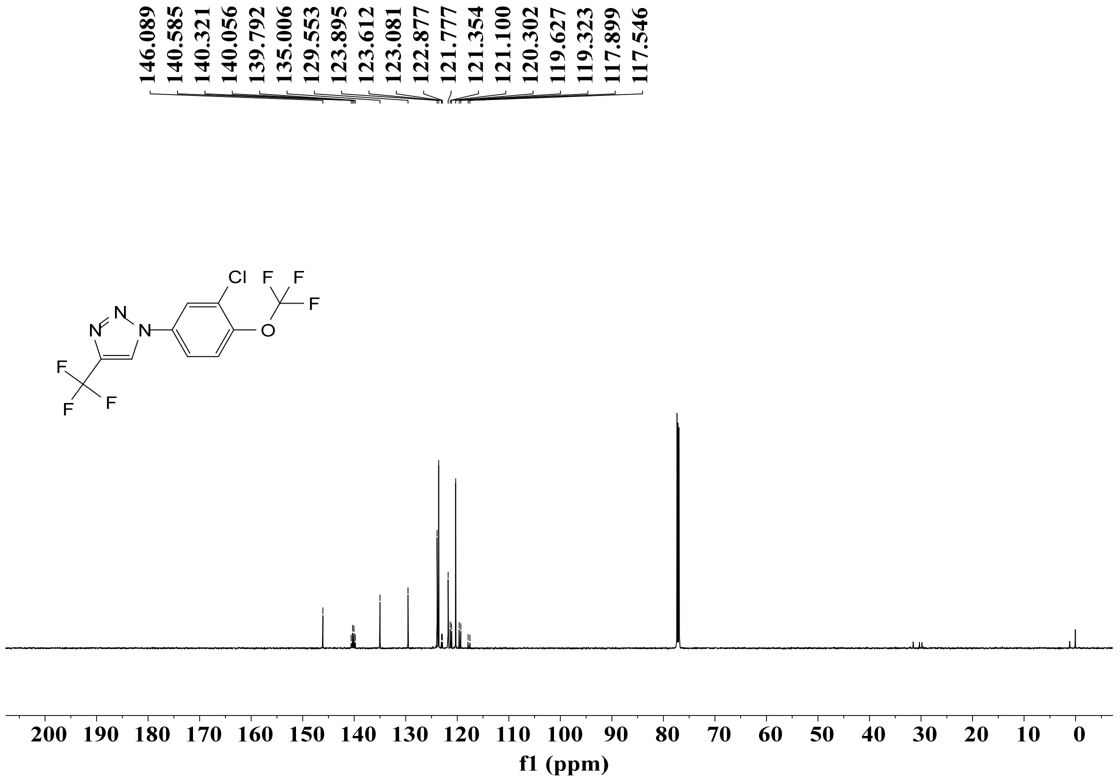
146.089
140.585
140.321
140.056
139.792
135.006
129.553
123.895
123.612
123.081
122.877
121.777
121.354
121.100
120.302
119.627
119.323
117.899
117.546
Cl
F
F
F
N
N
N
O
F
F
F
200 190 180 170 160 150 140 130 120 110 100 90 80 70 60 50 40 30 20 10 0
f1 (ppm)

3aa-^{13}C NMR

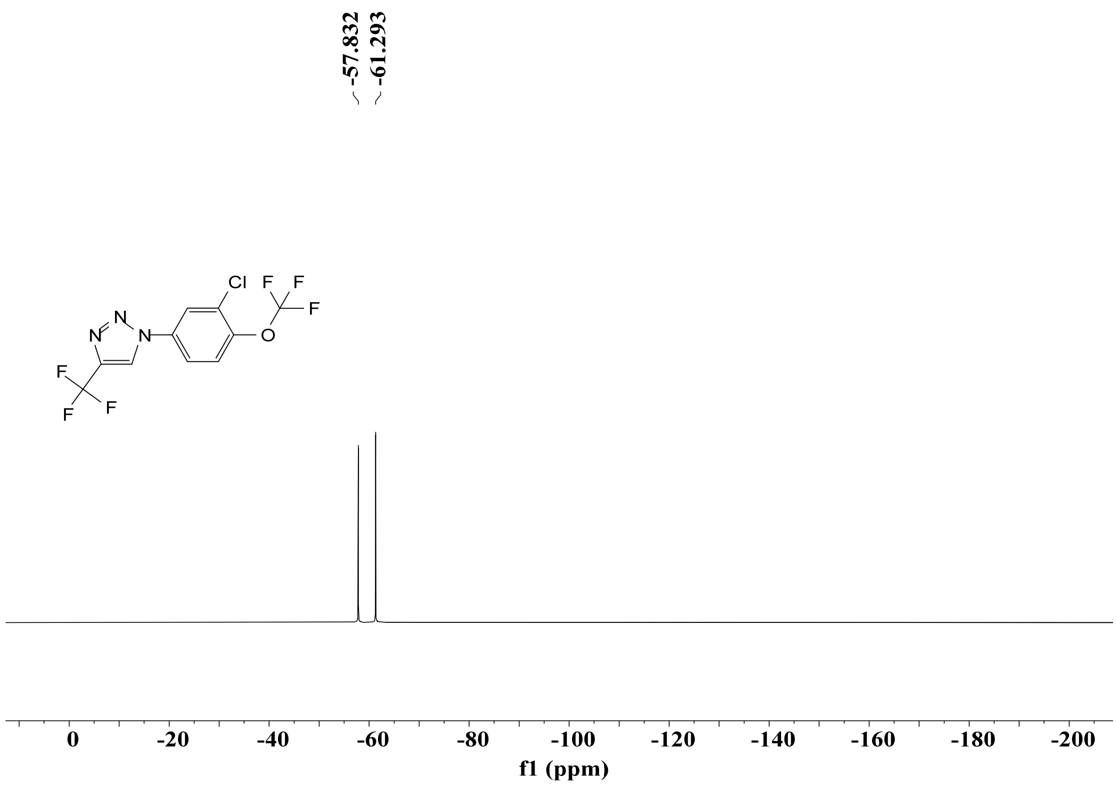

3aa-^{19}F NMR

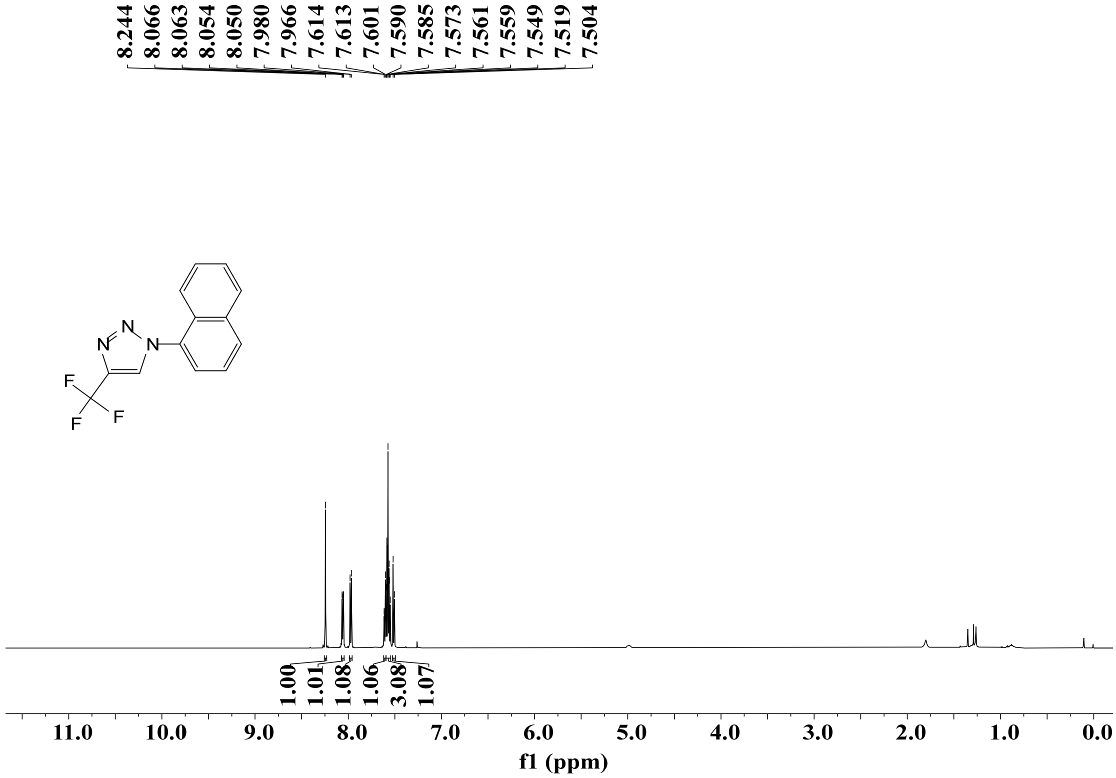

3ab-^{1}H NMR

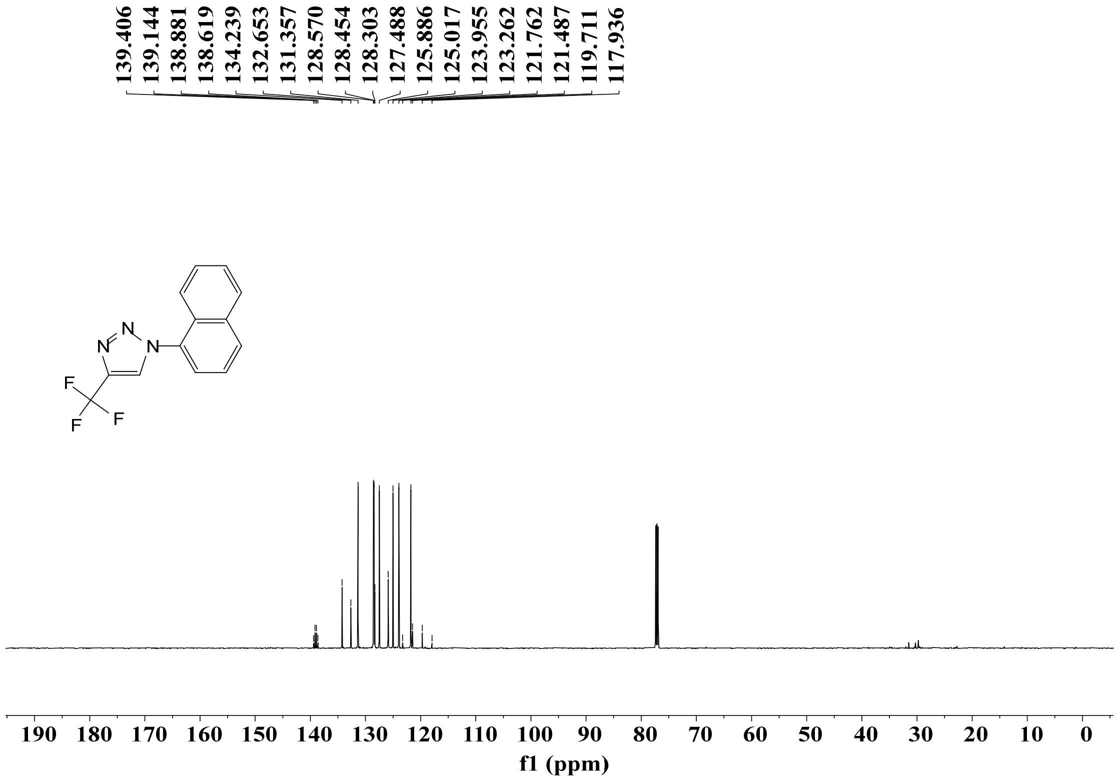
139.406
139.144
138.881
138.619
134.239
132.653
131.357
128.570
128.454
128.303
127.488
125.886
125.017
123.955
123.262
121.762
121.487
119.711
117.936
190 180 170 160 150 140 130 120 110 100 90 80 70 60 50 40 30 20 10 0
f1 (ppm)
N
N
N
F
F
F

3ab-^{13}C NMR

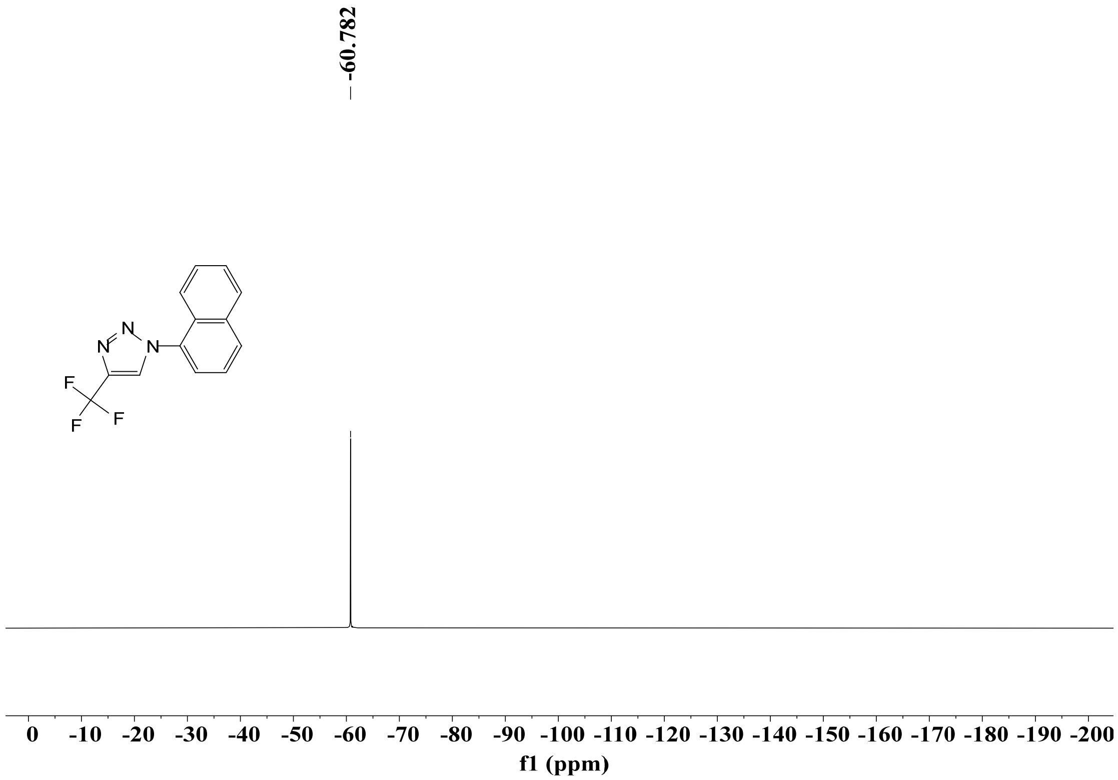
-60.782
N
N
N
F
F
F
0
-10
-20
-30
-40
-50
-60
-70
-80
-90
-100
-110
-120
-130
-140
-150
-160
-170
-180
-190
-200
f1 (ppm)

3ab-^{19}F NMR

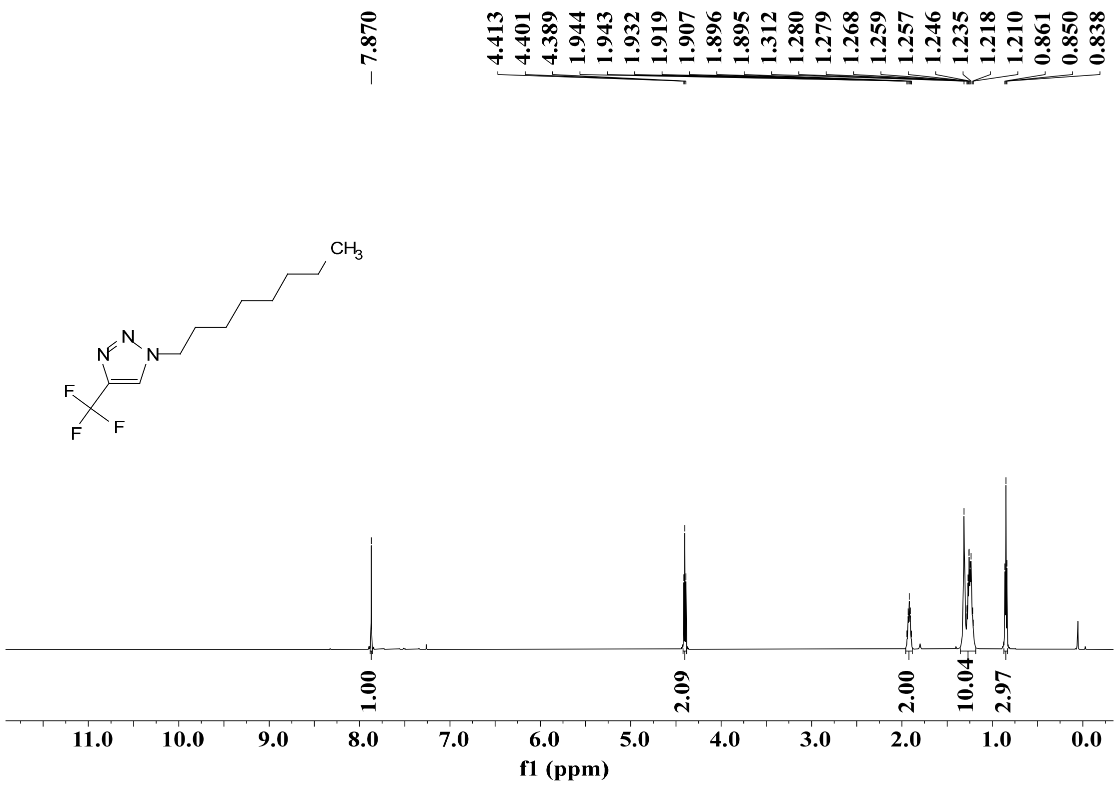

4a-¹H NMR

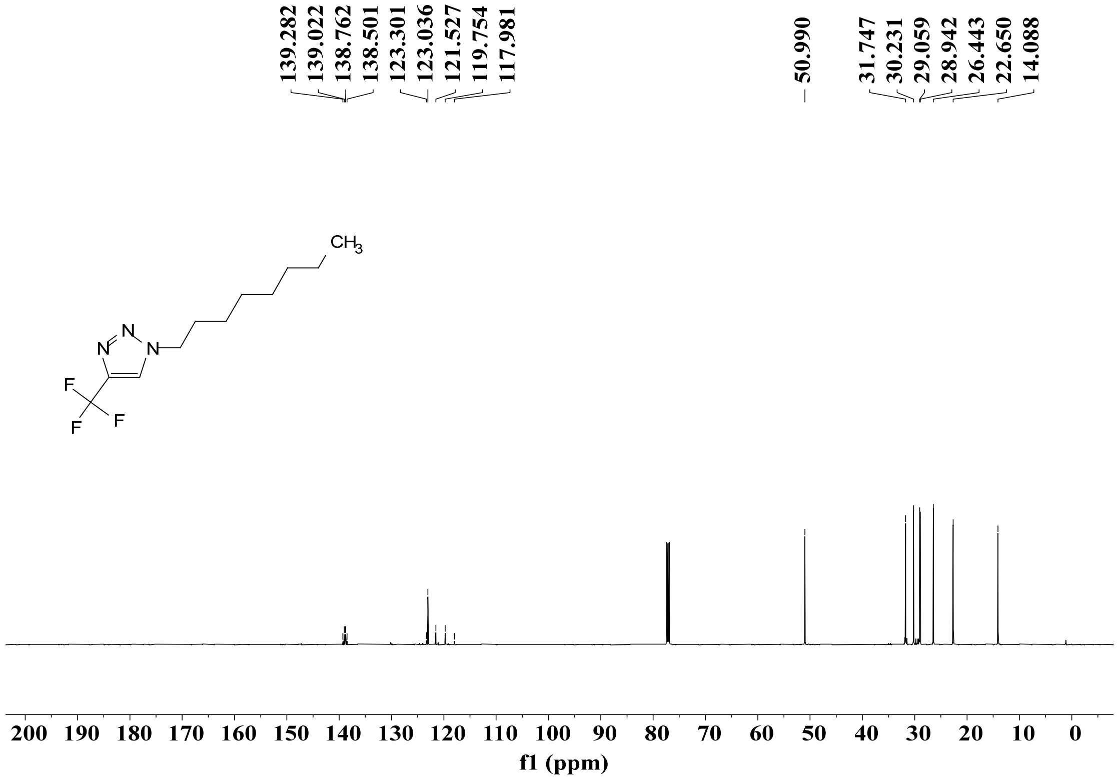

4a-¹³C NMR

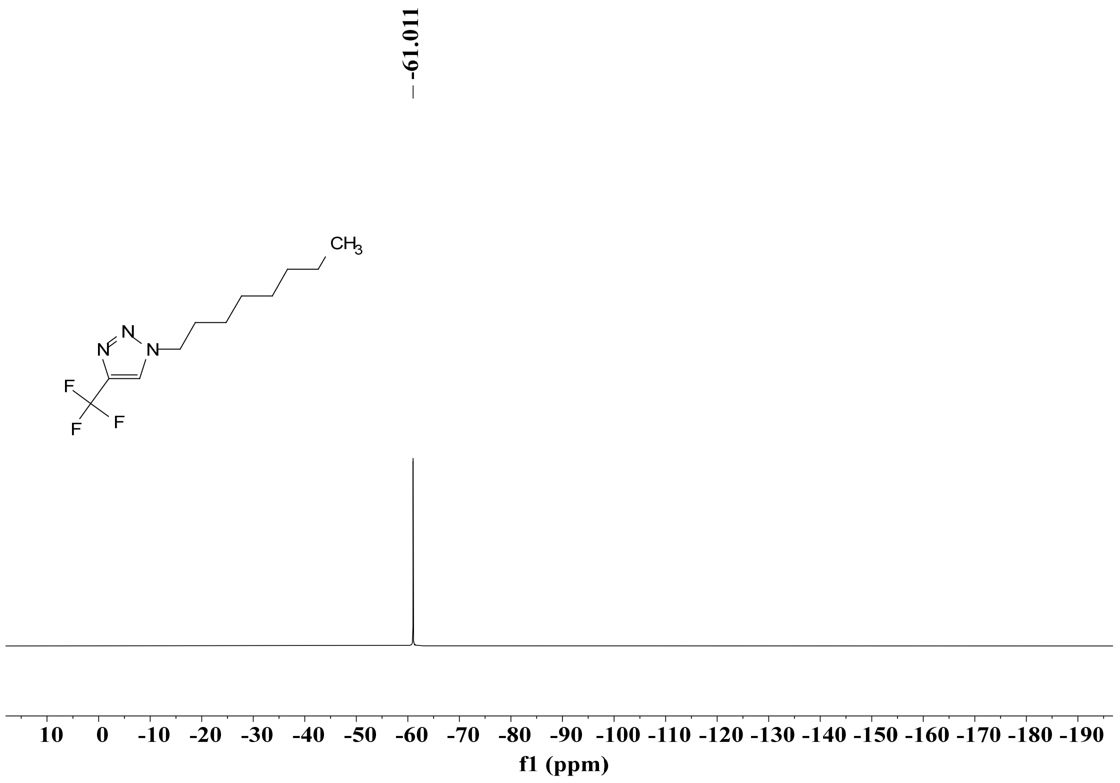
-61.011
CH3
N
N
N
F
F
F
10
0
-10
-20
-30
-40
-50
-60
-70
-80
-90
-100
-110
-120
-130
-140
-150
-160
-170
-180
-190
f1 (ppm)

4a-^{19}F NMR

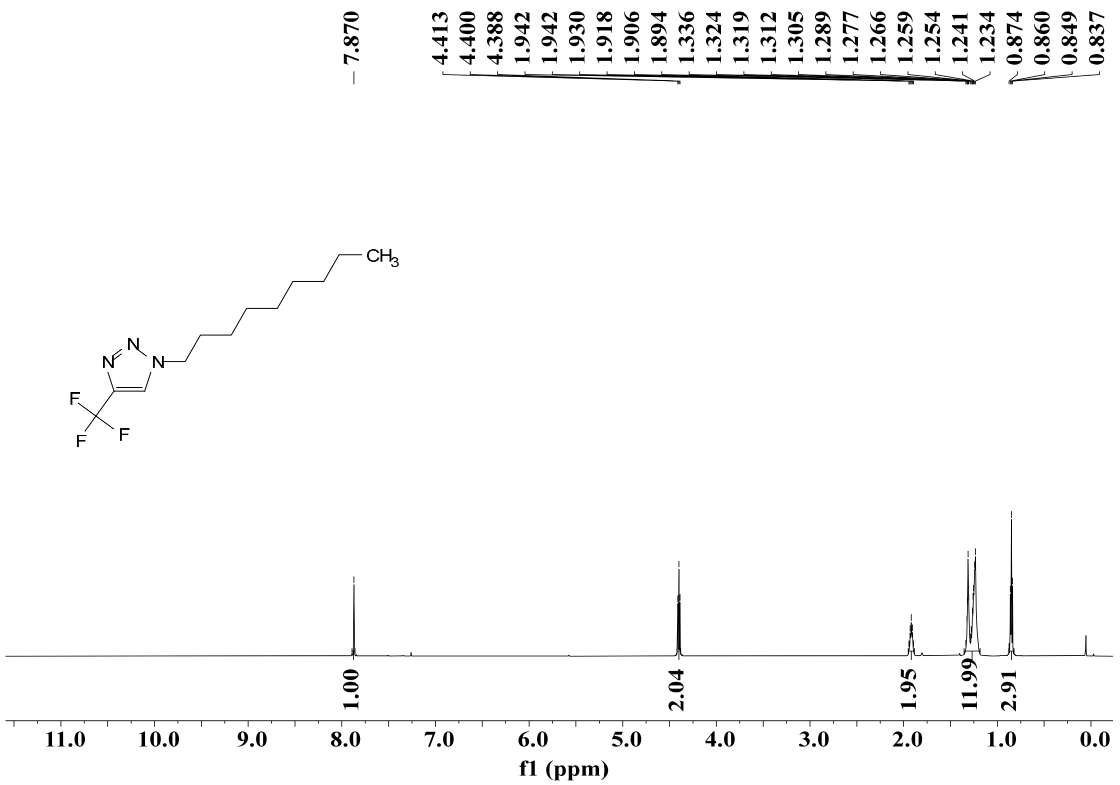

4b-¹H NMR

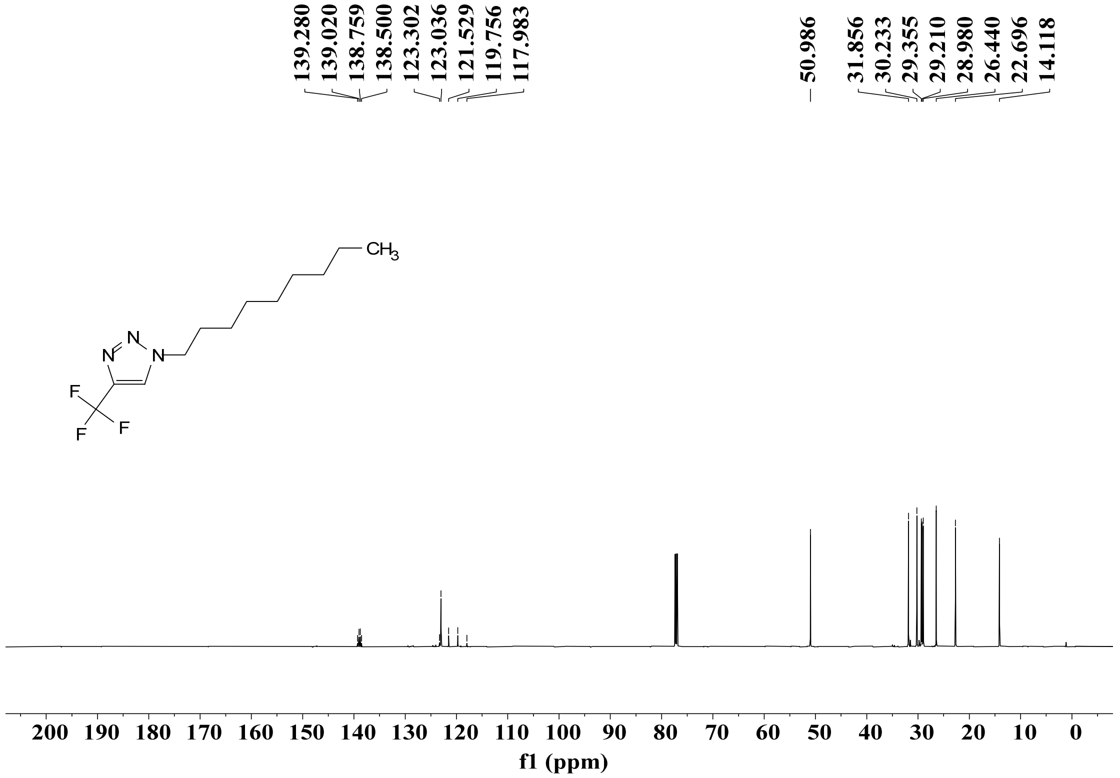

4b-¹³C NMR

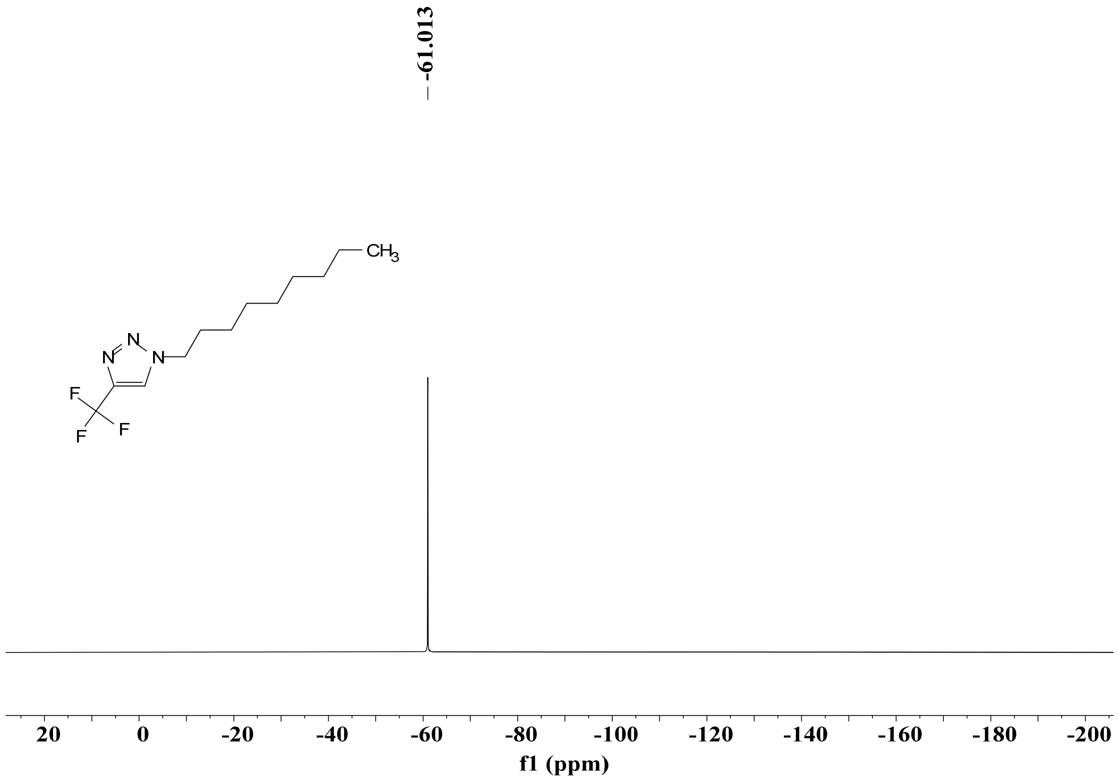

4b-^{19}F NMR

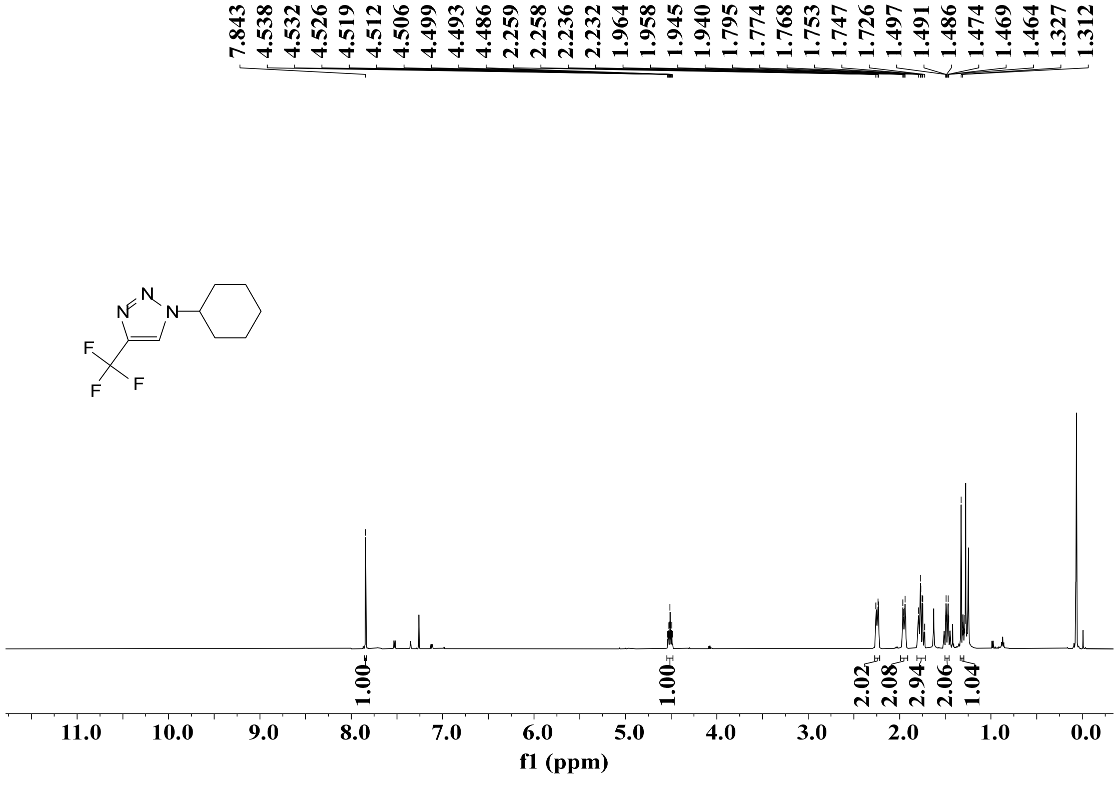

4c-^{1}H NMR

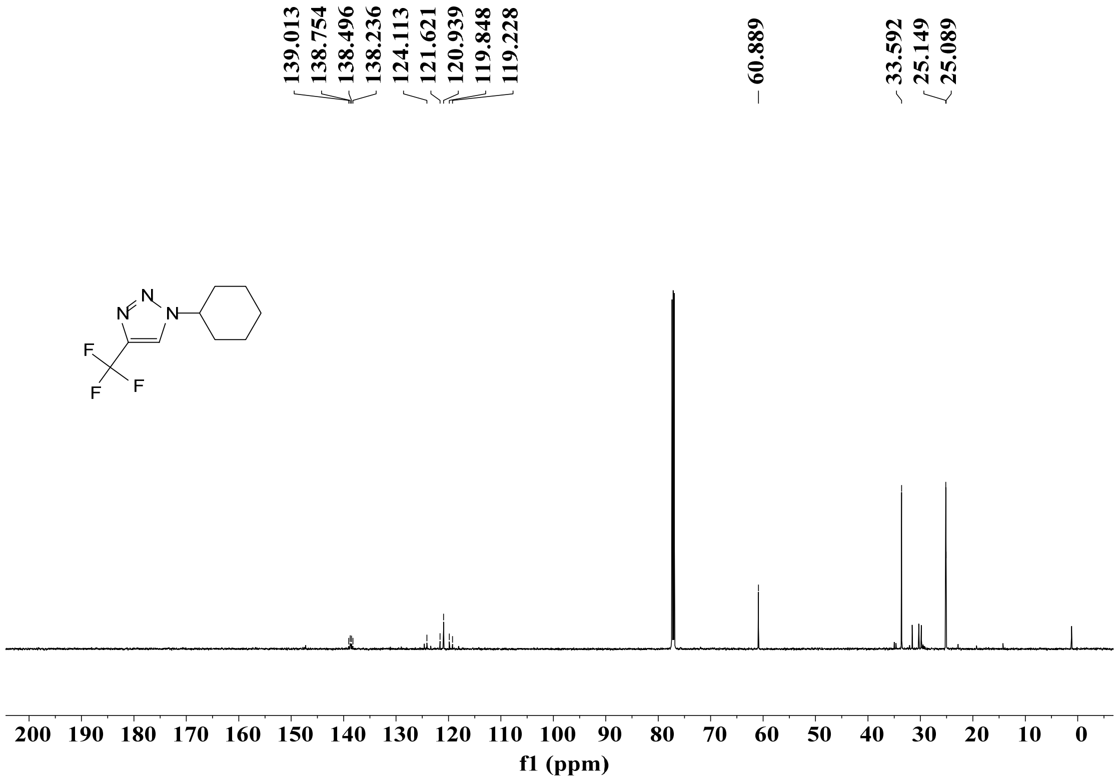
139.013
138.754
138.496
138.236
124.113
121.621
120.939
119.848
119.228
60.889
33.592
25.149
25.089
N
N
N
F
F
F
200 190 180 170 160 150 140 130 120 110 100 90 80 70 60 50 40 30 20 10 0
f1 (ppm)

4c-^{13}C NMR

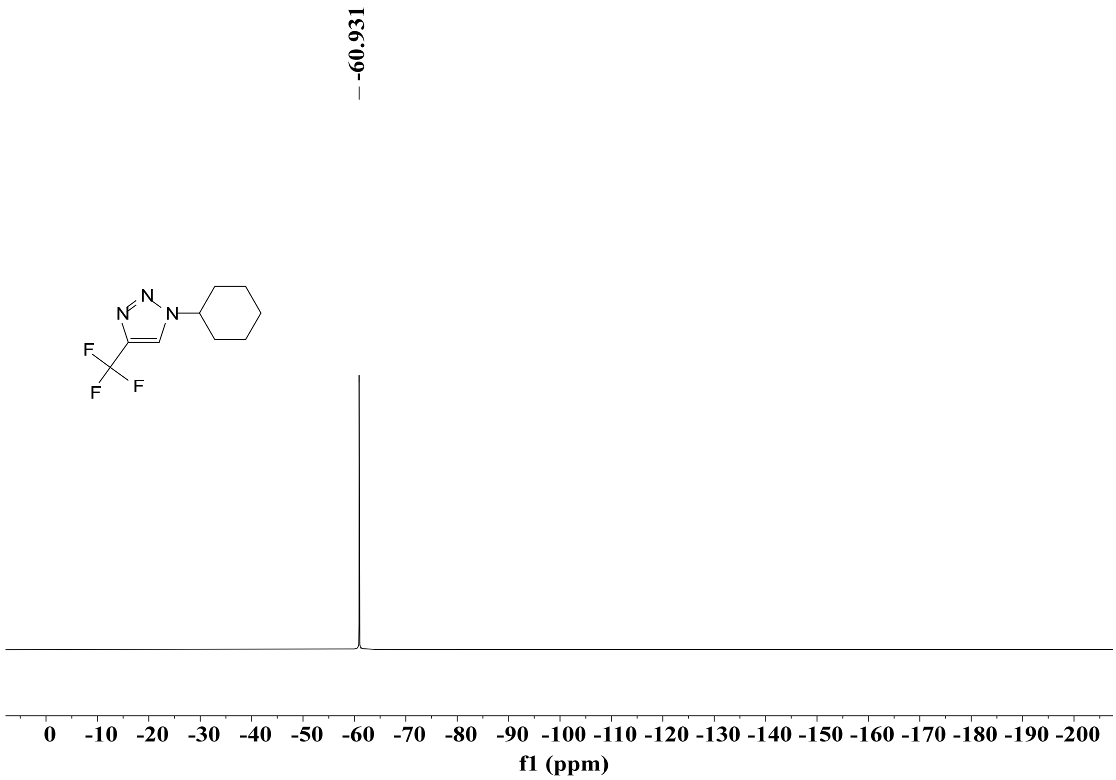

4c-^{19}F NMR

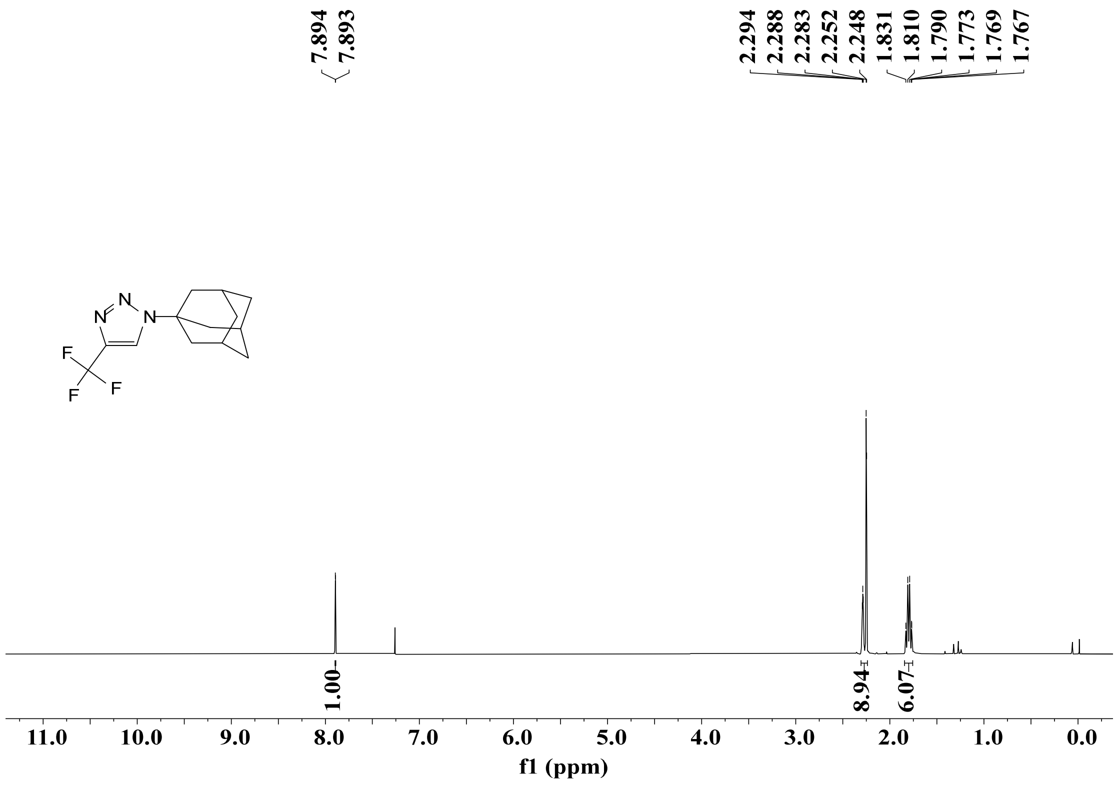

4d-^{1}H NMR

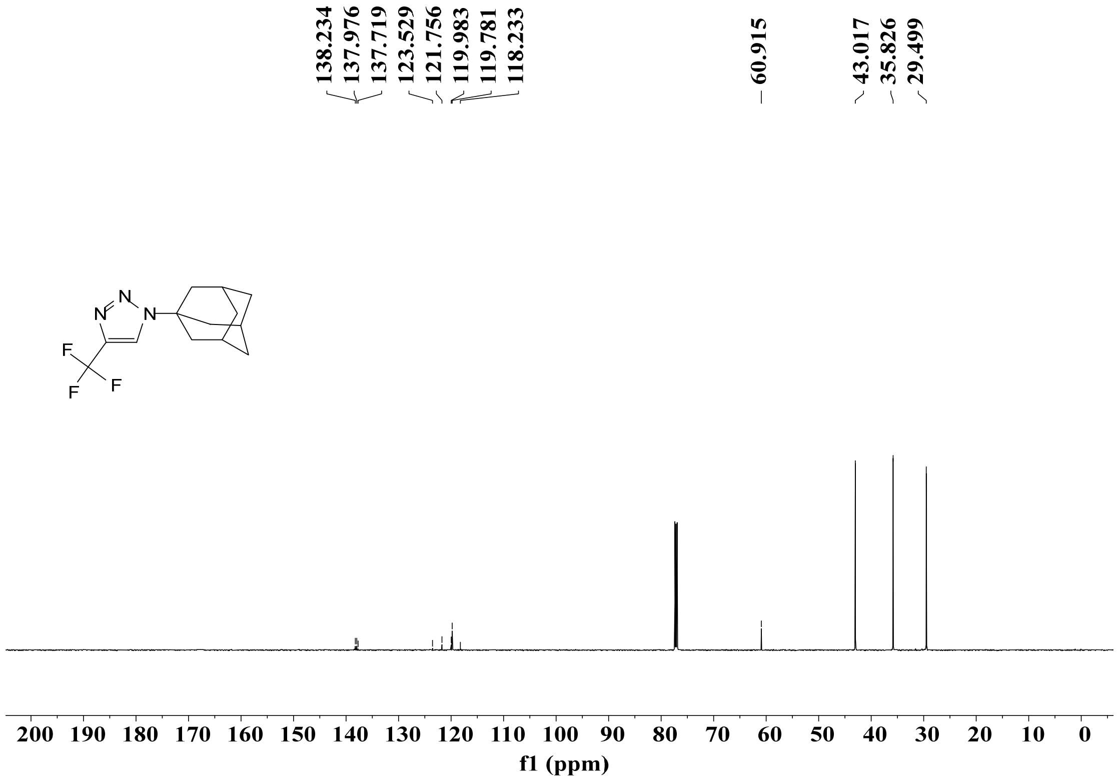
138.234
137.976
137.719
123.529
121.756
119.983
119.781
118.233
60.915
43.017
35.826
29.499
200 190 180 170 160 150 140 130 120 110 100 90 80 70 60 50 40 30 20 10 0
f1 (ppm)

4d-^{13}C NMR

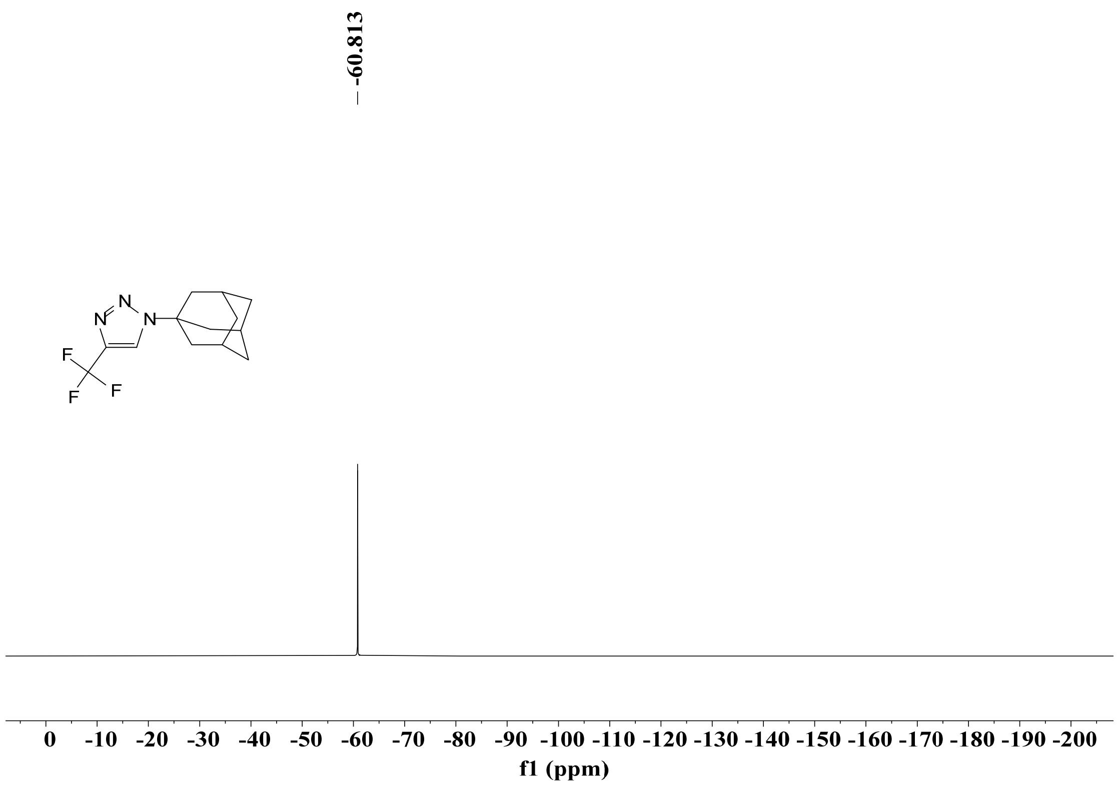
-60.813
0
-10
-20
-30
-40
-50
-60
-70
-80
-90
-100
-110
-120
-130
-140
-150
-160
-170
-180
-190
-200
f1 (ppm)

4d-^{19}F NMR

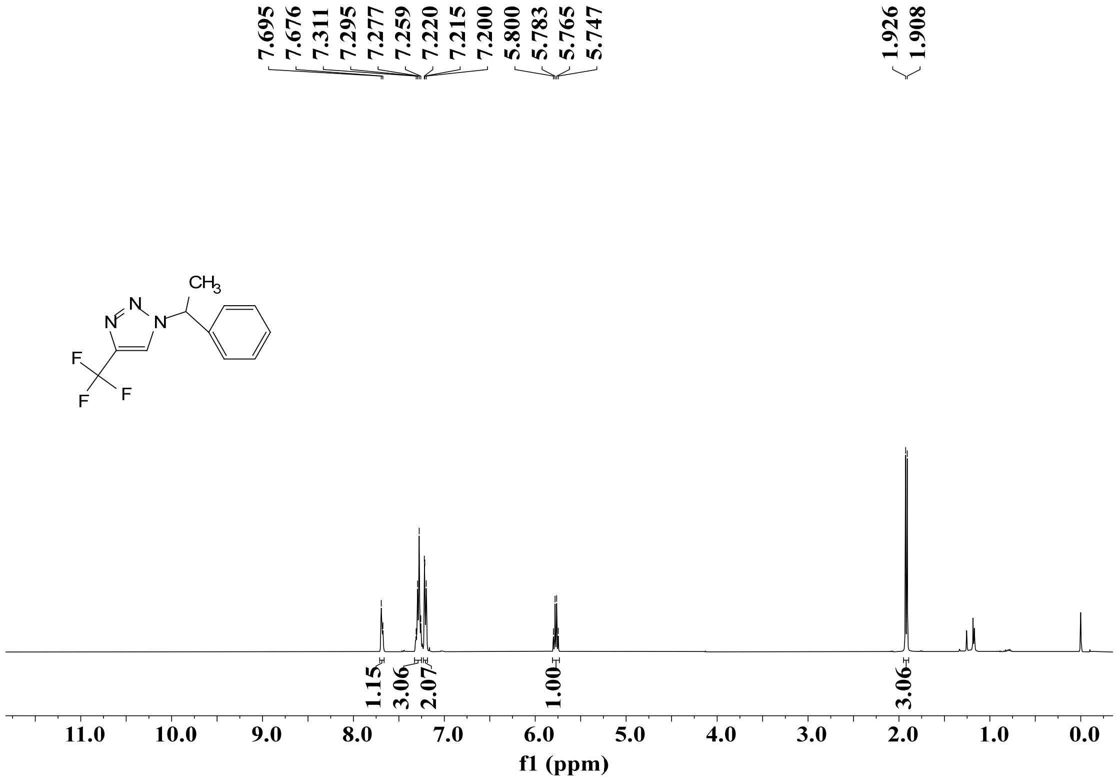

4e-¹H NMR

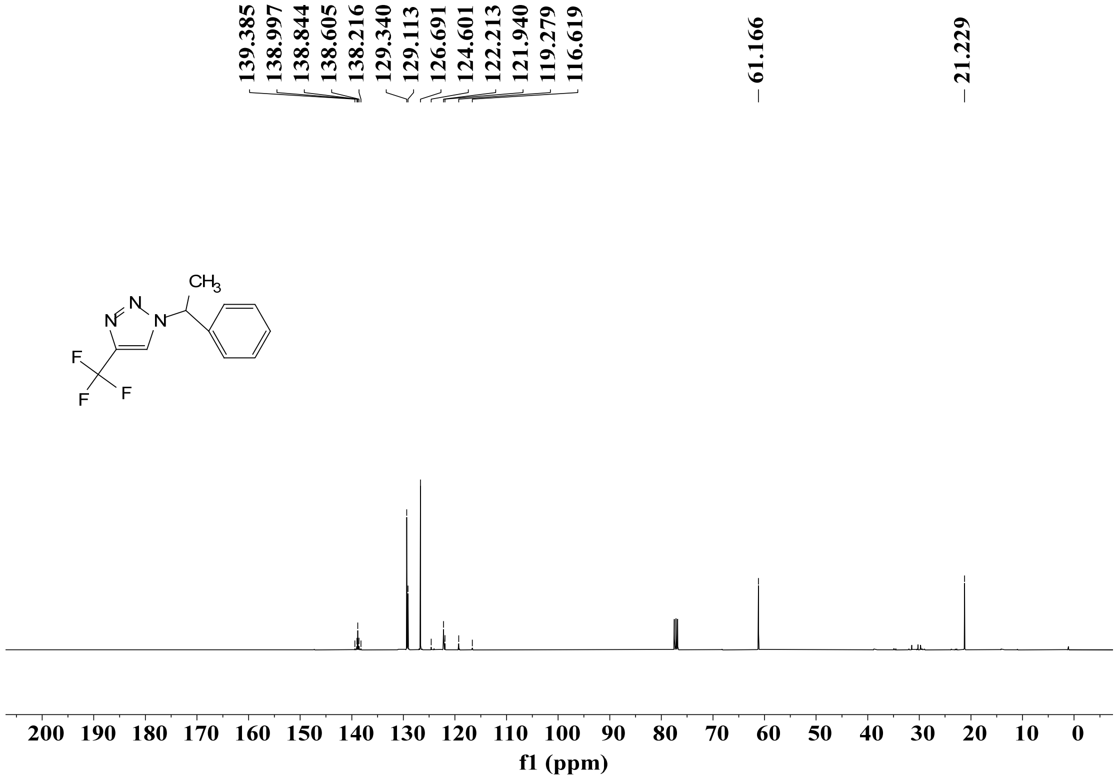

4e-¹³C NMR

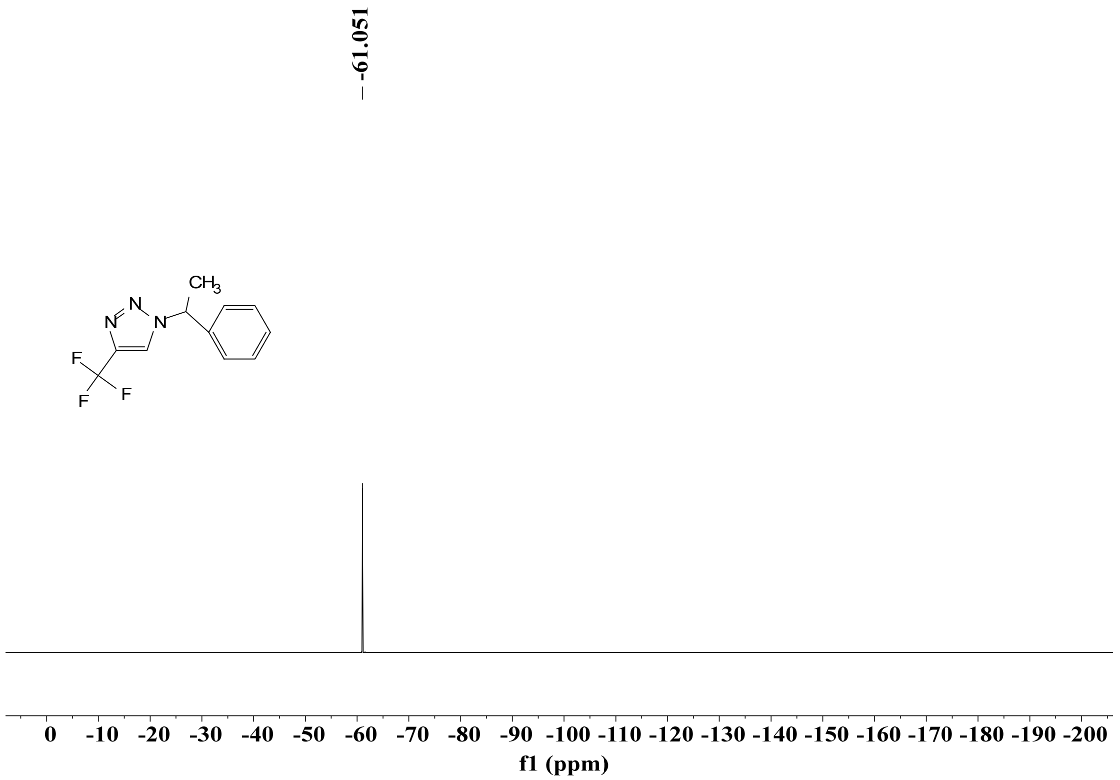

4e-^{19}F NMR

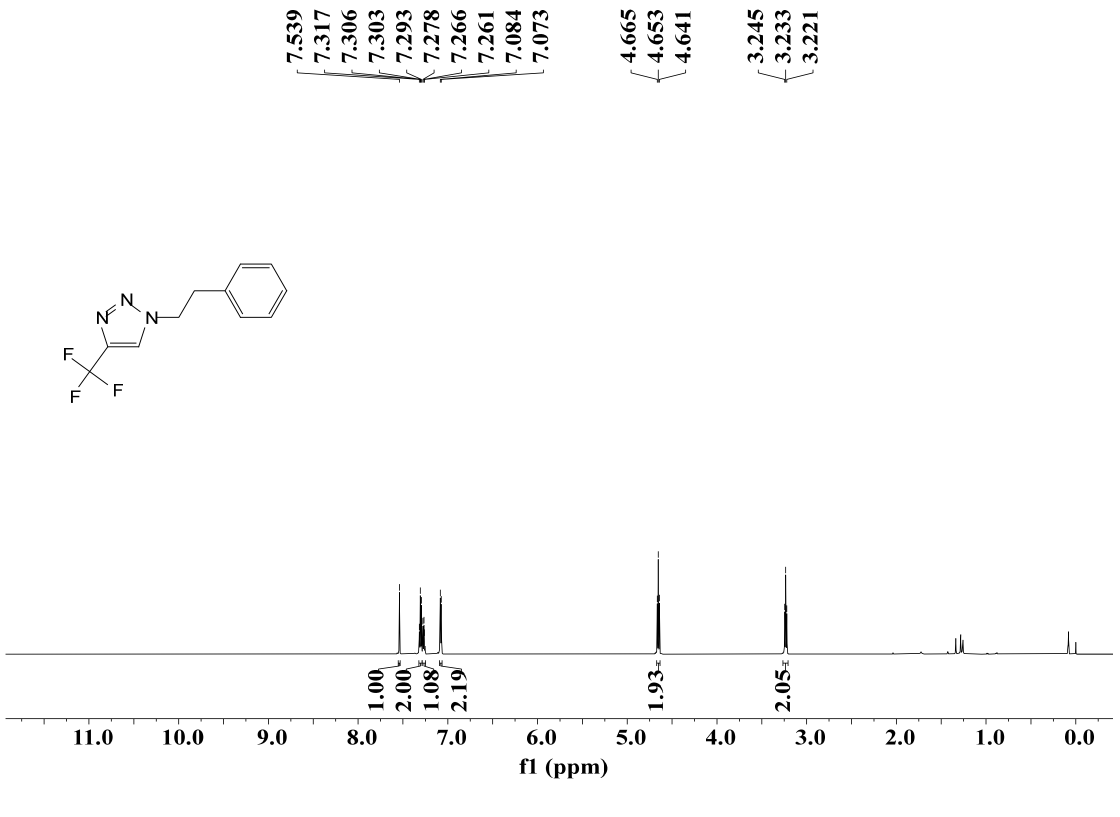

4f-^{1}H NMR

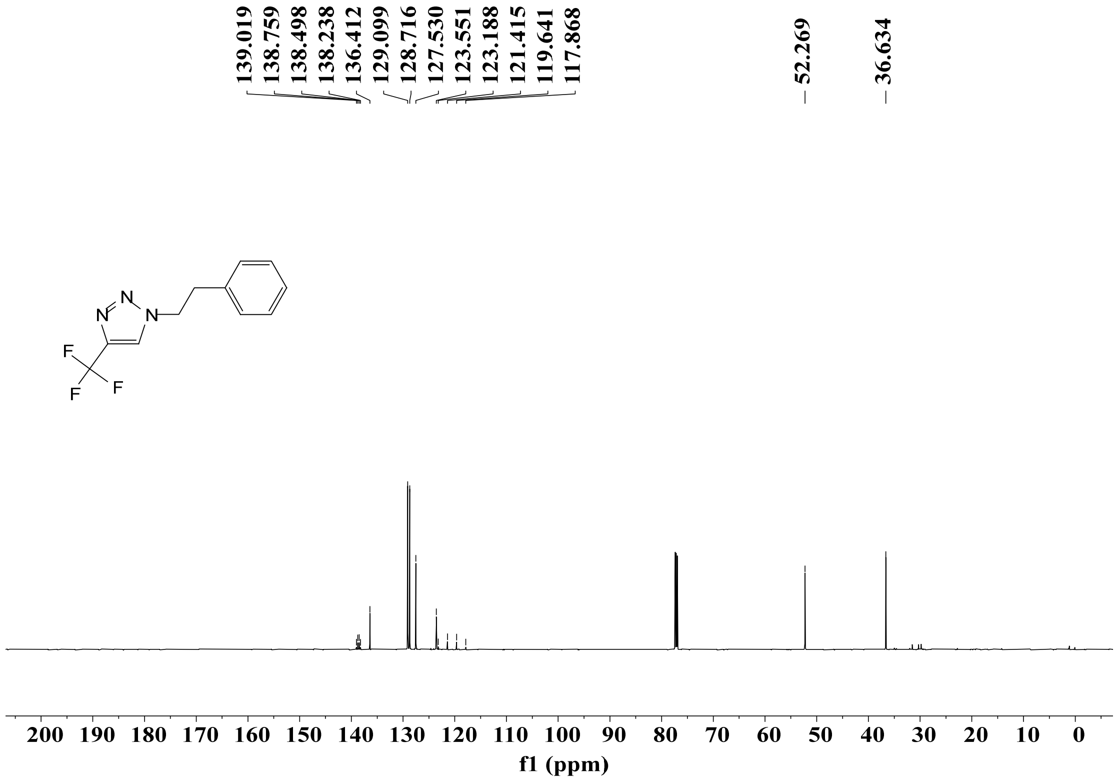
139.019
138.759
138.498
138.238
136.412
129.099
128.716
127.530
123.551
123.188
121.415
119.641
117.868
52.269
36.634
N
N
N
F
F
F
200 190 180 170 160 150 140 130 120 110 100 90 80 70 60 50 40 30 20 10 0
f1 (ppm)

4f-¹³C NMR

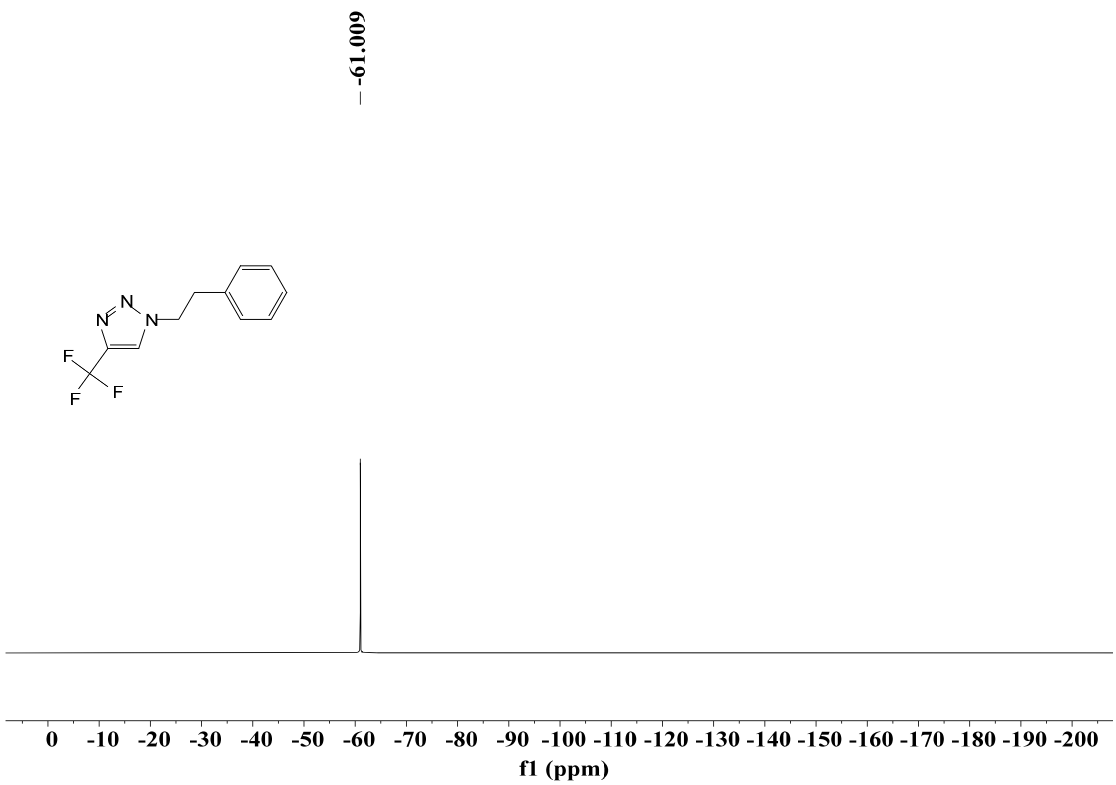

4f-^{19}F NMR

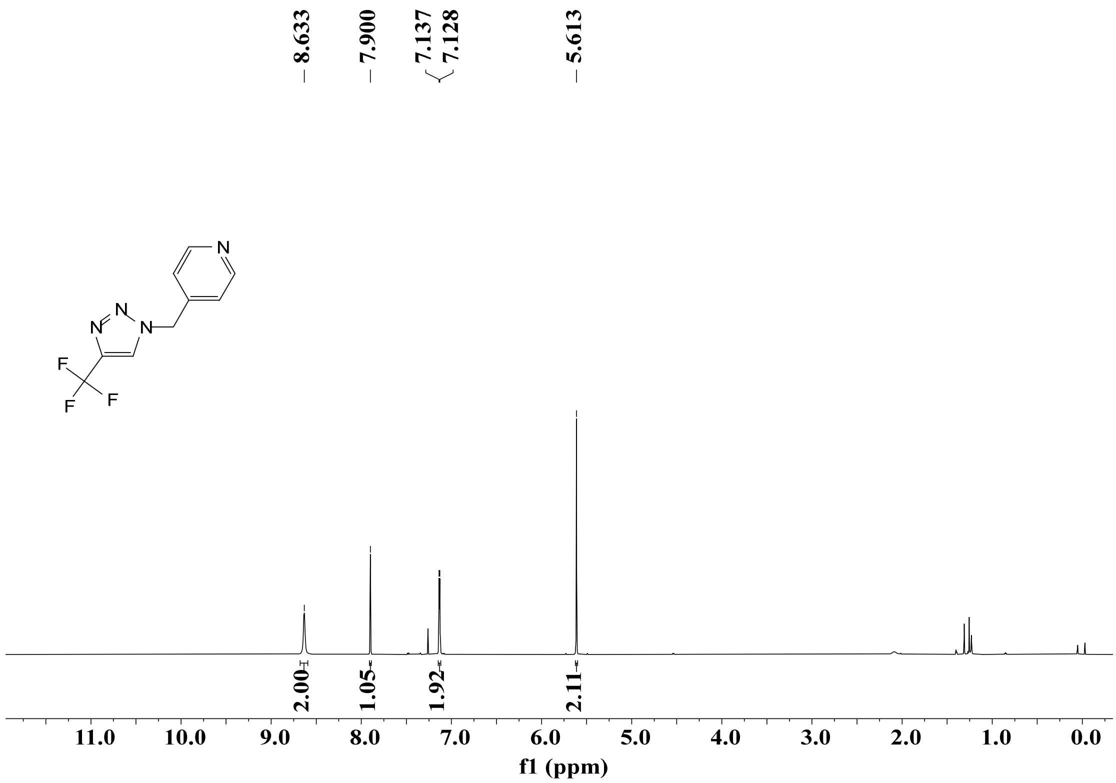

4g-^{1}H NMR

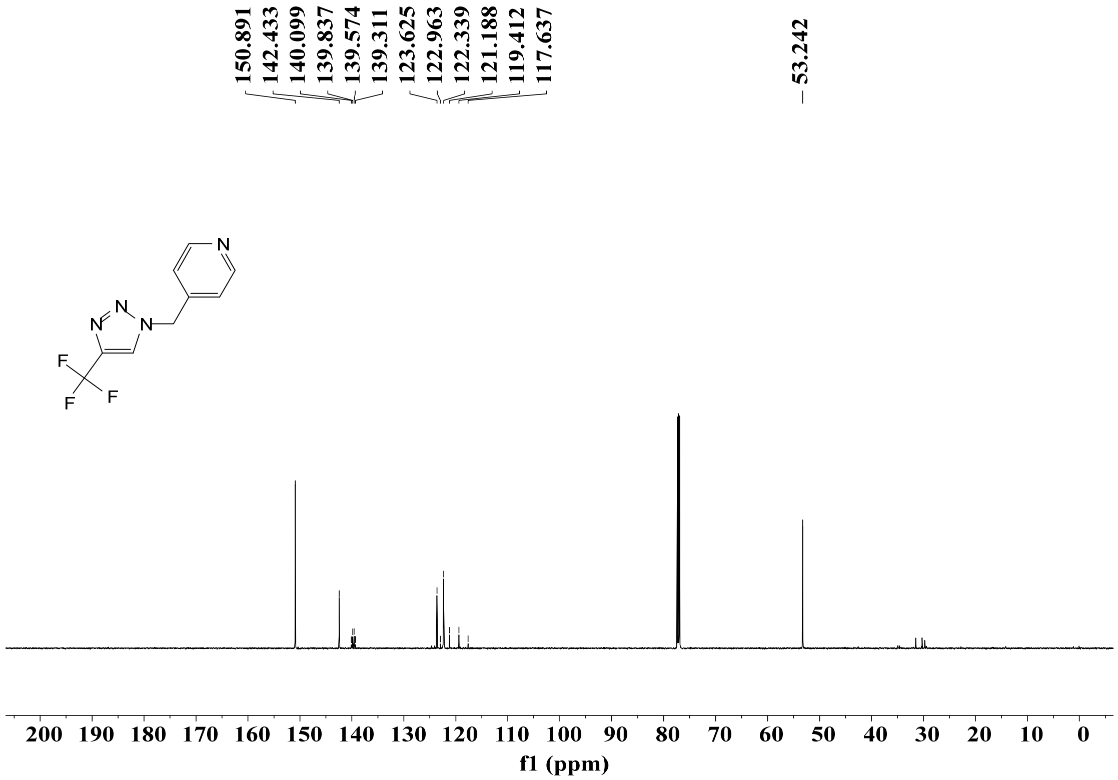
150.891
142.433
140.099
139.837
139.574
139.311
123.625
122.963
122.339
121.188
119.412
117.637
53.242
N
N
N
N
F
F
F
200 190 180 170 160 150 140 130 120 110 100 90 80 70 60 50 40 30 20 10 0
f1 (ppm)

4g-^{13}C NMR

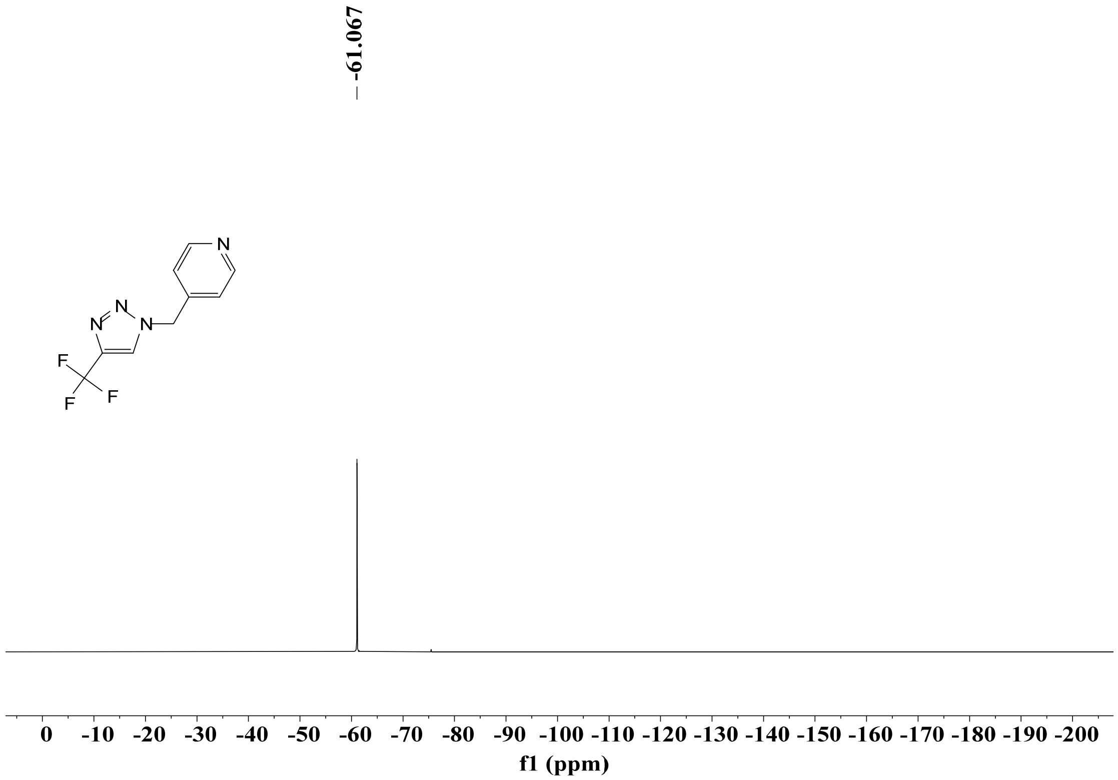

4g-^{19}F NMR

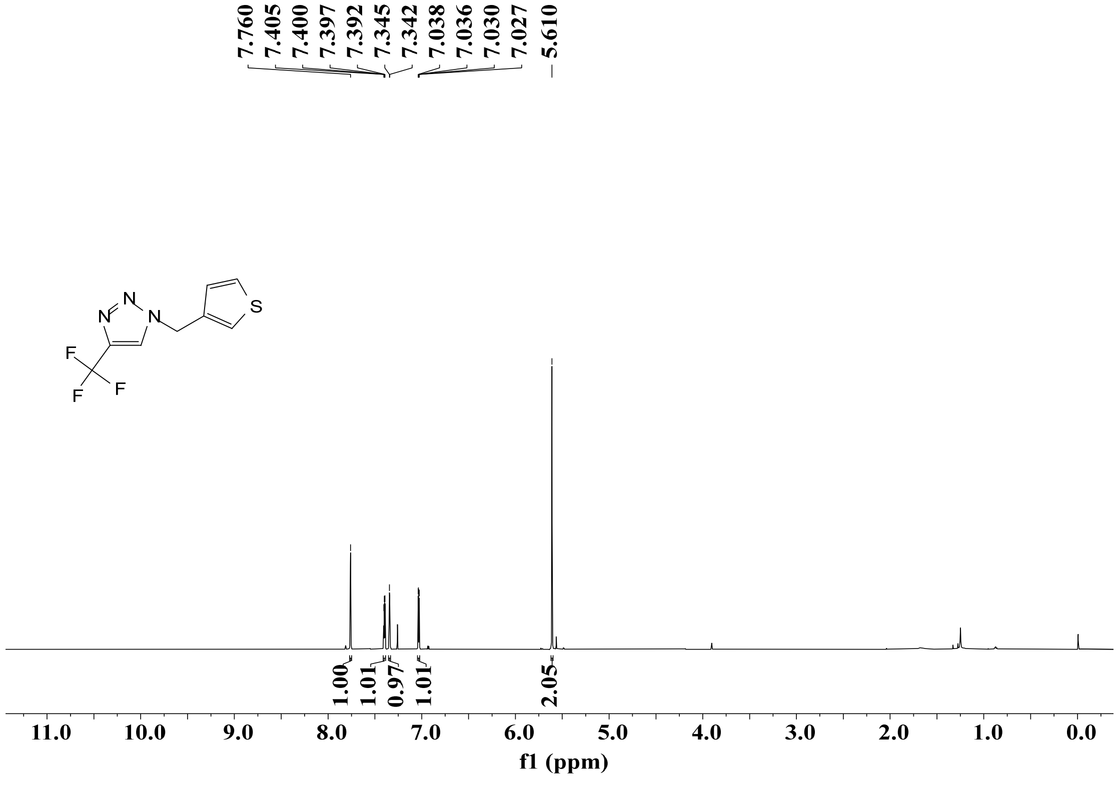

4h-^{1}H NMR

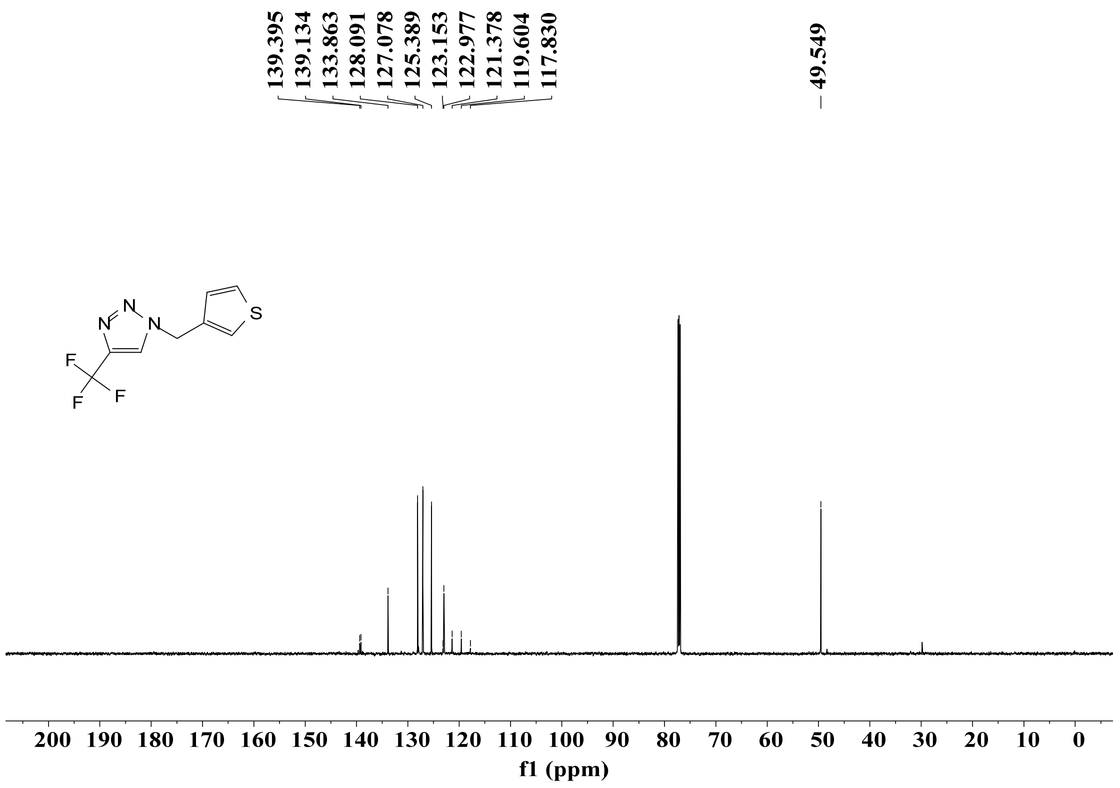
139.395
139.134
133.863
128.091
127.078
125.389
123.153
122.977
121.378
119.604
117.830
49.549
N
N
N
S
F
F
F
200 190 180 170 160 150 140 130 120 110 100 90 80 70 60 50 40 30 20 10 0
f1 (ppm)

4h-^{13}C NMR

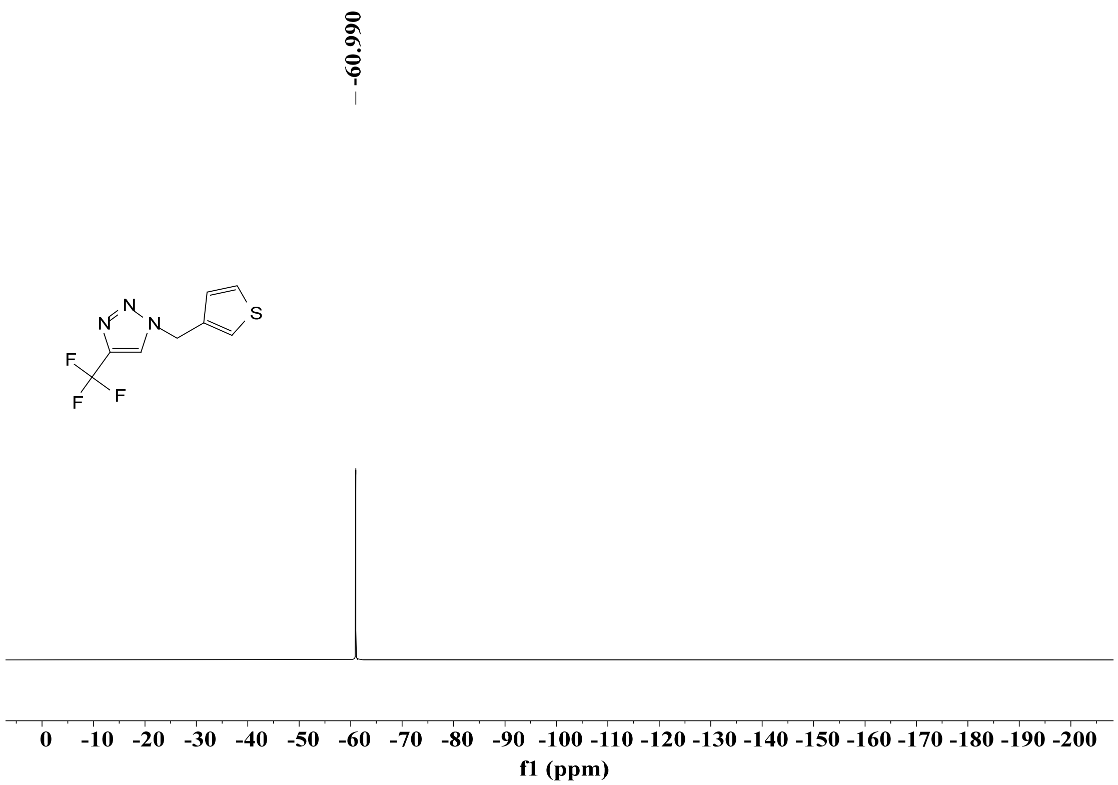

4h-^{19}F NMR

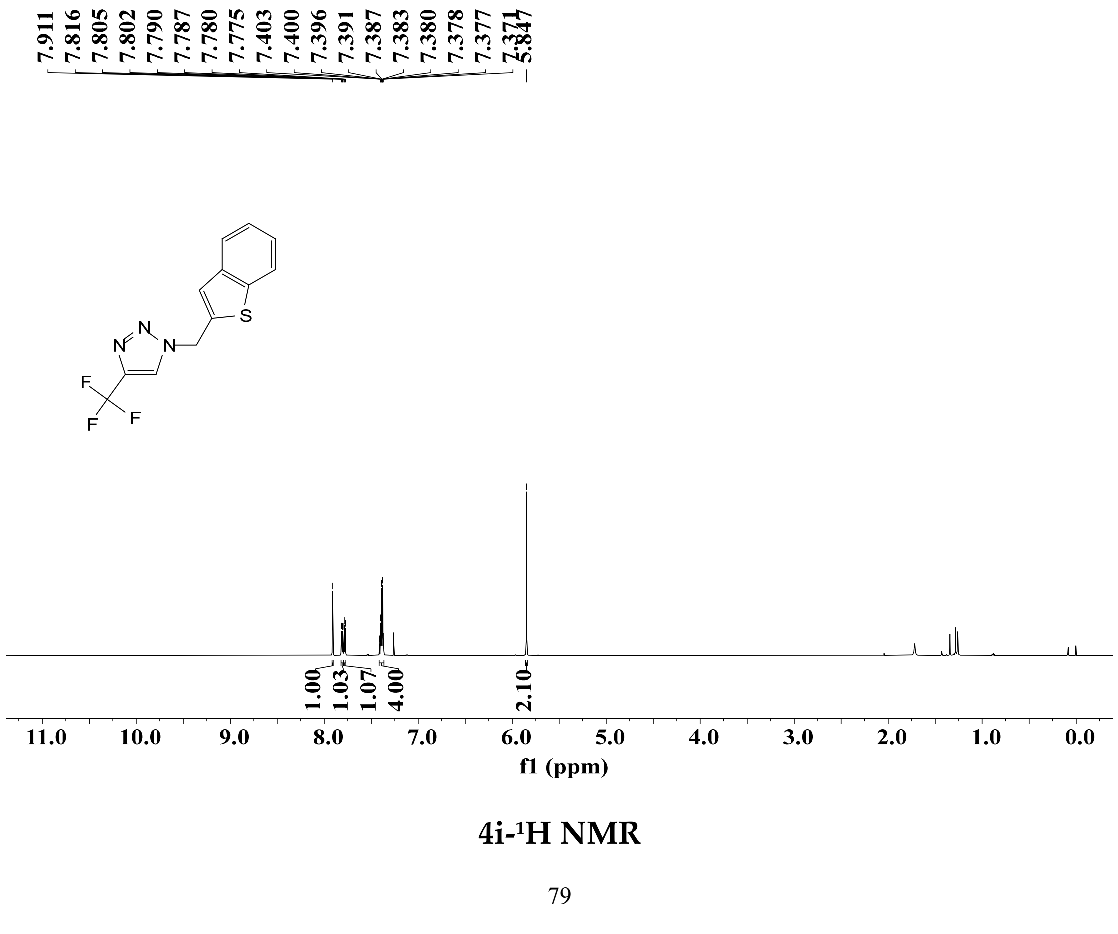

4i-^{1}H NMR

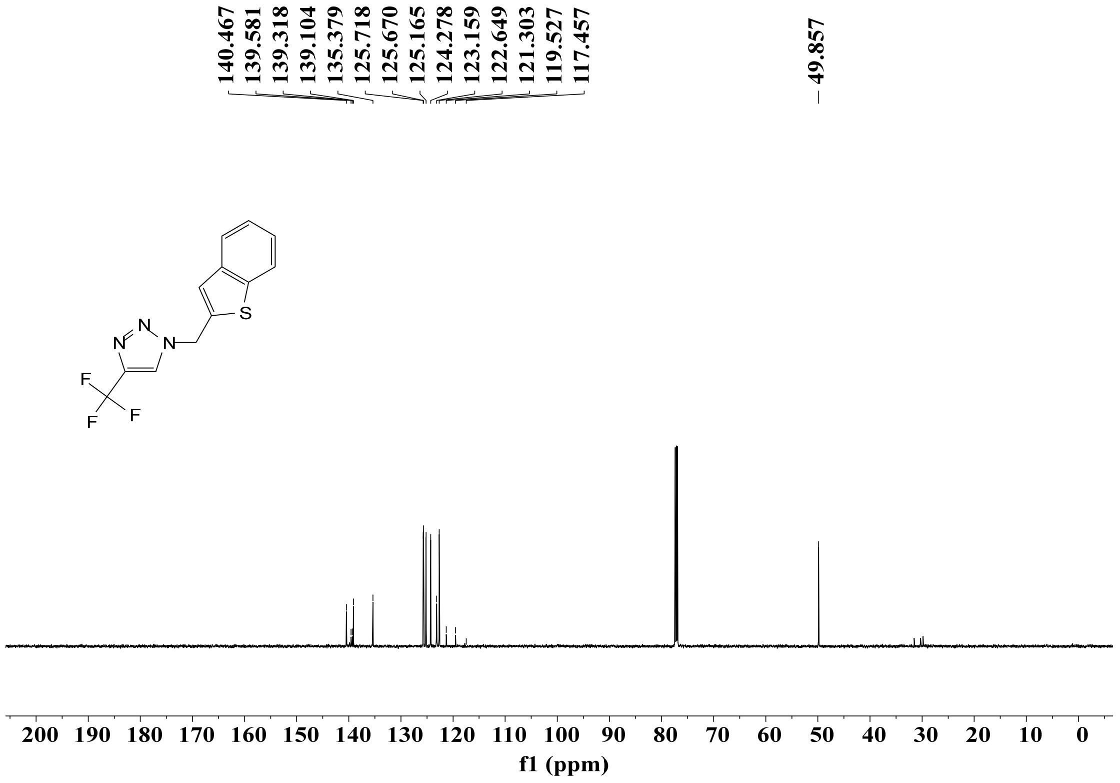
140.467
139.581
139.318
139.104
135.379
125.718
125.670
125.165
124.278
123.159
122.649
121.303
119.527
117.457
49.857
N
N
N
S
F
F
F
200 190 180 170 160 150 140 130 120 110 100 90 80 70 60 50 40 30 20 10 0
f1 (ppm)

4i-^{13}C NMR

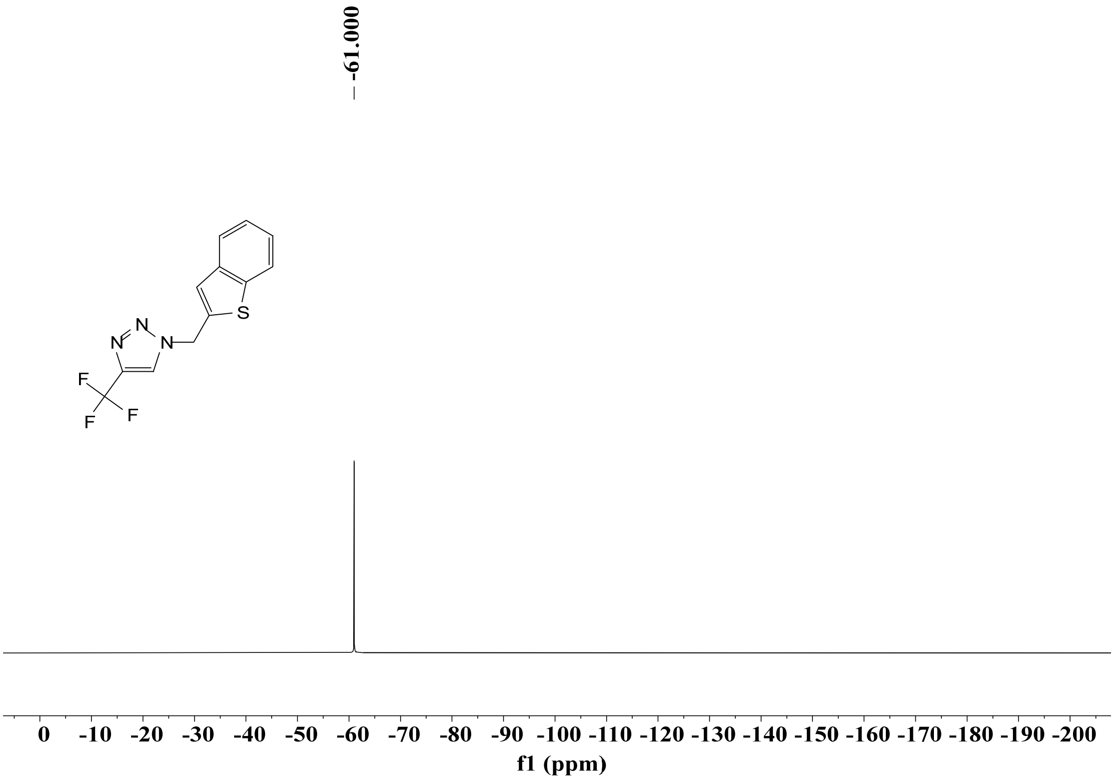

4i-^{19}F NMR

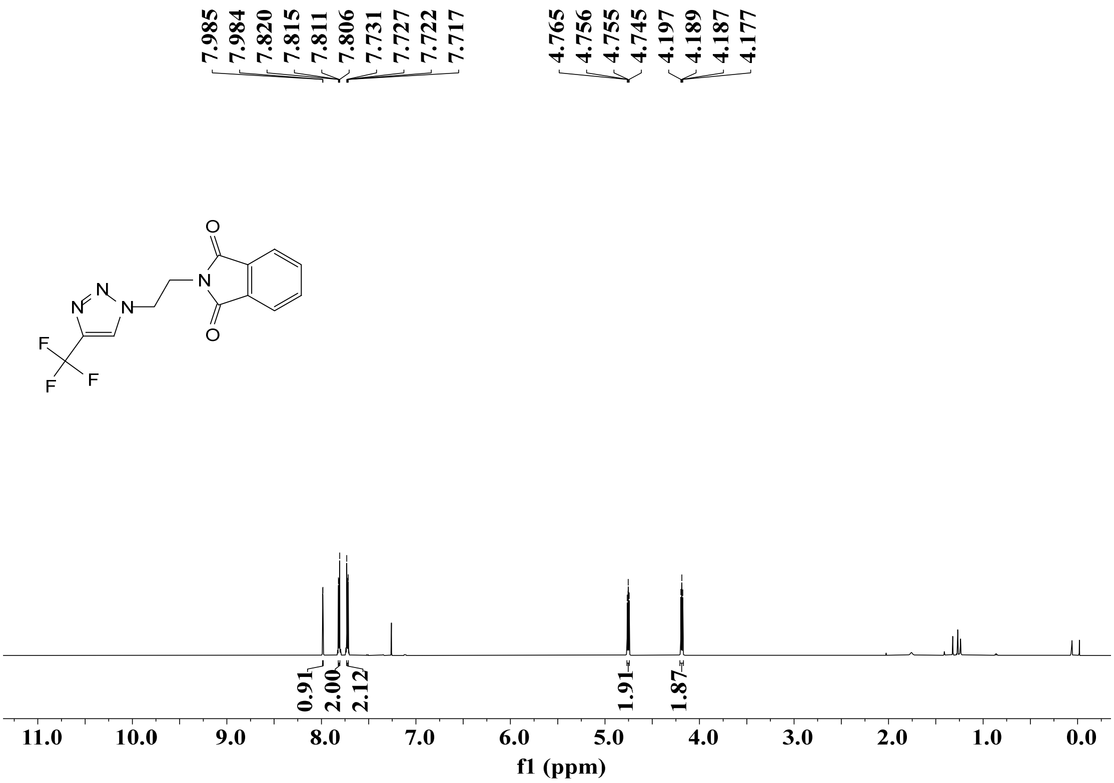

4j-^{1}H NMR

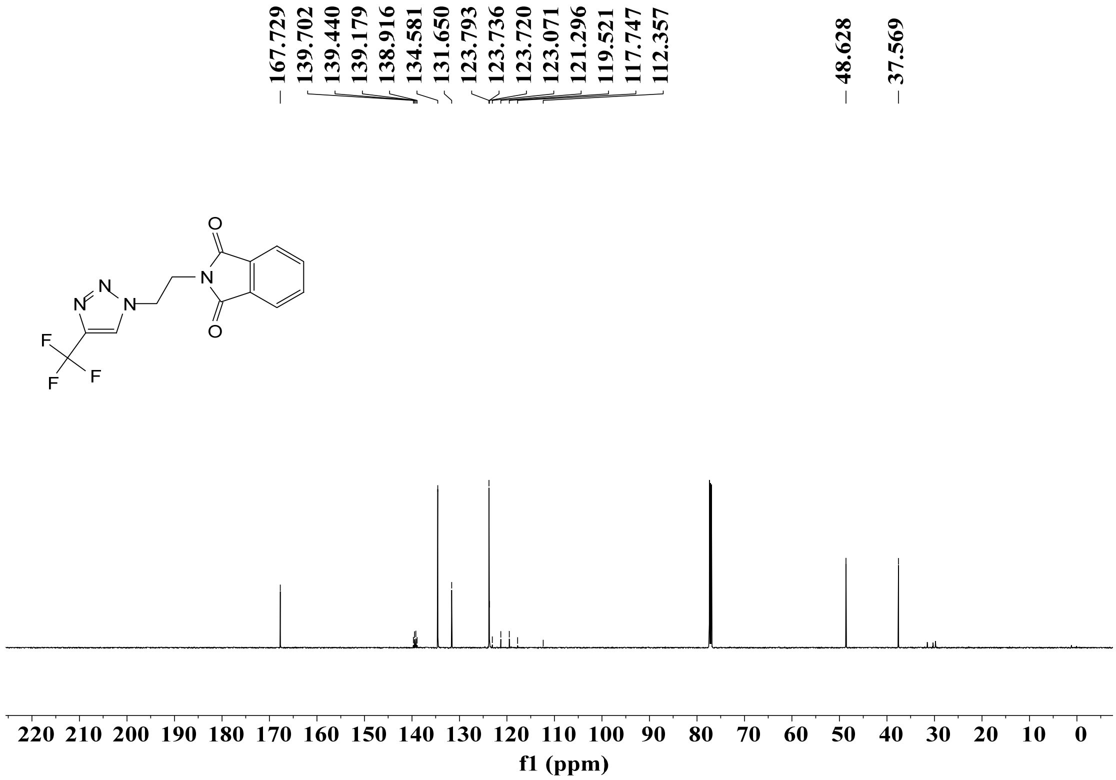
167.729
139.702
139.440
139.179
138.916
134.581
131.650
123.793
123.736
123.720
123.071
121.296
119.521
117.747
112.357
48.628
37.569
O
N
O
N
N
N
F
F
F
220 210 200 190 180 170 160 150 140 130 120 110 100 90 80 70 60 50 40 30 20 10 0
f1 (ppm)

4j-^{13}C NMR

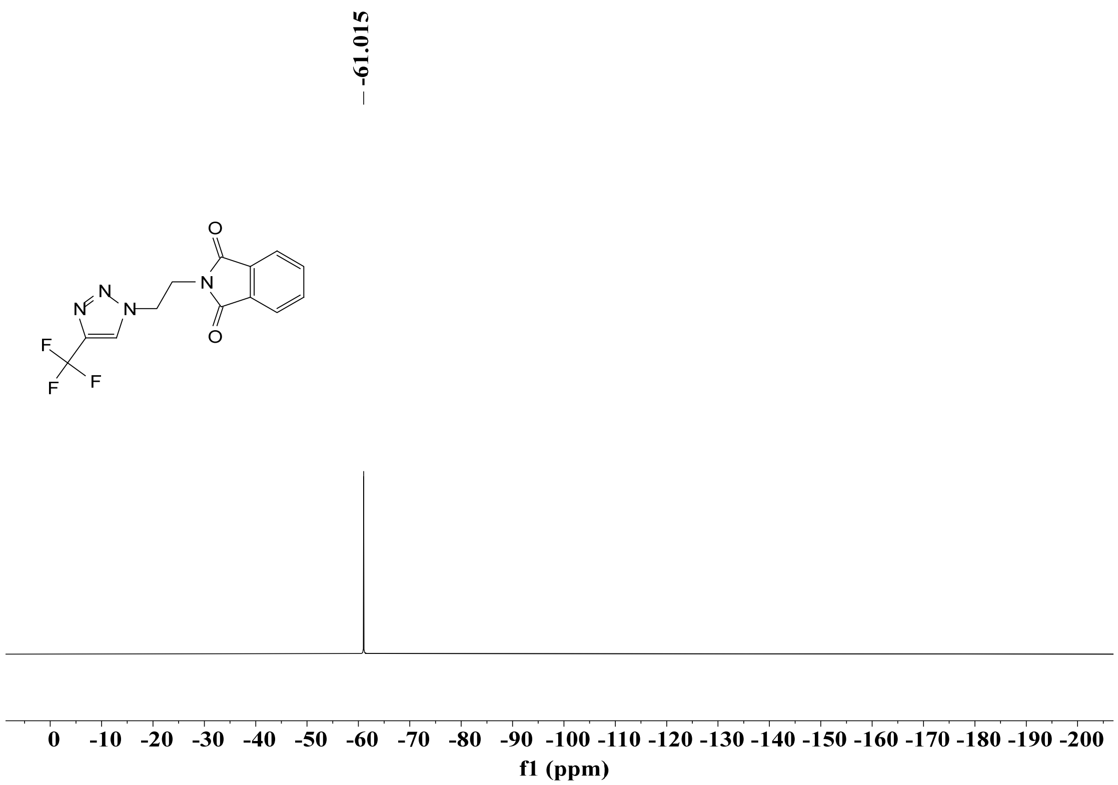

4j-^{19}F NMR

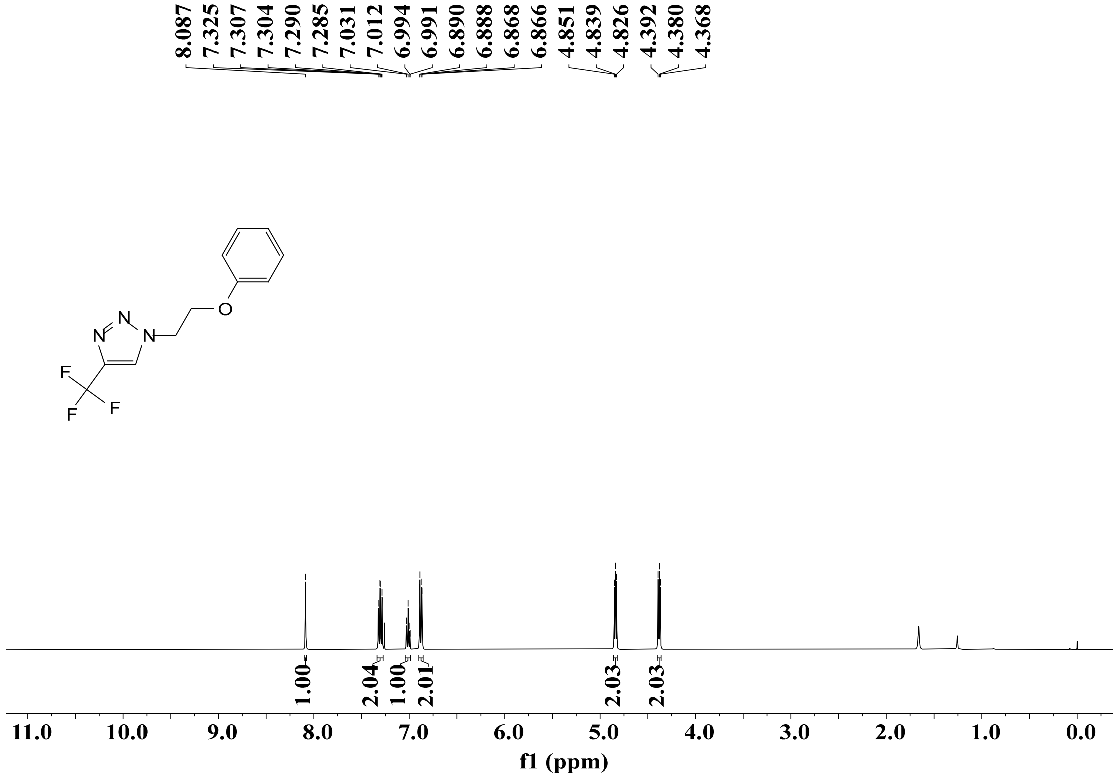

4k-^{1}H NMR

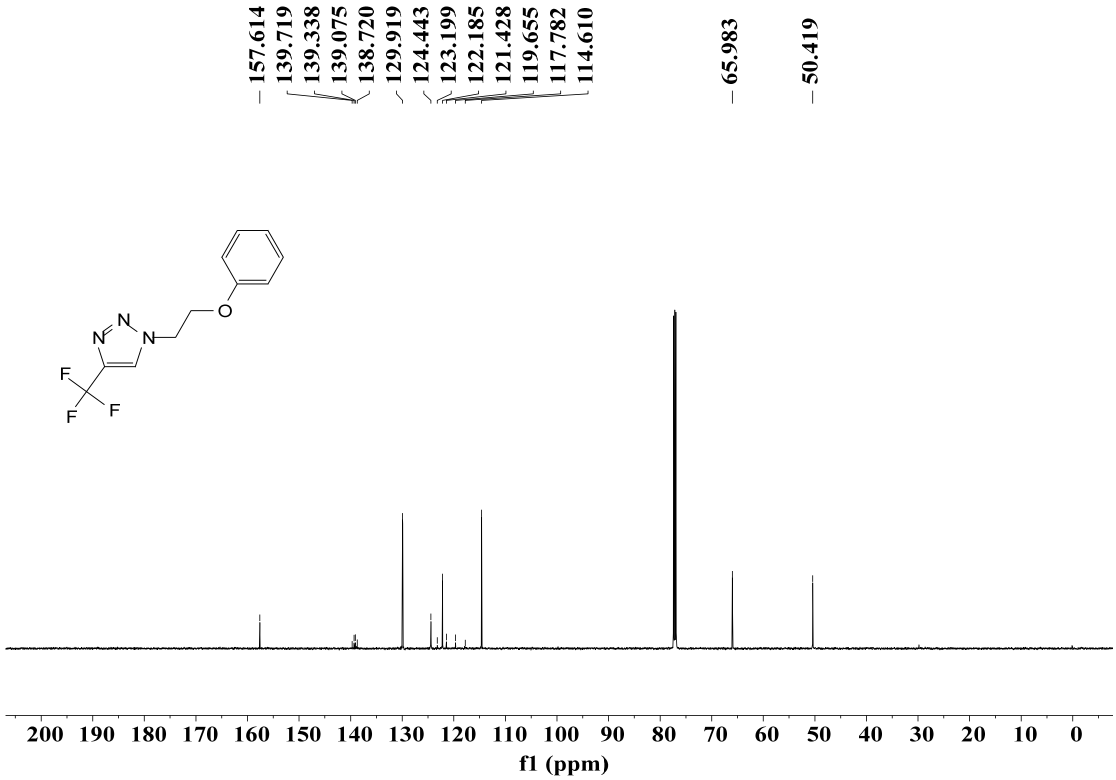
157.614
139.719
139.338
139.075
138.720
129.919
124.443
123.199
122.185
121.428
119.655
117.782
114.610
65.983
50.419
N
N
N
O
F
F
F
200 190 180 170 160 150 140 130 120 110 100 90 80 70 60 50 40 30 20 10 0
f1 (ppm)

4k-^{13}C NMR

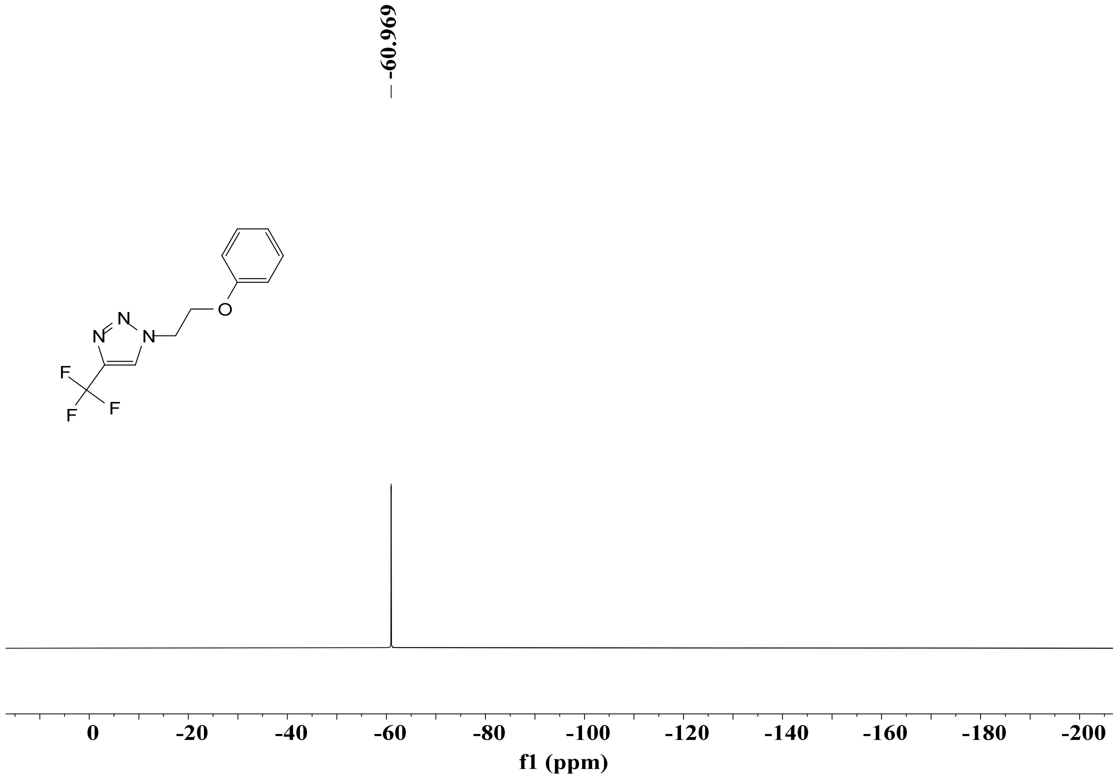
-60.969
N
N
N
O
F
F
F
0 -20 -40 -60 -80 -100 -120 -140 -160 -180 -200
f1 (ppm)

4k-^{19}F NMR

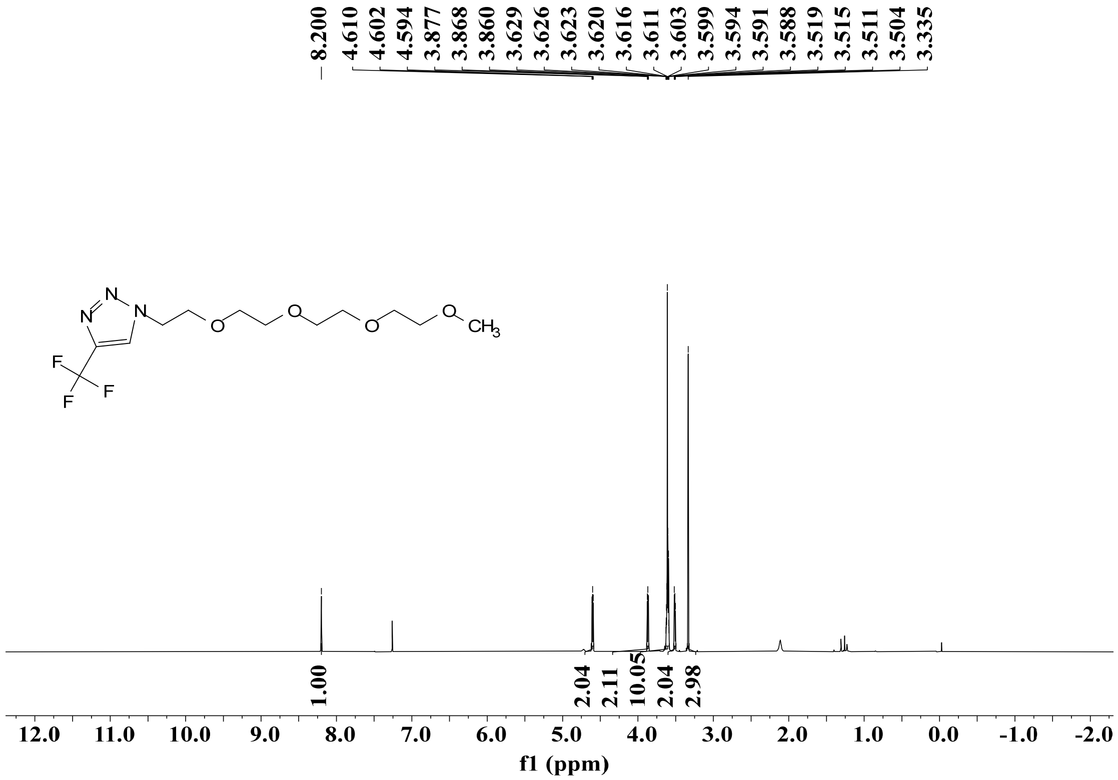

4l-^{1}H NMR

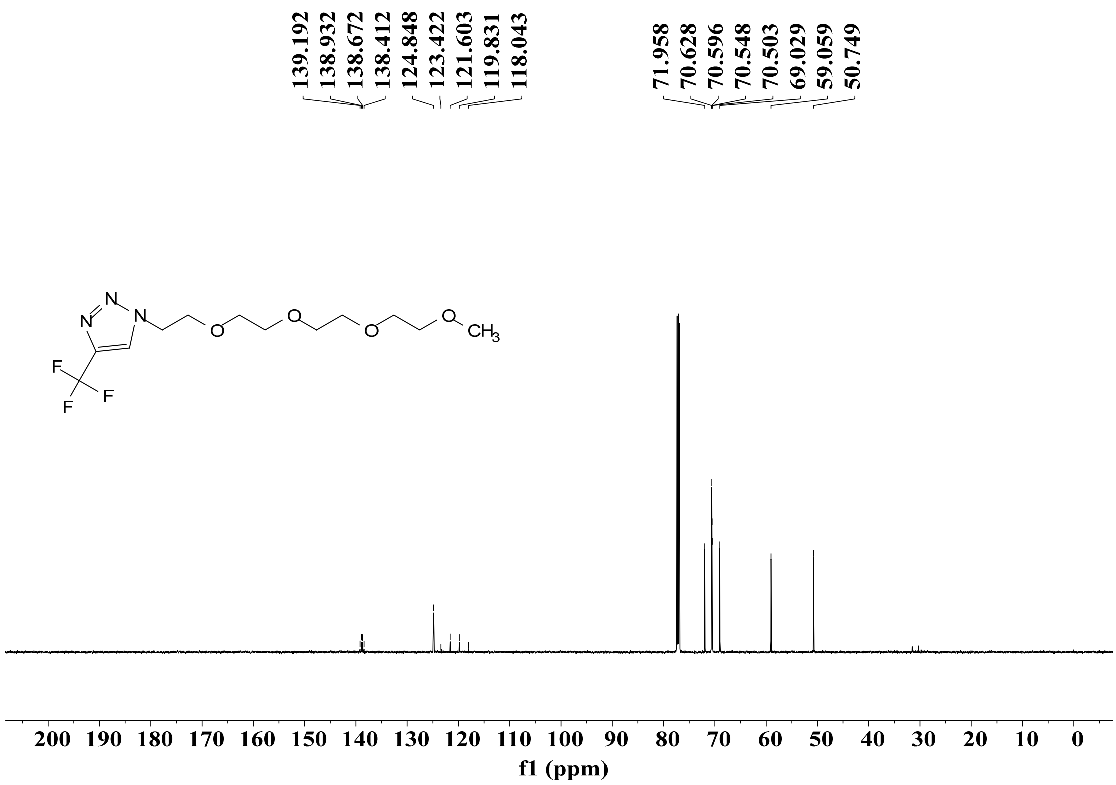

4l-^{13}C NMR

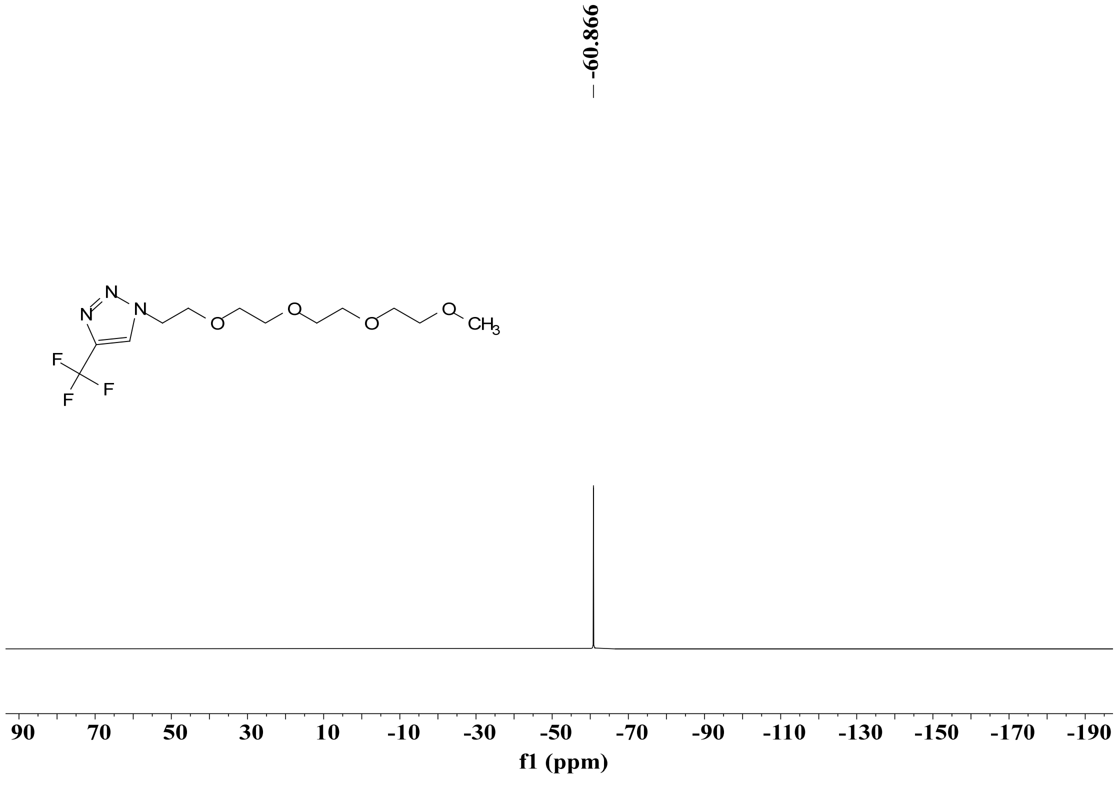

4l-^{19}F NMR

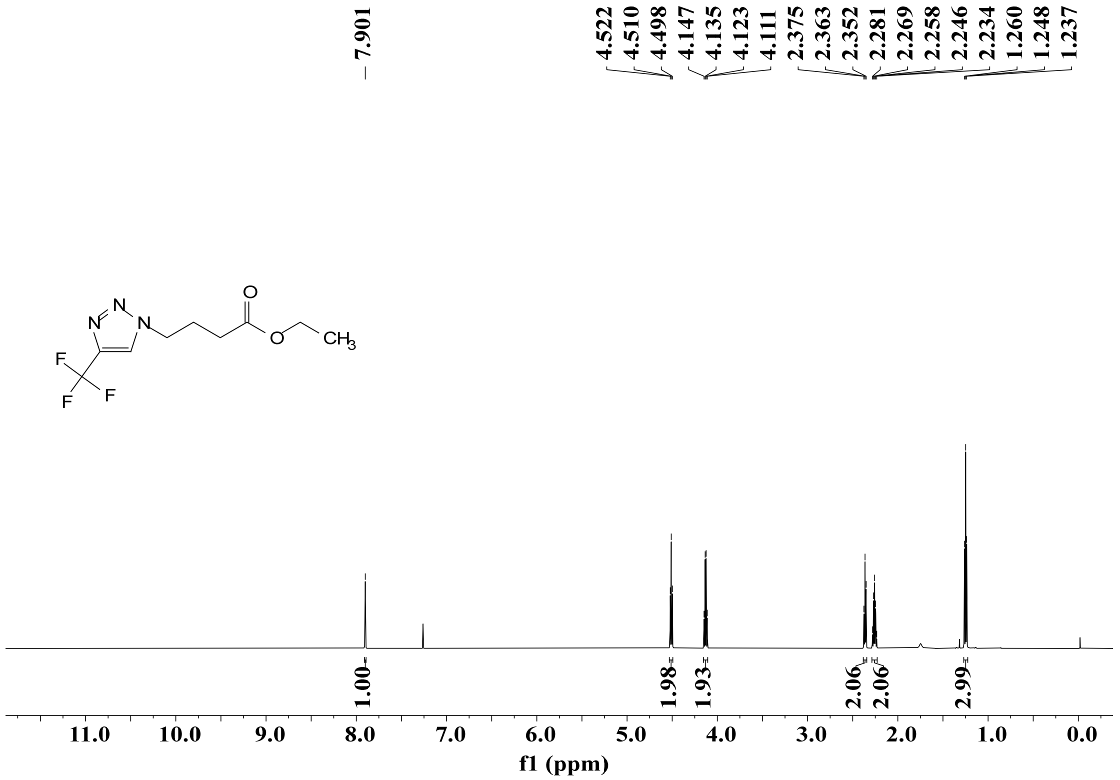

7.901
4.522
4.510
4.498
4.147
4.135
4.123
4.111
2.375
2.363
2.352
2.281
2.269
2.258
2.246
2.234
1.260
1.248
1.237
1.00
1.98
1.93
2.06
2.06
2.99
11.0
10.0
9.0
8.0
7.0
6.0
5.0
4.0
3.0
2.0
1.0
0.0
f1 (ppm)

4m-1H NMR

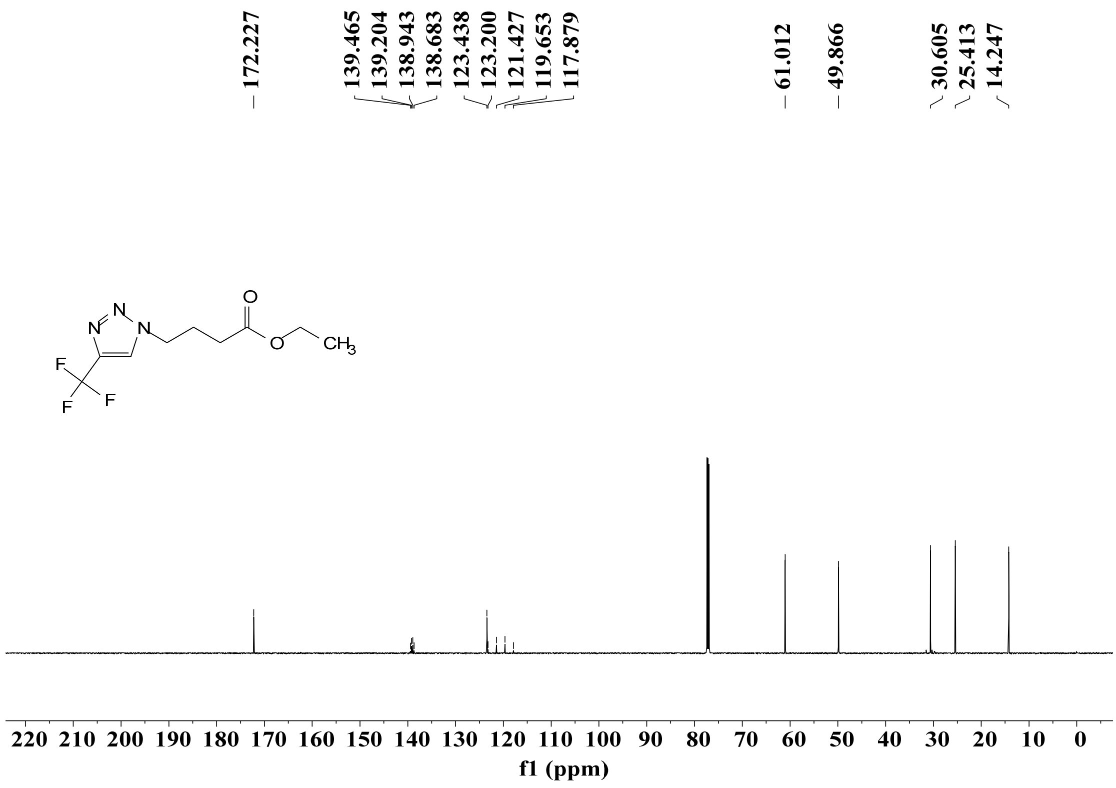

4m-^{13}C NMR

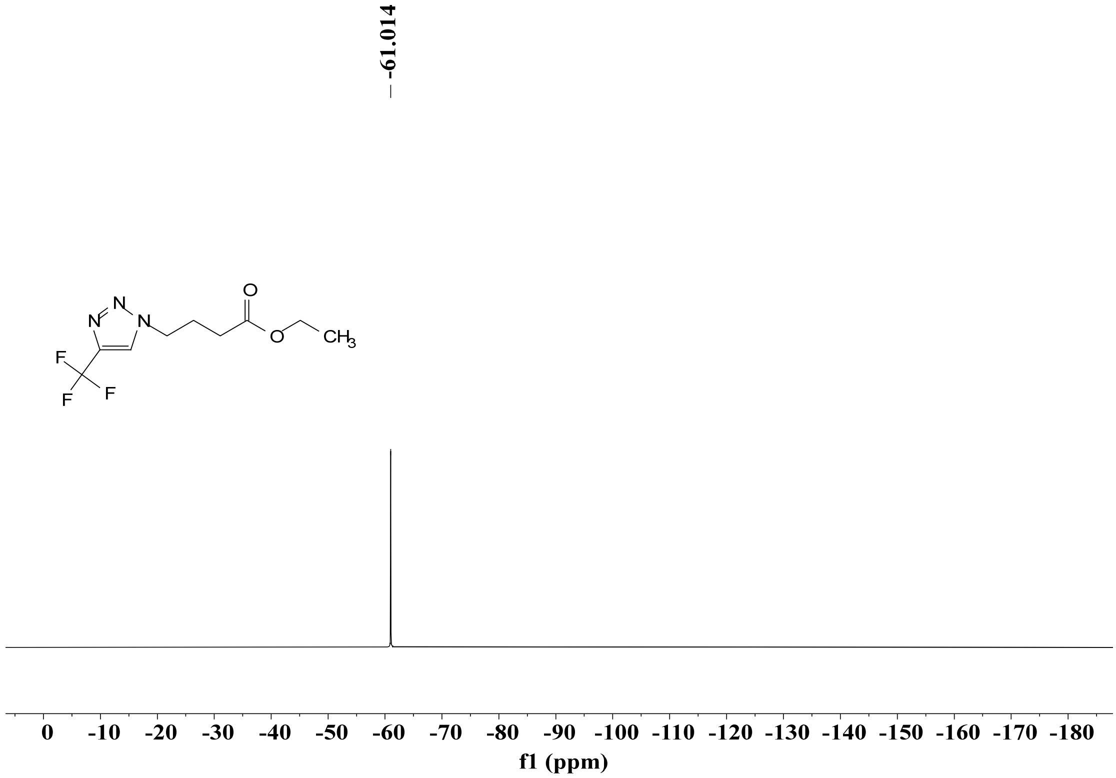

4m-^{19}F NMR

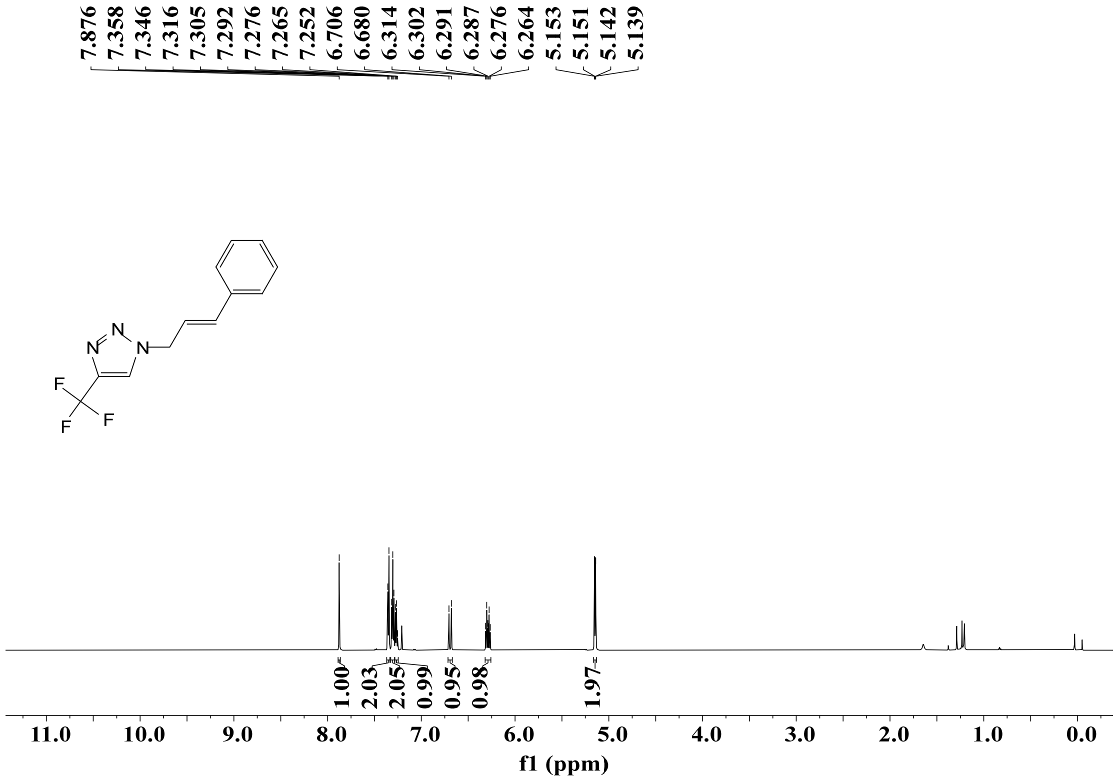
7.876
7.358
7.346
7.316
7.305
7.292
7.276
7.265
7.252
6.706
6.680
6.314
6.302
6.291
6.287
6.276
6.264
5.153
5.151
5.142
5.139
N
N
N
F
F
F
1.00
2.03
2.05
0.99
0.95
0.98
1.97
11.0 10.0 9.0 8.0 7.0 6.0 5.0 4.0 3.0 2.0 1.0 0.0
f1 (ppm)

4n-¹H NMR

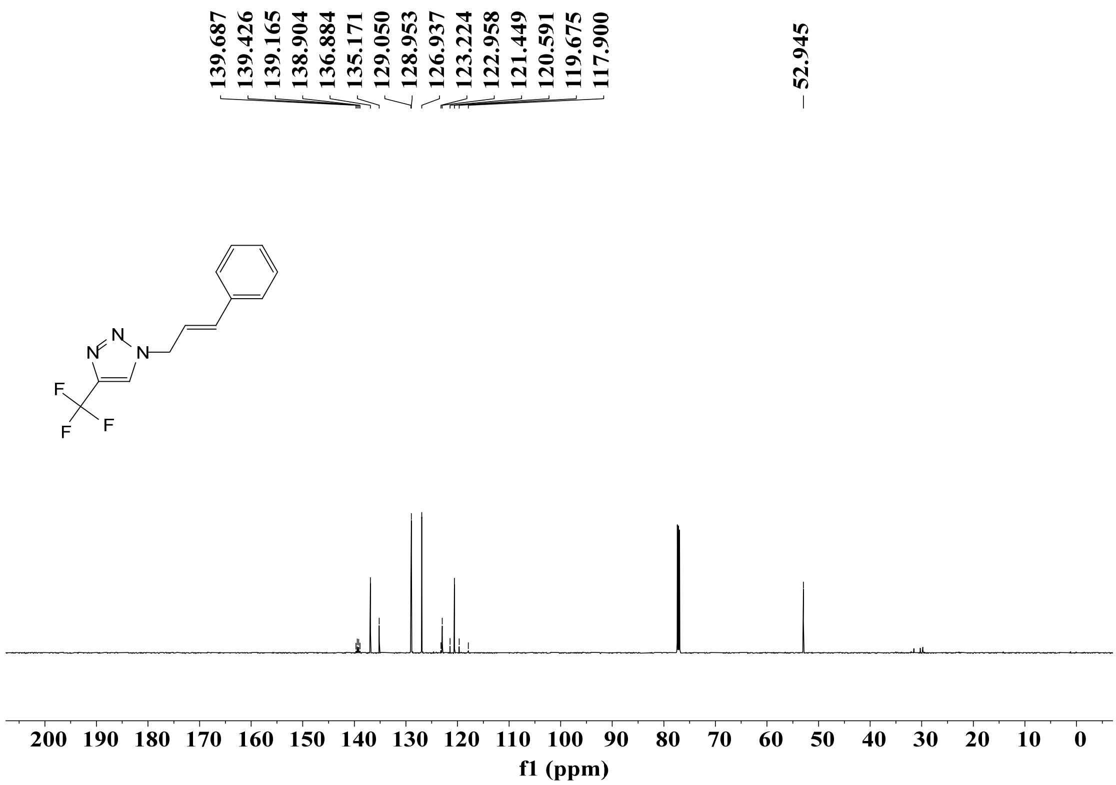

4n-^{13}C NMR

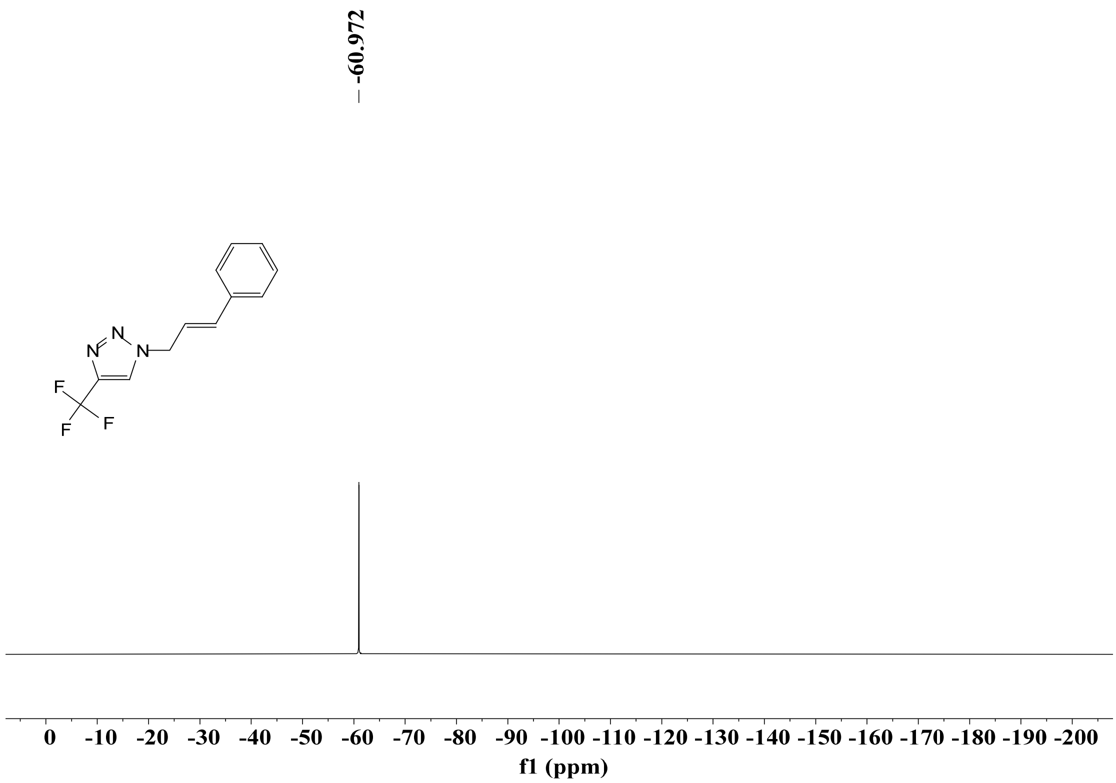

4n-^{19}F NMR
